# Supplementary material for: Recognition of anion-water clusters by peptide-based supramolecular capsules
Source: Nat Commun. 2024 Jul 18;15:6055. doi: 10.1038/s41467-024-50193-4 (PMC11258365; doi:10.1038/s41467-024-50193-4)
Supplement: Supplementary file 1 — Supplementary Information [file 41467_2024_50193_MOESM1_ESM.pdf]

Recognition of anion-water clusters by peptide-based supramolecular capsules

Victoria López-Corbalán[1], Alberto Fuertes[1], Antonio L. Llamas-Saiz[2], Manuel Amorín[1], & Juan R. Granja[1]*
[1] Centro Singular de Investigación en Química Biolóxica e Materiais Moleculares (CIQUS) and Organic Chemistry Department, Universidade de Santiago de Compostela, and [2] Unidad de Rayos X; Área de Infraestructuras de Investigación, Edificio CACTUS, Universidade of Santiago de Compostela, 15782, Santiago de Compostela, (Spain).
*e-mail: juanr.granja@usc.es

Supplementary Information

Table of Contents

**1. SUPPLEMENTARY FIGURES**	
**Supplementary Fig. 1. ^{1}H-NMR spectrum of D2.**	S4
**Supplementary Fig. 2. ESI-TOF exact mass analysis of D2.**	S5
**Supplementary Fig. 3. FTIR spectrum of D2.**	S5
**Supplementary Fig. 4. Comparison ^{1}H NMR spectra of D2 after addition of malononitrile and succinonitrile.**	S6
**Supplementary Fig. 5. ^{1}H NMR spectra of titration of D2 with malononitrile.**	S6
**Supplementary Fig. 6. NMR spectra after encapsulation of malononitrile inside D2.**	S7
**Supplementary Fig. 7. NMR observations upon succinonitrile addition over D2.**	S8
**Supplementary Fig. 8. DFT geometry optimization of MN$\subset$D2 and SN$\subset$D2.**	S9
**Supplementary Fig. 9. ^{1}H NMR titration of D2 with TBACl.**	S10
**Supplementary Fig. 10. Comparison of the most relevant in nOe crosspeaks of D2 before and after the complexation process with different anions.**	S11
**Supplementary Fig. 11. ESI-TOF negative mass spectra of the complex of D2 with chloride $mCl^-{\cdot}nH_2O\subset$2CP2.**	S12
**Supplementary Fig. S12. NMR study of the effect of drying solutions of $mA^-{\cdot}nH_2O\subset$ 2CP2 complexes with molecular sieves.**	S13
**Supplementary Fig. 13. Effects of water addition on the ^{1}H-NMR spectra of D2.**	S14
**Supplementary Fig. 14. ^{1}H NMR spectra of titration of D2 with TBAB.**	S15
**Supplementary Fig. 15. ^{1}H NMR spectra of titration of D2 with TBAI.**	S16
**Supplementary Fig. 16. ^{1}H NMR titration of D2 with TBAN.**	S17
**Supplementary Fig. 17. ^{1}H NMR spectra of titration of D2 with TBAAc.**	S18
**Supplementary Fig. 18. ^{1}H NMR spectra of titration of D2 with $TBAN_3$.**	S19
**Supplementary Fig. 19. ^{1}H NMR spectra of titrations of D2 with $TBABr_3$ and $TBAPF_6$.**	S20
**Supplementary Fig. 20. ^{1}H NMR spectra of titration of D1 with TBAF.**	S21
**Supplementary Fig. 21. ^{1}H NMR titration of D2 with NaAcO/15-crown-5.**	S22
**Supplementary Fig. 22. Comparison between ROESY NMR spectra of D2 complex with acetate using TBA^+ or Na^+ as counterion.**	S23
**Supplementary Fig. 23. Synthetic route for the preparation of CP1 and CP3.**	S24
**Supplementary Fig. 24. Formation of D2-3 heterodimer and subsequent addition of TBAF and TBACl.**	S25
**Supplementary Fig. 25. ESI-TOF exact mass analysis of D2-3.**	S26
**Supplementary Fig. 26. ^{1}H NMR spectra of titrations of D4 with TBAF and TBACl.**	S27
**Supplementary Fig. 27. Formation of D2-4 heterodimer and subsequent addition of TBAF.**	S28
**Supplementary Fig. 28. ESI-TOF exact mass analysis of D2-4.**	S29
**Supplementary Fig. 29. DFT geometry optimization of heterodimers D2-3 and D2-4.**	S30
**Supplementary Fig. 30. Fluoride hydration shell in $3F^-{\cdot}8H_2O\subset$2CP2.**	S31
**Supplementary Fig. 31. Fluoride occupancy in the two $3F^-{\cdot}8H_2O \subset$ 2CP2 that crystalized together.**	S32
**Supplementary Fig. 32. Apical and equatorial water molecules in $3F^-{\cdot}8H_2O\subset$2CP2.**	S33
**Supplementary Fig. 33. X-ray structure of D3.**	S34
**Supplementary Fig. 34. Lucigenin assay with D4.**	S34
**Supplementary Fig. 35. Lucigenin assays with D2 using $NaNO_3$ or Na_2SO_4.**	S35
**Supplementary Fig. 36. DLS measurements in lucigenin and HPTS assays.**	S36

**2. MATERIALS**	S37
**3. SUPPLEMENTARY DISCUSSION I: ^{1}H NMR TITRATION PROCEDURES**	S39
**Titrations with TBA^{+} salts**	
**Titrations with previous heterodimer formation**	
**Titrations with malononitrile and succinonitrile**	
**Titration of D2 with NaAcO/15-crown-5**	
**4. SUPPLEMENTARY DISCUSSION II: COMPLEX STRUCTURE ANALYSIS, NMR AND X-RAY DATA COMPARISON**	S40
**5. SUPPLEMENTARY DISCUSSION III: DFT CALCULATIONS**	S49
**5.1 Nitriles encapsulated inside D2 cavity.**	
**5.2 Homodimeric and heterodimeric structures**	
**6. SUPPLEMENTARY DISCUSSION IV: VESICLE PREPARATION AND TRANSPORT MEASUREMENTS**	S53
**6.1 Lucigenin assay**	
**6.1.1 Cation variation**	
**6.1.2 Anion variation**	
**6.1.3 Sulfate variation**	
**6.1.4 Data treatment**	
**6.2 HPTS assay**	
**6.2.1 FCCP variation**	
**6.2.2 Competition variation**	
**6.3.3 Data treatment**	
**7. SYNTHESIS AND CHARACTERIZATION**	S58
**8. SUPPLEMENTARY SPECTRA**	S67
**9. EXPERIMENTAL CRYSTAL STRUCTURE DETERMINATION**	S112
**10. SUPPLEMENTARY REFERENCES**	S120

1. SUPPLEMENTARY FIGURES

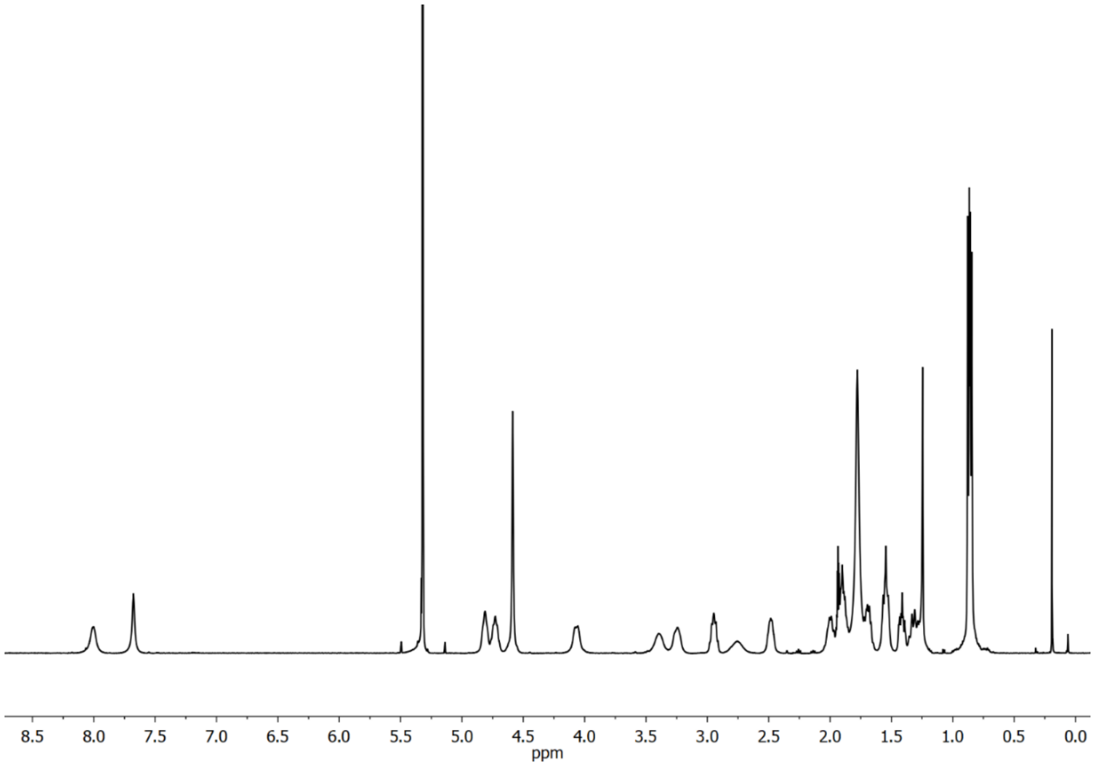

Supplementary Fig. 1. ¹H NMR spectrum of the receptor D2. The simplicity of the spectrum signals clearly indicates *C3*-simmetry ([**CP2**] = 5 mM in 10% CD_3CN/CD_2Cl_2).

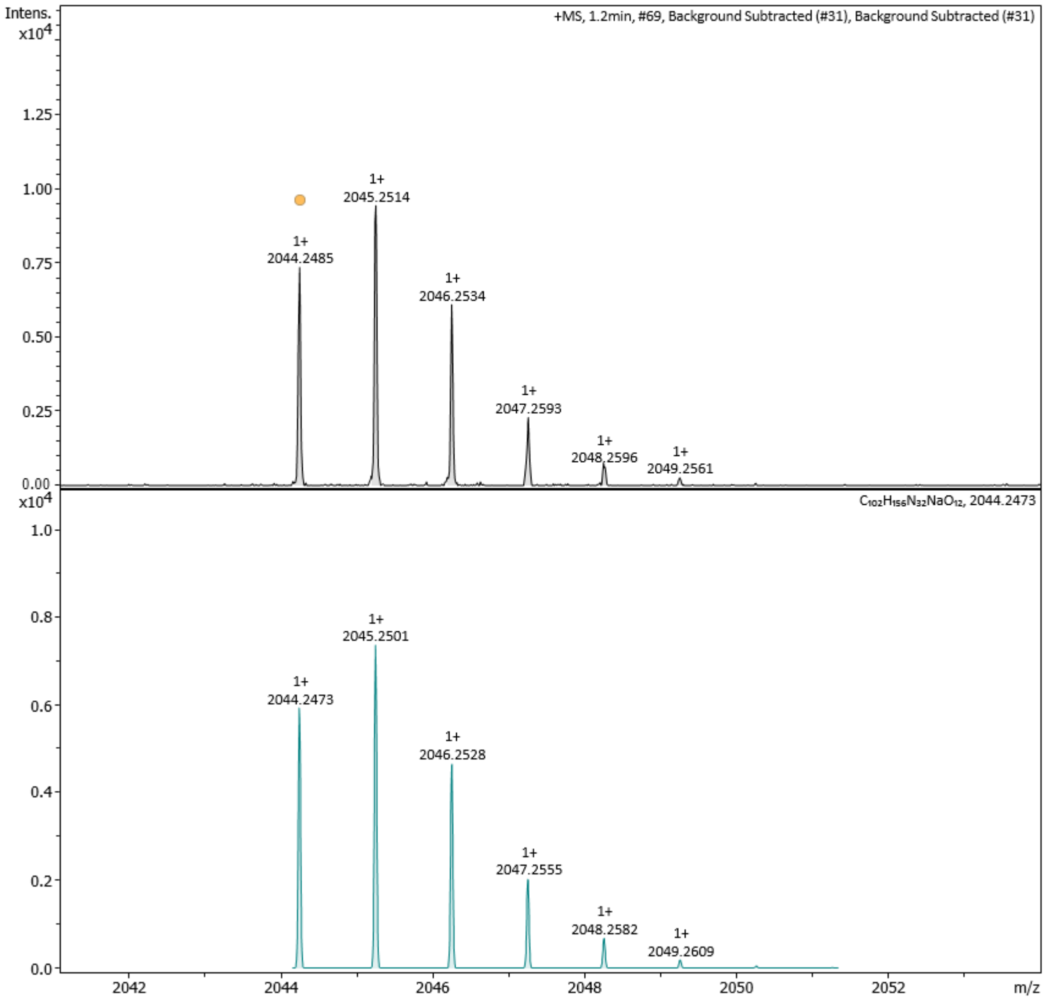

Meas. m/z	#	Ion Formula	Score	m/z	err [mDa]	err [ppm]	mSigma	rdb	e⁻ Conf	N-Rule
1033.6189	1	C51H78N16NaO6	100.00	1033.6182	-0.7	-0.6	21.6	21.0	even	ok
	2	C56H78N14NaO4	9.02	1033.6223	3.4	3.3	43.0	25.0	even	ok
2044.2485	1	C102H156N32NaO12	100.00	2044.2473	-1.2	-0.6	19.4	41.0	even	ok

Supplementary Fig. 2. **ESI-TOF exact mass analysis of D2**.

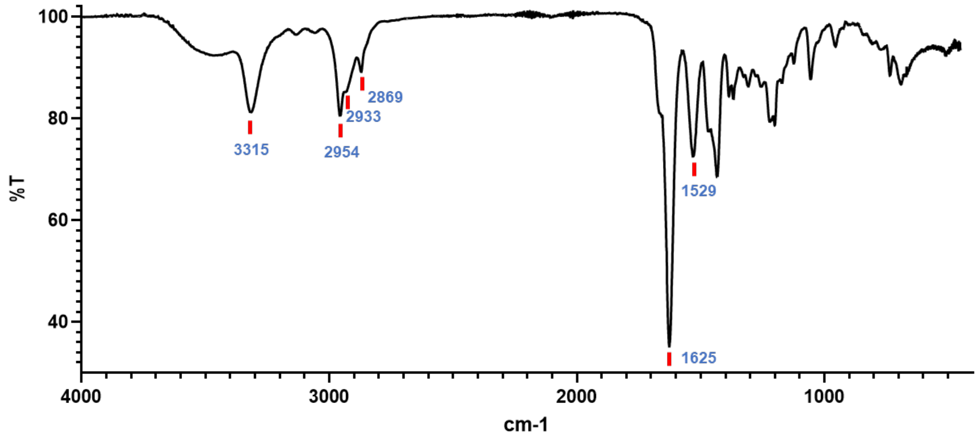

Supplementary Fig. 3. **FTIR spectrum of D2**.

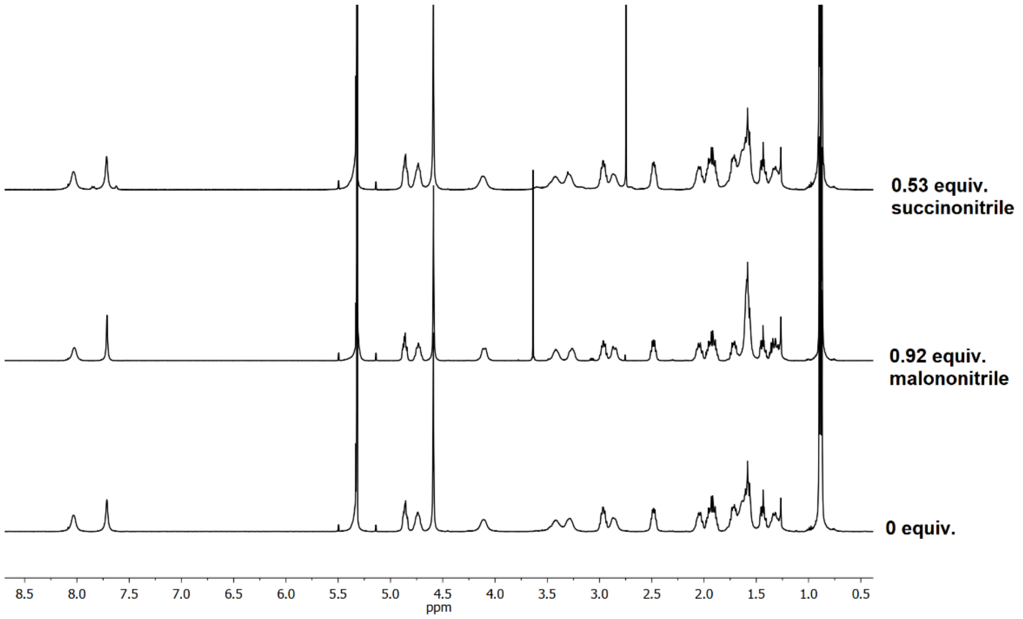

Supplementary Fig. 4. Comparison ^{1}H NMR spectra of D2 after addition of malononitrile and succinonitrile. ^{1}H NMR spectra of **D2** (CD_2Cl_2, TMSS was used as internal standard) upon the addition of 0.92 equivalents of malononitrile (singlet at 3.64 ppm) and 0.53 equivalents of succinonitrile (singlet at 2.75 ppm) over **D2**.

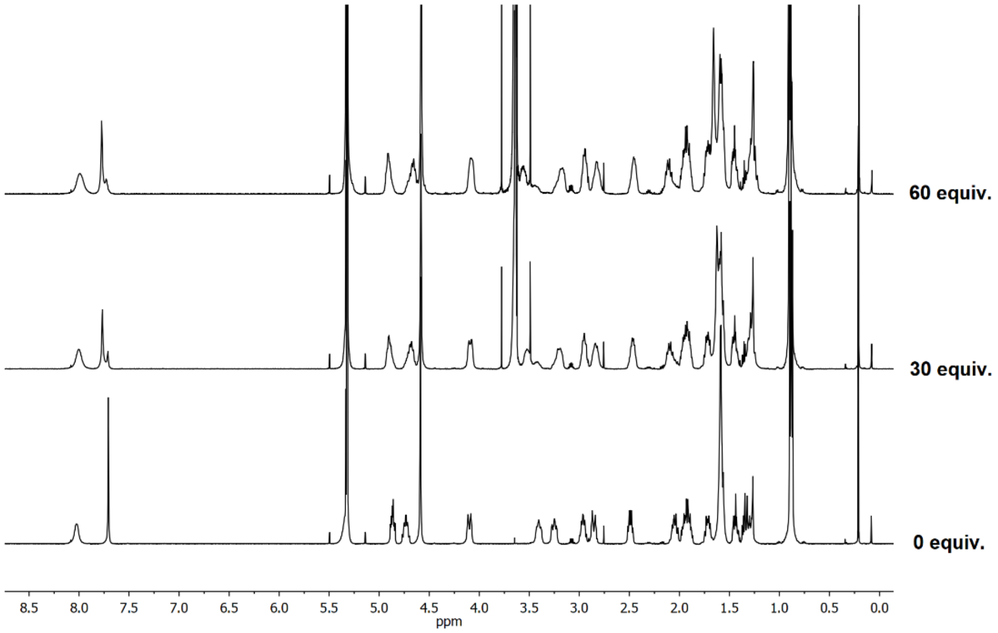

Supplementary Fig. 5. ^{1}H NMR spectra of titration of D2 with malononitrile. ^{1}H NMR spectra of **D2** ([**CP2**] = 4.7 mM in CD_2Cl_2 with TMSS as internal standard) after the addition of 30 and 60 equivalents of malononitrile.

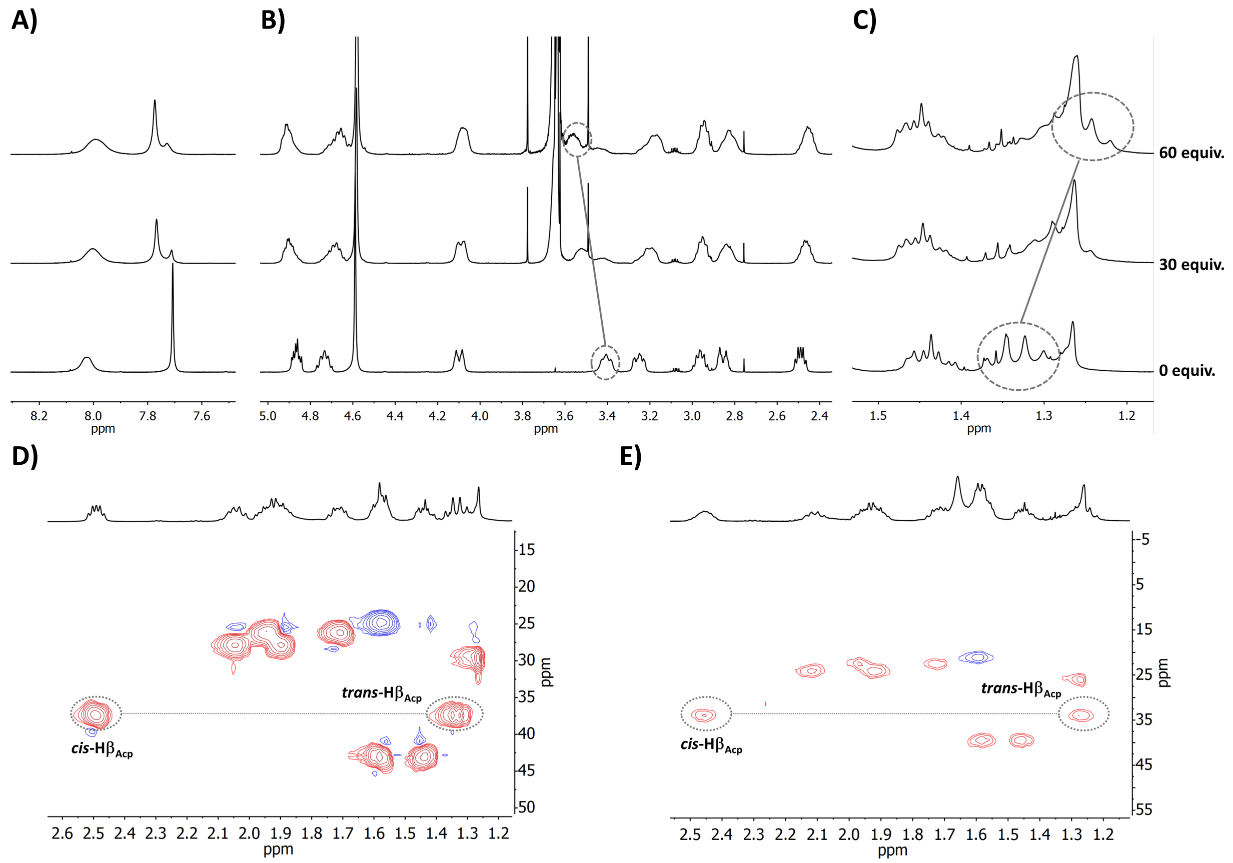

Supplementary Fig. 6. NMR spectra after encapsulation of malononitrile inside D2. Enlarged sections of the ^{1}H NMR spectra (CD_2Cl_2) corresponding to the additions of 30 and 60 equivalents of MN (Supplementary Fig. 5); A) The triazole proton of **D2** (7.71 ppm) suffers down-field shift to 7.77 ppm; B) broadening and shift ($\Delta\delta$=0.15 ppm) methylene protons signals (3.41 ppm) of tris(triazolylethyl)amine cap (see section 7 and 8 in **D2** characterization); C) up-field shift of the signal at 1.34 ppm corresponding to one of the protons of Hβ_{Acp} (*trans* to the carboxy and amino groups of Acp residue); D) and E) HSQC spectra of **D2** without and with malononitrile, respectively, after the addition of 60 equivalents. The signal at 2.5 ppm correspond to the axial oriented Hβ_{Acp} (*cis* to the carboxy and amino groups of Acp).

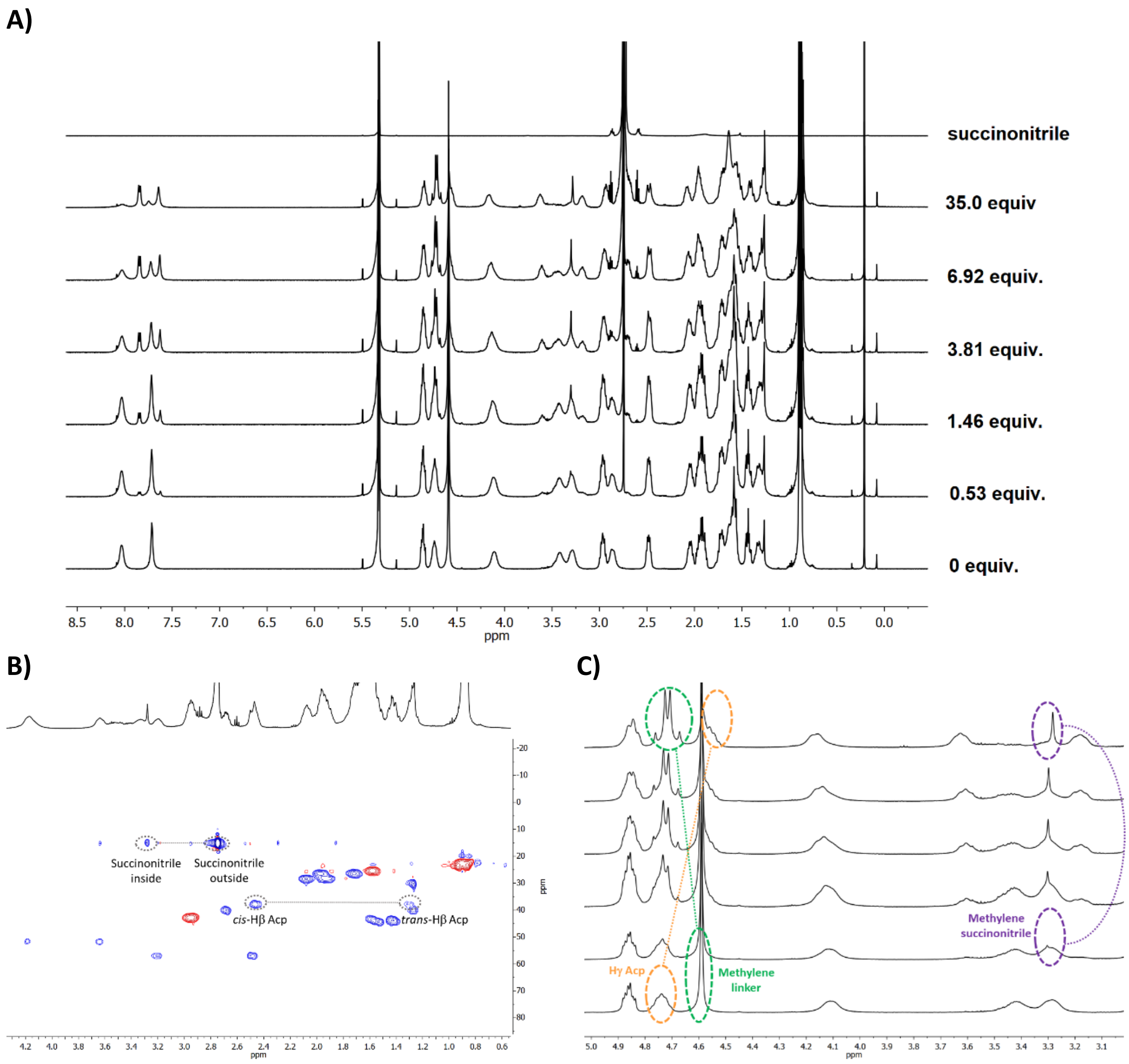

Supplementary Fig. 7. NMR observations upon succinonitrile addition over D2. A) Stack of ^{1}H NMR spectra corresponding to the titration of a solution of **D2** ([**CP2**] = 6.4 mM, CD_2Cl_2) with different equivalents of succinonitrile. The most relevant changes take place after the addition of ~0.5 equivalents in which a new set of signals are already beginning to appear. Especially evident are the changes of the amide and triazole protons that appear at 7.84 and 7.63 ppm, respectively. The new singlet at 3.30 ppm corresponds to the methylene groups of the entrapped SN (top spectrum); B) HSQC spectra in which the correlation between the encapsulated (3.30 ppm) and unentrapped (2.75 ppm) SN moiety with the same ^{13}C NMR signal is denoted. As previously mentioned for **MN** encapsulation the signal of *trans*-protons of $H\beta_{Acp}$ is also up-field shift (1.30 ppm); C) magnification of ^{1}H NMR spectra of **SN** additions to highlight the changes suffered by some signals of **D2** protons. In orange is highlighted the $H\gamma_{Acp}$ that experimented up-field shift. The methylene linker (in green), that now appears like an AB system indicates the decrease in freedom mobility of this group upon **SN** binding.

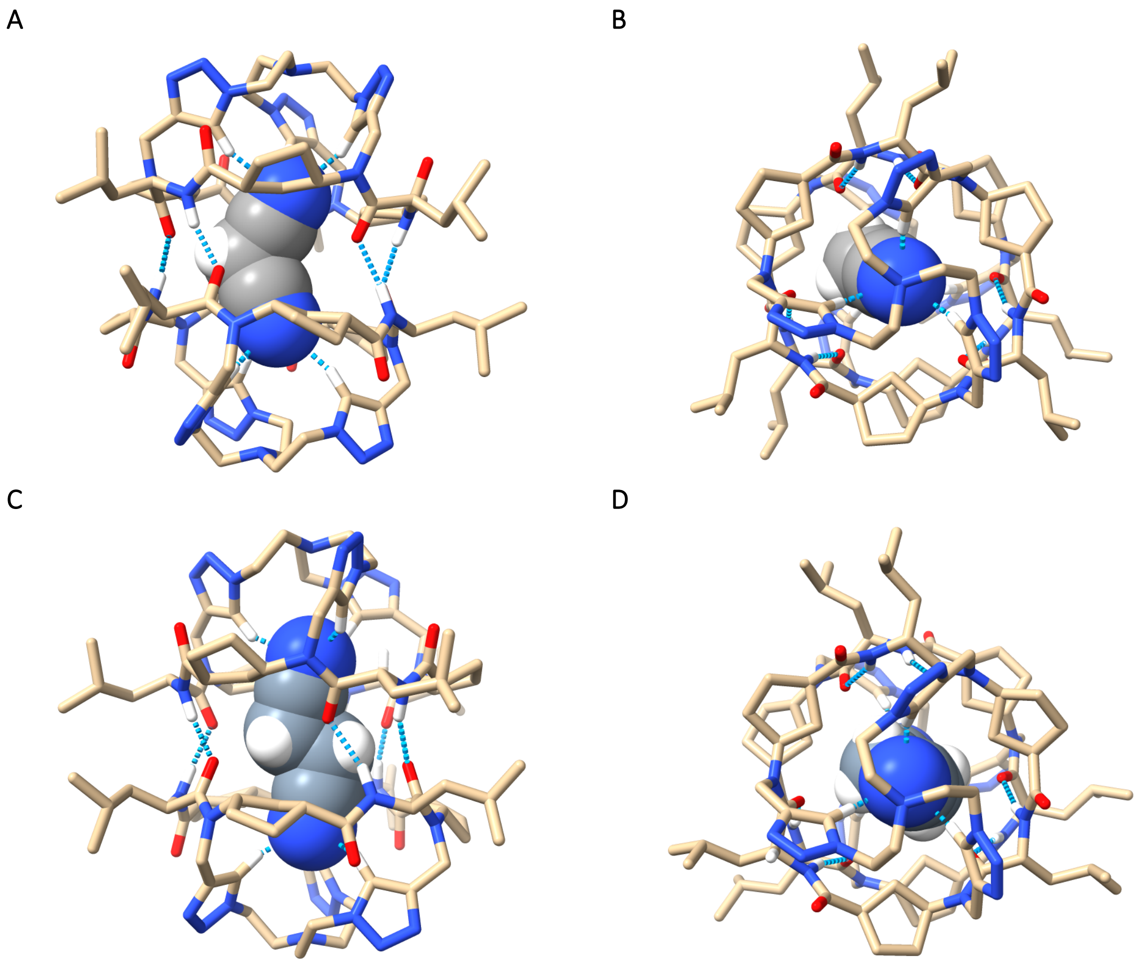

Supplementary Fig. 8. **DFT geometry optimization of MN⊂D2 and SN⊂D2**. The bisnitrile molecules are represented in CPK models. A) side and B) top view of **MN⊂D2**; C) side and D) top view of **SN⊂D2**. The level of calculation employed was B3LYP/6-31G(d,p), including GD3BJ as dispersion. The nitrogen atoms are depicted in blue, oxygens in red and hydrogens in white, as well as carbons in beige (except for the encapsulated bisnitriles molecules, where the carbons are depicted in grey).

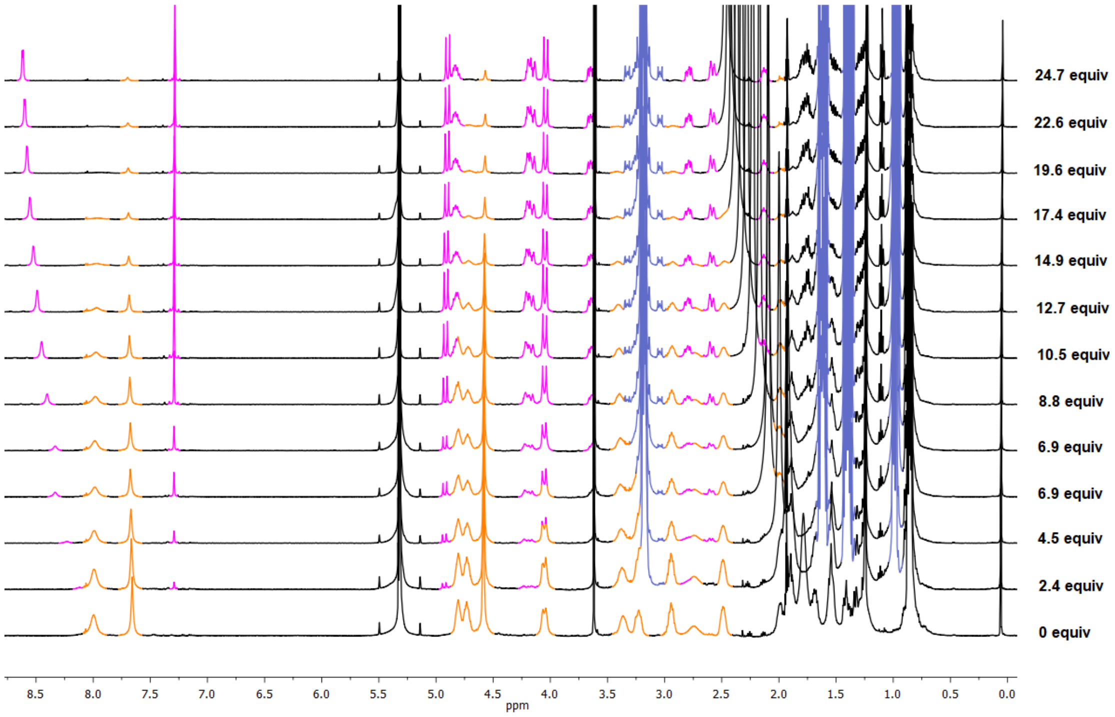

Supplementary Fig. 9. ^{1}H NMR spectra of titration of D2 with TBACl. Stack of ^{1}H NMR spectra of a solution of **D2** ([**CP2**] = 5.5 mM) in 10% CD_3CN/CD_2Cl_2, containing dioxane as internal standard, after the addition of different equivalents of TBACl. The signals corresponding to the empty capsule **D2** are highlighted in orange, while the new set of signals that emerge upon the additions, which belongs to the complex formed between **D2** and chloride, appear in magenta (light blue correspond to the signals of TBA^+ counterion). The singlet at 3.6 ppm correspond to dioxane that was used as internal standard.

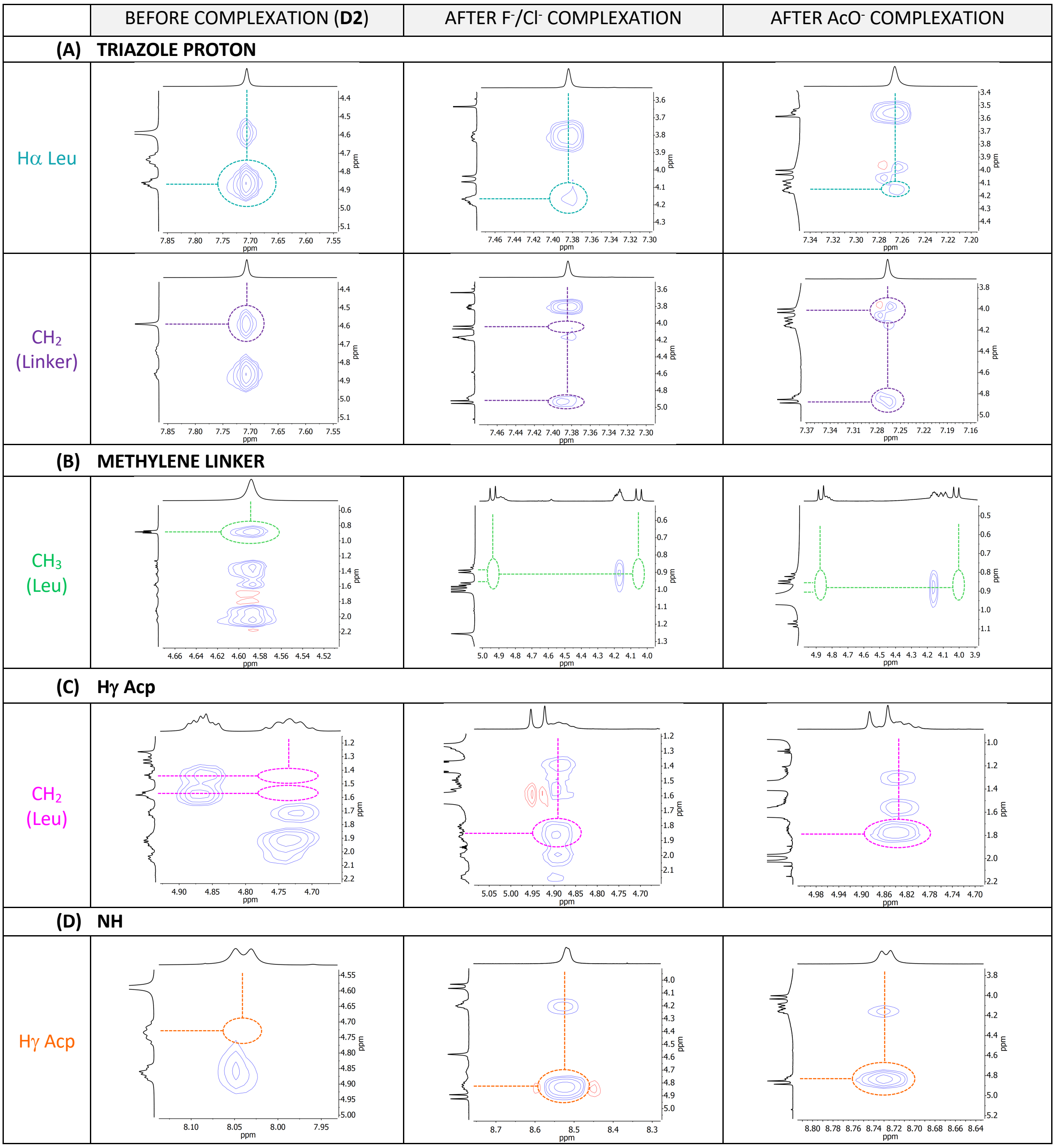

Supplementary Fig. 10. Comparison of the most relevant in nOe crosspeaks of D2 before and after the complexation process with different anions. Signals for four different protons are highlighted, (A) triazole proton, (B) methylene linker, (C) $H\gamma_{Acp}$ and (D) NH (for chloride addition), on selected ROESY spectra, showing de differences on nOe crosspeaks for both species. For a deeper analysis of the CP geometry through the complexation process, see Supplementary Discussion IV. ROESY spectrum of fluoride complex **$mF^{-}\cdot nH_2O \subset$ 2CP2** is compared with free **D2**. For the NH signal, **$mCl^{-}\cdot nH_2O \subset$ 2CP2** complex was used instead of the **$mF^{-}\cdot nH_2O \subset$ 2CP2** because their amide protons have not any cross-peak. The third row corresponds to the acetate complex, **$mAcO^{-}\cdot nH_2O \subset$ 2CP2**, confirming the similarity of both complexes. All the spectra were carried out in 10% CD_3CN/CD_2Cl_2.

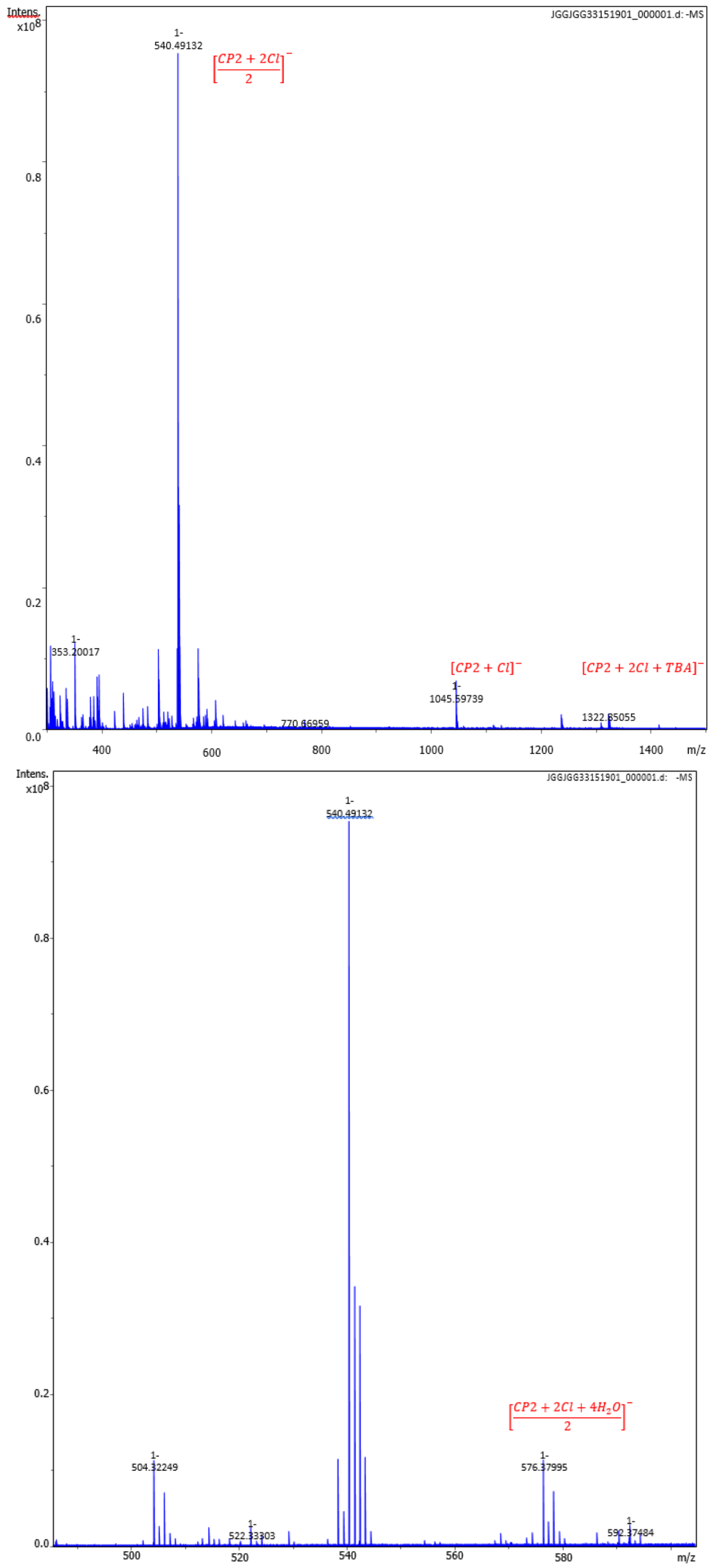

Supplementary Fig. S11. ESI-TOF negative mass spectra of the complex of D2 with chloride $mCl^- \cdot nH_2O \subset 2CP2$.

A)

B)

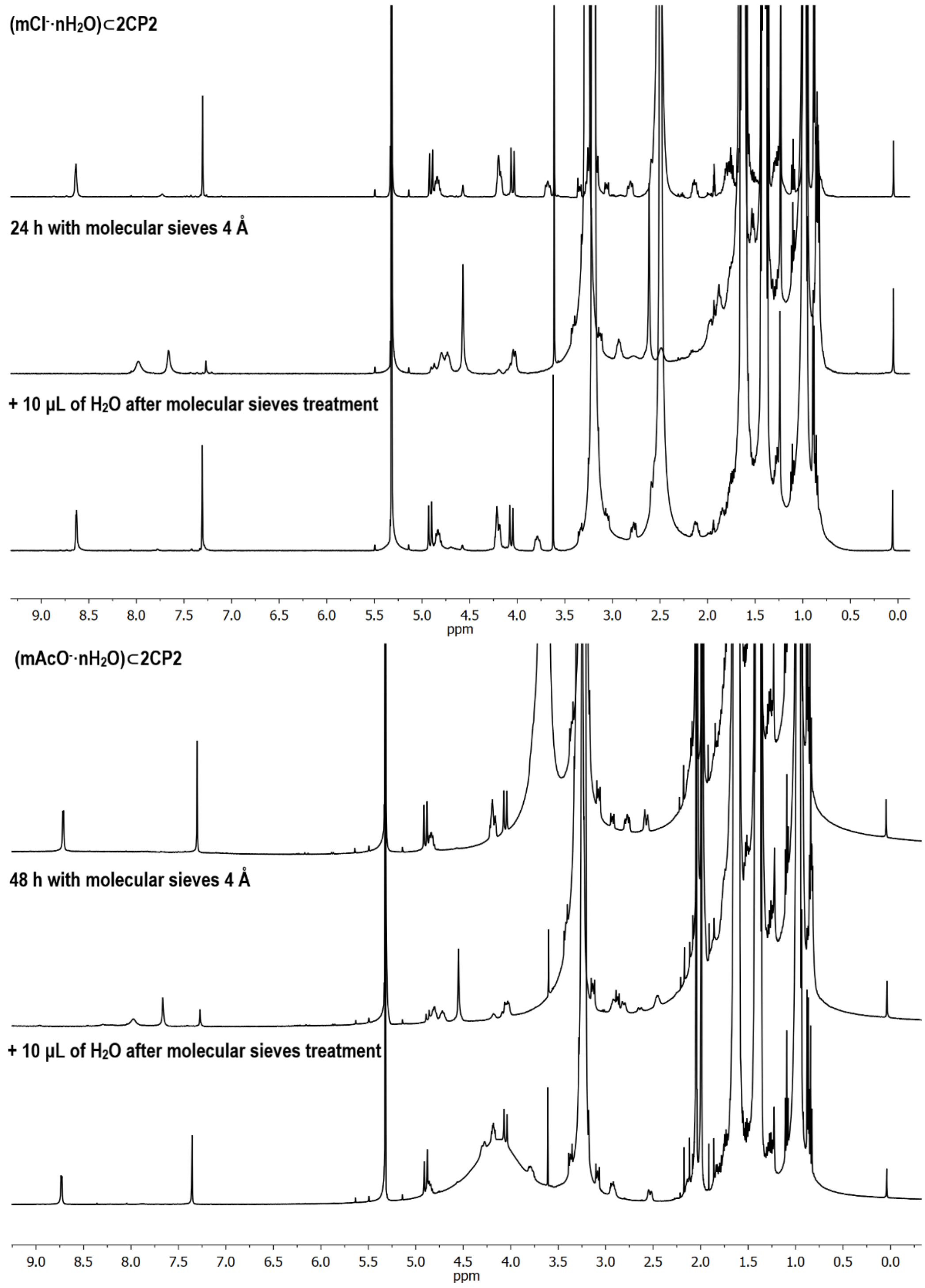

Supplementary Fig. 12. NMR study of the effect of drying solutions of mA$^-$·nH$_2$O⊂2CP2 complexes with molecular sieves. ^{1}H NMR spectra [10% CD_3CN/CD_2Cl_2], containing dioxane as internal standard] of (A) chloride complex **mCl$^-$·nH$_2$O⊂2CP2** and (B) acetate complex **mAcO$^-$·nH$_2$O⊂2CP2** after drying with molecular sieves and subsequent rehydration by the addition of water (10 µL). In the spectra of both samples, it can be observed that after the addition of water the characteristic signals of the corresponding complexes are restored. For the drying of the acetate complex longer time was required (48 h) compared with chloride (24 h).

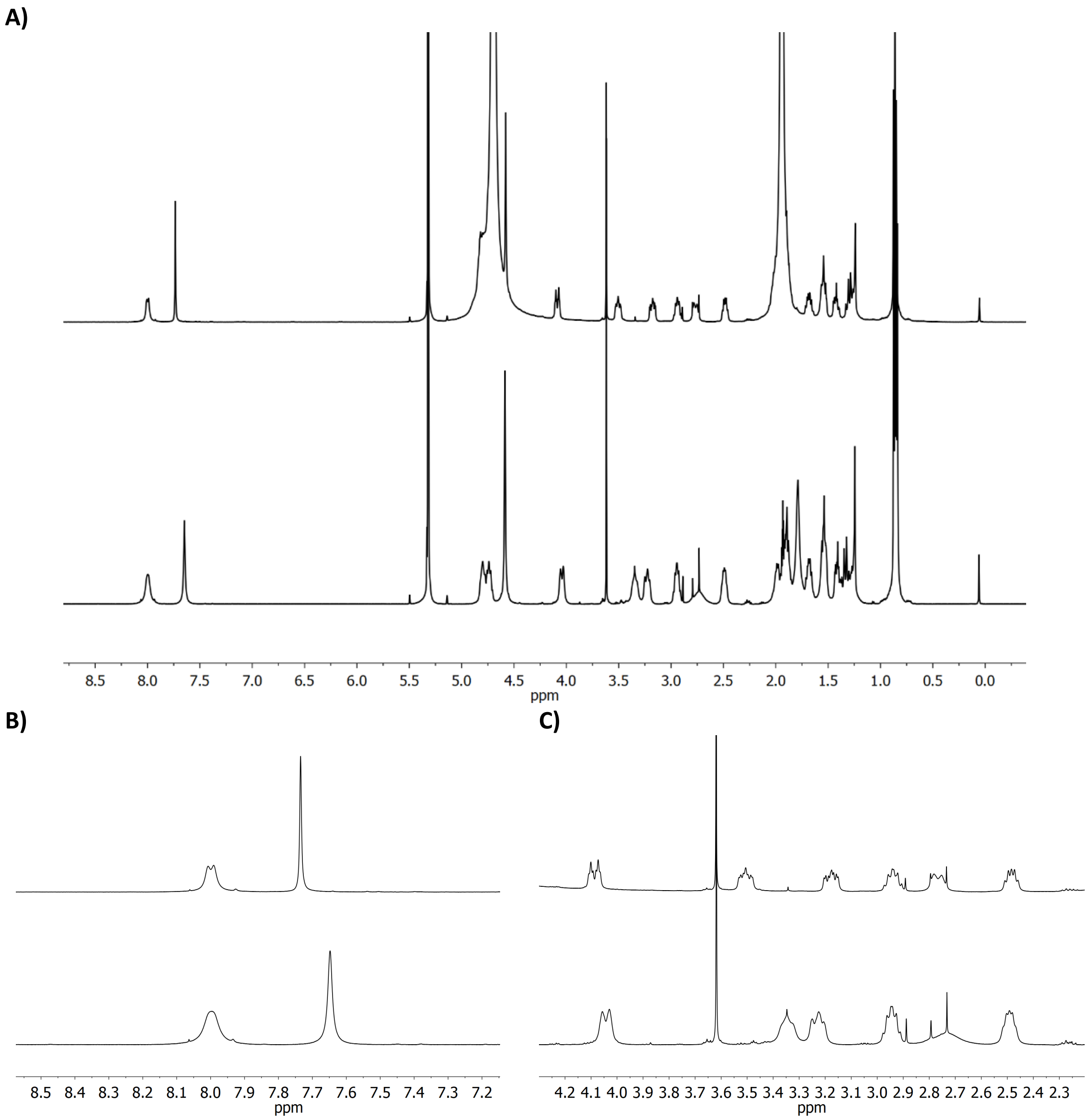

Supplementary Fig. 13. Effects of water addition on the ^{1}H NMR spectra of D2. (A) ^{1}H NMR spectra of a solution of **D2** ([**CP2**] = 5.6 mM) in 10% CD_3CN/CD_2Cl_2, containing dioxane as internal standard, before (bottom) and after (top) the addition of H_2O (7 µL) which causes the down-field shift of the triazole proton, as denoted in the (B) 8.56–6.94 ppm region of the ^{1}H NMR spectra. (C) The 4.2–2.2 ppm region of the ^{1}H NMR spectra denoting that after addition of water most of the signals are well defined.

A)

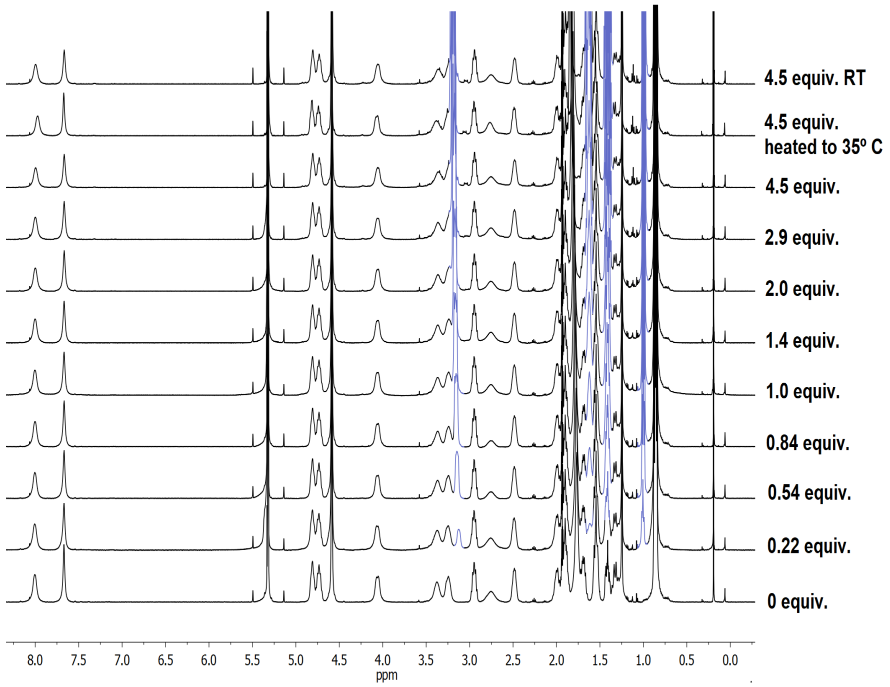

B)

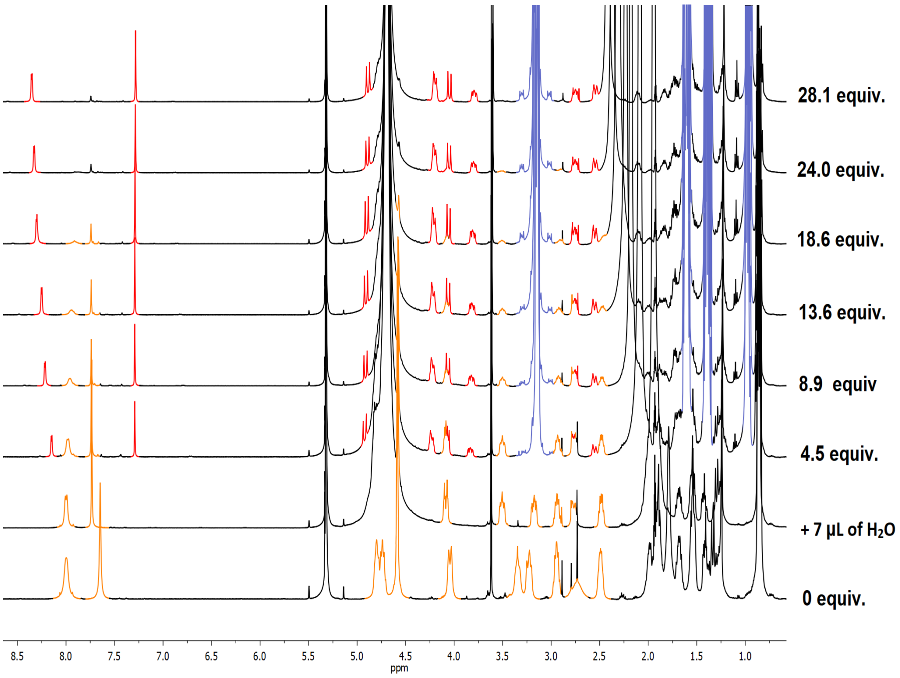

Supplementary Fig. 14. ^{1}H NMR spectra of titration of D2 with TBAB. A) Stack of ^{1}H NMR spectra of a solution of **D2** ([**CP2**] = 4.8 mM) in 10% CD_3CN/CD_2Cl_2, containing TMSS as internal standard, after different additions of the stock solution of tetrabutylammonium bromide (**TBAB**). No changes were observed after the addition of 4.5 equivalents of this salt; B) stack of ^{1}H NMR spectra of a solution of **D2** ([**CP2**] = 5.6 mM, in 10% CD_3CN/CD_2Cl_2, employing dioxane as internal standard, after the addition of H_2O (7 µL) and followed by the successive additions of TBAB stock solution. The most relevant signals from **D2** are depicted in orange, while the new set of signals that emerge after the additions of TBAB, which belongs to the complex formed between **CP2** and hydrated bromide [**$mBr^{-}\cdot nH_2O \subset 2CP2$**], are depicted in red (in light blue, the signals corresponding to TBA^+ counterion).

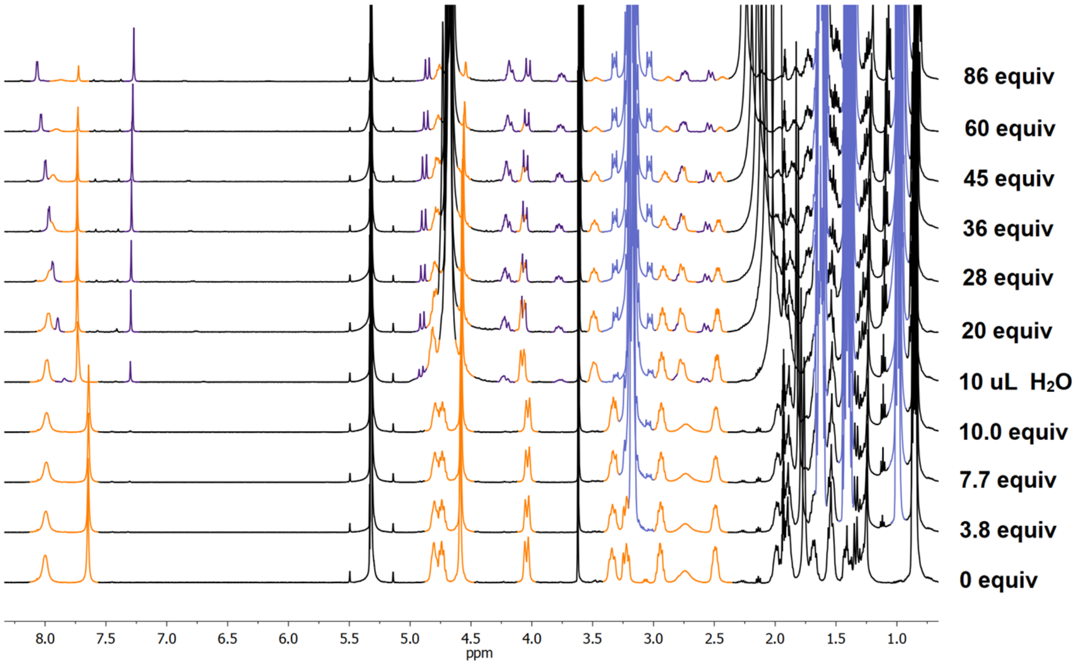

Supplementary Fig. 15. **^{1}H NMR spectra of titration of D2 with TBAI**. Stack of ^{1}H NMR spectra of a solution of **D2** ([**CP2**] = 7 mM) in 10% CD_3CN/CD_2Cl_2, containing dioxane as internal standard, after different additions of the stock solutions of tetrabutylammonium Iodide (**TBAI**). The most relevant signals of free **D2** are labelled in orange, while the new set of signals that emerge after the addition of TBAI, which belongs to the complex formed between **CP2** and iodide, **mI^-·nH_2O⊂2CP2**, appear in dark purple (in light blue, the signals from TBA^+ counterion). Upon the addition of 10 equivalents of TBAI, no changes were observed. Extra amount of water was needed to promote the formation of the complex with iodide.

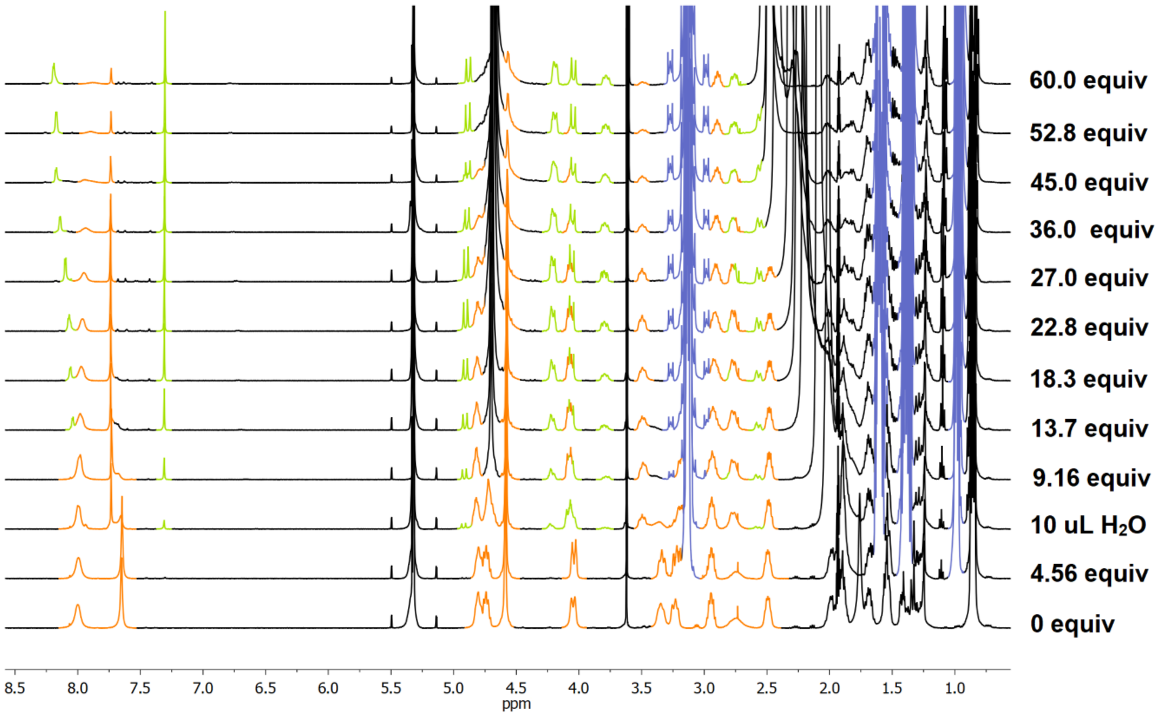

Supplementary Fig. 16. **^{1}H NMR spectra of titration of D2 with TBAN**. Stack of ^{1}H NMR spectra of a solution of **D2** ([**CP2**] = 7 mM) in 10% CD_3CN/CD_2Cl_2, containing dioxane as internal standard, after different additions of the stock solutions of tetrabutylammonium nitrate (**TBAN**). The most relevant signals of free **D2** are labelled in orange, while the new set of signals that emerge after the addition of TBAN, which belongs to the complex **$mNO_3^-{\cdot}nH_2O\subset 2CP2$**, appear in lime green (in light blue, the signals from TBA^+ counterion). Upon the addition of 4.56 equivalents of TBAN, no changes were observed. Extra amount of water (10 µL) was needed to promote the formation of the complex with nitrate.

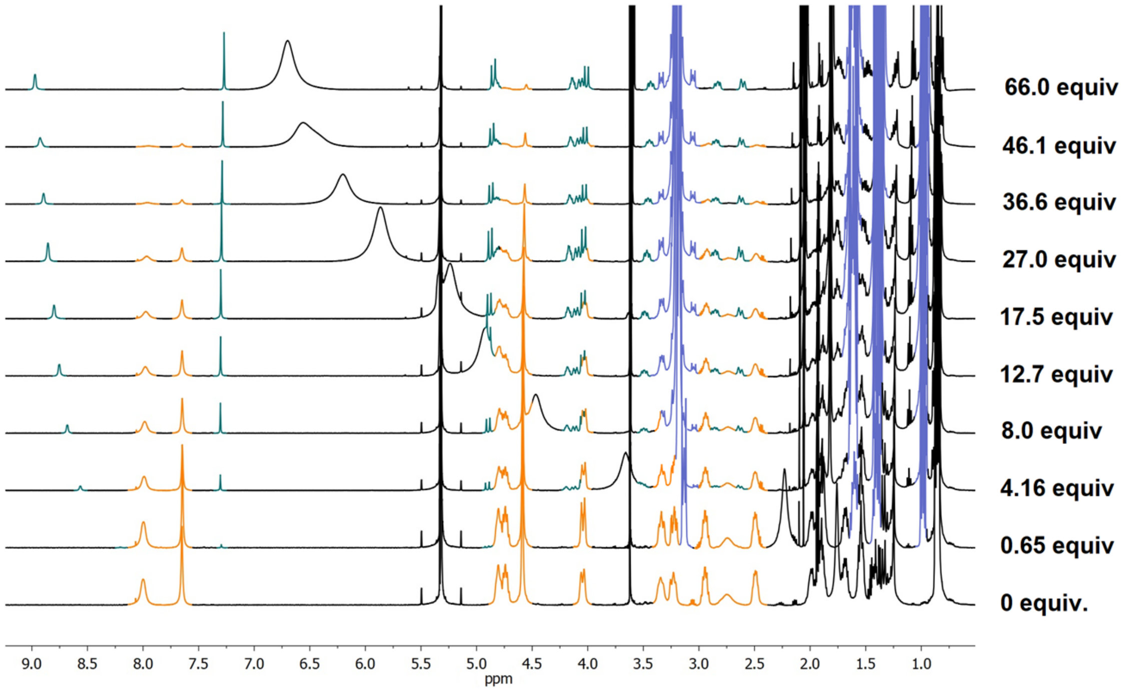

Supplementary Fig. 17. **^{1}H NMR spectra of titration of D2 with TBAAc**. Stack of ^{1}H NMR spectra of a solution of **D2** ([**CP2**] = 5.4 mM) in 10% CD_3CN/CD_2Cl_2, containing dioxane as internal standard, after different additions of the stock solutions of tetrabutylammonium acetate (**TBAAc**). The most relevant signals of free **D2** are labelled in orange, while the new set of signals that emerge after the additions of TBAAc, which belong to the complex **mAcO^-·nH_2O⊂2CP2**, appear in aquamarine blue (in light blue, the signals from TBA^+ counterion). Extra amount of water (10 µL) was needed to promote the formation of the complex with nitrate.

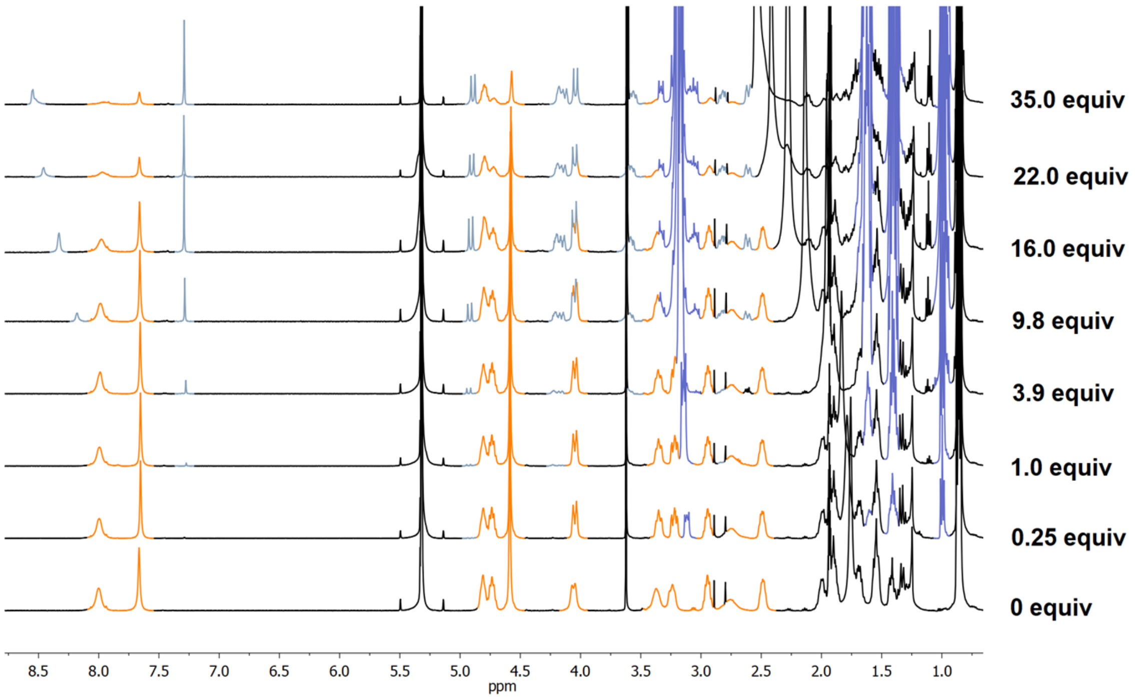

Supplementary Fig. S18. **^{1}H NMR spectra of titration of D2 with $TBAN_3$**. Stack of ^{1}H NMR spectra of a solution of **D2** ([**CP2**] = 6.9 mM) in 10% CD_3CN/CD_2Cl_2, containing dioxane as internal standard, after different additions of stock solution of tetrabutylammonium azide ($TBAN_3$). The most relevant signals of free **D2** are labelled in orange, while the new set of signals that emerge after the additions of $TBAN_3$, which belongs to the complex **$mN_3^-\cdot nH_2O\subset 2CP2$**, appear in grey (in light blue, the signals from TBA^+ counterion).

A) TBABr$_3$

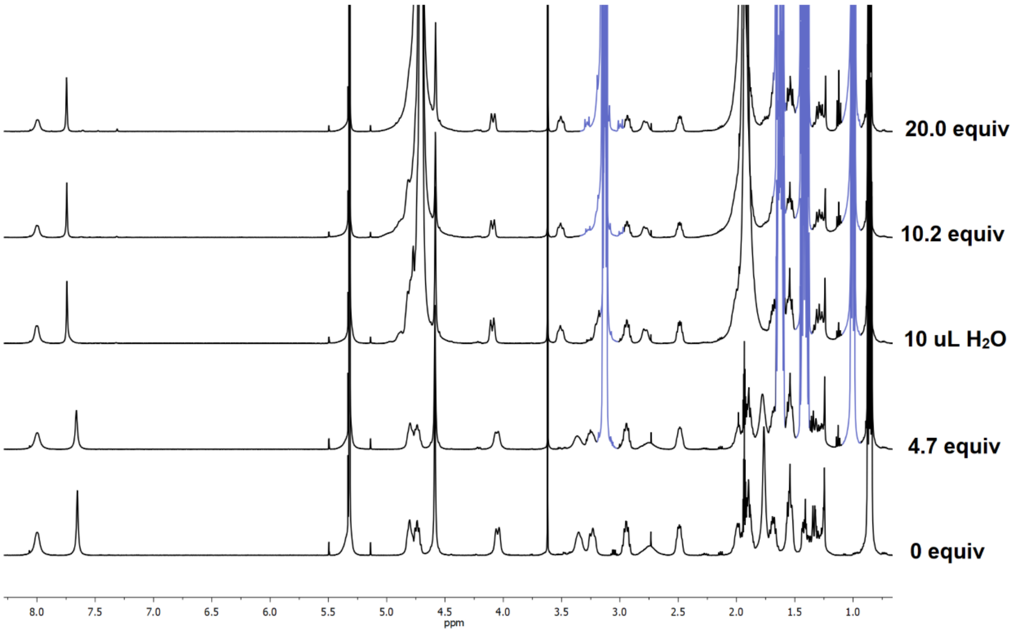

B) TBAPF$_6$

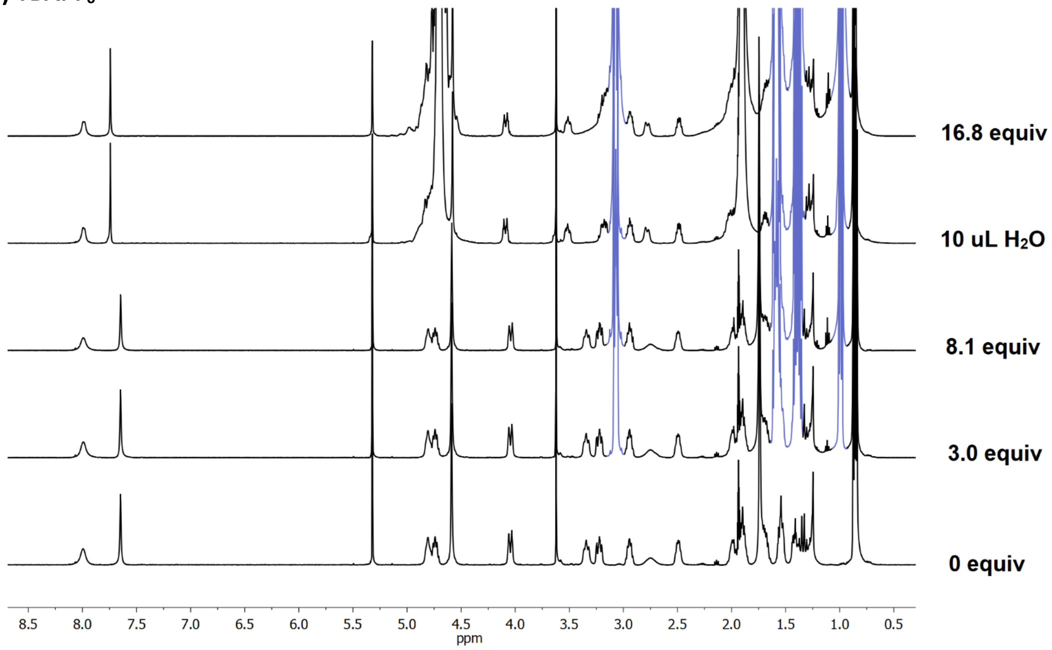

Supplementary Fig. 19. ^{1}H NMR spectra of titrations of D2 with TBABr$_3$ and TBAPF$_6$. (A) Stack of ^{1}H NMR spectra of a solution of **D2** ([**CP2**] = 7.3 mM) in 10% CD_3CN/CD_2Cl_2, containing dioxane as internal standard, after different additions of the stock solutions of tetrabutylammonium tribromide (**TBABr$_3$**). (B) Stack of ^{1}H NMR spectra of a solution of **D2** (5.5 mM) in 10% CD_3CN/CD_2Cl_2, containing dioxane as internal standard, after the addition of different equivalents of TBAPF$_6$. No changes were found after the addition of the mentioned salts.

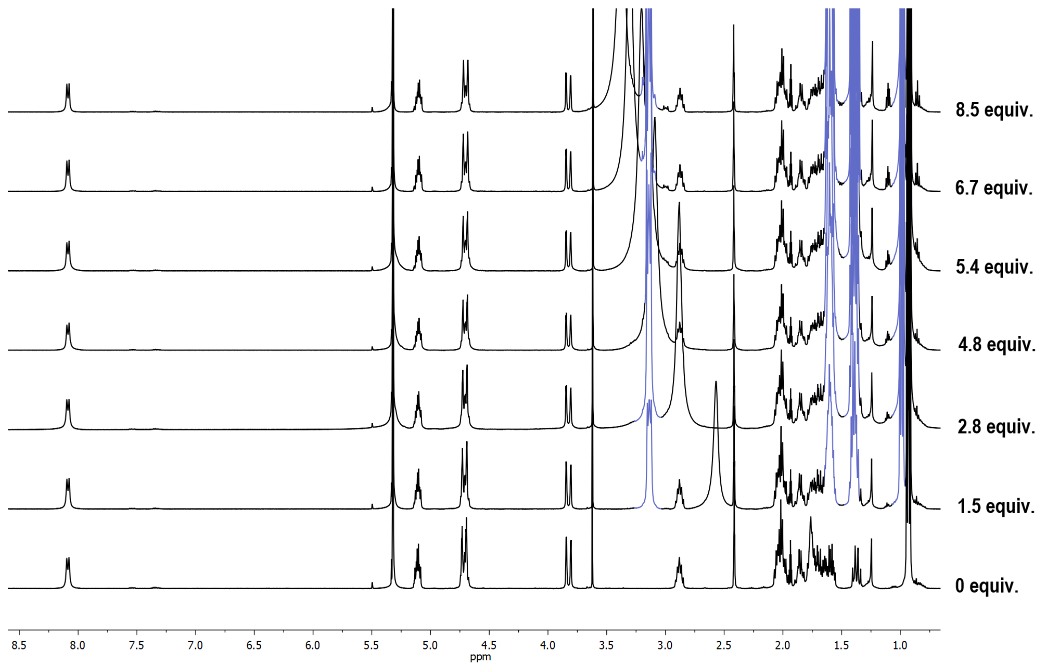

Supplementary Fig. 20. ^{1}H NMR spectra of titration of D1 with TBAF. Stack of ^{1}H NMR spectra of a solution of **D1** ([**CP1**] = 5.6 mM) in 10% CD_3CN/CD_2Cl_2, containing dioxane as internal standard, after different additions of the stock solutions of tetrabutylammonium fluoride (**TBAF**). No changes were observed with the additions of TBAF, confirming that this cyclic peptide dimer cannot recognize anions. In light blue, the signals from TBA^+ counterion.

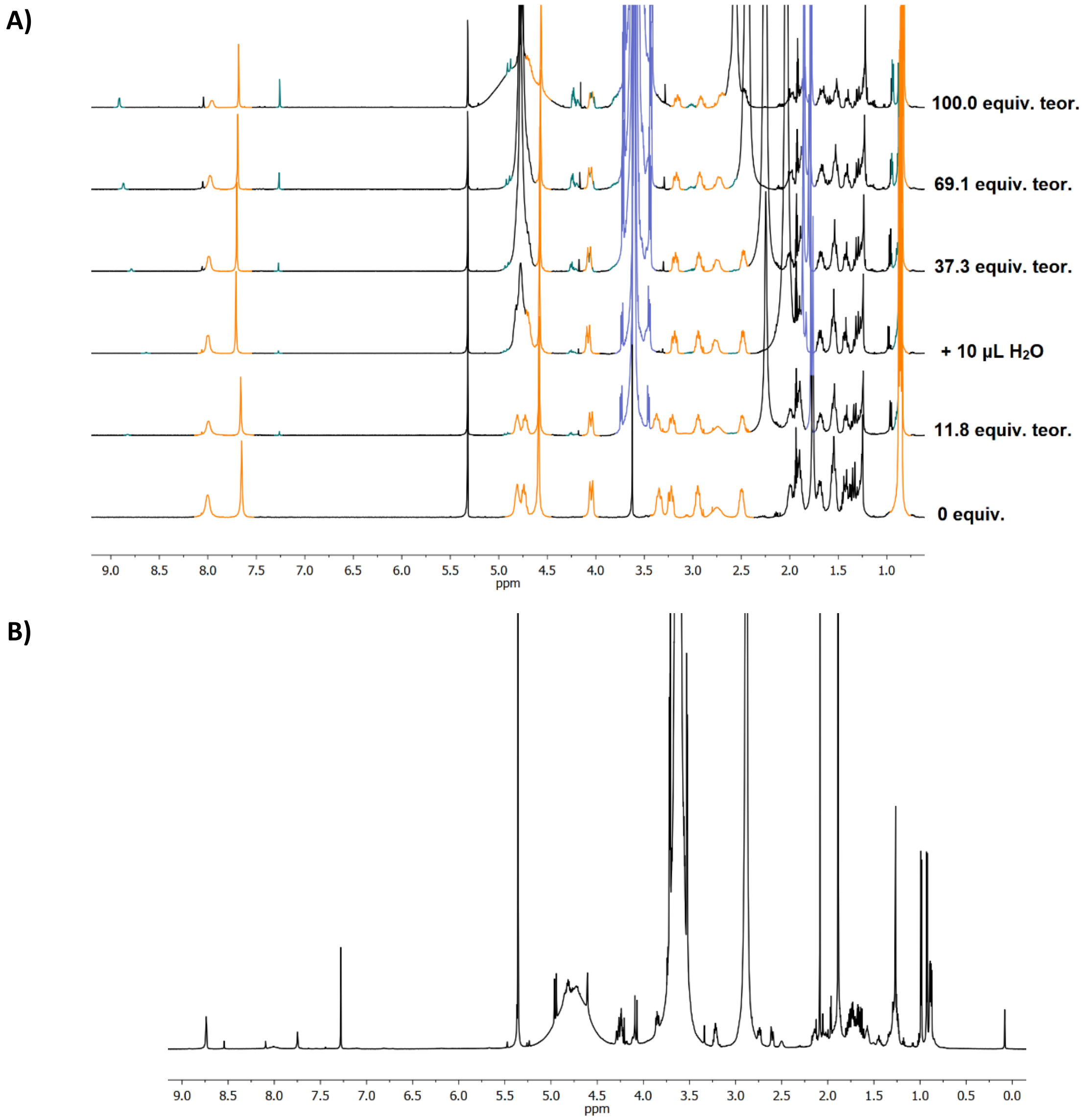

Supplementary Fig. 21. ^{1}H NMR spectra of titration of D2 with NaAcO/15-crown-5. (A) Stack of ^{1}H NMR spectra of a solution of **D2** ([**CP2**] = 6.9 mM) in 10% CD_3CN/CD_2Cl_2, containing dioxane as internal standard, after different additions of a solution of NaAcO/15-crown-5 in CD_3CN (2.28 M). The most relevant signals of free **D2** are labelled in orange, while the new set of signals that emerge after the addition of NaAcO/15-crown-5, which belongs to the acetate complex, appear in aquamarine blue (in light blue, the signals from TBA^+ counterion). Precipitation was observed, suggesting that the amount of available acetate added was smaller than the estimated considering the calculated concentration of the stock solution in CD_3CN. (B) ^{1}H NMR of the resulting solution of previous experiment after two weeks, in which higher concentration of the complex is observed.

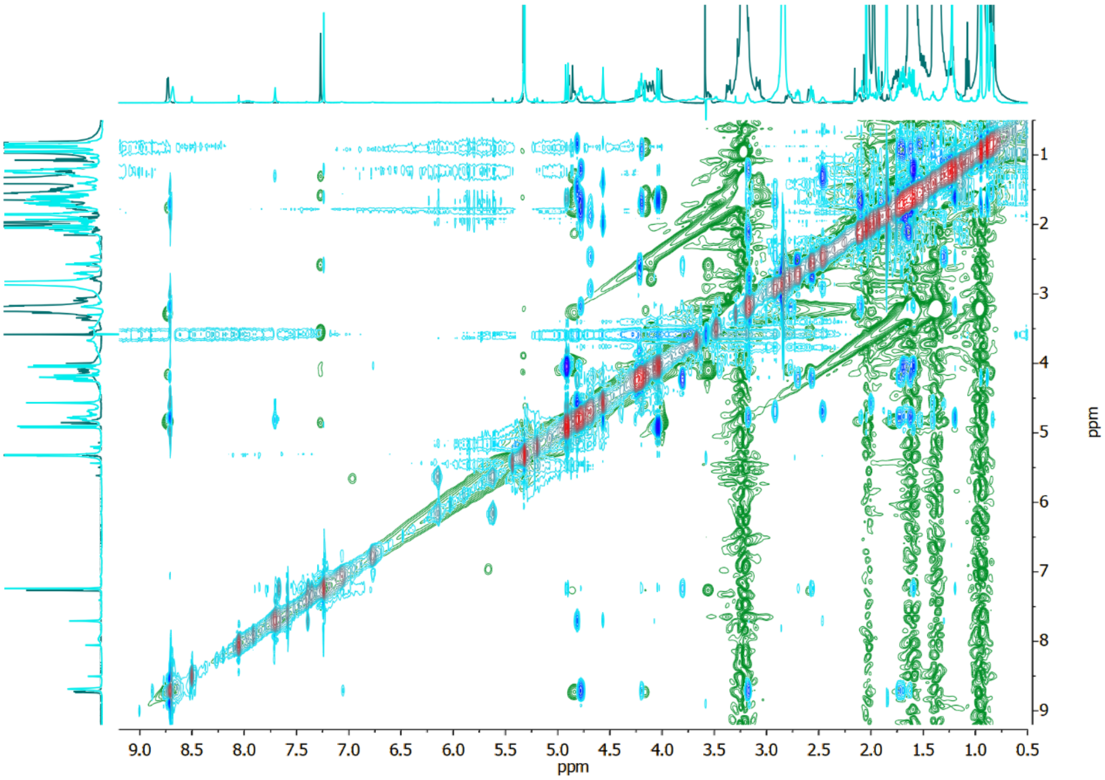

Supplementary Fig. 22. Comparison between ROESY NMR spectra of D2 complex with acetate using TBA$^+$ or Na$^+$ as counterion. The spectra derived by the addition of TBA$^+$ as counterion is depicted in green, and the spectra from the complex with Na$^+$ is depicted in light blue. The ^{1}H NMR spectra signals fit between both experiments, and also the nOe crosspeaks. The spectrum of sample with the Na$^+$ salt still containing some free **D2**, the nOe crosspeaks of which are still observed. Both experiments were run employing in 10% CD_3CN/CD_2Cl_2 as solvent.

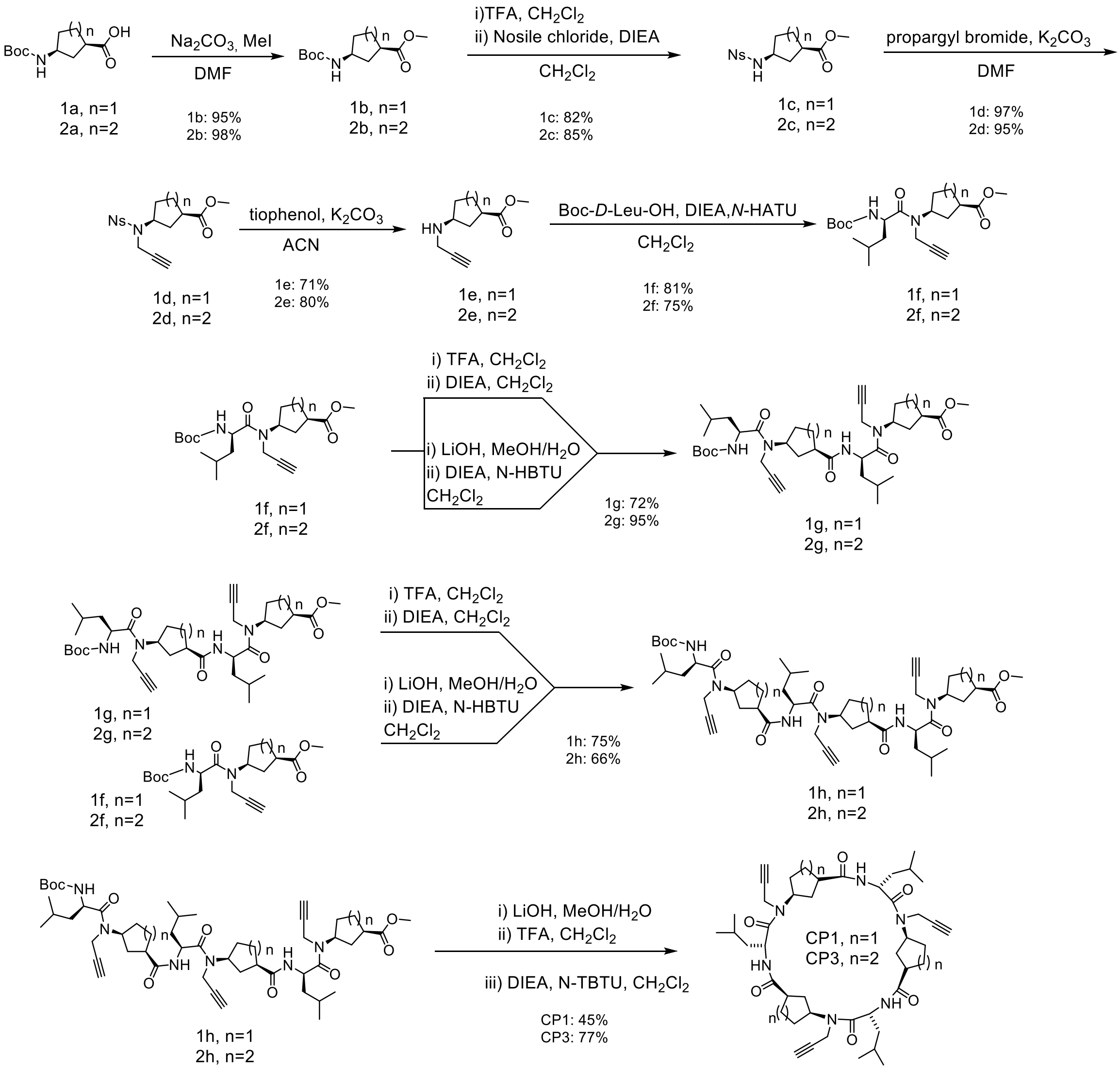

Supplementary Fig. 23. **Synthetic route used in the preparation of CP1 and CP3**. **CP1** is the Acp-based cyclic peptide and **CP3** is the Ach-based cyclic peptide; the same synthetic strategy was used for both compounds. See section 7 (pages 58-60, 62-65) and section 8 (pages 67-69 and 90-99) for the full synthetic procedures and characterization. Notice that both, **CP1** and **CP3** are able to self-assembly to form corresponding dimers **D1** and **D3**, respectively.

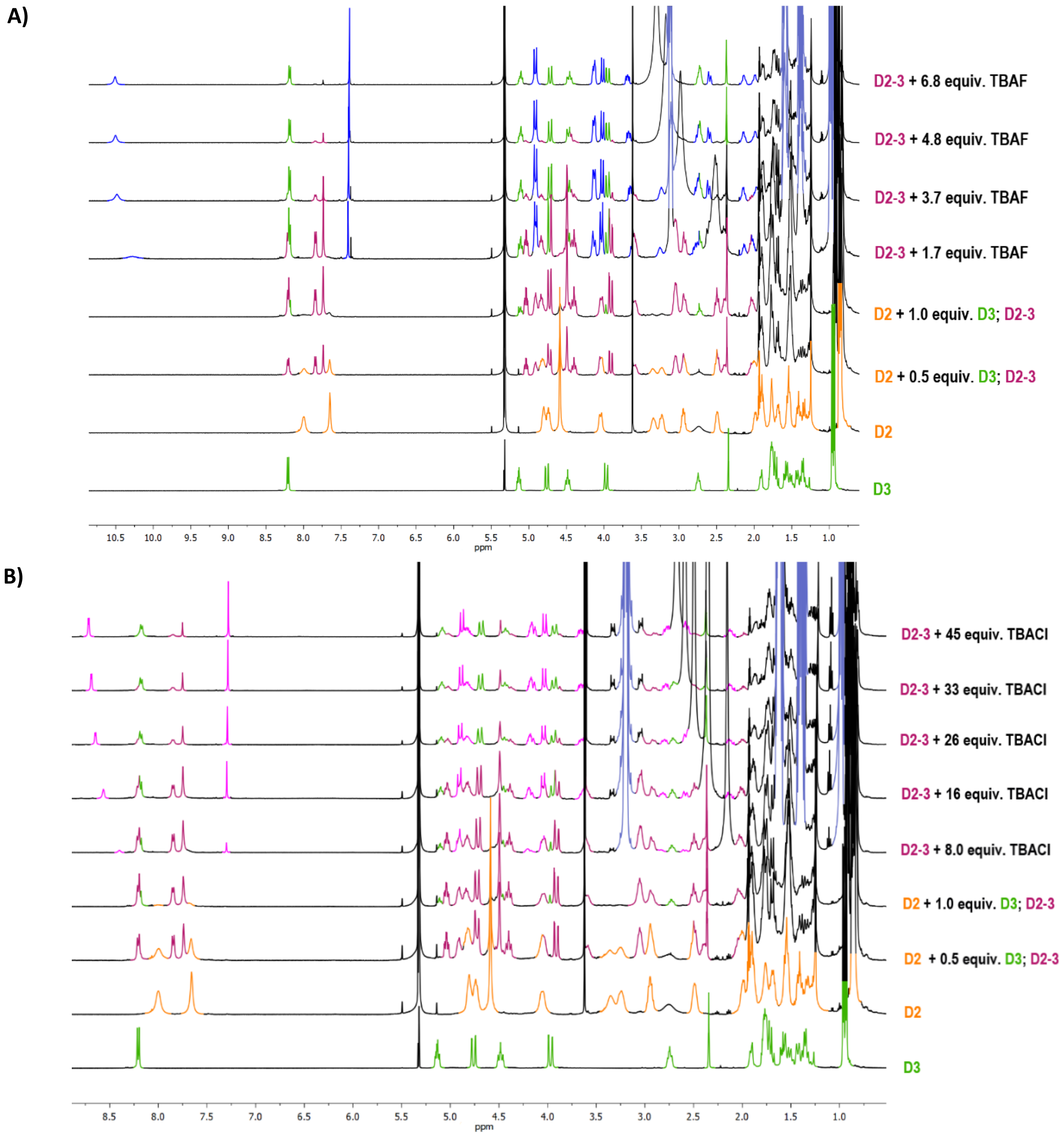

Supplementary Fig. 24. Formation of D2-3 heterodimer and subsequent addition of TBAF and TBACl. (A) Stack of ^{1}H NMR spectra of a solution of **D2** ([**CP2**] = 6.9 mM) in 10% CD_3CN/CD_2Cl_2, containing dioxane as internal standard, after different additions. Initially, **D3** was added over the solution of **D2**, and after that, different equivalents of TBAF were added. The most relevant signals of free **D2** are labelled in orange, in green the signals corresponding to **D3** while those corresponding to the heterodimer (**D2-3**) are in wine red. Finally, after the addition of TBAF, the signals assigned to the **$mF^{-}\cdot nH_2O\subset 2CP2$** complex are shown in blue. Under this conditions **D3** (in green) is recovered. (B) Stack of ^{1}H NMR spectra of a solution of **D2** ([**CP2**] = 6.4 mM) in 10% CD_3CN/CD_2Cl_2, containing dioxane as internal standard, after different additions. Initially **D3** was added over the solution of **D2**, and after that, different equivalents of TBACl were also added. The most relevant signals of free **D2** are labelled in orange, in green the signals corresponding to **D3** while those corresponding to the heterodimer (**D2-3**) are in wine red. Finally, after the addition of TBACl the signals assigned to the **$mCl^{-}\cdot nH_2O\subset 2CP2$** complex are shown in magenta. Under this conditions **D3** (in green) is recovered. For the full characterization of **D2-3** see sections 7 and 8.

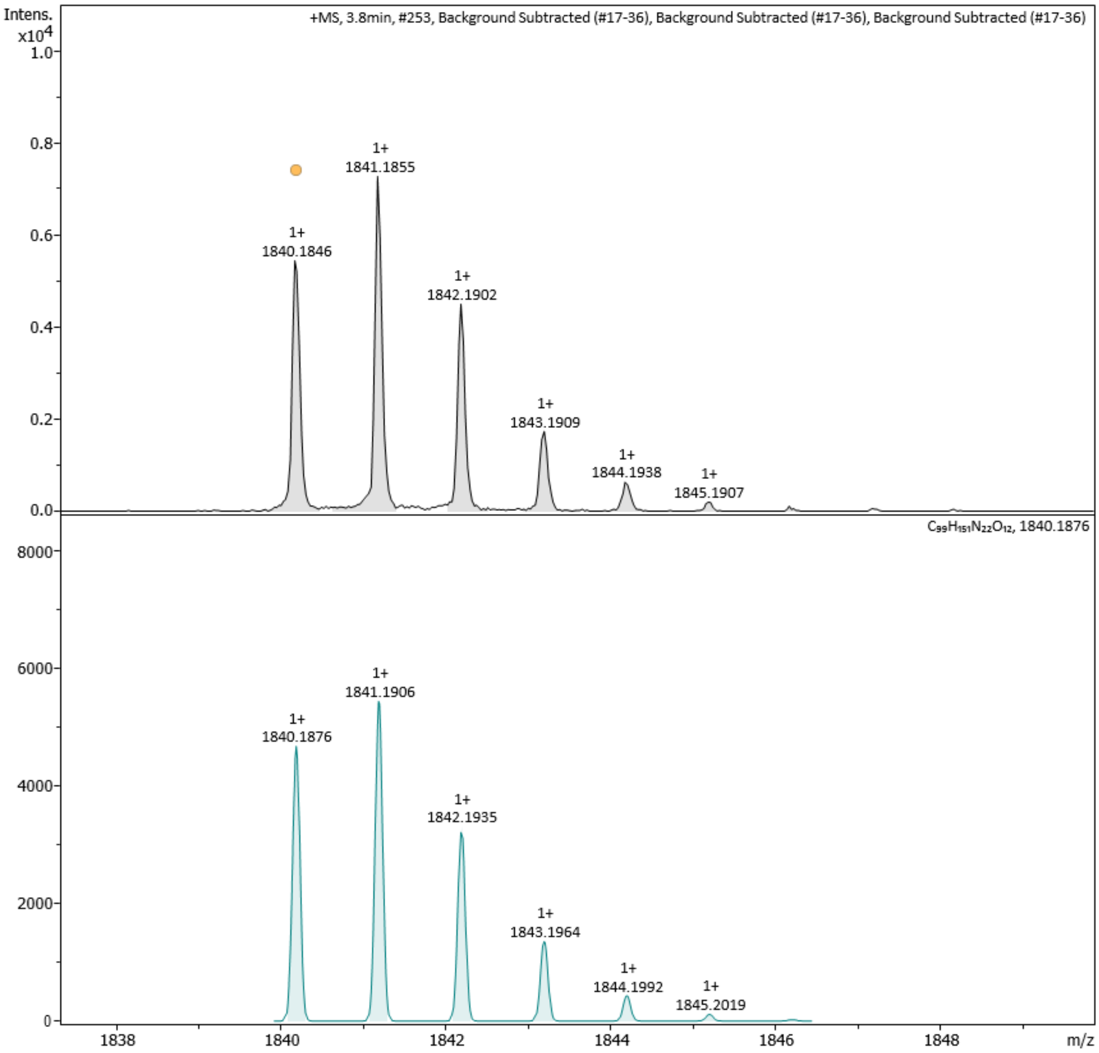

Meas. m/z	#	Ion Formula	Score	m/z	err [mDa]	err [ppm]	mSigma	rdb	e⁻ Conf	N-Rule
1840.1846	1	C99H151N22O12	100.00	1840.1876	3.0	1.6	44.1	36.0	even	ok

Supplementary Fig. 25. ESI-TOF exact mass analysis of D2-3.

A)

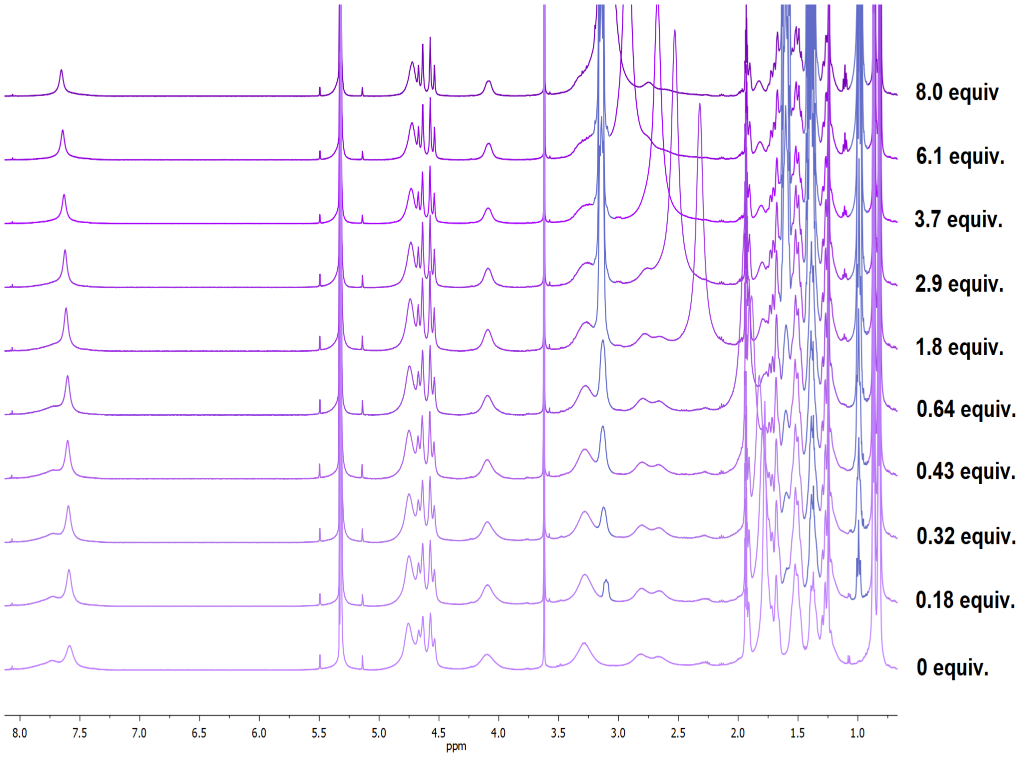

B)

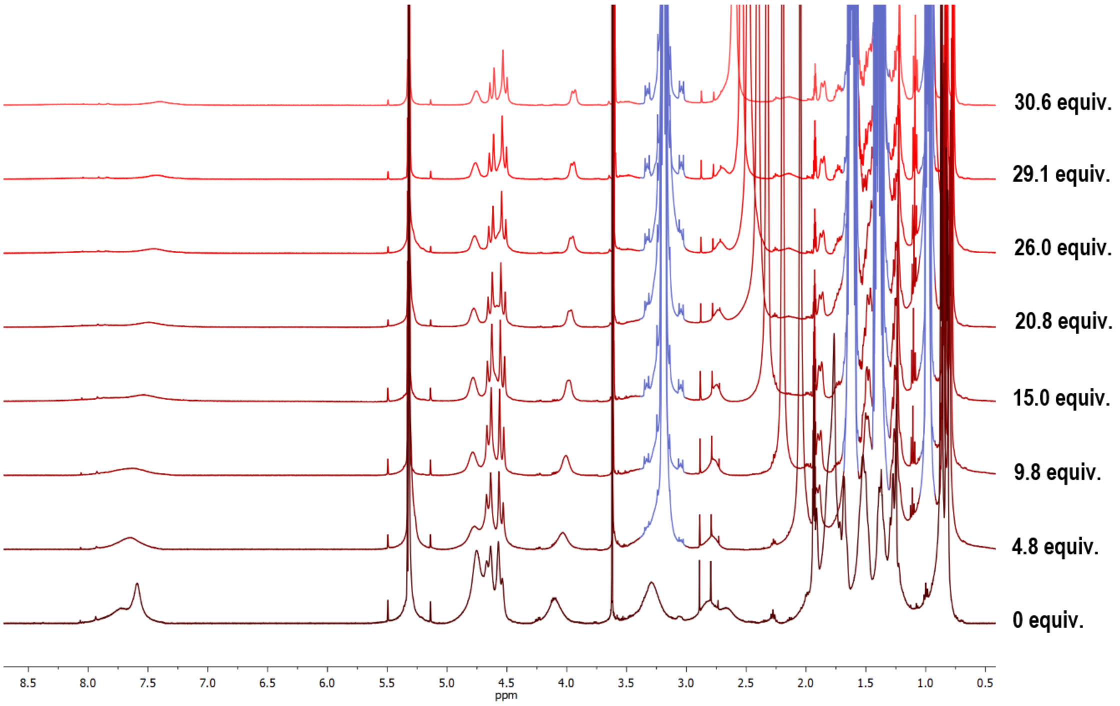

Supplementary Fig. 26. ^{1}H NMR spectra of titrations of D4 with TBAF and TBACl. (A) Stack of ^{1}H NMR spectra of a solution of **D4** ([**CP4**] = 6.1 mM) in 10% CD_3CN/CD_2Cl_2, containing dioxane as internal standard, after different additions of tetrabutylammonium fluoride (**TBAF**) stock solutions. (B) Stack of ^{1}H NMR spectra of a solution of **D4** ([**CP4**] = 6.4 mM) in 10% CD_3CN/CD_2Cl_2, containing dioxane as internal standard, after different additions of tetrabutylammonium chloride (**TBACl**) stock solutions. No changes similar to those found for **D2** could be observed. The main difference is the broadening of the signals after the addition of the salts which was attributed to added water due to the lower stability of **D4**.

A)

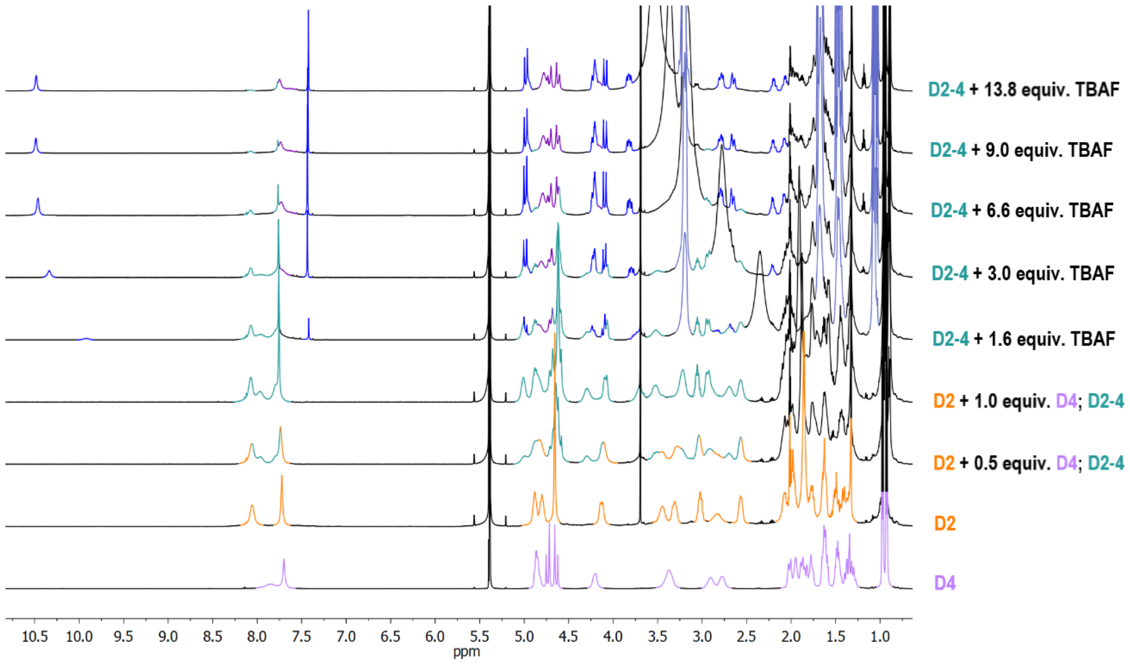

B)

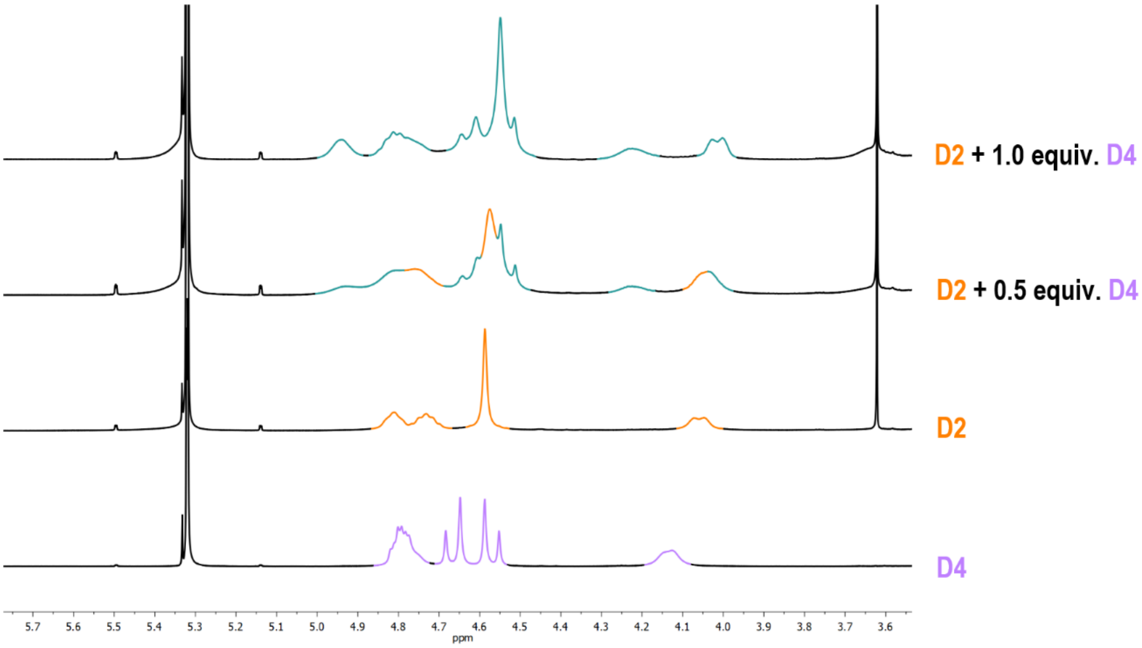

Supplementary Fig. 27. Formation of D2-4 heterodimer and subsequent addition of TBAF. (A) Stack of ^{1}H NMR spectra of a solution of **D2** ([**CP2**] = 8 mM) in 10% CD_3CN/CD_2Cl_2, containing dioxane as internal standard, after different additions. Initially **D4** was added over a solution of **D2** and then, different additions of tetrabutylammonium fluoride (**TBAF**) were done. The most relevant signals of free **D2** are labelled in orange, in lavender the signals corresponding to **D4** while those corresponding to the heterodimer (**D2-4**) are in teal color. Finally, after the addition of TBAF, the signals assigned to the **mF$^-$·nH$_2$O$\subset$2CP2** complex are shown in blue. Under this conditions, **D4** (in lavender) is recovered. (B) stacks of the 5.76–3.56 ppm region of the ^{1}H NMR spectra where the most remarkable signals attributed to the heterodimer are shown. For example, the broad signal at 4.95 ppm belongs to the new Hα_{Leu} from the **CP2** half of **D2-4** and the one at 4.22 ppm, to one of the methylene groups from the tris(triazolylethyl)amine cap from the **CP4** half of **D2-4**. For the full characterization of **D2-4** see sections 7 and 8.

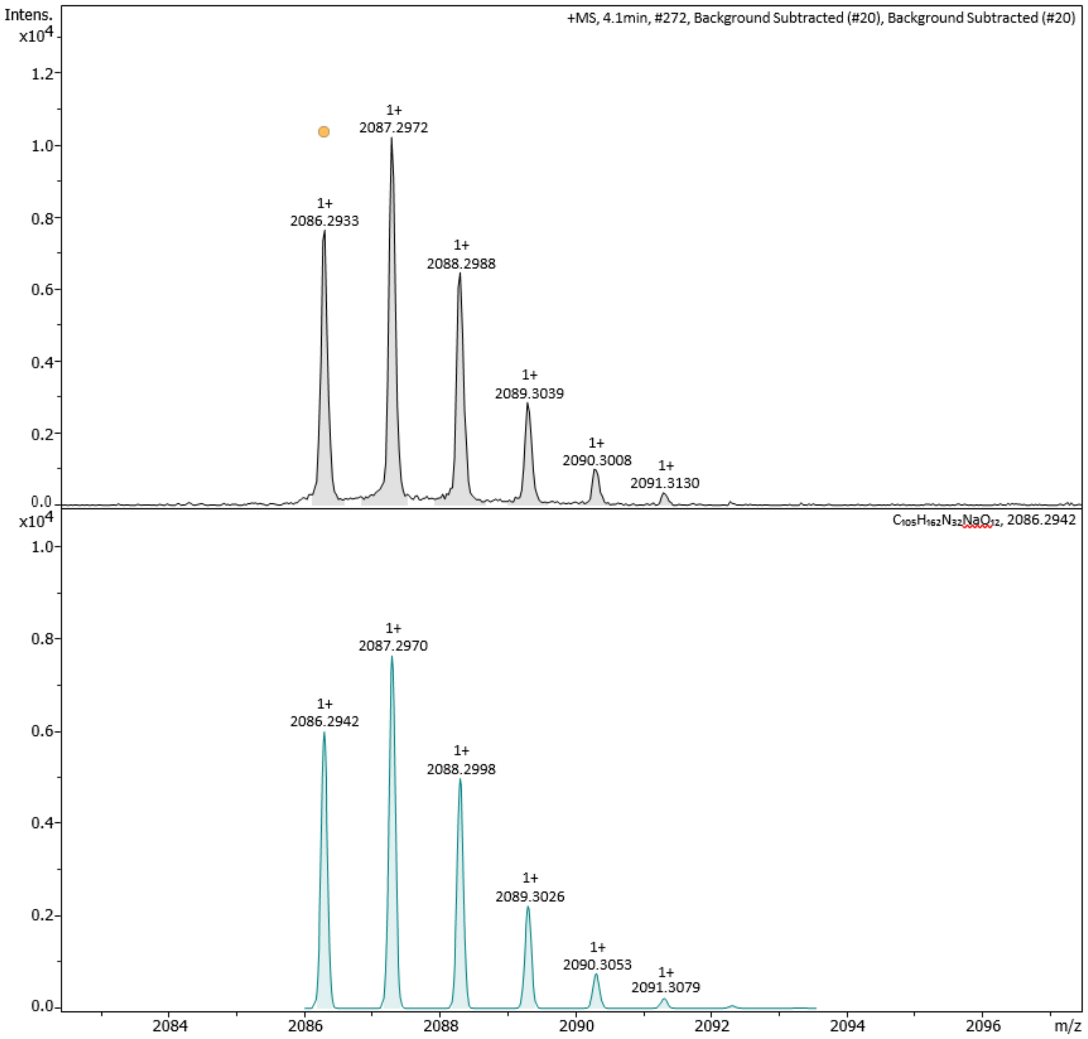

Meas. m/z	#	Ion Formula	Score	m/z	err [mDa]	err [ppm]	mSigma	rdb	e¯ Conf	N-Rule
2086.2933	1	$C_{105}H_{162}N_{32}NaO_{12}$	100.00	2086.2942	1.0	0.5	17.7	41.0	even	ok

Supplementary Fig. 28. ESI-TOF exact mass analysis of D2-4.

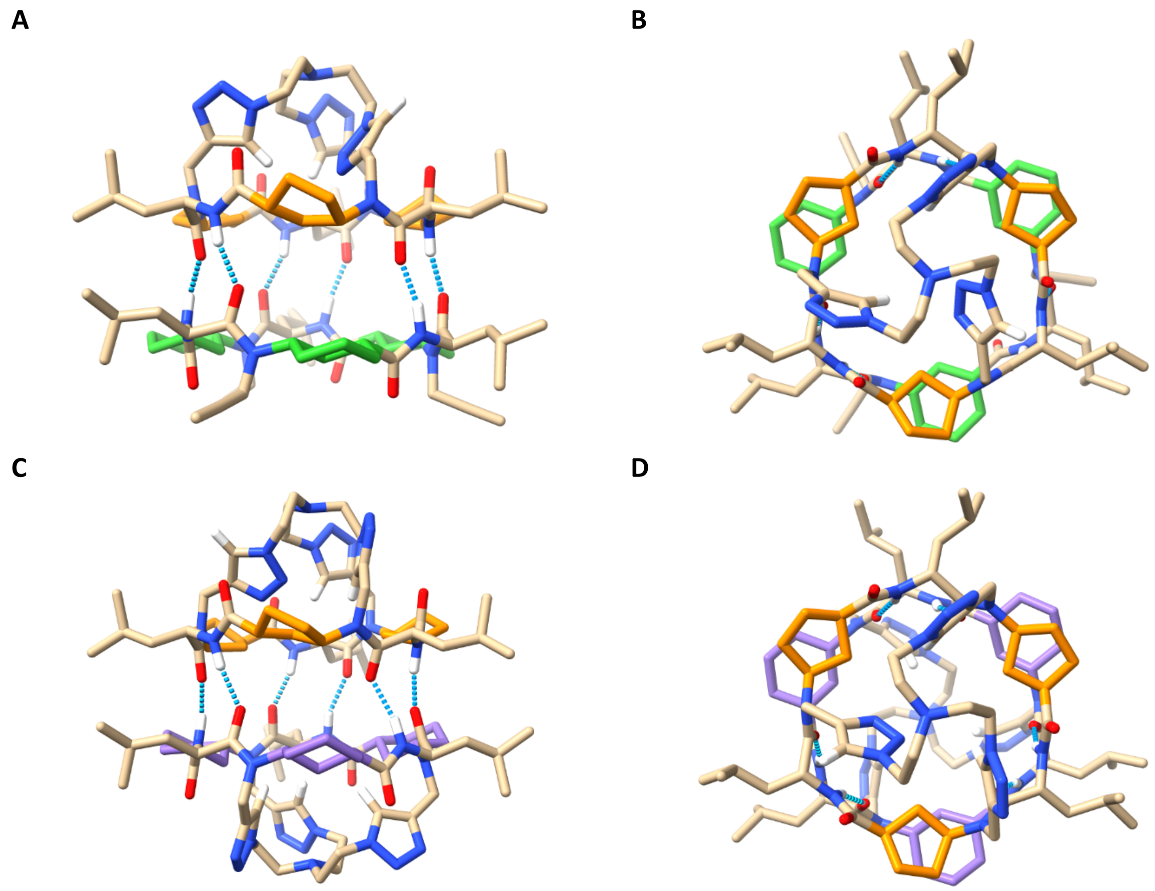

Supplementary Fig. 29. DFT geometry optimization of heterodimers D2-3 (A and B) and D2-4 (C and D). (A) side and (B) top view of the heterodimer **D2-3** (corresponding to the specie depicted in red wine in Supplementary Fig. 24); (C) side and (D) top view of the heterodimer **D2-4** (corresponding to the species depicted in teal color in Supplementary Fig. 27). The level of calculation employed was B3LYP/6-31G(d,p), including GD3BJ as dispersion. Notice that in both structures, **CP2** has one of the triazole proton pointing outwards the cavity, as it was found in the crystal structure of **D2**. The nitrogen atoms are depicted in blue, oxygens in red and hydrogens in white, as well as carbons in beige. The carbons from the five-member ring Acp-residues are depicted in orange, while six-member ring Ach-residue carbons are depicted in green for the uncapped **CP3** half of **D2-3** and in lavender for the **CP4** half of **D2-4**.

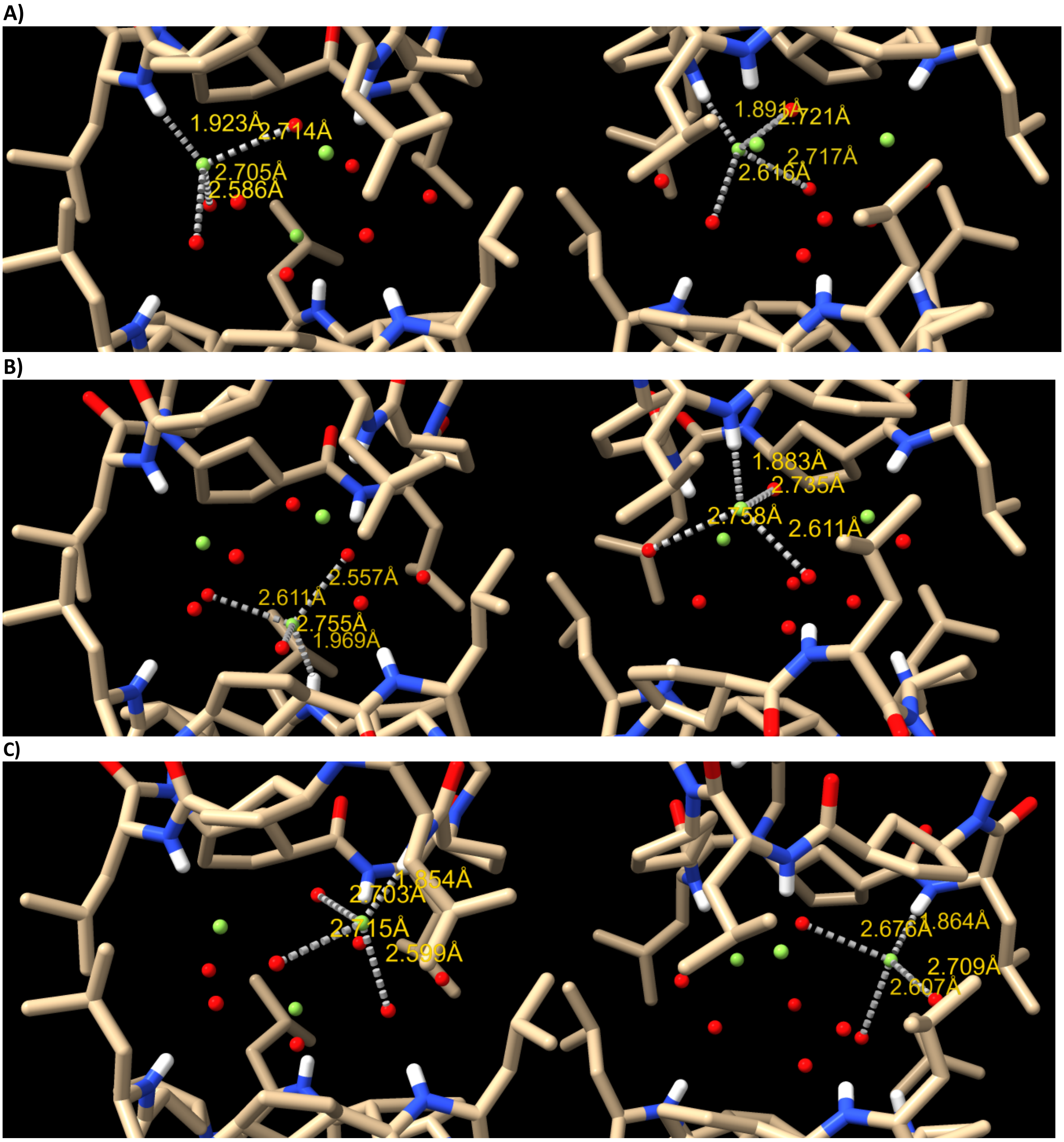

Supplementary Fig. 30. Fluoride hydration shell in $3F^{-}{\cdot}8H_2O \subset 2CP2$. View of the fluoride cluster in the crystal structure in which each fluoride is surrounded by three water molecules. The two **$3F^{-}{\cdot}8H_2O \subset 2CP2$** complexes are displayed. The distances are indicated for both of them, starting from the fluoride on the left (A), middle (B) and right (C) side of the view. The NH···F distances are also given. The nitrogen atoms are depicted in blue, oxygens in red and hydrogens in white, as well as carbons in beige and fluorides in green.

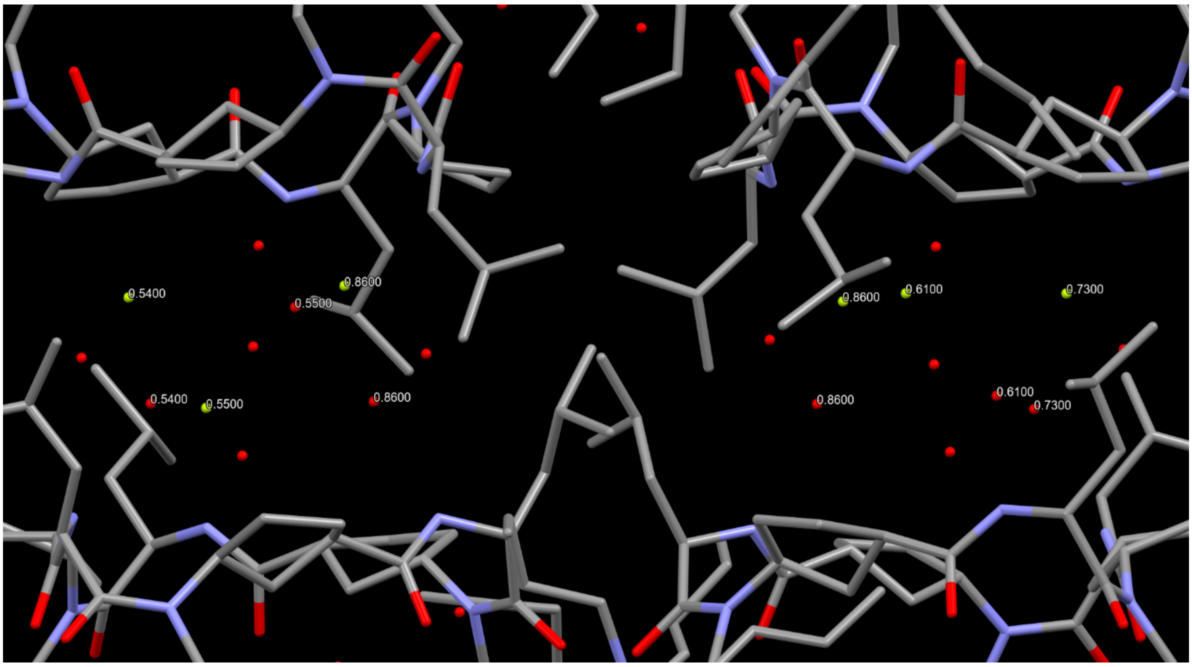

Supplementary Fig. 31. Fluoride occupancy in the two 3F^{-}·8H$_2$O⊂2CP2 that crystalized together. The nitrogen atoms are depicted in blue, oxygen in red and hydrogens in white, as well as carbons in grey and fluorides in green.

A)

B)

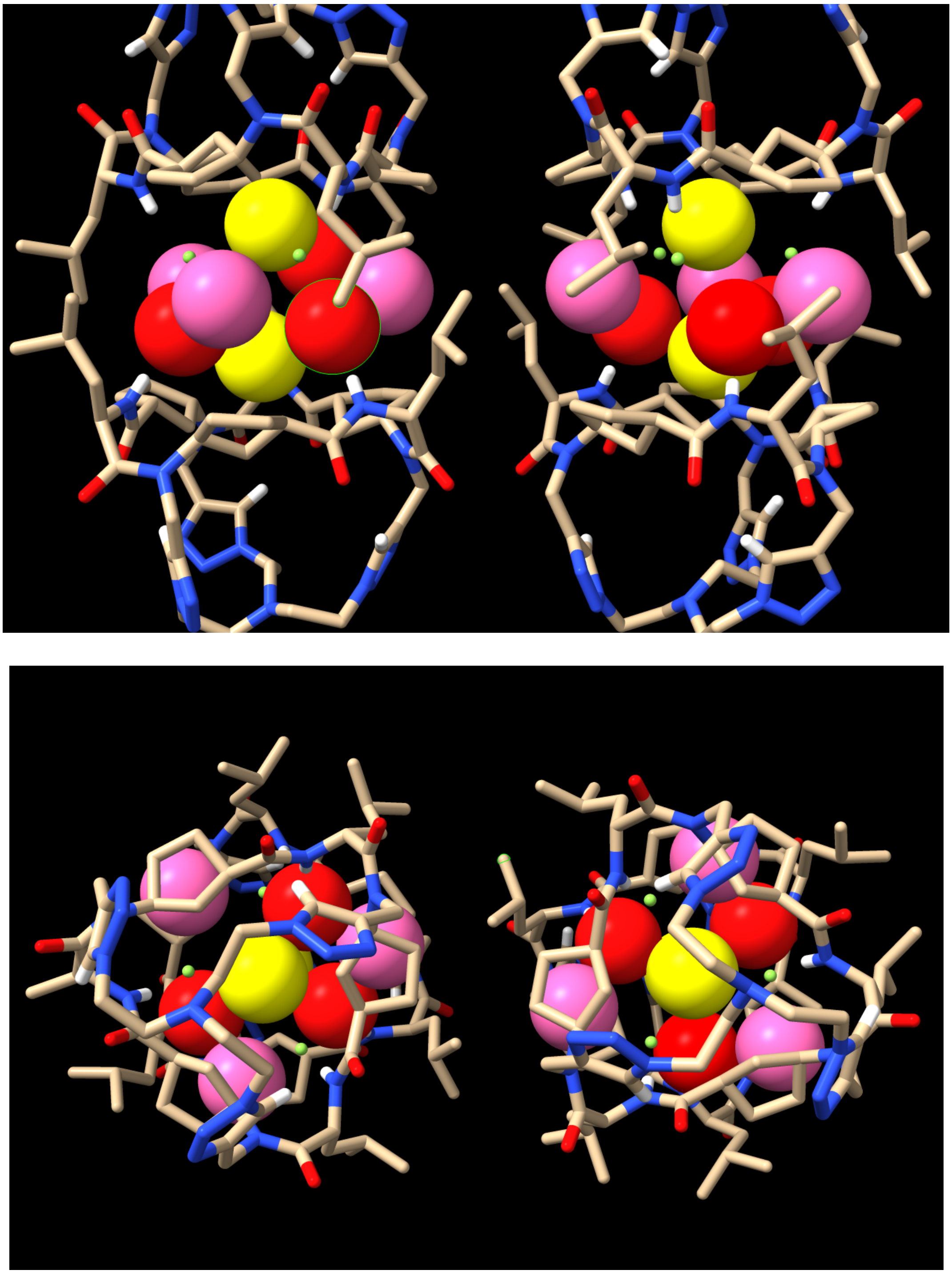

Supplementary Fig. 32. Apical and equatorial water molecules in $3F^{-}{\cdot}8H_2O{\subset}2CP2$. Side (A) and top (B) view of the complex $\mathbf{3F^{-}{\cdot}8H_2O{\subset}2CP2}$. The water molecules are depicted in CPK model. In yellow, the two apical water molecules, which are the only two water molecules hydrogen bonded to different fluorides. In pink, the equatorial ones. In light green, the fluoride atoms. In red, the three water molecules that share disordered positions with the three fluoride anions. By other side, the nitrogen atoms are depicted in blue, oxygen in red and hydrogens in white, as well as carbons in beige.

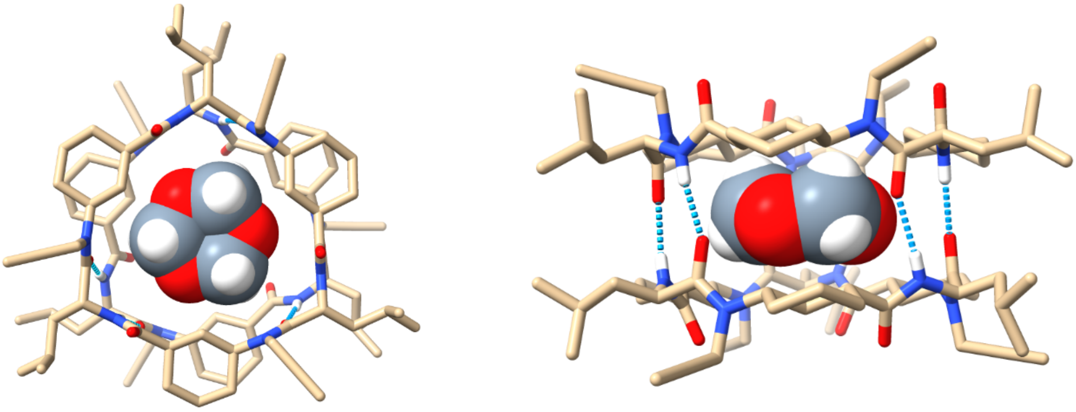

Supplementary Fig. 33. **X-Ray structure of D3**. Side (left) and top (right) view of the crystal structure of **D3** with a disordered dioxane molecule along a crystallographic 3-fold symmetry axis. The dioxane molecule is represented in a CPK model and is entrapped in between the two units. The nitrogen atoms are depicted in blue, oxygens in red and hydrogens in white, as well as carbons in beige.

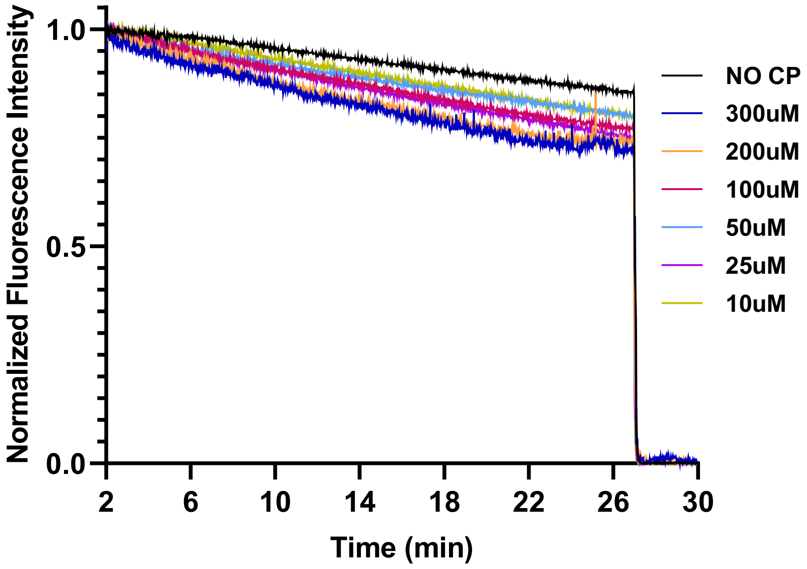

Supplementary Fig. 34. **Lucigenin assay with D4**. Representative normalized fluorescence kinetics of transport-mediated experiments using different concentration of the Ach-based peptide (the concentrations correspond to **CP4**) in which almost no quenching on the lucigenin emission was found even at concentration as high as 300 µM. It should be mentioned that at concentrations higher than 200 µM some precipitation is already detected, while **CP2** did not precipitate even at 650 µM. For this experiment λexc = 450 nm and λem = 535 nm.

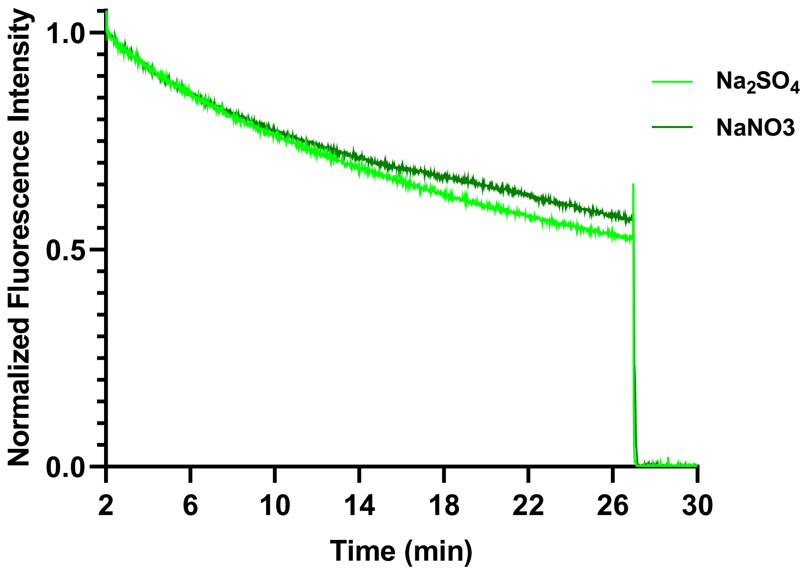

Supplementary Fig. 35. **Lucigenin assays with D2 using $NaNO_3$ or Na_2SO_4.** Effect of the nature of the intra- and extra-vesicular aqueous solution on the transport of **CP2** (50 μM), in which isosmotic buffer solutions of $NaNO_3$ or Na_2SO_4 were compared. No differences between both experiments were found, confirming that anion transport is not based on the antiport mechanism.

A) LUCIGENIN ASSAY

Particle Size

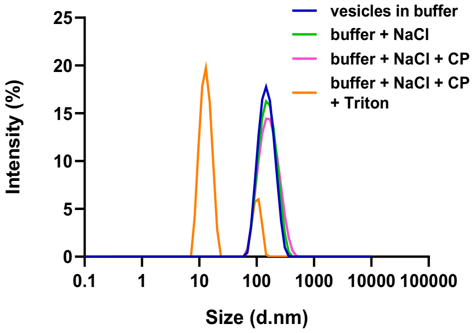

Correlation Coefficient

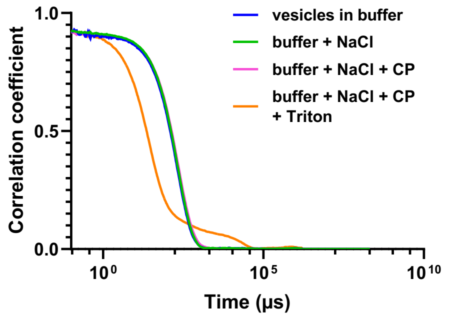

Particle Concentration

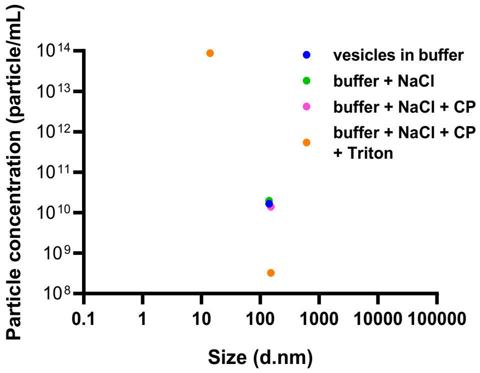

Control Values

	Mean	St. dev.	RSD	Min	Max
Z-Average	146.6	1.173	0.801	145.2	147..5
Polydispersity Index (PI)	0.1043	0.0076	7.312	0.0992	0.1131
Particle concentration (particle/mL)	$1.65x10^{10}$	-	-	-	-

B) HPTS ASSAY

Particle Size

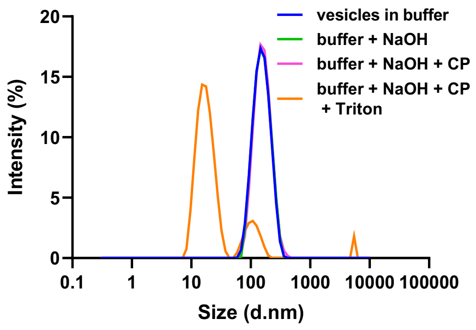

Correlation Coefficient

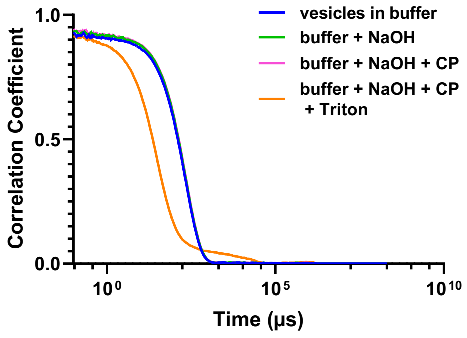

Particle Concentration

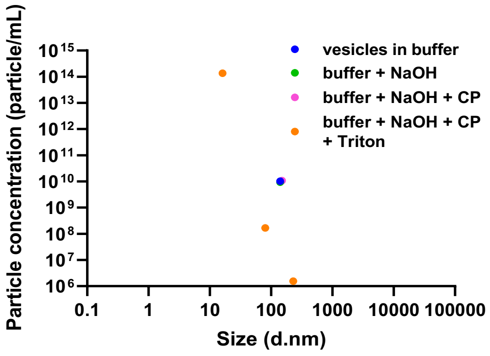

Control Values

	Mean	St. dev.	RSD	Min	Max
Z-Average	146.1	1.721	1.17	144.2	147.6
Polydispersity Index (PI)	0.0873	0.0077	8.906	0.0808	0.0960
Particle concentration (particle/mL)	$1.02x10^{10}$	-	-	-	-

Supplementary Fig. 36. DLS measurements in lucigenin and HPTS assays. Particles size, Correlation coefficient, particle concentration and control values are given for each vesicle batch.

2. MATERIALS

All solvents and chemicals were purchased from commercial sources and used without further purification unless otherwise stated. CH_2Cl_2 and DIEA were distilled from CaH_2 under argon immediately prior to their use whenever anhydrous conditions were required. Analytical thin-layer chromatography was performed on E. Merck silica gel 60 F254 plates. Compounds which were not UV active, were visualized by dipping the plates in a ninhydrin or phosphomolybdic acid solution and heating. Silica gel flash chromatography was performed using E. Merck silica gel (type 60SDS, 230-400 mesh). Solvent mixtures for chromatography are reported as v/v ratios.

For the preparation of vesicles, a solution of EYPC in chloroform (25 mg/mL) from Avanti Polar Lipids was used. Sephadex G-25 from Sigma Aldrich was used as stationary phase to elute the vesicle dispersion. Milli-Q water was used for the preparation of all the aqueous solutions.

Hamilton microsyringes were used for the preparation of all the solutions for the NMR titration experiments as well as for the transport experiments.

^1H NMR spectra were recorded on AVIII 500 MHz, NEO 750 MHZ or Varian Mercury 300 MHz spectrometers. Chemical shifts (δ) were reported in parts per million (ppm) relative to the deuterated solvent employed. ^1H NMR splitting patterns are designated as singlet (s), doublet (d), AB quartet (AB), triplet (t), or doublet of doublets (dd). All first order splitting patterns were assigned on the basis of the appearance of the multiplet. Splitting patterns that could not be easily interpreted are designated as multiplet (m) or broad (br). ^{13}C NMR spectra were recorded on AVIII 500 MHz. Carbon resonances were assigned using either Distortionless Enhancement by Polarization Transfer (DEPT) spectra obtained with phase angles of 135° or by Heteronuclear Single Quantum Coherence experiments (HSQC). ^1H NMR spectra signals of the peptides were identified from the corresponding double-quantum-filled 2D COSY, TOCSY, HSQC, NOESY and/or ROESY spectra acquired at concentration and temperature indicated (mixing times for ROESY are 350 ms).

Electrospray (ESI) mass spectra were recorded on a Bruker BIOTOF II mass spectrometer and are reported as mass-per-charge ratio *m/z*. Accurate mass determination (HRMS) using ESI-MS were performed on a Sciex QSTAR Pulsar spectrometer.

FT-IR measurements were made on a JASCO FT/IR-400 spectrophotometer placing the sample on a CaF_2 pellet or on a Perkin Elmer Spectrum Two ATR-FTIR, directly depositing the sample as a thin film over its diamond plate (neat).

Fluorescence assays were performed on a FluoroMax-3 spectrofluorometer (Jobin-Yvon Spex) equipped with a stirrer and a temperature controller (all experiments were carried out at 25 °C). All measurements were carried out as duplicates or triplicates.

LUV extrusion procedure was carried out utilizing a Mini-Extruder set purchased from Avanti Lipids, as well as filter supports and 200 nm polycarbonate membranes.

DLS control vesicles measures were done in a MALVERN ZetaSizer ULTRA using plastic fluorescence cuvette (12.5 x 12.5 x 45 mm, 2.5 mL capacity).

The products **CP1-CP4** were purified using an Agilent preparative HPLC 1260 Infinity II with a Waters SunFire preparative 5µ Silica, 19 x 150 mm column.

Boc-*L*-Ace-OH and Boc-*L*-Ach-OH enantiopurity was checked on an Agilent Super Fluid Chromatography (SFC) HPLC with a Lux® 5µ i-cellulose-5, 250 x 4.6 mm column.

3. SUPPEMENTARY DISCUSSION I: ^{1}H NMR TITRATION PROCEDURES

In general, for titration experiments the samples were prepared as indicated in the *Methods* section of the manuscript. Additional details about the titration experiments are included below.

TITRATIONS WITH THE TBA^{+} SALTS

To carry out the experiments with the tetrabutylammonium (TBA) salts in a more reproducible manner, different stock solutions were prepared to carry all the additions. Commercial tetrabutylammonium salts (F^-, Cl^-, Br^-, I^-, NO_3^-, N_3^-, AcO^-, Br_3^- and PF_6^-) were used. These stock solutions were prepared to adjust each of the additions to 10 µL, containing 0.2 equivalents (for the most dilute titrations), 0.5, 1.0, 3, 5 or even 10 equivalents for those experiments with the anions with lower affinity. During these titrations, samples were diluted around 12-15%. This dilution factor was considered during the normalization process, using the internal standard (Dioxane or TMSS), to calculate the concentration of all the species through the titration (free dimer, complex and TBA^{+} salts).

TITRATIONS AFTER HETERODIMER FORMATION

For the experiments of heterodimers formation, only one stock solution of the titrated peptides (**D3** or **D4**) was prepared employing the mixture of solvents that already contains the internal standard. This stock solution was more concentrated (60 mM), so that the heterodimeric species could be formed after only two additions of 19 µL of each peptide solution. (D3 or D4)

TITRATIONS WITH MALONONITRILE AND SUCCINONITRILE

The experiments with malononitrile (**MN**) and succinonitrile (**SN**) were carried out following a procedure similar to the anion additions. Several stock solutions of both nitriles were prepared, and upon analysis of the behavior of **D2** with the more diluted ones, we assessed the suitable stock solutions of nitriles to use. For both experiments TMSS was used as internal standard. CD_3CN was not used in these experiments to avoid competition with the titrated nitriles.

TITRATION OF **D2** WITH NaOAc/15-CROWN-5 ETHER

The experiment with NaOAc and the 15-crown-5-ether was performed in a slightly different manner as compared to previous titrations. The stock solution of the salt was prepared by dissolving the crown ether (1.1 equivalents with respect NaOAc) in of CD_3CN (440 µL). The CD_3CN was chosen as the best, non-competitive solvent to solubilize the mixture of crown ether and NaOAc. Subsequently, NaOAc (206 mg) was added, and the mixture stirred until a clear and colorless solution was obtained. Different additions of this solution were made on the NMR tube containing the solution of **D2** (~5mM, CD_2Cl_2).

Approximately, 10 µL of this solution contains 10 equivalents of acetate. However, some precipitation was observed during the additions, suggesting that not all the acetate was soluble in the final sample mixture, reducing the amount of available acetate.

4. SUPPLEMENTARY DISCUSSION II: COMPLEX STRUCTURE ANALYSIS, NMR AND X-RAY DATA COMPARISON.

NMR COMPLEXES CARACTERIZATION

After carrying out the ^{1}H NMR titrations of **D2** with different salts, we realized that the signals of the assembled complexes, although having chemical shifts with variations, were really similar. Therefore, we believe that the geometry of the complexes is similar. A detailed analysis of the spectra of these complexes, specifically of the ROESY, was carried out, comparing them with each other and with that of **D2**.

Initially, we began to compare the ROESY spectrum of **D2** with that of the complex formed after the addition of fluoride [**$3F^{-}$·nH_2O⊂2CP2**]. This was specially selected since it required adding the smaller amount of salt, so its spectra presented a better resolution. To follow the analysis, see Supplementary Fig. 37, where the most significant signals of the protons of **D2** and fluoride complex are assigned, and sections 7 and 8, where the complete characterization of mentioned species is included. In this discussion, it is a question of relating the NMR information with the crystallographic data to determine the structural coincidence between both sources.

1D-NMR OBSERVATIONS

The most relevant change in chemical shift and signal appearance includes the NH proton, which suffers a sharp down-field shift from 8.03 ppm in **D2** to 10.00 ppm after the addition of three equivalents of fluoride that is related with the formation of the complex [**$3F^{-}$·nH_2O⊂2CP2**]. However, the triazole proton suffers a down-field shift from 7.71 to 7.78 ppm. For **D2**, the signals of protons of the methylene, which links the triazole ring and the cyclic peptide backbone, appear as a singlet at 4.59 ppm that is split into two doublets, 4.94 and 4.05 ppm, upon complex formation. $H\alpha_{Leu}$, which initially resonates at 4.81 ppm, undergoes a noticeable up-field shift to 4.15 ppm (for fluoride), which is the typical behavior in α-helix, [1,2,3,4] while the $H\gamma_{Acp}$, initially at 4.73 ppm, undergoes the opposite response, shifting is signal to down-field up to 4.90 ppm. These two changes can be correlated with the observed structural changes in the crystal structure, since in **ACN⊂D2**, $H\alpha_{Leu}$ was exposed to the aromatic electron density of the triazole ring, while it aligns with the oxygen of carbonyl group of the Acp moiety. However, the capsule deformation associated with the recognition process leaves that proton ($H\alpha_{Leu}$) facing away from the

peptide backbone and thus unable to stablish the aforementioned interactions. In contrast, $H\gamma_{Acp}$ is now exposed to the electronic density of the anion (≈ 2.7 Å). Similar behavior is experimented by $H\alpha_{Acp}$, whose signal also down-field shifts from 2.96 to 3.20 ppm.

With respect to the four protons of the ethylene moieties corresponding to the tris(triazolylethyl)amine cap, we have named them in this study as H_A and H_B, for those on one carbon, and H_C and H_D, for those on the other. In the **D2** spectra, H_A and H_B appear at 4.09 (dt) and 3.40 (ddd) ppm ($\Delta\delta_{A-B}$ = 0.69 ppm), respectively. H_C and H_D also resonate at very different chemical shifts, 3.25 (ddd) and 2.85 (dt) ppm ($\Delta\delta_{C-D}$ = 0.67 ppm), respectively, suggesting that they are pointing at very different environments (See the full NMR spectra data in sections 7 and 8). The A-B and the C-D pairs were differentiated based on the analysis of their nOe cross-peaks of triazole proton, whose strongest observed cross-peak is with H_B. Therefore, H_A and H_B must be the protons of the closest carbon to the triazole ring, while H_C and H_D are the protons corresponding to the carbon closest to the nitrogen of the tertiary amine. It is noteworthy that the triazole proton presents nOe cross-peaks with the four protons, although in the X-ray structure H_C and H_D are oriented in the opposite direction to the triazole ring. This fact confirms the flexibility of the molecular cap that allows some conformational freedom of the ethylene moiety.

After the formation of the complex, the four cap signals maintain the same multiplicity pattern as in **D2**. A double triplet (dt) appears at 4.19 ppm, and its geminal proton appears at 3.80 as a doublet of doublets of doublets (ddd). The others methylene protons appear at 2.74 (ddd) and 2.61 (dt). It is important to note that, although we take the complex of **$3F^{-}\cdot nH_2O\subset 2CP2$** as a model for this analysis, in all the complexes studied, this set of characteristic signals have a similar behavior, except for small differences in the chemical shifts.

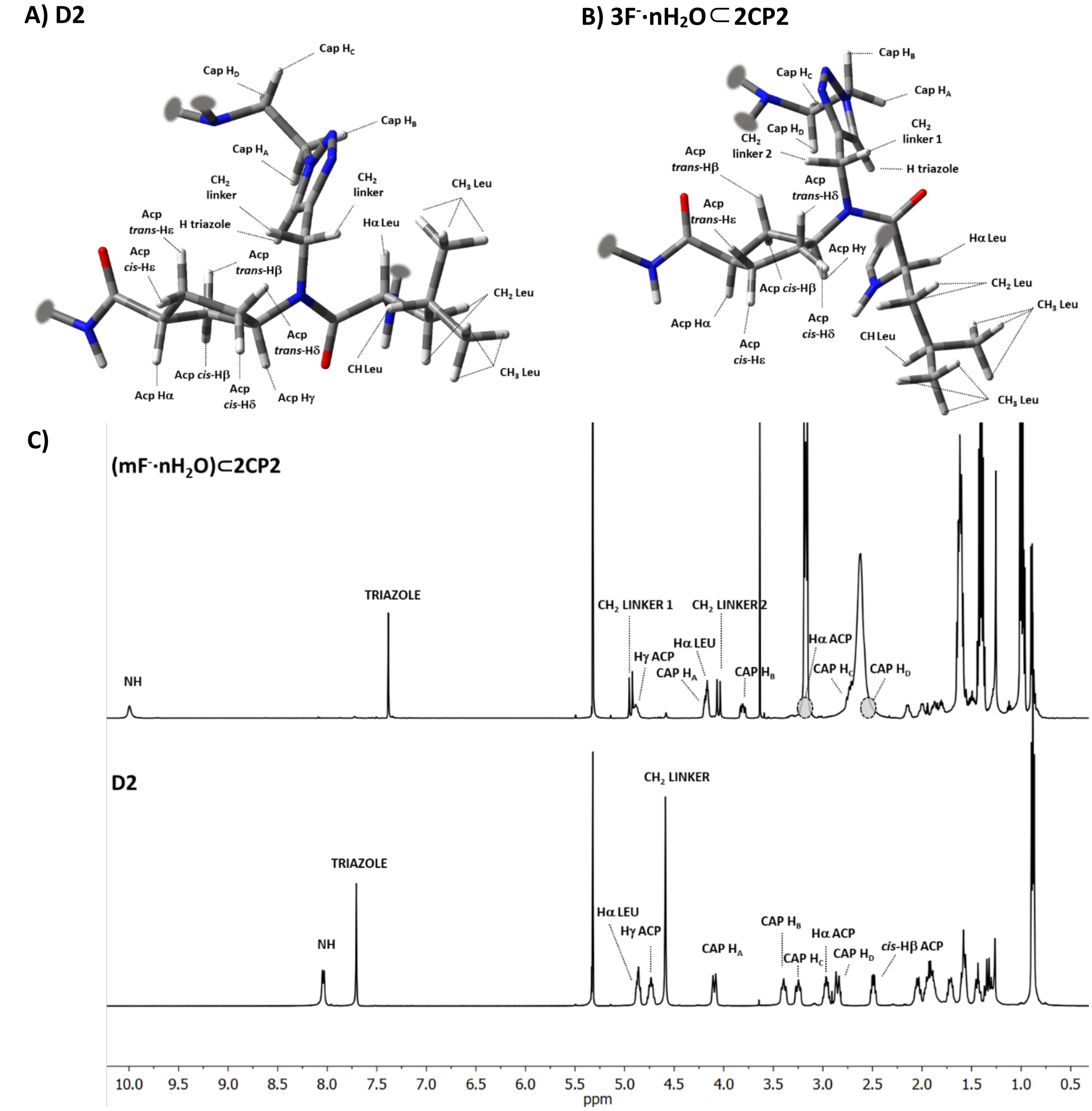

Supplementary Fig 37. **Assignation of protons on the NMR spectra of D2 and the complex $3F^{-}\cdot nH_2O\subset$ 2CP2.** A) and B) show the 3D-structure proton labels; C) show the signals corresponding to those protons in solution ^{1}H NMR (10% CD_3CN/CD_2Cl_2 as solvent).

In addition, both $H\beta_{Acp}$, originally at 2.50 (*cis*) and 1.34 (*trans*) ppm that are up-field shifted upon anion recognition to 1.57 (*trans*) and 1.39 (*cis*) ppm. These protons were pointing into the peptide cavity before the formation of the complex. Once the complex is formed, they are, according to the X-ray structure, pointing to the top of the capsule. This can be correlated with the changes in nOe cross-peaks with $H_{triazole}$, for both *cis* and *trans* Hβ. Thus, *cis*-$H\beta_{Acp}$ is not anymore affected by the interaction with carbonyl oxygen of Leu because the carbonyl moiety is now pointing outwards of the cavity. In contrast, *trans*-$H\beta_{Acp}$ is now aligned with the oxygen of carbonyl group of Acp residue that match with the observed down-field shift of this signal.

2D-NMR OBSERVATIONS

Apart from the already mentioned changes in the ^{1}H NRM between both species, the recognition process, according to the X-ray information, has widely altered the peptide skeleton conformation. These changes can also be correlated with the ROESY spectra (See Supplementary Fig. 10 for nOe signals, Supplementary Fig. 39 for superimposed ROESY spectra of **D2** and **3F⁻·nH₂O⊂2CP2**, and sections 7 and 8 for full characterization).

The distance, measured on the crystal structure of **ACN⊂D2**, between the triazole proton and the $H\alpha_{Leu}$ is 2.95 Å, which is consistent with the strong nOe cross-peak found on the ROESY experiment. On the other hand, in the spectra of the fluoride complex, a clear reduction in the nOe signal between both protons is observed, something that agrees with the measured increase of this distance in the crystal structure. Furthermore, no nOe was observed between $H_{triazole}$ and the protons of the methylene linker (the connector between the CP backbone and the tris-triazole cap), consistent with the X-ray data, suggesting less conformational freedom of the structure of the complex. In addition, the cross-peaks changes in the protons of the methylene linker also provide additional structural information. The short distance (2.45 Å) between these protons and the methyl groups of Leu side chain obtained from crystal structure also correlates quite well with the strong nOe found between these protons in solution. In contrast, once the complex is formed, this cross-peak disappears, as would be expected considering the longer distance between these protons in the solid state (6.52 Å).

In addition, there is another key change with respect to the $H\gamma_{Acp}$ that also matches with the observed in the X-ray structure, cause a new nOe peak with the methylene of Leu side chain was found upon complexation. This is also in agreement with the structural changes found in both structures in which the twisting of the Leu side chain after the complexation leaves its methylene oriented towards $H\gamma_{Acp}$, whereas before complexation it was oriented in the opposite direction towards the Acp ring.

Finally, the NH proton also provides important information. In the dimeric structure, the distance between this proton and the $H\gamma_{Acp}$ in about 4.6-4.8 Å for the one belonging to the same CP moiety, and only 3.7 Å with the $H\gamma_{Acp}$ corresponding to the other CP. Although it has been confirmed the dimeric structure by different techniques (MS or X-Ray), no nOe cross-peak between these two protons was found, something that already happened in previously reported dimeric structures. However, a strong nOe cross-peak was observed in the chloride complex in the complex structure (See Supplementary Figures 10, 39 and 41), that matches quite well with the structural deformation suffered in the peptide backbone in which NH···Hγ distance is reduced to ca. 2.2 Å. Such peak, although the proposed complexes have similar conformation, was not found in the fluoride complex, but this proton is not giving any nOe with any other proton.

BEFORE COMPLEXATION	AFTER COMPLEXATION
**TRIAZOLE PROTON**	
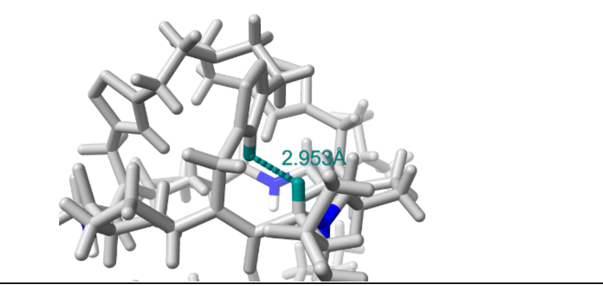	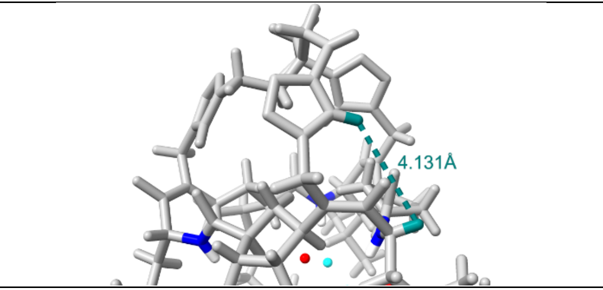
**METHYLENE LINKER**	
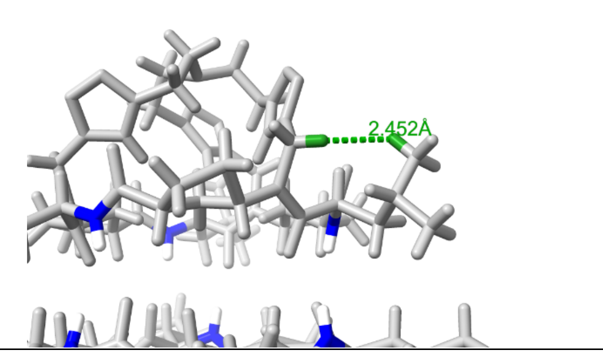	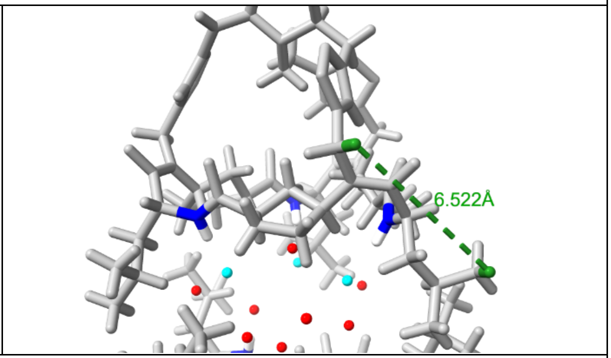
**Hγ ACP**	
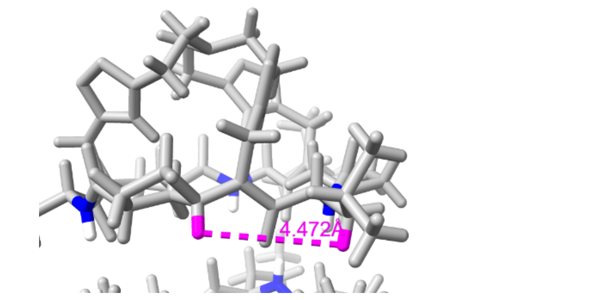	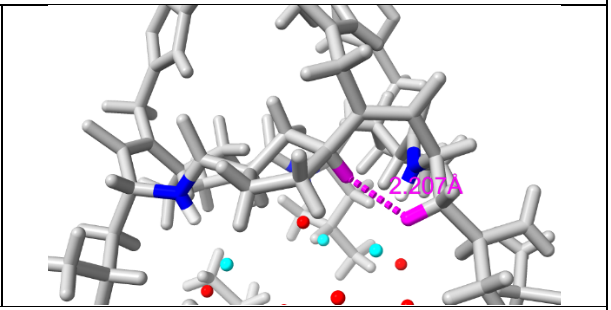
**NH**	
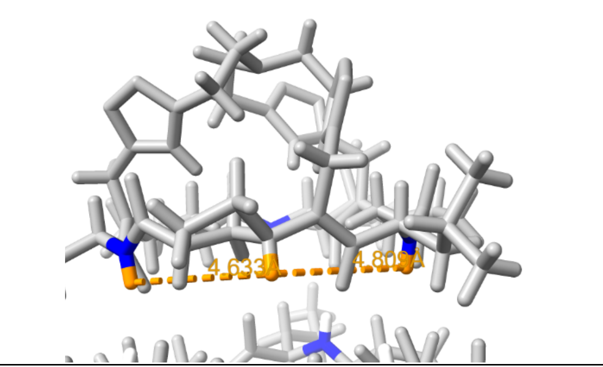	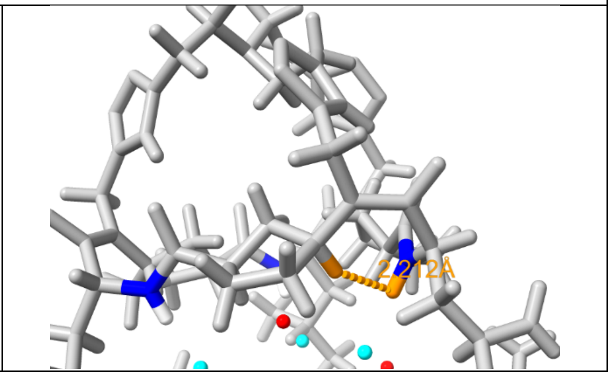

Supplementary Fig. 38. Distances changes (Å) between different protons of CP2 before and after the fluoride complexation. Notice the correlation with the signals highlighted in Supplementary Fig. 10, where the color code has been maintained. The rest of the peptide backbone is depicted in grey, except for the amidic nitrogens and protons, depicted in blue and white, respectively. Full crystallography data of both species can be obtained free of charge from The Cambridge Crystallographic Data Centre via https://www.ccdc.cam.ac.uk/structures, CCDC-2311118 and CCDC-2311117.

Even though this analysis was performed using as reference the ^{1}H NMR spectrum of the fluoride complex **$3F^{-}\cdot nH_2O \subset 2CP2$** (except as mentioned regarding the nOe of amide proton), the conclusions derived from this analysis can be extended to the rest of complexes since most of the mentioned changes, excluding the small differences in chemical shifts (See Supplementary Fig. 40 and 44), are also observed in the other complexes. Furthermore, ROESY spectra shows similar structural orientation as it is illustrated in Supplementary Fig. 41 and 42, in which the spectra of two different complexes are overlapped.

D2/$3F^{-}\cdot nH_2O \subset 2CP2$

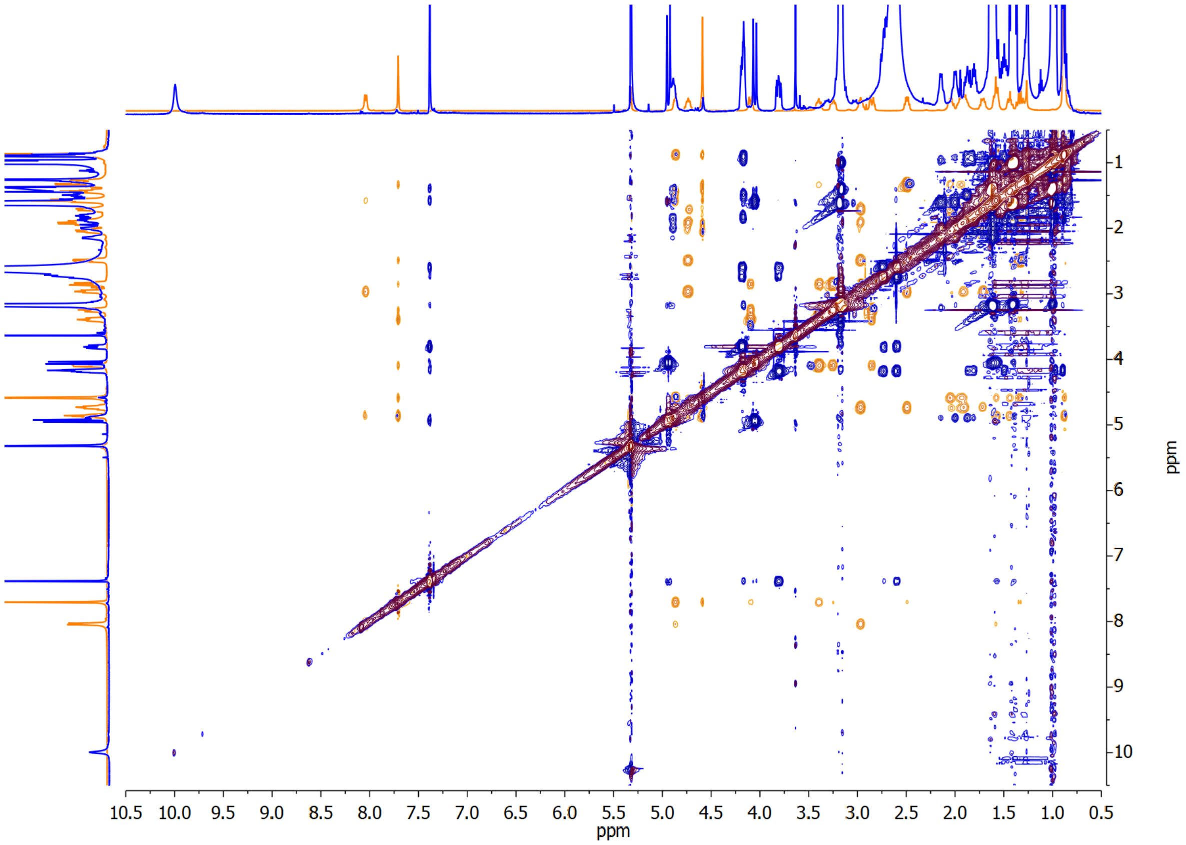

Supplementary Fig. 39. Superimposed ROESY spectra of D2 (orange) and the fluoride complex $3F^{-}\cdot nH_2O \subset 2CP2$ (blue). For **D2** spectra CD_2Cl_2 was employed as solvent, while **$3F^{-}\cdot nH_2O \subset 2CP2$** spectra was recorded in CD_3CN/CD_2Cl_2 (10%).

D2/3Cl$^-$·nH$_2$O⊂2CP2

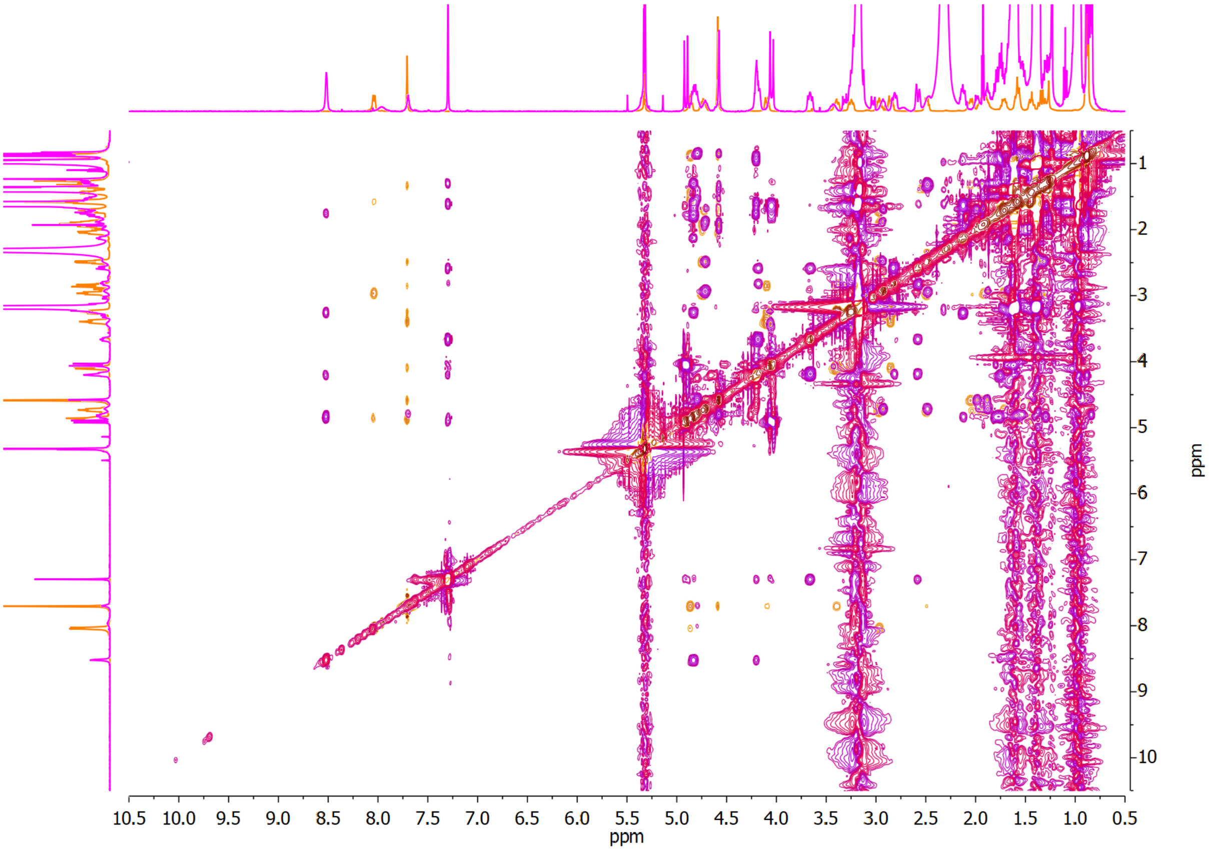

Supplementary Fig. 40. Superimposed ROESY spectra of D2 (orange) and the chloride complex 3Cl$^-$·nH$_2$O⊂2CP2 (magenta). Notice that in the spectrum of the chloride complex also contains some signal corresponding to the remaining free **D2**. For **D2** spectra CD_2Cl_2 was employed as solvent, while **3Cl$^-$·nH$_2$O⊂2CP2** spectra was recorded in CD_3CN/CD_2Cl_2 (10%).

$3Cl^{-}\cdot nH_2O\subset 2CP2/3F^{-}\cdot nH_2O\subset 2CP2$

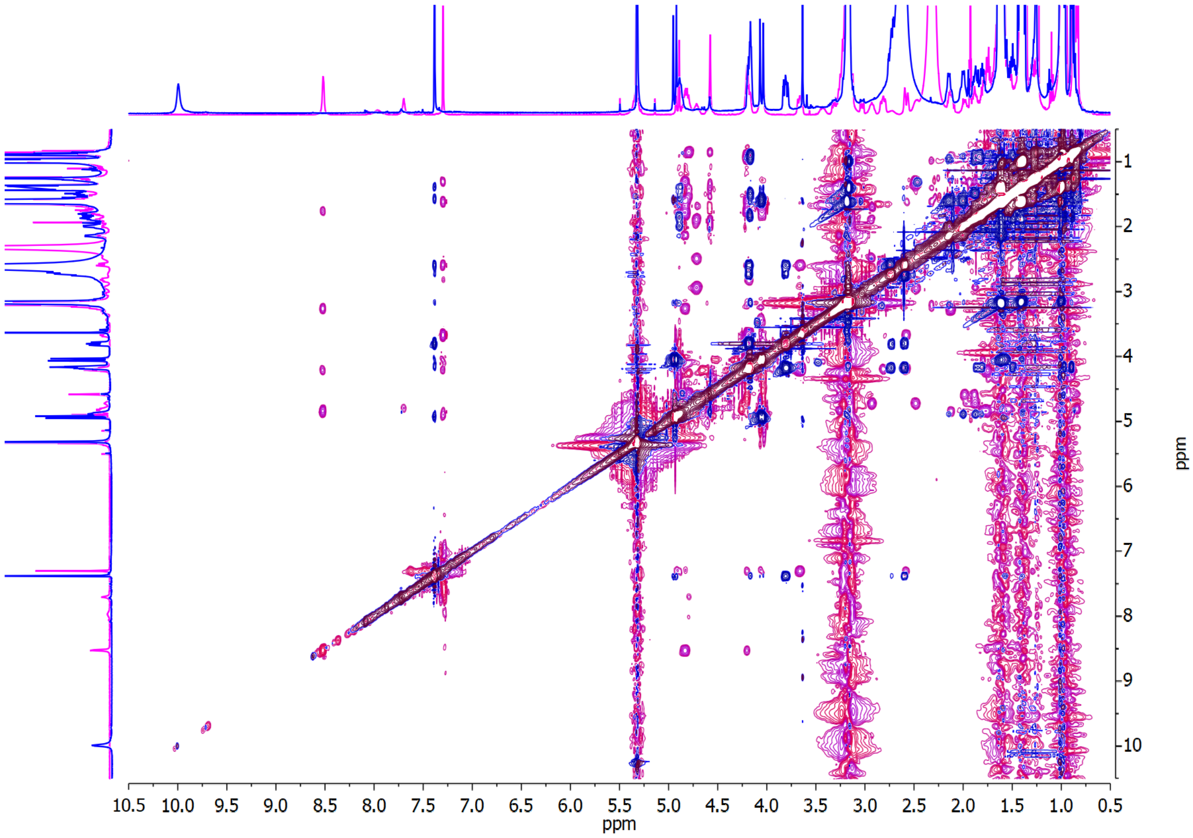

Supplementary Fig. 41. Superimposed ROESY spectra of the complexes $3Cl^{-}\cdot nH_2O\subset 2CP2$ (magenta) and $3F^{-}\cdot nH_2O\subset 2CP2$ (blue). Note that in the spectrum of the chloride complex also contains some signal corresponding to the remaining free **D2**. Both experiments were run employing in CD_3CN/CD_2Cl_2 (10%) as solvent.

$3Cl^{-}\cdot nH_2O\subset 2CP2$/$mAcO^{-}\cdot nH_2O\subset 2CP2$

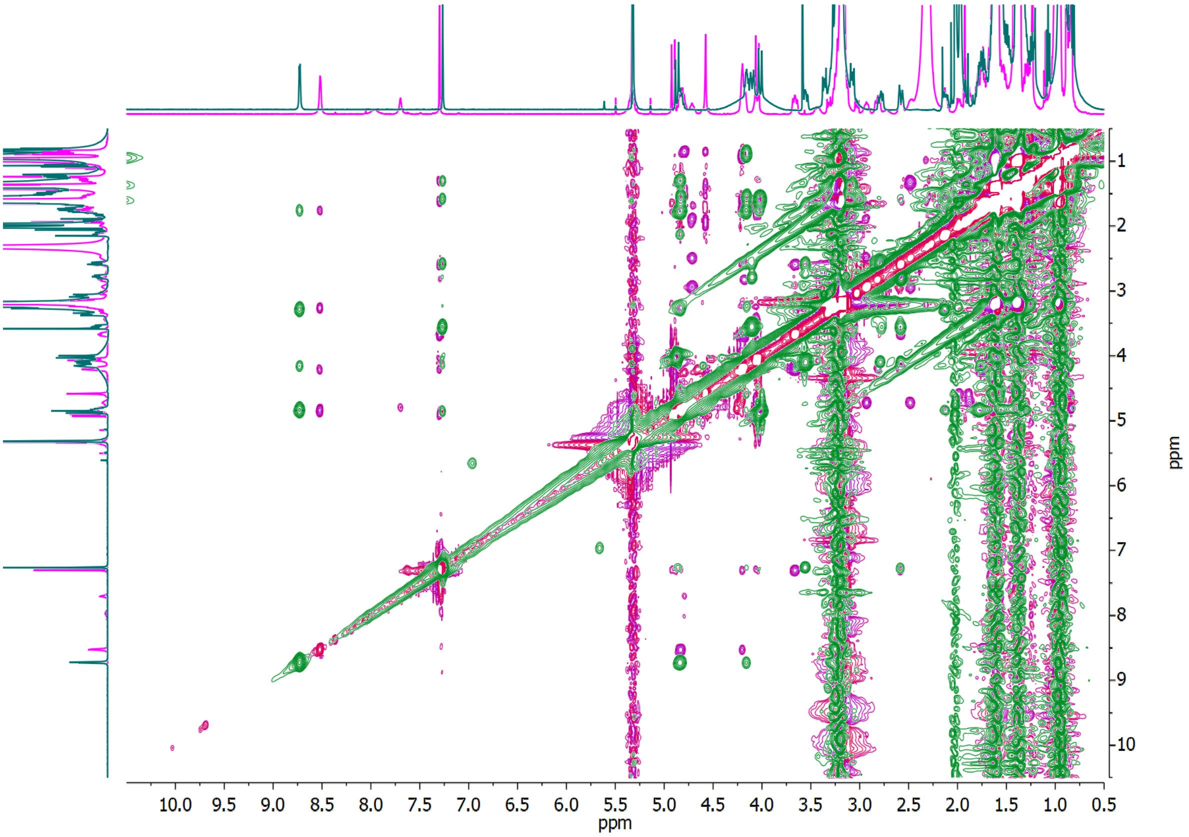

Supplementary Fig. 42. **Superimposed ROESY spectra of the complexes $3Cl^{-}\cdot nH_2O\subset 2CP2$ (magenta) and $mAcO^{-}\cdot nH_2O\subset 2CP2$ (green).** Note that in the spectrum of the chloride complex also contains some signal corresponding to the remaining free **D2**. Both experiments were run employing in CD_3CN/CD_2Cl_2 (10%) as solvent.

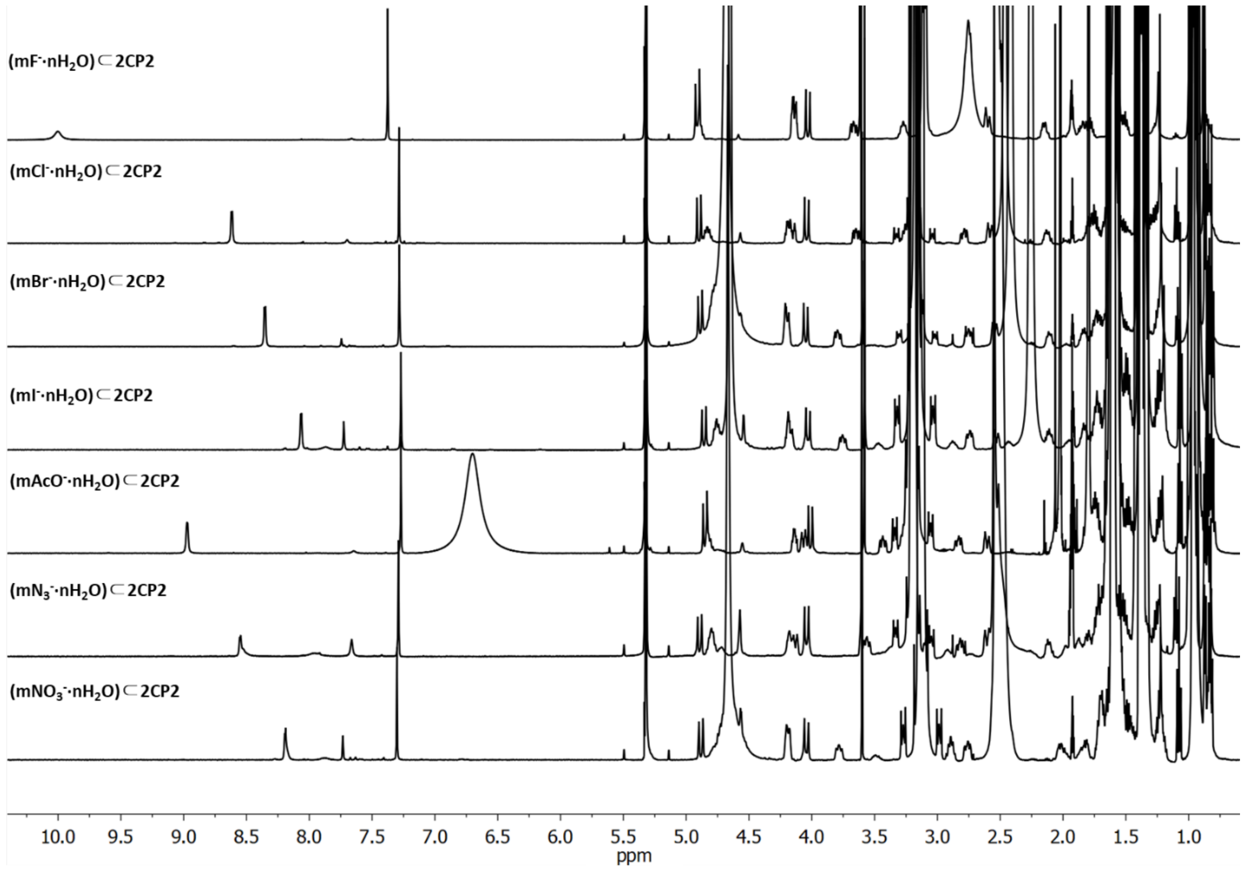

Supplementary Fig. 43. **Stacked ^{1}H NMR spectra of the complexes between CP2 and different anions.** All the experiments were run employing in CD_3CN/CD_2Cl_2 (10%) as solvent.

5. SUPPLEMENTARY DISCUSSION III: DFT CALCULATIONS.

In order to further investigate the supramolecular dimers exposed in this work, we carried out some DFT geometry optimizations of those structures employing B3LYP as functional and 6-31G(d,p) as basis set. We also included GD3BJ as dispersion correction.[5] This level of calculation has been employed previously for supramolecular assemblies where the hydrogen bonds play a key role, such as Rebek's capsules.[6] Moreover, by using this method, we observed a high reproducibility of the geometries compared with the crystalline structures provided in this study.

5.1 NITRILES ENCAPASULATED INSIDE D2 CAVITY.

The geometry optimization of **MN⊂D2** and **SN⊂D2**, showed in Supplementary Fig. 8, showed that both bisnitriles fit inside **D2** cavity. In order to compare those structures with the one of **ACN⊂D2**, we also optimized the last one, following the same method (Supplementary Fig. 44). The most significant distances, $d_{O\cdots H}$ and $d_{N\cdots Htriazole}$, are specified in the Supplementary Table. 1.

A B

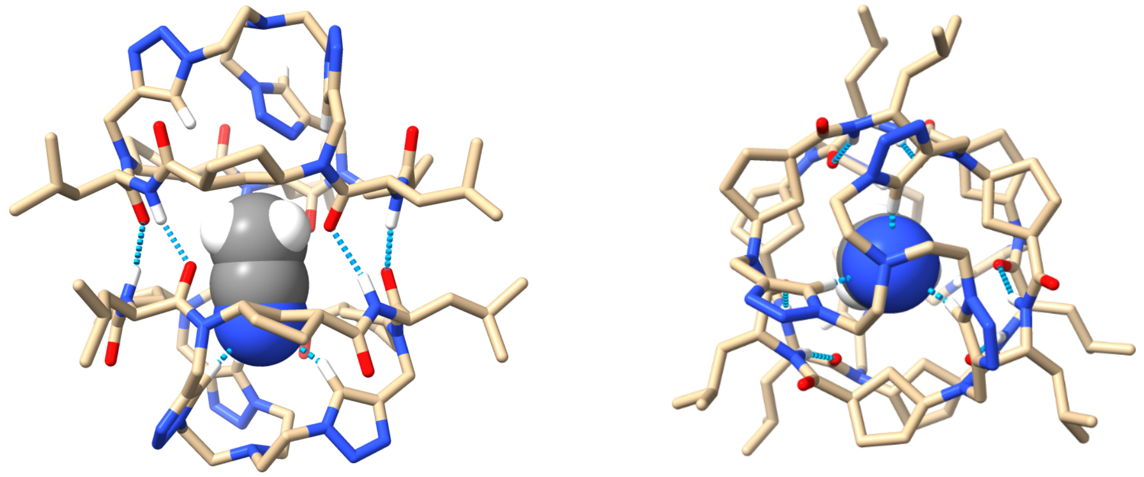

Supplementary Fig. 44. Front (A) and top (B) view of the ACN⊂D2 DFT geometry optimization structure. The nitrogen atoms are depicted in blue, oxygens in red and hydrogens in white, as well as carbons in beige (except for the encapsulated acetonitrile molecule, where the carbons are depicted in grey). The level of calculation employed was B3LYP/6-31G(d,p), including GD3BJ as dispersion.

	$d_{O\cdots H}$	$d_{N\cdots Htriazole}$	δ_{NH}	$\delta_{Htriazole}$
**ACN⊂D2**	1.94 (2.10)	2.46 (2.74)	8.02	7.71
**MN⊂D2**	1.85	2.65	8.01	7.77
**SN⊂D2**	1.98	2.36	7.85	7.62

Supplementary Table 1. Relevant parameters regarding the structures of the different nitriles encapsulated inside D2. The $d_{O\cdots H}$ and $d_{N\cdots Htriazole}$ values are the ones obtained from the optimized geometries of **ACN⊂D2**, **MN⊂D2** and **SN⊂D2**. In parentheses, the experimental values for the specie **ACN⊂D2** are indicated. The distances are given in angstrom and the chemical shift in ppm.

The obtained hydrogen bond distance for the species **ACN⊂D2** (1.94 Å) is shorter than the experimental one (2.10 Å), and also the $d_{N\cdots Htriazole}$. Remarkably, the O···H distance for **MN⊂D2** (1.85 Å) is shorter than the one found for **ACN⊂D2**, despite the bigger size of the guest. Experimentally, we did not appreciate a stronger hydrogen bond for **MN⊂D2**. However, the reduction in the N···$H_{triazole}$ distance for **MN⊂D2** does match with the experimental down-field shift suffered by this proton (7.77 ppm), compared with **ACN⊂D2** (7.71 ppm). Moreover, for the biggest guest, succinonitrile, we observed the up-field shift of the amide proton (from 8.02 to 7.75 ppm) and we attributed that to a weaker hydrogen bond interaction. The optimized structure of **SN⊂D2** reported a bigger O···H distance (1.98 Å) than those found for the other two nitriles. In contrast, the N···$H_{triazole}$ is the shorter one (2.36 Å), which disagree with the up-field shift suffered by the triazole proton. We propose that, maybe, the contraction of the **MN⊂ D2** structure showed by the computational structure could be due to the overestimation of the six N···$H_{triazole}$ contacts compared with the three that are possible in the **ACN⊂D2** structure. Moreover, in this calculation we did not consider the solvent molecules that surround the complexed. Then, for the

experimental up-field shift found for **SN⊂D2**, we propose that the triazole moieties could turn their proton outside making room for the biggest guest. The interaction of those protons with other succinonitrile molecules present in the solution, could stabilize that conformation. Moreover, we have to consider that the chemical shift that we observe could be an average of the triazole ring being exposed to slightly different chemical environments.

5.2 HOMODIMERIC AND HETERODIMERIC STRUCTURES.

As is has been proved experimentally, the heterodimers **D2-3** and **D2-4** are more stable that the corresponding homodimers **D2**, **D3** and **D4**. The addition of **D3** or **D4** over a **D2** solution shifted the equilibria in benefit of the formation of the mentioned heterodimeric species, as it is shown in the Supplementary Fig. 24 and Supplementary Fig. 27. Moreover, both **D2-3** and **D2-4** structures are illustrated in the Supplementary Fig. 29, where it is possible to appreciate that one of the triazole ring of the **CP2** moiety is turned outside, as in the crystal structure of **D2**. In order to compare between dimers and heterodimers, we also optimized **D2**, **D3** and **D4** (Supplementary Fig. 45), removing the acetonitrile and dioxane molecules found inside their cavities in their crystalline structures. In the Supplementary Table 2 we have collected some relevant geometry parameters and the experimental δ_{NH}. According to the O···H distances, **D2-3** show a shorter value (1.89 Å) than **D2** (1.94 Å) but not than **D3** (1.84 Å). In the case of **D2-4**, this distance (1.93 Å) is shorter than **D2** and **D4** (1.98 Å). Related to that, we realized that the δ_{NH} of **CP2** half of both heterodimers (8.21 ppm in **D2-3** and 8.02 in **D2-4**) higher than the one found for **D2** at the beginning of the experiment (8.00 ppm). The opposite situation was found for the Ach-based peptides **D3** and **D4**. The chemical shifts of the amide protons of **CP3** forming **D2-3** and also of the ones of **CP4** forming **D2-4** are both shielded compared to **D3** and **D4**. This could mean that the Acp-based cyclic peptides would be the more favored in the formation of the heterodimeric structures, and not the Ach-based ones. Further studies regarding the interaction and complexation energies of both dimers and heterodimers should be done to deeper investigate that process. However, that is out of the scope of this work. Moreover, regarding the angles C=O···H, the ones found for **D2-3** (142.3°) and **D2-4** (141.2°) are smaller than those of the homodimers **D2** (135.4°), **D3** (138.7°) and **D4** (138.3°). The optimal C=O···H alignment is supposed to be 120° if we assume sp^2 hybridization of the carbonylic oxygen. This would mean that the homodimers are better aligned than the heterodimers. However, it seems like the presence of the molecular cap force the carbolic group pointing inward the cavity, and that is what decrease the C=O··H angle in **D2** and **D4**, while **D3** present similar value but due to the rotation between both **CP3** subunits. For the N-H···O angle, the closest it is to 180°, the best is the alignment. For **D2-3**, this angle (163.3°) is bigger than the ones of **D2** (162.2°) but smaller than the one of **D3** (165.8°). However, for **D2-4**, the N-H···O angle (160.9°) is smaller than those of D2 and D4 (161.6°).

D2

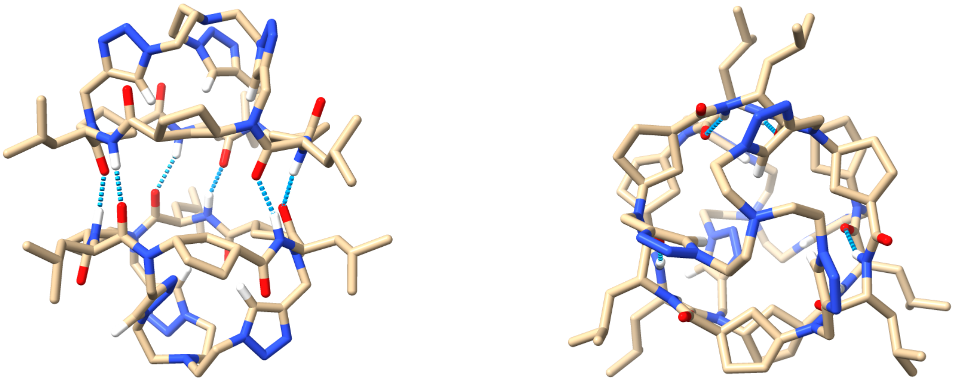

D3

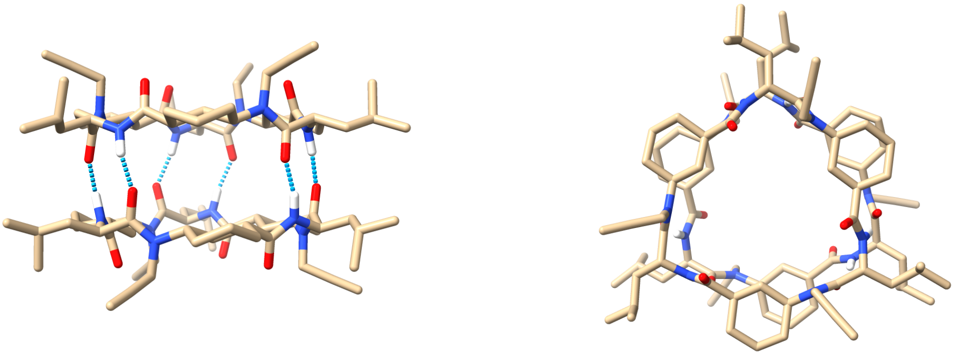

D4

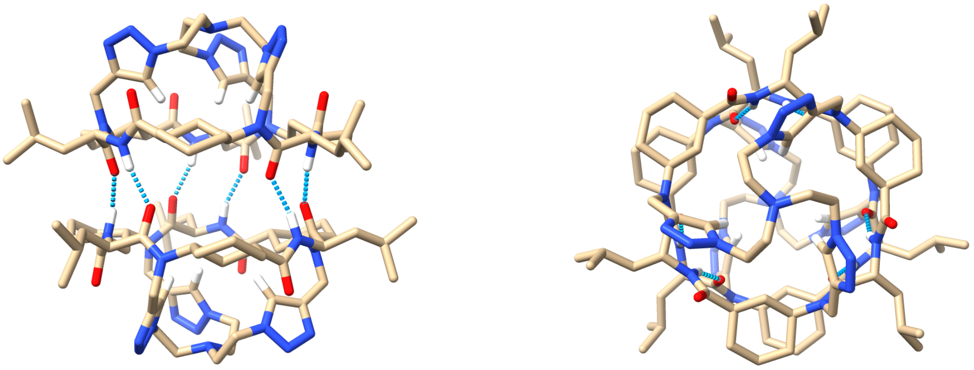

Supplementary Fig. 45. DFT geometry optimization of D2, D3 and D4. The nitrogen atoms are depicted in blue, oxygens in red and hydrogens in white, as well as carbons in beige. The level of calculation employed was B3LYP/6-31G(d,p), including GD3BJ as dispersion.

	$d_{O\cdots H}$	C=O···H	N-H···O	δ_{NH}
**D2**	1.94	135.4	162.2	8.00
**D3**	1.84	138.7	165.8	8.20
**D4**	1.98	138.3	161.6	7.74
**D2-3**	1.89	142.3	163.3	8.21 (**CP2**)/7.64 (**CP3**)
**D2-4**	1.93	141.2	160.9	8.02 (**CP2**)/7.91 (**CP4**)

Supplementary Table 2. Relevant parameters regarding the structures of the homodimers D2, D3 and D4, compared with the heterodimers D2-3 and D2-4. The distances are given in angstrom, the angles in degrees and the chemical shift in ppm.

6. SUPPLEMENTARY DISCUSSION IV: VESICLES PREPARATION AND TRANSPORT MEASUREMENTS

The general procedures for vesicle preparation and transport measurements are already reported in the *Methods* section of the manuscript. Here, we describe specific changes on these assays.

6.1 LUCIGENIN ASSAY

The N, N'-dimethyl-9,9'-biacridinium dinitrate, commonly known as lucigenin, is the normally used fluorophore to study chloride transport through lipid membranes.[7,8] The lucigenin emission depends on the counterion coupled to its aromatic structure. Thus, when halide anions (excluding fluoride) are internalized, the emission of the dye is quenched. To induce this transport, a pulse of a sodium salt (chloride, bromide, and iodide) was used.

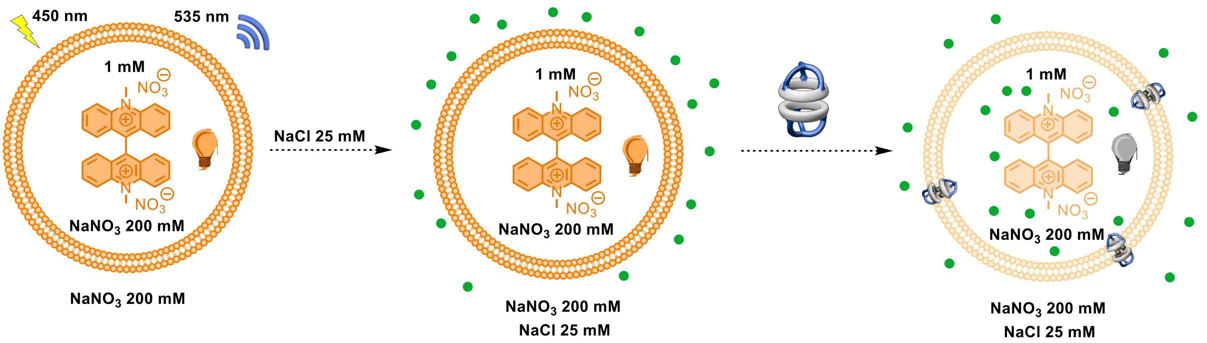

Supplementary Fig. 46. Schematic representation of the lucigenin assay. The dye emission was quenched after the addition of **D2**, due to the chloride transport that it promoted across the lipid membrane. The green dots represent chloride anions.

The liposomes were prepared following the procedure described in the methods section, at the main manuscript. The intravesicular media contains lucigenin (1 mM) in an aqueous solution of $NaNO_3$ (200 mM). The vesicles were suspended in an aqueous solution of $NaNO_3$ (200 mM) that was used as extravesicular media.

Measurement protocol: Vesicle suspension (50 µL) was added into a plastic cuvette containing of $NaNO_3$ (1950 mL, 200 mM). After 60 s, NaCl is added (25 µL, 2 M) and then elapsed 60 more seconds until transporter aliquots (**CP2** or **CP4**, the given concentrations are referred to CP, not to dimers) under study (*i*-PrOH solutions) was added and the change in lucigenin fluorescence was measured (λexc = 450 nm y λem = 535 nm). After 27 min, Triton-X 100 was added (50 µL, 10% v/v solution in water) to lyse all the vesicles and normalize the fluorescence trace. Experiment ended after 30 min.

6.1.1 LUCIGENIN ASSAY - CATION VARIATION

The preparation of vesicles for this assay is the same that for the normal lucigenin assay. The intra- and extravesicular media were kept also constant. In this assay, we kept constant the CP concentration (75 µM) between measurements. The variations depend on the chloride salt added. Lucigenin quenching was measured through the addition of chloride salts (LiCl, NaCl, KCl and CsCl) in separated experiments. For each chloride salt, the measurements were repeated at least three times. All the concentrations of the stock solutions of the chloride salts (2 M) used these experiments were identical.

6.1.2 LUCIGENIN ASSAY - ANION VARIATION

The preparation of vesicle for this assay was similar to the previously described lucigenin assay. The intra- and extravesicular media was kept also constant. In this assay, the peptide concentration ([CP] = 75 µM) was kept constant along measurements. The main difference was on the additions of sodium salts. Lucigenin quenching was measured through the addition of the salts (NaCl, NaBr and NaI) in separated experiments. All the measurements were repeated at least three times. All the concentrations of the stock solutions of the chloride salts (2 M) used these experiments were identical.

6.1.3 LUCIGENIN ASSAY - SULFATE VARIATION

In this case, the protocol for the preparation of vesicle was similar to the previously described lucigenin assay but substituting the nitrate for sulfate in both extra- and intravesicular media (200 mM Na_2SO_4). The peptide concentration ([CP] = 50 µM) was kept constant along measurements. The rest of parameters were kept constant with respect to the procedure described in 6.1. The measurements were recorded three times, and the average was taken as result and it was compared to experiments carried out with the same peptide concentration but using $NaNO_3$.

6.1.4 LUCIGENIN ASSAY – DATA TREATMENT

The data was normalized using the following equation:

$$F_n = \frac{F_t - F_\infty}{F_0 - F_\infty} \quad \mathbf{(1)}$$

Where F_n is the normalized fluorescence, F_t is the fluorescence at certain time, F_∞ is the fluorescence intensity after the total lysis of the liposomes (i.e., 30 min) and F_0 is the fluorescence value by the time of the addition of the compound under study (i.e., 127 s).

The resulting data was adjusted to the Hill equation:

$$Y = \frac{Y_0 + (Y_{max} - Y_0)}{\left[1 + \left(\frac{EC_{50}}{c}\right)^n\right]} \quad \mathbf{(2)}$$

Where Y is the transport % at 26 min of the experiment (Y_{26}), Y_0 is the minimum transport, promoted by the smallest peptide concentration, EC_{50} correspond to the concentration which promote the 50% of the maximum activity (Y_{max}). The Hill coefficient (n) is related with the stoichiometry or the cooperativity of the transport. Finally, c is the concentration of the transported for each Y_{26}.

6.2 HPTS ASSAY

The 8-hydroxypyrene-1,3,6-trisulfonic acid trisodium salt (HPTS), commonly known as pyranine, is a fluorophore widely used for testing the H^+/OH^- transport ability of an ionophore through the monitoring of internal pH of liposomes. The emission of the HPTS is dependent on the pH, increasing its signal with the increase of pH and vice versa, therefore it is used to follow changes in the internal pH of liposomes.[9] Once the chloride transport ability of **D2** on lucigenin assays was proven, it was decided to evaluate if the chloride transport was couple to a change in the pH of intravesicular medium using HPTS assay. During the experiment, a base pulse (NaOH) was added, inducing a pH gradient between the intra- and the extravesicular media. If an enhancement on the emission is observed, signal of vesicle basification, that result could be related with an antiporter transport of OH^- or to symporter transport of H^+.

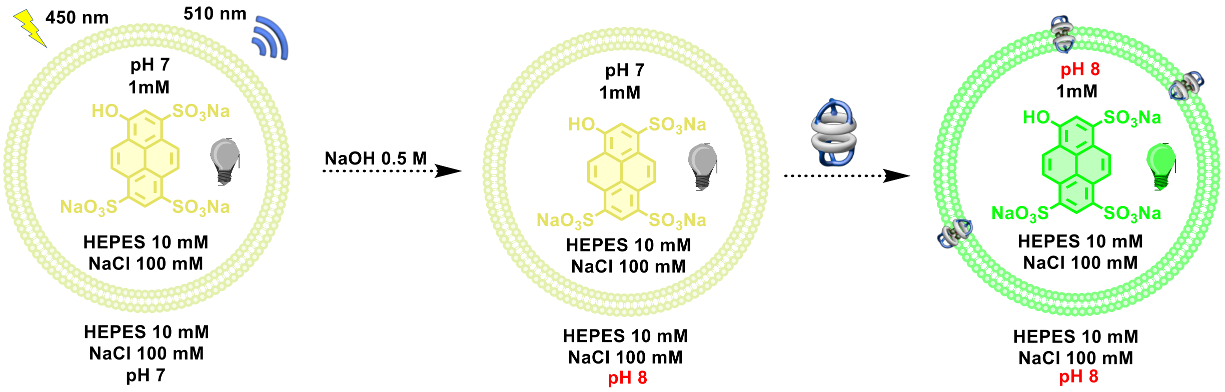

Supplementary Fig. 47. **Schematic representation of the HPTS assay**. The dye emission was switched off after the addition of **D2**, due to, either H^+ symport or OH^- antiport, that it promoted across the lipid membrane.

For the preparation of vesicles for this assay, the general procedure described in the methods section, at the main manuscript, was followed. The intravesicular buffer contains HPTS (1 mM) in an aqueous solution of HEPES (10 mM, pH 7.0) containing NaCl (100 mM). The same buffer solution conditions, i.e., HEPES (10 mM, pH 7), NaCl (100 mM), was used as extravesicular media, but adjusted to pH 7 with aqueous solutions of HCl or NaOH.

Measurement protocol: 50 µL of the vesicle suspension were dispersed into a cuvette containing 1980 µL of the buffer, composed by HEPES (10 mM) and NaCl (100 mM) at pH 7.0. After 60 s, NaOH (25 µL, 0.5 M) was added and then elapsed 60 more seconds until transporter aliquots (DMSO solutions) were added and the change in HPTS fluorescence (basification) was measured (λ_{exc} = 450 nm and λ_{em} = 510 nm). After 27 min, Triton-X 100 was added (50 µL, 10% solution in water) to lyse all the vesicles and normalize the fluorescence traces. Experiment ended after 30 minutes.

6.2.1 HPTS ASSAY in the presence of FCCP

The preparation of the samples for this assay was similar to the previously described HPTS assay (5.2). However, before the NaOH and CP additions, the FCCP (25 µL, 132 µM in DMSO, 1% mol with respect to the lipid concentration) proton transporter was added one minute after experiment started, then NaOH (25 µL, 0.5 M) was added after two minutes and later CP in DMSO after three minutes. Finally, Triton X-100 (50 µL, 10% solution in water) was added after 27 min.

FCCP is an efficient proton transporter, therefore its presence allows to evaluate if proton transport is rate-limiting step. If the transporter activity clearly increases with the addition of FCCP it would mean that the transport mechanism is limited by the H^+ migration and, therefore, this would be the rate-limiting step.

6.2.2 HPTS ASSAY-COMPETITION VARIATION

The preparation of the samples for this assay was the same that the previously described HPTS assay (6.2). The buffer composed by HEPES (10 mM) and NaCl (100 mM NaCl) at pH 7.0 was used to elute the vesicles on the size exclusion chromatography. This vesicle suspension (50 µL) was dispersed on the extravesicular media (1950 µL of the mentioned buffer at pH 7.0) containing different sodium salts (100 mM). In this case, no NaOH pulse was required. In this experiment, addition of a DMSO solution of CP (25 µL) was carried out after 2 min and the measurements were performed for at least 10 min.

The signal input is generated by transport rates of Cl^- compared with the tested anion. Those anions that are transported more efficiently than chloride give rise to acidification of the intravesicular medium, that is, a decrease in the emission of HPTS. On the contrary, the anions that are transported with rates slower than chloride, give rise to the basification of the intravesicular medium and, therefore, an increase in emission is observed.

6.2.3 HPTS ASSAY – DATA TREATMENT

The data was normalized using the following equation:

$$F_n = \frac{F_t - F_0}{F_\infty - F_0} \quad \mathbf{(3)}$$

Where F_n is the normalized fluorescence, F_t is the fluorescence at certain time, F_∞ is the fluorescence intensity after the total lysis of the liposomes (i.e., 30 min) and F_0 is the fluorescence value by the time of the addition of the compound under study (i.e., 127 s). In the experiments with FCCP or valinomycin, F_0 is the fluorescence value at 187 s, as the peptide was added 1 minute later than in other experiments.

The obtained data were adjusted to the Hill equation:

$$Y = \frac{Y_0 + (Y_{max} - Y_0)}{\left[1 + \left(\frac{EC_{50}}{c}\right)^n\right]} \quad \mathbf{(2)}$$

Where Y is the percentage of transport calculated after 26 min of experiment (Y_{26}), Y_0 is the transport promoted by the lowest concentration of peptide, EC_{50} corresponds to the calculated concentration that promotes 50% of the maximum activity (Y_{max}). The Hill coefficient (n) is related with the stoichiometry or the cooperativity of the transport. Finally, c is the concentration of the transporter for each Y_{26}.

7. SYNTHESIS AND CHARACTERIZATION

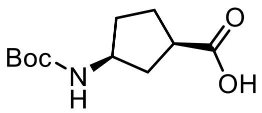

Boc-*L*-γ-Acp-OH (1a). A solution of **Boc-*L*-γ-Ace-OH** (2.8 g, 12 mmol) in EtOH (60 mL) was treated with Pd/C (10% mol, 1.2 mmol, 1.3 g) and stirred overnight under hydrogen atmosphere (ballon pressure). After that, the mixture was filtered over celite and the mother liquors were concentrated under reduced pressure, affording the compound **1a** as a white solid, that was used in the following step without further purification [2.77 g, 98%]. **^{1}H-NMR** ($CDCl_3$, 250.13 MHz) δ (ppm): 6.34 and 5.03 (m, 1H), 4.15-3.73 (1H), 2.81 (m, 1H), 2.18 (m, 1H), 1.39 (s, 9H). **^{13}C-NMR** ($CDCl_3$, 62.90 MHz) δ (ppm): 181.7 (CO_2H), 155.4 (CO), 79.2 (C), 51.9 (CH), 41.7 (CH), 36.0 (CH_2), 33.0 (CH_2), 28.3 (CH_3), 27.9 (CH_2). **MS (ESI)** [m/z (%)]: 230 ([MH]$^+$, 42), 174 (89), 156 (66), 130 ([MH-Boc]$^+$, 100), 112 (74), 95 (10), 84 (11). **HRMS [MH]$^+$** calculated for $C_{11}H_{20}NO_4$: 230.139233, found: 230.140269.[10,11]

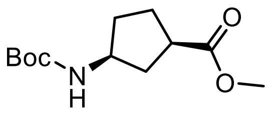

Boc-*N*-*L*-γ-Acp-OMe (1b). The compound **1a** (2.7 g, 11.8 mmol) was dissolved in dry DMF (50 mL) and treated with Na_2CO_3 (2.5 g, 23.5 mmol). Subsequently, MeI (3 mL, 47.2) was added dropwise. The resulting solution stirred for 3 h under an argon atmosphere. After that time, AcOEt (30 mL) and H_2O (30 mL) were added. The organic layer was washed with H_2O (2x25 mL) $NaHCO_3$ (sat, 3x30 mL), and NaCl (sat, 3x30). Then, it was dried over anhydrous $MgSO_4$, filtered and concentrated to afford the compound **1b** as a pale-yellow oil [2.8 g, 98%], that was used in the following step without further purification. **^{1}H-NMR** ($CDCl_3$, 300 MHz) δ (ppm): 4.93 and 4.45 (br, 1H), 4.04 (br, 1H), 3.67 (2s, 3H), 2.85-2.79 (br, 1H), 2.24-1.49 (m, 6H), 1.43 (s, 9H). **^{13}C-NMR** ($CDCl_3$, 75.4 MHz) δ (ppm): 177.2 (CO), 155.3 (CO), 78.9 (C), 51.9 (CH_3), 51.8 (CH), 41.6 (CH), 36.4 (CH_2), 32.0 (CH_2), 28.3 (CH_3), 27.8 (CH_2). **MS (ESI)** [m/z (%)]: 266 ([M+Na]$^+$, 100), 267 ([M+Na]$^+$, 25), 228 ([M-Me]$^+$, 1). **HRMS (ESI)** Calculated for $C_{12}H_{21}NO_4$: 243.1471, found**:** 243.1473.[12]

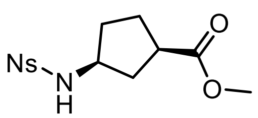

Ns-*N*-*L*-γ-Acp-OMe (1c). A solution of compound **1b** (2.8 g, 11.5 mmol) in CH_2Cl_2 (46 mL) was treated with TFA (12 mL). After stirring for 15 minutes, the solution was concentrated in a rotary evaporator and then under high vacuum for 4 h. The resulting solid was redissolved in dry CH_2Cl_2 (38 mL) and DIEA (7.7 mL, 46.3 mmol) was added, followed by 2-nitrobenzenesulfonyl chloride (2.76 g, 12.7 mmol). The mixture stirred for 24 h under an argon atmosphere. Then, it was washed with HCl aq. (5%, 2x50 mL) and $NaHCO_3$ (sat., 2x50 mL), dried over anhydrous $MgSO_4$, filtered and concentrated in vacuo. After purification by column chromatography (15-50% AcOEt/Hexane), the compound **1c** was obtained as a yellow solid. [3.58 g, 94%]. **^{1}H-NMR** ($CDCl_3$, 300 MHz) δ (ppm): 8.29 (d, *J* = 8.8 Hz, 2H), 8.02 (d, *J* = 8.8 Hz, 2H), 5.84 and 5.33 (2d, *J* = 6.9 and 7.2 Hz, 1H), 3.61 and 3.57 (2s, 3H), 3.78 (br, 1H, Hγ), 2.76 (m, 1H). **^{13}C-RMN** ($CDCl_3$, 75 MHz) δ (ppm): 176.6 (C=O), 150.0 (Ar), 133.7 (Ar), 133.4 (Ar), 132.8 (Ar), 130.7 (Ar), 125.3 (Ar), 55.5 (CH_3), 52.8 (CH), 41.6 (CH), 36.4 (CH_2), 33.2 (CH_2), 27.9 (CH_2). **MS (ESI)** [m/z (%)]: 328 ([M+H]$^+$, 100). **HRMS (ESI)** Calculated for $C_{13}H_{16}N_2NaO_6S$: 351.0621, found: 351.0608.[12]

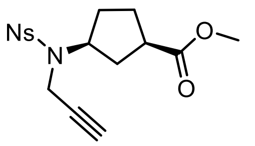

Ns-^{propargyl}*N*-*L*-γ-Acp-OMe (1d). The compound **1c**, (2.5 g, 7.6 mmol) was dissolved in dry DMF (38 mL) and treated with K_2CO_3 (4.2 g, 30.0 mmol) and propargyl bromide (80%, 2.68 mL, 30.0 mmol). The mixture was stirred overnight under Ar atmosphere and then DMF was mostly removed by consecutives extractions with H_2O (4x30 mL) using CH_2Cl_2 as organic phase. The organic layer was washed with aqueous HCl (5%, 2x50 mL) and saturated aqueous solutions of $NaHCO_3$ (3x50 mL). The organic layer was dried over anhydrous $MgSO_4$, filtered, concentrated. The remaining DMF was removed by consecutives toluene addition followed by rotavapor concentrations. The product was purified by flash chromatography (10-20% AcOEt/hexane) providing the desired product **1d** as a light-yellow oil [2.7 g, 97%].**^{1}H-NMR** ($CDCl_3$, 300 MHz) δ (ppm): 8.18-8.06 (m, 1H), 7.76-7.53 (m, 3H), 4.41-4.19 (m, 1H), 4.17 (2d 2H), 3.74-3.56 (s, 3H), 2.95-2.64 (m, 1H), 2.24-1.79 (m, 7H). **^{13}C-NMR** ($CDCl_3$, 75 MHz) δ (ppm): 176.0

(C=O), 148.0 (Q), 133.8 (CH), 133.5 (Q), 131.8 (CH), 131.2 (CH), 124.2 (CH), 79.4 (Q), 73.1 (CH), 58.8 (CH), 58.3 (CH_3),51.9 (CH), 40.7 (CH), 32.9 (CH_2), 32.6 (CH_2), 28.5 (CH_2), 26.9 (CH_2). **MS (ESI)** [m/z (%)]: 240 ($[MH]^+$-Ns-^{propargyl}*N*, 60), 367 ($[MH]^+$, 35). **HRMS (ESI)** Calculated for $C_{16}H_{18}N_2NaO_6S$: 389.0778, found: 389.0776.[12]

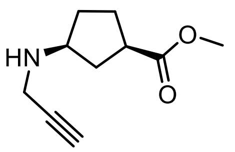

***^{propargyl}N-L*-γ-Acp-OMe (1e).** A solution of the compound **1d** (3.2 g, 8.74 mmol) in MeCN (70 mL) was treated with K_2CO_3 (6.0 g, 43.7 mmol) and PhSH (3.5 mL, 35.0 mmol). The mixture stirred overnight and then acetonitrile was evaporated under vacuum. The resulting residue was dissolved in CH_2Cl_2 and washed with H_2O (2x25 mL) and brine (2x25 mL). The organic layer was dried over anhydrous $MgSO_4$, filtered and concentrated under reduced pressure. The resulting residue was purified by flash chromatography (0-8% MeOH/CH_2Cl_2) to afford the desired product **1e** as colorless oil [1.12 g, 71%]. **^{1}H-NMR** ($CDCl_3$, 300 MHz) δ (ppm): 4.14-3.86 (m, 1H), 3.56 (s, 3H), 3.43- 3.13 (m, 3H), 2.69 (m, 1H), 2.21-1.13 (m, 7H). **^{13}C-NMR** ($CDCl_3$, 75 MHz) δ (ppm): 176.3 (C=O), 82.2 (Q), 71.1 (CH), 57.5 (CH_3), 51.6 (CH), 42.1 (CH), 36.5 (CH_2), 36.2 (CH_2), 32.1 (CH_2), 27.4 (CH_2). **MS (ESI)** [m/z (%)]: 182 ([MH]+, 100). **HRMS (ESI)** Calculated for $C_{10}H_{16}NO_2$: 182.1176, found: 182.1175.[12]

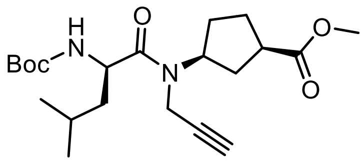

Boc-*D*-Leu-*^{propargyl}N-L*-γ-Acp-OMe (1f). A solution of compound **1e** (1.5 g, 8.3 mmol) in dry CH_2Cl_2 (40 mL) was treated with DIEA (4.3 mL, 24.9 mmol). Separately, the Boc-*D*-Leu-OH (512 mg, 2.1 mmol) was dissolved in dry CH_2Cl_2 (40 mL), and then, DIEA (4.3 mL, 24.9 mmol) and *N*-HATU were added (4.7 g, 12.5 mmol). After stirring under Ar for 4 hours, the resulting solution was washed with HCl 5% (2x40 mL) and sat. aq. $NaHCO_3$ (2x40 mL), dried with anhydrous $MgSO_4$, filtered and concentrated under vacuum. The crude mixture was purified by flash column chromatography (0-4% MeOH/CH_2Cl_2), to give the compound **1f** as light-yellow foam [2.65 g, 81%]. **^{1}H-NMR** ($CDCl_3$, 300 MHz) δ (ppm): 5.49 and 5.11 (2d, *J* = 8.92, 1H), 4.88-4.57 (m, 1H), 4.40 (m, 1H), 4.21-3.83 (m, 2H), 3.67 (d, *J* = 6.5 Hz, 3H), 3.06-2.71 (m, 1H), 2.43-1.07 (m, 19H), 1.05-0.81 (m, 6H). **^{13}C-NMR** ($CDCl_3$, 75 MHz) δ (ppm): 175.8 (C=O), 172.5 (C=O), 155.3 (C=O), 80.4 (Q), 72.7 (Q), 57.6 (CH_3), 51.7 (CH), 48.6 (CH), 42.0 (CH_2), 41.0 (CH), 32.6 (CH_2), 29.6 (CH_2) 28.2 (CH_3), 27.8 (CH_2), 27.4 (CH_2) 24.5 (CH_3), 23.4 (CH), 21.6 (CH). **HRMS (ESI)** Calculated for $C_{21}H_{34}N_2O_5Na$: 417.2360, found: 417.2362.[12]

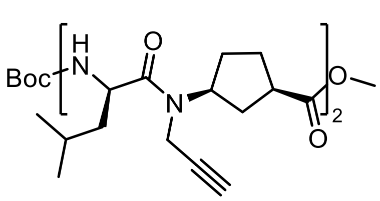

Boc-[(*D*-Leu-*^{propargyl}N-L*-γ-Acp)$_2$]-OMe (1g). A solution of compound **1f** (656 mg, 1.7 mmol) in MeOH/H_2O (Vt = 8.5 mL, 3:1) was treated with LiOH (204 mg, 8.5 mmol) and stirred for 4 h. After this time, MeOH was concentrated under reduced pressure and the resulting solution was acidified until pH 3 (HCl 5% aq.) and extracted with CH_2Cl_2 (4x15 mL). The combined organic layers were dried with anh. $MgSO_4$, filtered and concentrated under reduced pressure to give the *C*- deprotected dipeptide. Parallelly, a different fraction of compound **1f** (656 mg, 1.7 mmol) was dissolved in CH_2Cl_2 (4 mL) and treated with TFA (4 mL). After stirring for 30 min, the solvent was removed under reduced pressure and the resulting oil was redissolved in CH_2Cl_2 and further evaporated, repeating this process up to three times. The *C*-deprotected dipeptide was dissolved in dry CH_2Cl_2 (9 mL) and subsequently DIEA (1.3 mL, 5.1 mmol) and *N*-HBTU (966 mg, 2.6 mmol) were added. The *N*-deprotected dipeptide was dissolved in dry CH_2Cl_2 (9 mL) and DIEA was added (1.3 mL, 5.1 mmol). Finally, both fractions were mixed and reacted under Ar for 4 h. This solution was washed with HCl 5% (2x15 mL) and sat. aq. $NaHCO_3$ (2x15 mL), dried with anhydrous $MgSO_4$, filtered and concentrated under reduced pressure. The crude mixture was purified by flash column chromatography (25-50% AcOEt/hexane) to give the compound **1g** as a foam [804 mg, 72%]. **HRMS (ESI)** Calculated for $C_{36}H_{57}N_4O_7$: 657.4222 [$(MH)^+$]; found: 657.4222.[13]

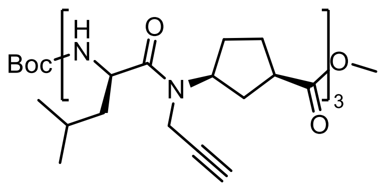

Boc-[*D*-Leu-^{propargil}*N*-*L*-γ-Acp]$_3$-OMe (1h). The compound **1g** (925 mg, 1.4 mmol) was dissolved in CH_2Cl_2 (2.5 mL) and treated with TFA (3.5 mL). After stirring for 30 min, the solvent was removed under reduced pressure and the resulting oil was redissolved in CH_2Cl_2 and further evaporated, repeating this process up to three times. Parallelly, the dipeptide **1f** (656 mg, 1.7 mmol) was dissolved in MeOH/H_2O (Vt = 8.5 mL, 3:1) was treated with LiOH (204 mg, 8.5 mmol) and stirred for 4 h. After this time, MeOH was concentrated under reduced pressure and the resulting solution was acidified until pH 3 (HCl 5% aq.) and extracted with CH_2Cl_2 (4x15 mL). The combined organic layers were dried with anhydrous $MgSO_4$, filtered, and concentrated under reduced pressure to give the *C*- deprotected dipeptide. The *C*- deprotected dipeptide was dissolved in dry CH_2Cl_2 (8 mL) and subsequently DIEA (1.3 mL, 5.1 mmol) and *N*-HBTU (966 mg, 2.6 mmol) were added. The *N*-deprotected tetrapeptide dissolved in dry CH_2Cl_2 (8 mL) and DIEA was added (1.3 mL, 5.1 mmol). Finally, both fractions were mixed and reacted under Ar for 4 h. This solution was washed with HCl 5% (2x15 mL) and sat. aq. $NaHCO_3$ (2x15 mL), dried with anhydrous $MgSO_4$, filtered, and concentrated under reduced pressure. The crude mixture was purified by flash column chromatography (25-70% AcOEt/hexane) to give the title compound as a white foam. [963 mg, 75%]. **HRMS (ESI)** Calculated for $C_{51}H_{79}N_6O_9$: 919.5903 [(MH)+]; found: 919.5904.[13]

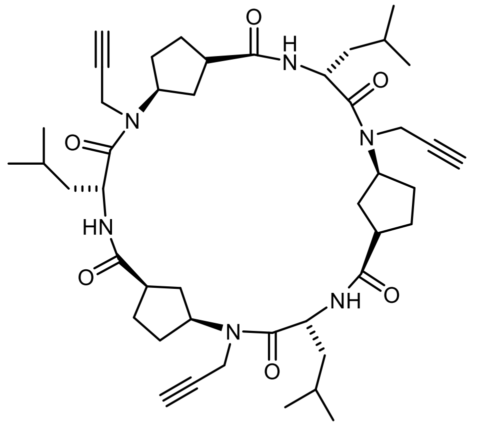

c-[(*D*-Leu-^{propargyl}*N*-*L*-γ-Acp)$_3$] (CP1). The hexapeptide **1h** (1.1 g, 1,2 mmol) was dissolved in MeOH (6 mL) and water (2 mL) and then LiOH (143 mg, 6.0 mmol) was added. After stirring for 4h, the organic solvent was evaporated, and the remaining aqueous layer was acidified until pH 3 with HCl. The resulting white precipitate was extracted with CH_2Cl_2 (4x5 mL), dried, filtered and concentrated. Free acid was further deprotected by a solution of TFA/CH_2Cl_2 (1:1, Vt = 6 mL), in which the product was stirred for 15 min. After completion of the reaction, TFA was removed under reduced pressure and the resulting oil was redissolved in CH_2Cl_2 and further evaporated, repeating this process up to three time. For the cyclization step, the oil obtained in the previous step was dissolved in dry CH_2Cl_2 (800 mL) and treated with DIEA (1 mL, 3.6 mmol) and *N*-TBTU (1.14 g, 3.6 mmol). The mixture was stirred overnight under Ar and then concentrated to reduce the volume in order to carry out the work up. Then, it was washed with 5% HCl (2x50 mL) and sat. aq. $NaHCO_3$ (2x50 mL). The organic layer was dried with $MgSO_4$, filtered and concentrated. The crude was purified by flash column chromatography CH_2Cl_2/MeOH 0-10% to give the desired product **CP1** as a white powder [421 mg, 45%]. **^{1}H-NMR** ($CDCl_3$, 300 MHz) δ (ppm): 8.11 (d, J = 9.5 Hz, 3H, NH), 5.10 (m, 3H, $H\alpha_{Leu}$), 4.80 (AB, J = 20.1 Hz, 3H, propargylic $CH_{2(1)}$), 4.66 (m, 3H, $H\gamma_{Acp}$), 3.82 (AB, J = 20.1 Hz, 3H, propargylic $CH_{2(2)}$), 2.86 (m, 3H, $H\alpha_{Acp}$), 2.27 (s, 3H, C≡CH), 2.21-1.28 (m, 27H, Acp + CH_2 Leu + CH Leu), 0.94-0.84 (dd, 18H, CH_3 Leu). **^{13}C-NMR** ($CDCl_3$, 75 MHz) δ (ppm): 175.7 (CO), 173.6 (CO), 80.2 (C), 72.4(CH), 54.9 (CH), 47.2 (CH), 41.4 (CH), 41.4 (CH_2), 35.9 (CH_2), 32.7 (CH_2), 27.7 (CH_2), 27.5 (CH_2), 24.6 (CH), 23.3 (CH_3), 22.0 (CH3). **FTIR** (CaF_2 pellet): ν = 3301 (amide A), 1665 (amide III),1620 (amide I), 1533 cm-1 (amide II). **HRMS (ESI)** Calculated for $C_{45}H_{67}N_6O_6$: 787.5117 [$(MH)^+$]; found: 787.5116.[13]

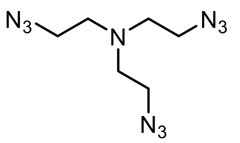

Tris(2-azidoethyl)amine. The tris(2-aminoethyl)amine (300 mg, 2.05 mmol) was dissolved in methanol. Then, K_2CO_3 (2.126 g, 15.4 mmol) and $CuSO_4{\cdot}5H_2O$ (16 mg, 0.06 mmol) were added. Finally, the imidazole-1-sulfonyl azide hydrochloride was added. The blue solution was stirred overnight, after which, the color turned pale green. The conversion was checked by mass spectrometry, where only the mass of the product was found. The methanol was removed under reduced pressure and the product was extracted with ethyl acetate and washed with H_2O (3x15 mL). The organic layer was dried with $MgSO_4$, filtered and concentrated. The product, a transparent and colorless oil, was used in the following step without further purification [276 mg, 60%] and its spectroscopic properties coincide with the previously reported.**^{1}H-NMR** (300 MHz, $CDCl_3$) δ (ppm): 3.36 (t, *J* = 6.1 Hz, 3H, CH_2-N), 2.80 (t, *J* = 6.2 Hz, N_3-CH_2). **MS (ESI)** [m/z (%)]: 225 (100) (M^+).[14]

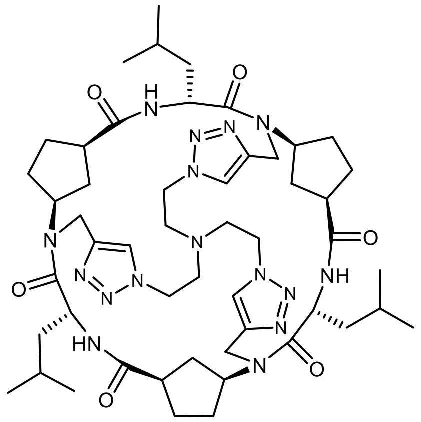

c-[(*D*-Leu-*^{triazolylamine}N*-*L*-γ-Acp-)$_3$] (CP2/D2). Cyclic peptide **CP1** (50 mg, 0.064 mmol), tris(2-azidoethyl)amine (21 mg, 0.095 mmol) and DBU (20 uL, 0.192 mmol), were dissolved in dry toluene (64 mL). Then, the solution was degassed with Ar flow for 40 minutes. After that, the mixture was heated to reflux and then CuBr (1.8 mg, 0.013 mmol) was added. The mixture was stirred at reflux under Ar overnight. After cooling, the mixture was filtered over filter paper. The product, D2, was insoluble in toluene. We used methanol to remove the product from the filter paper and then we concentrated it to dryness under reduced pressure. The residue was dissolved in CH_2Cl_2 (30 mL) and treated with an aqueous solution of KCN (10 mg/mL) (30 mL). The resulting two phases mixture was vigorously stirred for two hours. The organic layer was dried with $MgSO_4$, filtered and concentrated. The crude was purified by flash column chromatography (0-10% CH_2Cl_2-MeOH) and by normal-phase HPLC (2-10% MeOH-CH_2Cl_2 over 30 min) to give **D2** as a white solid (31 mg, 48 %, t_R = 18 min). **^{1}H-NMR** (500 MHz, CD_2Cl_2) δ (ppm): 8.04 (d, *J* = 9.2 Hz, 1H, NH), 7.71 (s, 1H, $H_{triazole}$), 4.86 (td, *J* = 9.3 and 4.3 Hz, 3H, $H\alpha_{Leu}$), 4.80 – 4.68 (m, 3H, $H\gamma_{Acp}$), 4.59 (s, 6H, $(CH_2)_{linker}$), 4.09 (dt, *J* = 14.0 and 4.0 Hz, 3H, H_A), 3.40 (ddd, *J* = 13.5, 9.1, 3.5 Hz, 3H, H_B), 3.25 (ddd, *J* = 15.0, 9.1 and 3.4 Hz, 3H, H_C), 2.96 (dt, *J* = 10.6 and 7.3 Hz, 3H, $H\alpha_{Acp}$), 2.85 (dt, *J* = 15.0 and 4.1 Hz, 3H, H_D) , 2.49 (dt, *J* = 12.6 and 6.7 Hz, 3H, *cis*-$H\beta_{Acp}$), 2.10 – 2.00 (m, 3H, *trans*-$H\varepsilon_{Acp}$), 1.95 (m, 3H, *cis*-$H\delta_{Acp}$), 1.90 (m, 3H, *cis*-$H\varepsilon_{Acp}$), 1.76 – 1.66 (m, 3H, *trans*-$H\delta_{Acp}$), 1.62-1.54 (m, 6H, CH_{Leu} + CH_{2Leu}), 1.44 (tt, *J* = 10.0 and 4.2 Hz, 3H, CH_{2Leu}), 1.34 (q, *J* = 11.6 Hz, 3H, *trans*-$H\beta_{Acp}$), 0.95 – 0.83 (m, 18H, CH_{3Leu}). **^{13}C-NMR** (126 MHz, CD_2Cl_2) δ (ppm): 175.3 (C), 174.9 (C), 145.2 (C), 124.4 (CH), 57.8 (CH_2), 57.0 (CH), 51.1 (CH_2), 49.3 (CH), 43.6 (CH_2), 43.0 (CH), 40.3 (CH_2), 38.1 (CH_2), 28.3 (CH_2), 28.6 (CH_2), 25.3 (CH), 23.7 (CH_3), 22.3 (CH_3). **FTIR (CaF2)**: ν = 3286 (amide A), 1623 (amide I) and 1532 cm-1 (amide II). **MS (ESI)** [m/z (%)]: 1033.6 (100) ($[M+Na]^+$), 2045.2 ($[2M+Na]^+$). **HRMS (ESI)** Calculated for $C_{51}H_{78}N_{16}NaO_6$: 1033.6182; found: 1033.61.89. **HRMS (ESI)** Calculated for $C_{102}H_{156}N_{32}NaO_{12}$: 2044.2473; found: 2044.2485.

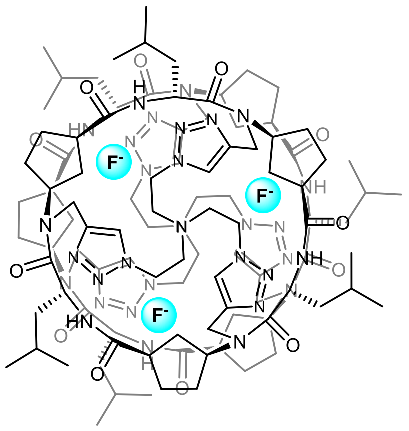

(mF$^-$·nH$_2$O)⊂2CP2. In an NMR tube, 450 µL of a solution of **D2** in 10% CD_3CN/CD_2Cl_2 (7 mM, 2.5 mM dioxane as standard) were treated with consecutives portions of a solution of TBAF in 10% CD_3CN/CD_2Cl_2 containing dioxane (2.5 mM) until 3.1 equivalents were added. **^{1}H-NMR** (500 MHz, CD_2Cl_2) δ (ppm): 9.99 (s, 3H, NH), 7.38 (s, 3H, $H_{triazole}$), 4.94 (d, *J* = 16.0 Hz, 3H, $(CH_2)_{linker}$), 4.90 (m, 3H, $H\gamma_{Acp}$), 4.19 (m, 3H, $H_{A'}$), 4.16 (m, 3H, $H\alpha_{Leu}$), 4.05 (d, *J* = 16.0 Hz, 3H, $(CH_2)_{linker}$), 3.81 (ddd, *J* = 14.4, 8.7 and 3.0 Hz, 3H, $H_{B'}$), 3.18 (m, 3H, Hα Acp), 2.74 (ddd, *J* = 14.7, 8.8 and 3.3 Hz, 3H, $H_{C'}$), 2.61 (dt, *J* = 15.1 and 3.9 Hz, 3H, $H_{D'}$), 2.15 (m, 3H, cis-$H\varepsilon_{Acp}$), 2.01 (m, 3H, *cis*-$H\delta_{Acp}$), 1.88 (m, 3H, $(CH_2)_{Leu}$), 1.80 (m, 3H, CH_{Leu}), 1.62 (m, 6H, *trans*-$H\delta_{Acp}$ + *trans*-$H\varepsilon_{Acp}$) 1.57 (m, 3H, *trans*-$H\beta_{Acp}$), 1.50 (m, 3H, $(CH_2)_{Leu}$), 1.39 (m, 3H, *cis*-$H\beta_{Acp}$) , 0.97 (d, *J* = 6.5 Hz, 9H, Me_{Leu}), 0.89 (d, *J* = 6.5 Hz, 9H, Me_{Leu}). **^{13}C-NMR** (126 MHz, CD_2Cl_2) δ (ppm): 176.8 (C), 173.7 (C), 148.3 (C), 124.4 (CH), 58.0 (CH_2), 55.6 (CH), 53.9 (CH_2), 49.9 (CH), 41.9 (CH_2), 39.8 (CH), 39.1 (CH_2), 32.5 (CH_2), 30.2 (CH_2), 29.0 (CH_2), 25.6 (CH), 23.3 (CH_3), 22.1 (CH_3). **FTIR (CaF2)**: ν = 3244 (amide A), 1632 (amide I), 1577 and 1556 cm-1 (amide II).

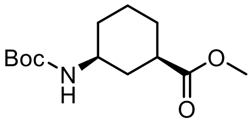

Boc-*L*-γ-Ach-OMe (2b). Amino acid **2a**, previously described,[10] (2.3 g, 9.46 mmol) was dissolved in dry DMF (34 mL) and then Na_2CO_3 (2.0 g, 19 mmol) and MeI (2.4 mL, 38 mmol) were added. The resulting solution was stirred overnight under Ar atmosphere. After this time, H_2O (30 mL) and AcOEt (30 mL) were added. The DMF was mostly removed by further extractions with H_2O (4x30 mL). The ethyl acetate layer was washed with saturated aqueous $NaHCO_3$ sat. (3x50 mL). The organic layer was dried over $MgSO_4$ (anh.), filtered, concentrated. The remaining DMF was removed by consecutives toluene additions followed by rotary evaporations. The resulting solid was purified by flash chromatography (5-20% AcOEt/hexane) to afford the desired product as a white solid [2.4 g, 98%]. **^{1}H-NMR** (500 MHz, $CDCl_3$) δ (ppm): 4.45 (br, 1H), 3.64 (s, 3H), 3.43 (br, 1H, Hγ), 2.37 (ddt, *J* = 12.0, 7.1 and 3.6 Hz, 1H), 2.22-2.12 (m, 1H), 1.92 (dddt, *J* = 17.9, 12.8, 3.4 and 1.7 Hz, 2H), 1.82 (dq, *J* = 13.3 and 3.4 Hz, 1H), 1.41 (s, 9H), 1.38 – 1.13 (m, 3H), 1.03 (qd, *J* = 12.3 and 3.7 Hz, 1H) . **^{13}C-NMR** (126 MHz, $CDCl_3$) δ (ppm): 175.5 (C), 155.2 (C), 79.3 (C), 51.7 (CH_3), 49.0 (CH), 42.3 (CH), 35.7 (CH2), 32.9 (CH_2), 28.5 (CH_3), 28.3 (CH2), 24.3 (CH_2). **MS (ESI)** [m/z (%)]: 158 (100) ([M-Boc]$^+$), 280 (11.2) ([M+Na]$^+$). **HRMS (ESI)** Calculated for $C_{13}H_{23}NO_4$: 258.1700, found: 258.1692.

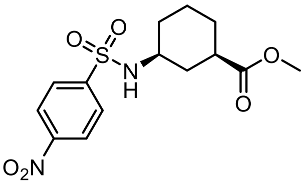

Ns-*L*-γ-Ach-OMe (2c). A solution of **2b** (2.4 g, 9.3 mmol) in CH_2Cl_2 (24 mL) was treated with TFA (24 mL). After 15 min, the solution was concentrated under vacuum and further dried at high vacuum for 2 h. The resulting solid was dissolved in dry CH_2Cl_2 (35 mL) and DIEA (6.5 mL, 37.8 mmol) was added, followed by *p*-nitrobenzenesulfonyl chloride (3.0 g, 13.9 mmol). The mixture was stirred overnight under Ar atmosphere. The solution was washed with aqueous HCl (5%, 2x50 mL) and saturated aqueous solution of $NaHCO_3$ (2x50 mL), dried over anhydrous $MgSO_4$, filtered, and concentrated under vacuum. The resulting residue was purified by flash chromatography (15-50% AcOEt/hexane) to provide **2c** as a yellow solid [2.7 g, 85%]. **^{1}H-NMR** (500 MHz, $CDCl_3$) δ (ppm): 8.35 (d, *J* = 8.8 Hz, 2H), 8.07 (d, *J* = 8.8 Hz, 2H), 6.68 (br, 0.5 H), 5.28 (d, *J* = 8.0 Hz, 2H), 3.89 (ddt, *J* = 11.0, 7.3 and 4.1 Hz, 0.5 H), 3.68 and 3.64 (2s, 3H), 3.22 (tdt, *J* = 11.7, 8.0 and 4.0 Hz, 1H, Hγ), 2.53-1.05 (m, 9H). **^{13}C-NMR** (126 MHz, $CDCl_3$) δ (ppm): 175.6 (C), 175.1 (C), 150.1 (C), 147,5 (C), 128.2 (CH), 124.6 (CH), 52.4 (CH_3), 52.0 (CH_3), 48.2 (CH), 42.0 (CH), 41.5 (CH), 35.8 (CH_2), 33.8 (CH_2), 33.5 (CH_2), 31.6 (CH_2), 28.1 (CH_2), 27.9 (CH_2), 23.8 (CH_2), 23.3 (CH_2). **MS (ESI)** [m/z (%)]: 311 (100) ([M-OMe]$^+$). **HRMS (ESI)** Calculated for $C_{14}H_{18}N_2O_6S$: 343.0958, found: 343. 0966.

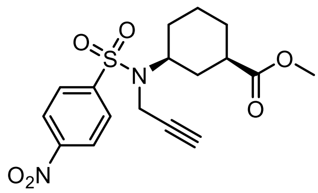

Ns-^{propargyl}*N-L*-γ-Ach-OMe (2d). Amino acid Ns-*L*-γ-Ach- OMe (**2c**) (1.8 g, 5.26 mmol) was dissolved in dry DMF (26 mL) and treated with K_2CO_3 (2.9 g, 21.0 mmol) and propargyl bromide (1.9 mL, 21 mmol). The mixture was stirred overnight under Ar atmosphere and then DMF was mostly removed by consecutives extractions with H_2O (4x30 mL) using CH_2Cl_2 as organic phase. The organic layer was washed with aqueous HCl (5%, 2x50 mL) and saturated aqueous solutions of $NaHCO_3$ (3x50 mL). The organic layer was dried over $MgSO_4$ (anh.), filtered, concentrated. The remaining DMF was removed by consecutives toluene addition followed by rotavapor concentrations. The product was purified by flash chromatography (10-20% AcOEt/hexane), providing the entitled product as a light-yellow viscous oil [1.9 g, 95%]. **^{1}H-NMR** (500 MHz, $CDCl_3$) δ (ppm): 8.34 (d, *J* = 8.9 Hz, 2H), 8.11 (d, *J* = 8.9 Hz, 2H), 4.28 – 4.09 (m, 2H), 3.78 (tt, *J* = 12.1 and 3.8 Hz, 1H, Hγ), 3.65 (s, 3H), 2.39 (tt, *J* = 12.1 and 3.6 Hz, 1H, Hα), 2.16 (t, *J* = 2.5 Hz, 1H, C≡CH), 2.02 – 1.92 (m, 2H), 1.88 (dt, *J* = 13.1 and 3.3 Hz, 1H), 1.80 – 1.64 (m, 2H), 1.54 (qd, *J* = 12.4 and 3.7 Hz, 1H), 1.40 – 1.15 (m, 2H). **^{13}C-NMR** (126 MHz, $CDCl_3$) δ (ppm): 174.8 (C), 150.2 (C), 146.9 (C), 128.7 (CH), 124.3 (CH), 79.2 (C), 73.5 (CH), 57.6 (CH_3), 51.6 (CH), 42.9 (CH), 33.5 (CH_2), 32.7 (CH_2), 30.6 (CH_2), 28.0 (CH_2), 24.8 (CH_2). **MS (ESI)** [m/z (%)]: 141 (100) ([(M+2Na+H)/3]$^+$). **HRMS (ESI)** Calculated for $C_{17}H_{21}N_2O_6S$: 381.1115, found 381.1124.

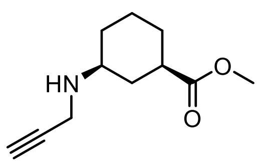

^{propargyl}*N-L*-γ-Ach-OMe (2e). Nosyl protected amino acid Ns-^{propargyl}*N-L*-γ-Ach-OMe (**2d**) (0.867 g, 2.3 mmol) was dissolved in MeCN (20 mL) and treated with K_2CO_3 (1.6 g, 11.4 mmol) and PhSH (2.0 mL, 18.4 mmol). The mixture was stirred overnight and then acetonitrile was evaporated under vacuum. The resulting residue was dissolved in CH_2Cl_2 and washed with H_2O (2x25 mL) and brine (2x25 mL). The organic phase was dried over $MgSO_4$ (anh.), filtered and concentrated under reduced pressure. The resulting residue was purified by flash chromatography (0-8% MeOH/CH_2Cl_2) to afford the desired product as colorless oil [0.365 g; 80%; R*f* = 0.13 (5% MeOH/CH_2Cl_2)]. **^{1}H-NMR** (500 MHz, $CDCl_3$) δ (ppm): 3.66 (s, 3H), 3.46 (d, *J* = 2.5 Hz, 2H), 2.70 (tt, *J* = 11.2 and 3.8 Hz, 1H, Hγ), 2.35 (tt, *J* = 12.1 and 3.4 Hz, 1H, Hα), 2.19 (t, *J* = 2.4 Hz, 1H, C≡CH), 2.14 (dtd, *J* = 10.3 and 3.6, 1.7 Hz, 1H), 1.99 – 1.80 (m, 3H), 1.36 – 1.29 (m, 2H), 1.20 (td, *J* = 12.4 and 11.2 Hz, 1H), 1.09 – 0.98 (m, 1H). **^{13}C-NMR** (126 MHz, $CDCl_3$) δ (ppm): 175.8 (C), 82.3 (C), 71.4 (CH), 54.4 (CH), 51.8 (CH_3), 42.2 (CH), 35.3 (CH_2), 35.2 (CH_2), 32.5 (CH_2), 28.7 (CH_2), 24.1 (CH_2). **MS (ESI)** [m/z (%)]: 196 (100) ([M]$^+$). **HRMS (ESI)** Calculated for $C_{11}H_{17}NO_2$: 196.1332, found 196.1330.

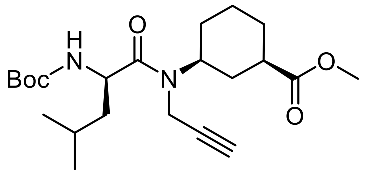

Boc-*D*-Leu-^{propargyl}*N-L*-γ-Ach-OMe (2f). Compound **2e** (365 mg, 1.9 mmol) was dissolved in dry CH_2Cl_2 (10 mL) and DIEA was added (1 mL, 6.3 mmol). Separately, the Boc-*D*-Leu-OH (512 mg, 2.1 mmol) was dissolved in dry CH_2Cl_2 (10 mL) and DIEA (1 mL, 6.3 mmol) and *N*-HATU was added (1.1 g, 2.85 mmol). Both solutions were mixed, and the reaction stirred under Ar for 4 hours. The resulting solution was washed with HCl 5% (2x5 mL) and sat. aq. $NaHCO_3$ (2x5 mL), dried with anhydrous $MgSO_4$, filtered, and concentrated under vacuum. The crude mixture was purified by flash column chromatography (0-4 % MeOH/CH_2Cl_2), to give the dipeptide as a foam [552 mg, 75%]. **^{1}H-NMR** (500 MHz, $CDCl_3$) δ (ppm): 5.26 and 5.06 (d, 1H, NH), 4.65 (m, 1H, Hα Leu), 4.46 and 3.87 (dd, 2H, N-CH_2), 4.38 and 3.78 (m, 1H, Hγ Ach), 4.07 (td, 1H, C≡CH), 3.70 and 3.66 (s, 3H, OMe), 2.45 (m, 1H, Hα Ach), 2.28 and 2.14 (2H, t, CH_2 Leu), 2.03-1.28 (m, 8H, Ach), 1.43 (s, 9H, Boc), 1.00 and 0.94 ppm (dd, 6H, CH_3). **^{13}C-NMR** (126 MHz, $CDCl_3$) δ (ppm): 175.3 (C), 174.8 (C), 173.5 (C), 172.3 (C), 155.6 (C), 155.5 (C), 80.4 (C), 80.1 (C), 79.6 (C), 79.5 (C), 72.6 (CH), 70.5 (CH), 55.8 (CH), 52.9 (CH), 51.9 (CH3), 51.7 (CH_3), 49.3 (CH), 48.8 (CH), 43.4 (CH_2), 42.9 (CH), 42.5 (CH), 42.3 (CH_2), 33.2 (CH2), 32.5 (CH_2), 31.9 (CH_2), 30.9 (CH_2), 30.6 (CH_2), 29.7 (CH_2), 28.3 (CH_3), 28.2 (CH_2), 28.0 (CH_2), 24.7 (CH), 24.6 (CH), 24.6 (CH_2), 24.5 (CH_2), 23.5 (CH_3), 23.4 (CH_3), 22.0 (CH_3), 21.7 (CH_3).

MS (ESI) [m/z (%)]: 449 (29) ([M+H]$^+$), ([M-tBu]$^+$) 393 (100) **HRMS (ESI)** Calculated for $C_{22}H_{36}N_2O_5$: 408.2621, found 408.2622.

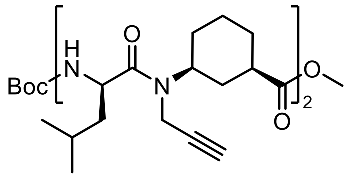

Boc-[*D*-Leu-*propargylN*-*L*-γ-Ach]$_2$-OMe (2g)**. A solution of Boc-*D*-Leu-^{propargyl}*N*-*L*-γ-Ach-OMe (**2f**) (184 mg, 0.45 mmol) in MeOH/H_2O (Vt = 3 mL, 3:1) was treated with LiOH (54 mg, 2.25 mmol) and stirred for 4 h. After this time, MeOH was concentrated under reduced pressure and the resulting solution was acidified until pH 3 (HCl 5% aq.) and extracted with CH_2Cl_2 (4x15 mL). The combined organic layers were dried with anh. $MgSO_4$, filtered and concentrated under reduced pressure to give the *C*- deprotected dipeptide. Parallelly, a different fraction of Boc-*D*-Leu-^{propargyl}*N*-*L*-γ-Ach-OMe (**2f**) (184 mg, 0.45 mmol) was dissolved in CH_2Cl_2 (3 mL) and treated with TFA (3 mL). After stirring for 30 min, the solvent was removed under reduced pressure and the resulting oil was redissolved in CH_2Cl_2 and further evaporated, repeating this process up to three times. The *C*- deprotected dipeptide was dissolved in dry CH_2Cl_2 (3 mL) and subsequently DIEA (0.30 mL, 1.35 mmol) and *N*-HBTU (255 mg, 0.675 mmol) were added. The *N*-deprotected dipeptide was dissolved in dry CH_2Cl_2 (3 mL) and DIEA was added (1.5 mL, 6.75 mmol). Finally, both fractions were mixed and reacted under Ar for 4 h. This solution was washed with HCl 5% (2x15 mL) and sat. aq. $NaHCO_3$ (2x15 mL), dried with anhydrous $MgSO_4$, filtered and concentrated under reduced pressure. The crude mixture was purified by flash column chromatography (25-50% AcOEt/hexane) to give the title compound as a foam [297 mg, 95%].^{1}**H-NMR** (500 MHz, $CDCl_3$) δ (ppm): 6.33 and 6.08 (dd, 1H, NH), 5.23 and 5.09 (dd, 1H, NH), 4.97 and 4.65 (m, 2H, Hα Leu), 4.40 (m, 2H, Hγ Ach), 4.11-3.73 (m, 4H, propargylic CH_2), 3.62-3.73 (m, 3H, OMe), 2.80 (s, 2H, C≡CH), 2.45 (m, 1H, Hα Ach), 2.33-2.20 (m, 1H, Hα Ach), 1.43 (s, 9H, Boc), 0.99 and 0.93 ppm (m, 12H, CH_3 Leu). **MS (ESI)** [m/z (%)]: 685.5 (100) ([M+H]$^+$), 585 (61) ([M-Boc]$^+$), 342.1 (30) ([(M+2) /2]$^+$). **HRMS (ESI)** calculated for $C_{38}H_{61}N_4O_7$ 685.4535, 685.4533.

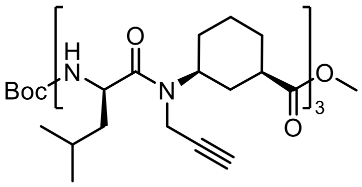

Boc-[*D*-Leu-*propargylN*-*L*-γ-Ach]$_2$-OMe (2h).** The tetrapeptide Boc-*D*-Leu-^{propargyl}*N*-*L*-γ-Ach-*D*-Leu-^{propargyl}*N*-*L*-γ-Ach-OMe (**2g**) (297 mg, 0.43 mmol) was dissolved in CH_2Cl_2 (7 mL) and treated with TFA (1.3 mL). After stirring for 30 min, the solvent was removed under reduced pressure and the resulting oil was redissolved in CH_2Cl_2 and further evaporated, repeating this process up to three times. Parallelly, the dipeptide Boc-*D*-Leu-^{propargyl}*N*-*L*-γ-Ach-OMe (**2f**) (184 mg, 0.45 mmol) was dissolved in MeOH/H_2O (Vt = 3 mL, 3:1) was treated with LiOH (54 mg, 2.25 mmol) and stirred for 4 h. After this time, MeOH was concentrated under reduced pressure and the resulting solution was acidified until pH 3 (HCl 5% aq.) and extracted with CH_2Cl_2 (4x15 mL). The combined organic layers were dried with anh. $MgSO_4$, filtered and concentrated under reduced pressure to give the *C*- deprotected dipeptide. The *C*- deprotected dipeptide was dissolved in dry CH_2Cl_2 (3 mL) and subsequently DIEA (0.30 mL, 1.35 mmol) and *N*-HBTU (256 mg, 0.675 mmol) were added. The TFA salt was dissolved in dry CH_2Cl_2 (3 mL) and DIEA was added (1.5 mL, 6.75 mmol). Finally, both fractions were mixed and reacted under Ar for 4 h. This solution was washed with HCl 5% (2x15 mL) and sat. aq. $NaHCO_3$ (2x15 mL), dried with anhydrous $MgSO_4$, filtered, and concentrated under reduced pressure. The crude mixture was purified by flash column chromatography (25-70% AcOEt/hexane) to give the title compound as a white foam. [272 mg, 66%]. ^{1}**H-NMR** (500 MHz, $CDCl_3$) δ (ppm):6.38-6.05, 6.58-5.14, 4.71-4.56 (m, 3H, NH), 4.99 y 4.64 (m, 3H, Hα Leu), 4.42 (m, 3H, Hγ Ach), 4.15-3.72 (m, 6H, propargylic CH_2), 3.62-3.72 (m, 3H, OMe), 2.28 (m, 3H, C≡CH), 2.43, 2.14 (m, 3H, Hα Ach), 2.03-1.50 (m, 33H), 1.43 (s, 9H, Boc), 0.96 ppm (m, 18H, CH_3 Leu). **MS (ESI)** [m/z (%)]: 881 (100) ([M-Boc]$^+$), 961.6 (31) ([M+H]$^+$). **HRMS (ESI)** Calculated for $C_{54}H_{84}N_6O_9$: 961.6373; found: 961.6371.

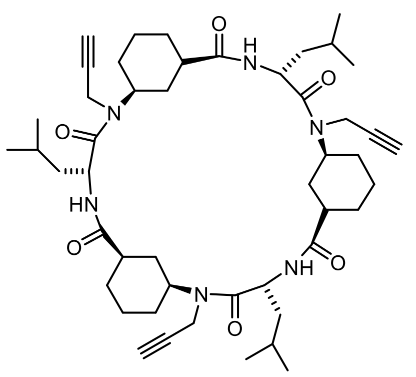

c-[(*D*-Leu-^{propargyl}*N*-*L*-Ach)$_3$] (CP3). The hexapeptide **2h** (182 mg, 0.19 mmol) was dissolved in MeOH (2 mL) and water (0.5 mL) and then LiOH (23 mg, 0.95 mmol) was added. After stirring for 4h, the organic solvent was evaporated, and the remaining aqueous layer was acidified until pH 3 with HCl aq. (5%). The resulting white precipitate was extracted with CH_2Cl_2 (4x5 mL), dried, filtered, and concentrated. Free acid was further deprotected by a solution of TFA/CH_2Cl_2 (1:1, Vt = 3.5 mL), in which the product was stirred for 15 min. After completion of the reaction, TFA was removed under reduced pressure and the resulting oil was redissolved in CH_2Cl_2 and further evaporated, repeating this process up to three time. For the cyclization step, the oil obtained in the previous step was dissolved in dry CH_2Cl_2 (126 mL) and treated with DIEA (1.5 mL, 6.75 mmol) and *N*-TBTU (183 mg, 0.57 mmol). The mixture was stirred overnight under Ar and then washed with 5% HCl (2x15 mL) and sat. aq. $NaHCO_3$ (2x15 mL). The organic layer was dried with $MgSO_4$, filtered and concentrated. The crude was purified by flash column chromatography CH_2Cl_2-MeOH 0-10% (120 mg, 77%). **^{1}H-NMR** (500 MHz, $CDCl_3$) δ (ppm): 8.28 (d, *J* = 9.4 Hz, 3H, NH), 5.17 (td, *J* = 10.0 and 4.0 Hz, 3H, Hα Leu), 4.87 (d, *J* = 19.1 Hz, 3H, propargylic H_1), 4.48 (tt, *J* = 12.6 and 3.9 Hz, 3H, Hγ Ach), 3.96 (d, *J* = 19.2 Hz, 3H, propargylic H_2), 2.75 (ddt, *J* = 11.8, 7.8 and 4.1 Hz, 3H, Hα Ach), 2.25 (s, 3H, C≡CH), 1.91 (m, 3H, Hε Ach), 1.84-1.76 (m, 9H, Ach), 1.74 (m, 6H, CH_2 Leu), 1.58-1.42 (m, 10H, 3H CH Leu, 6H Ach), 1.36 (m, 6H, Ach), 0.94 (dd, *J* = 14.8 and 6.5 Hz, 18H, CH_3 Leu). **^{13}C-NMR** (126 MHz, CD_2Cl_2) δ (ppm): 175.7 (C), 174.2 (C), 81.6 (C), 72.5 (CH), 52.4 (CH), 47.6 (CH), 43.3 (CH), 41.2 (CH_2), 32.8 (CH_2), 32.2 (CH_2), 30.3 (CH_2), 30.0 (CH_2), 25.8 (CH_2), 25.6 (CH), 24.0 (CH_3), 22.2 (CH_3). **FTIR (CaF2)**: ν = 3295 (amide A), 1623 (amide I), 1525 cm-1 (amide II). **MS (ESI)** [m/z (%)]: 829.5 (100) ($[M+H]^+$), 846.6 (85) ($[M+Na]^+$). **HRMS (ESI)** Calculated for $C_{48}H_{72}N_6O_6$: 829.5586; found: 829.5584.

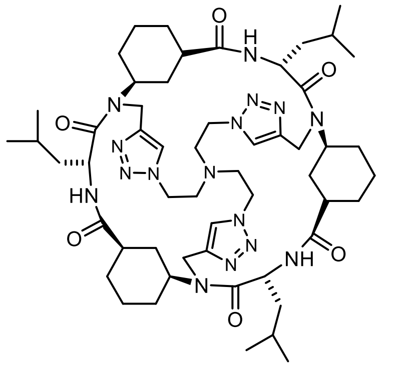

c-[(*D*-Leu-^{triazolylamine}*N*-*L*-Ach)$_3$] (D4). The cyclic peptide **CP3** (30 mg, 0.036 mmol), the tris(2-azidoethyl)amine (12 mg, 0.054 mmol) and DBU (22 uL, 0.144 mmol), were dissolved in dry toluene (40 mL). Then, the solution was degassed with Ar flow for 40 minutes. After that, mixture was heated until reflux was observed (110˚ C). Then, CuBr (1.5 mg, 0.0048 mmol) was added. The mixture was stirred overnight under Ar at 110˚ C. After cooling down, the mixture was filtered over filter paper. The product, that remains in the filter, was recovered using methanol as solvent. The methanol was removed under reduced pressure and the mixture was dissolved in CH_2Cl_2 (30 mL) in a 100 mL round bottom flask. An aqueous solution of KCN (10mg/mL) (30 mL) was added in the flask and the two phases were left to stir for two hours. After recovering the organic layer, it was dried with $MgSO_4$, filtered and concentrated. The crude was purified by flash column chromatography 0-10% MeOH-CH_2Cl_2 and after that, by normal-phase HPLC (2-10% MeOH-CH_2Cl_2 over 30 min) to give the product as a white solid (19 mg, 50%, t_R = 15 min). **^{1}H-NMR** (500 MHz, CD_2Cl_2) δ (ppm)7.79 (br s, 3H, NH), 7.64 (s, 3H, H triazole), 4.79 (m, 6H, Hα Leu + Hγ Ach), 4.67 (d, *J* = 17.7 Hz, 3H, methylene linker H_1), 4.57 (d, *J* = 17.7 Hz, 3H, methylene linker H_2), 4.13 (m, 3H, H_A),3.30 (m, 6H, H_B + H_C), 2.83 (m, 3H, Hα Ach), 2.70 (m, 3H, H_D), 1.94 (m, 3H, Ach), 1.83-1.63 (m, 9H, Ach (1.80 ppm *cis*-$H_β$ Ach)), 1.60-1.46 (m, 9H, 3H $CH_{2(1)}$ Leu + 3H CH Leu + 3H Ach), 1.45-1.33 (m, 6H, 3H $CH_{2(2)}$ Leu + 3H Ach), 1.33-1.18 (m, 6H, Ach (1.23 ppm *trans*-$H_β$ Ach)), 0.87 (18H, dd, *J* = 21.9, 6.3 Hz, CH_3 Leu). **^{13}C-NMR** (126 MHz, CD_2Cl_2) δ (ppm): 174.8 (C), 174.3 (C), 145.6 (C), 124.1 (CH), 52.7 (CH_2), 48.5 (CH_2), 44.5 (CH), 44.0 (CH_2), 39.4 (CH_2), 34.5 (CH_2), 25. (CH_2), 25.4 (CH), 23.8 (CH_3), 22.2 (CH_3). **FTIR (CaF_2)**: ν = 3313 (amide A), 1614 (amide I), 1526 cm-1 (amide II). **MS (ESI)** [m/z (%)]: 1053.7 ($[M]^+$, 100). **HRMS (ESI)** Calculated for $C_{54}H_{84}N_{16}O_6$: 1053.6833; found: 1053.6871.

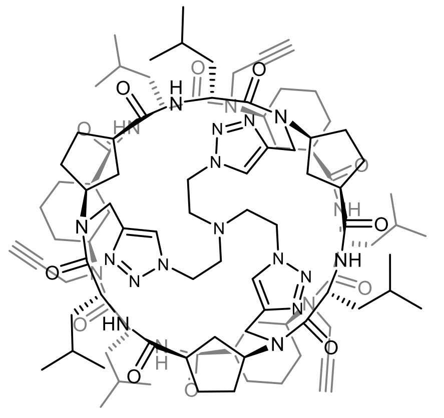

D2-3. 450 µL of **D2** (6.4 mM in 10% CD_3CN/CD_2Cl_2, dioxane 2.5 mM as internal standard) were placed into al NMR tube. Following the assembly by NMR, **D3** was added (38 µL from a stock solution 59 mM) until no signals from **D2** were observed, due to the formation of the heterodimeric specie **D2-3**. **^{1}H-NMR** (500 MHz, CD_2Cl_2) δ (ppm): 8.21 (d, *J* = 9.8 Hz, 3H, NH_{ACP}), 7.64 (d, *J* = 9.2 Hz ,3H, NH_{Ach}), 7.74 (s, 3H, $H_{triazole}$), 5.04 (td, *J* = 9.8 and 3.9 Hz, 3H, Hα Leu_{Ach}), 4.91 (m, 3H, Hα Leu_{Acp}), 4.83 (m, 3H, Hγ Acp), 4.72 (dd, *J* = 18.9 and 2.5 Hz, 3H, propargylic Ach H_1), 4.50 (s, 6H, methylene linker Acp), 4.40 (td, *J* = 12.3 and 6.1 Hz, 3H, Hγ Ach), 4.04 (d, *J* = 13.9 Hz, 3H, CAP Acp H_A), 3.91 (dd, *J* = 19.1 and 2.5 Hz, 3H, propargylic Ach H_1), 3.60 (m, 3H, CAP Acp H_B), 3.05 (m, 6H, Hα Acp + CAP Acp H_C), 2.93 (m, 3H, CAP Acp H_D), 2.50 (td, *J* = 11.6, 9.7 and 5.7 Hz, 3H, Hα Ach), 2.40 (m, 3H, *cis*-H_β Acp), 2.37 (s, 3H, , C≡CH Ach), 2.10-1.17 (57H, Hδ Acp + Hε Acp + *trans*-H_β Acp + Hδ Ach + Hε Ach + Hβ Ach + CH_2 Leu Ach + CH_2 Leu Acp + CH Leu Ach + CH Leu Acp), 0.95-0.80 (36H, CH_3 Leu Acp + CH_3 Leu Ach). **^{13}C-NMR** (126 MHz, CD_2Cl_2) δ (ppm): 175.9 (C), 178.0 (C), 174.6 (C), 174.0 (C), 144.6 (C), 124.8 (CH), 81.2 (C), 72.6 (CH), 67.4 (CH_2), 57.3 (CH_2), 56.3 (CH), 52.8 (CH), 52.2 (CH), 50.6 (CH_2), 49.0 (CH), 47.6 (CH), 44.4 (CH), 43.3 (CH_2), 42.3 (CH), 41.4 (CH_2), 39.6 (CH_2), 36.3 (CH_2), 32.6 (CH_2), 32.4 (CH_2), 30.1 (CH_2), 29.9 (CH_2), 29.5 (CH_2), 27.3 (CH_2), 25.9 (CH), 25.2 (CH), 23.8 (CH_3), 23.2 (CH_3), 22.4 (CH_3), 21.9 (CH_3). **HRMS (ESI)** Calculated for $C_{99}H_{151}N_{22}O_{12}$: 1840.1876; found: 1840.1846.

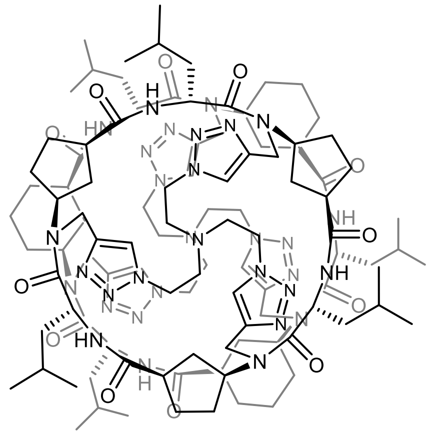

D2-4. 450 µL of D2 (8 mM in 10% CD_3CN/CD_2Cl_2 with dioxane 2.5 mM as internal standard) were placed into al NMR tube. Following the assembly by NMR, **D4** was added (38 µL from a stock solution 59 mM) until no signals from **D2** were observed, due to the formation of the heterodimeric specie **D2-4**. **^{1}H-NMR** (500 MHz, CD_2Cl_2) δ (ppm): 8.02 (broad s, 3H, NH Acp), 7.91 (broad s, 3H, NH Ach), 7.70 (s, 6H, triazole Acp + triazole Ach), 4.94 (m, 3H, Hα Leu_{Acp}), 4.81 (m, 3H, Hγ Acp), 4.75 (m, 3H, Hα Leu_{Ach}), 4.67-4.47 (m, 12H, 6H methylene linker Acp + 6H methylene linker Ach + 3H Hγ Ach), 4.23 (m, 3H, CAP Ach H_A), 4.02 (d, *J* = 13.9 Hz, 3H, CAP Acp H_A), 3.64 (m, 3H, CAP Acp H_B), 3.44 (s, 3H, CAP Ach H_B), 3,22-3.08 (m, 6H, CAP Ach H_C + CAP Acp H_C), 2.98 (m, 3H, Hα Acp), 2.86 (d, *J* = 15.1 Hz, 3H, CAP Acp H_D), 2.80 (m, 3H, CAP Ach H_D), 2.63 (m, 3H, Hα Ach), 2.49 (m, 3H, $H\beta_{Acp}$), 2.14-1.11 (m, 75H, Acp + Ach + Leu), 0.97-0.75 (m, 36H, CH_3 Leu). **^{13}C-NMR** (126 MHz, CD_2Cl_2) δ (ppm): 175.5 (C), 174.4 (C), 145.5 (C), 144.7 (C), 124.3 (CH), 67.4 (CH_2), 56.8 (CH), 50.5 (CH_2), 49.1 (CH), 48.4 (CH), 46.3 (CH_2), 45.0 (CH) , 43.5 (CH_2), 42.2 (CH), 39.9 (CH_2), 39.2 (CH_2), 36.6 (CH_2), 34.3 (CH_2), 30.0 (CH_2), 29.6 (CH_2), 29.0 (CH_2), 26.3 (CH_2), 25.9 (CH_2), 25.3 (CH), 25.2 (CH), 23.6 (CH_3), 23.4 (CH_3), 22.2 (CH_3), 21.8 (CH_3). **HRMS (ESI)** Calculated for $C_{105}H_{162}N_{32}NaO_{12}$: 2086.2942; found: 2086.2933.

8. SUPPLEMENTARY SPECTRA

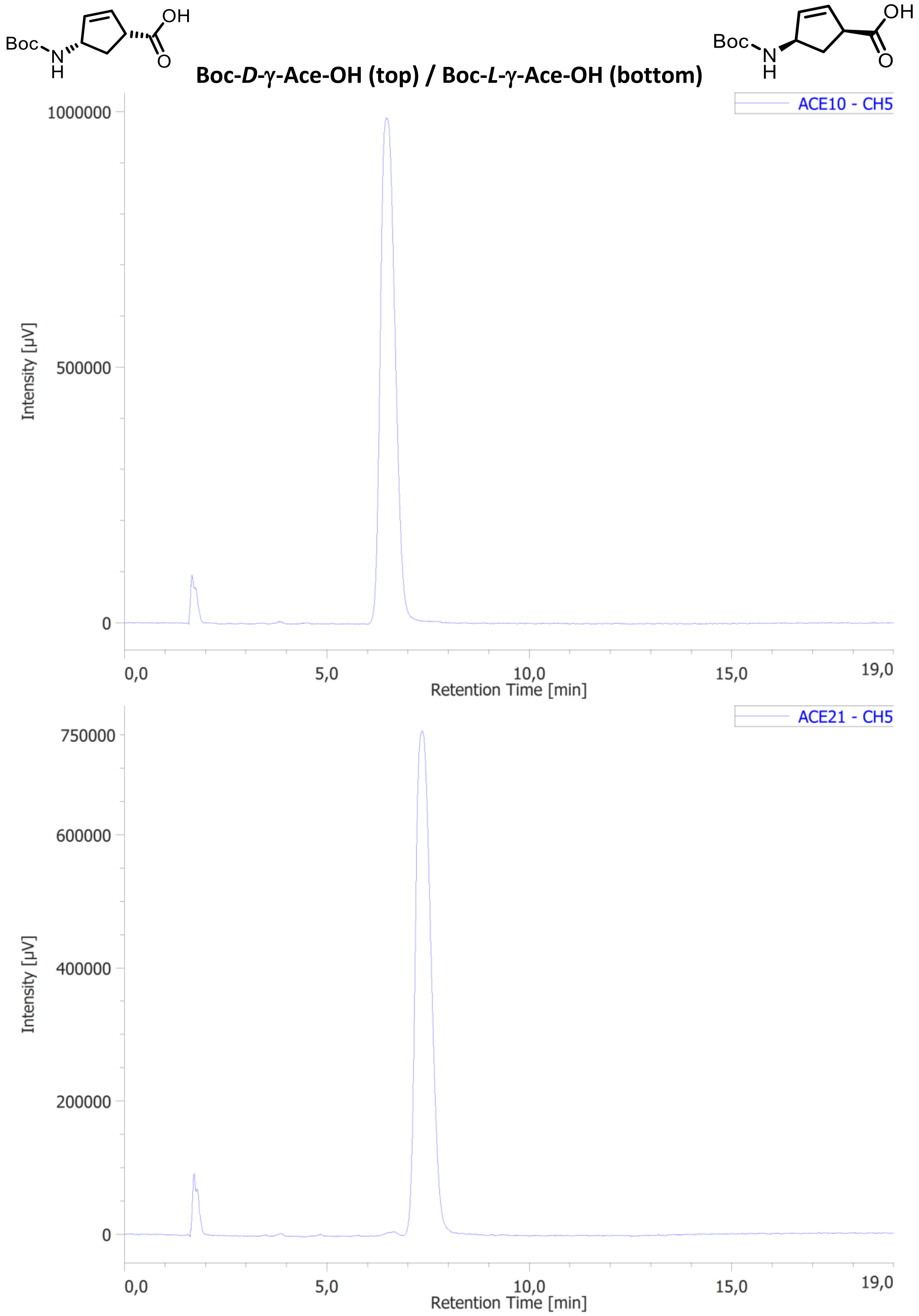

Supplementary Fig. 48. Superfluid Liquid Chromatography (SFC) chromatograms for the enantiopure Boc-*D*-γ-Ace-OH and Boc-*L*-γ-Ace-OH species. The chromatograms were recorded by following the absorbance at 222 nm.

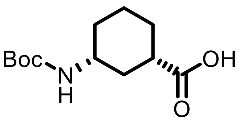

Boc-*D*-γ-Ach-OH (top) / Boc-*L*-γ-Ach-OH (bottom)

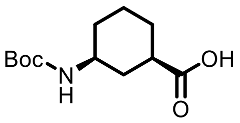

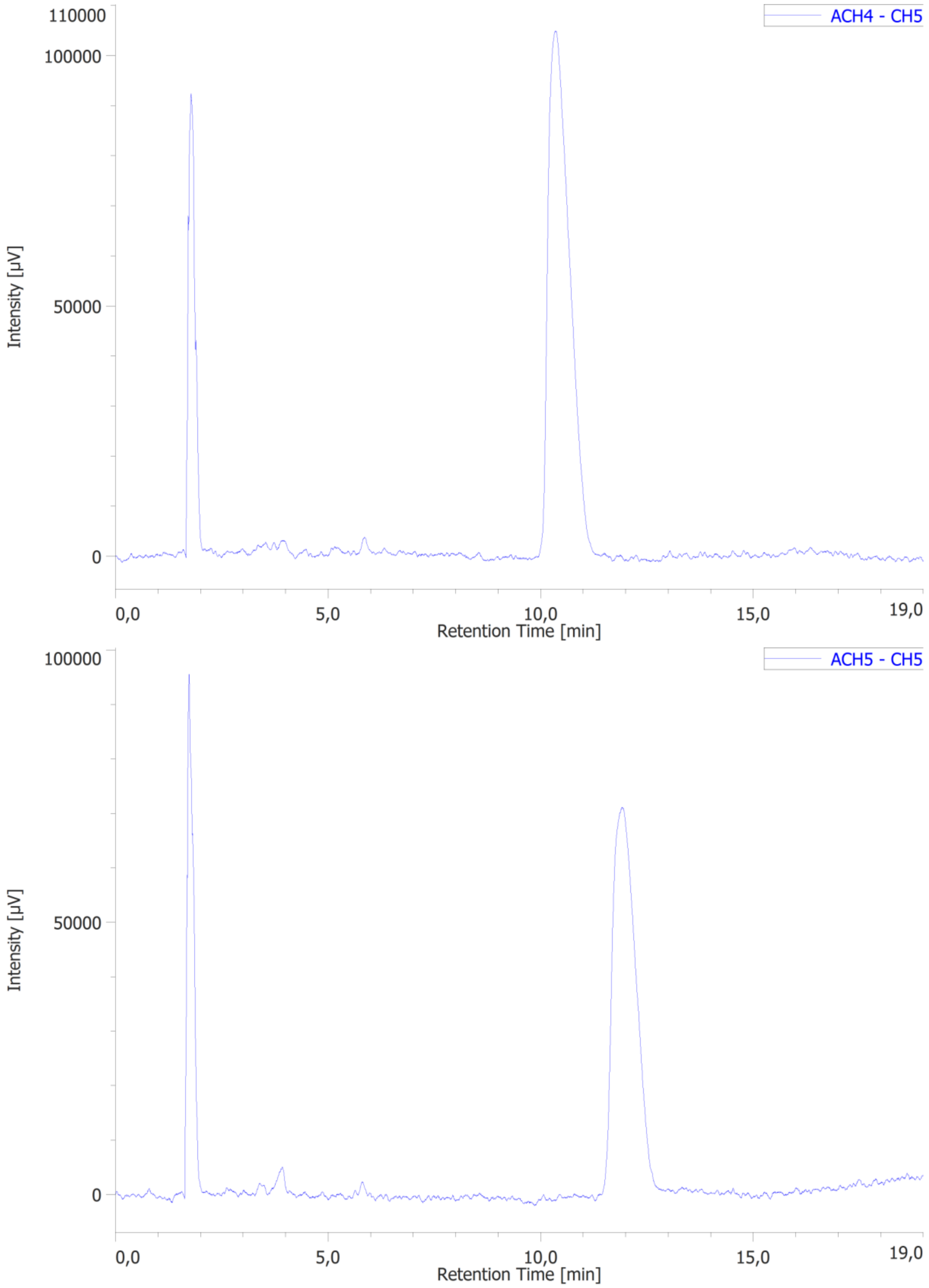

Supplementary Fig. 49. Superfluid Liquid Chromatography (SFC) chromatograms for the enantiopure Boc-*D*-γ-Ach-OH and Boc-*L*-γ-Ach-OH species. The chromatograms were recorded by following the absorbance at 222 nm.

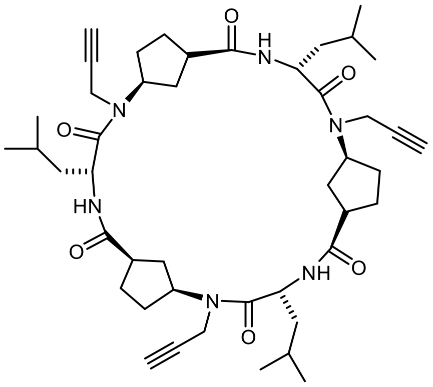

CP1

^{1}H-NMR ($CDCl_3$, 298 K, 300 MHz)

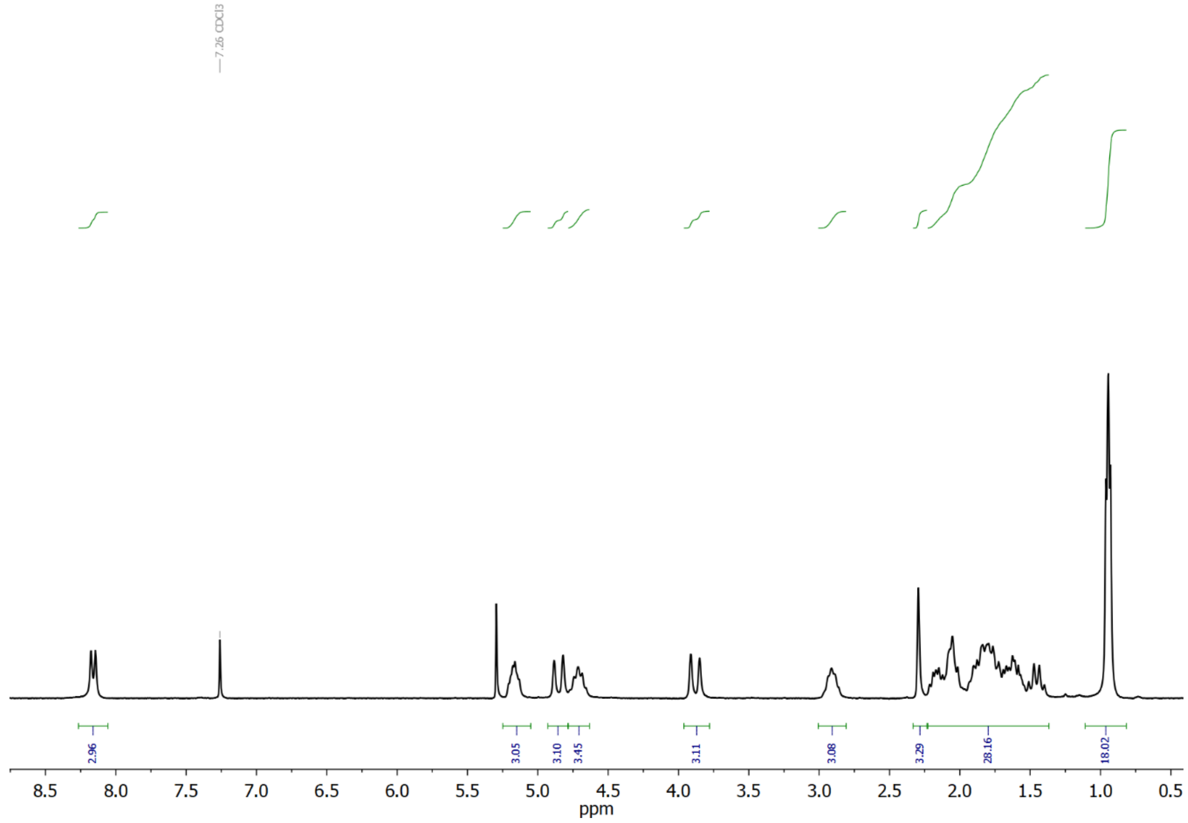

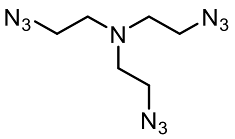

Tris(2-azidoethyl)amine

^{1}H-NMR ($CDCl_3$, 298 K, 300 MHz)

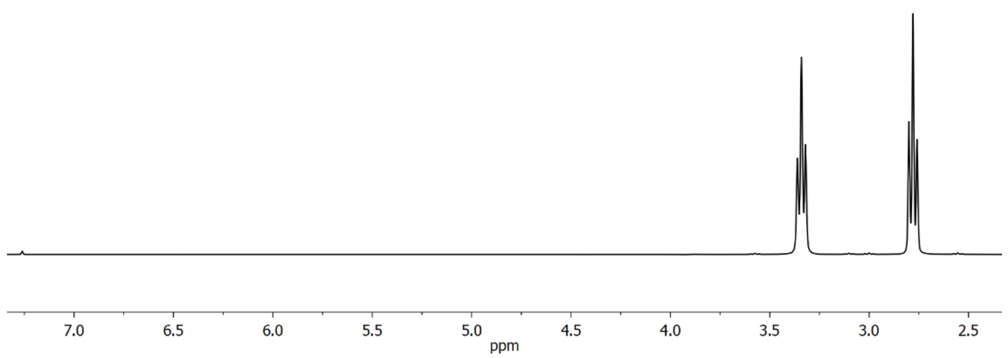

^{13}C and DEPT NMR ($CDCl_3$, 298 K, 75 MHz)

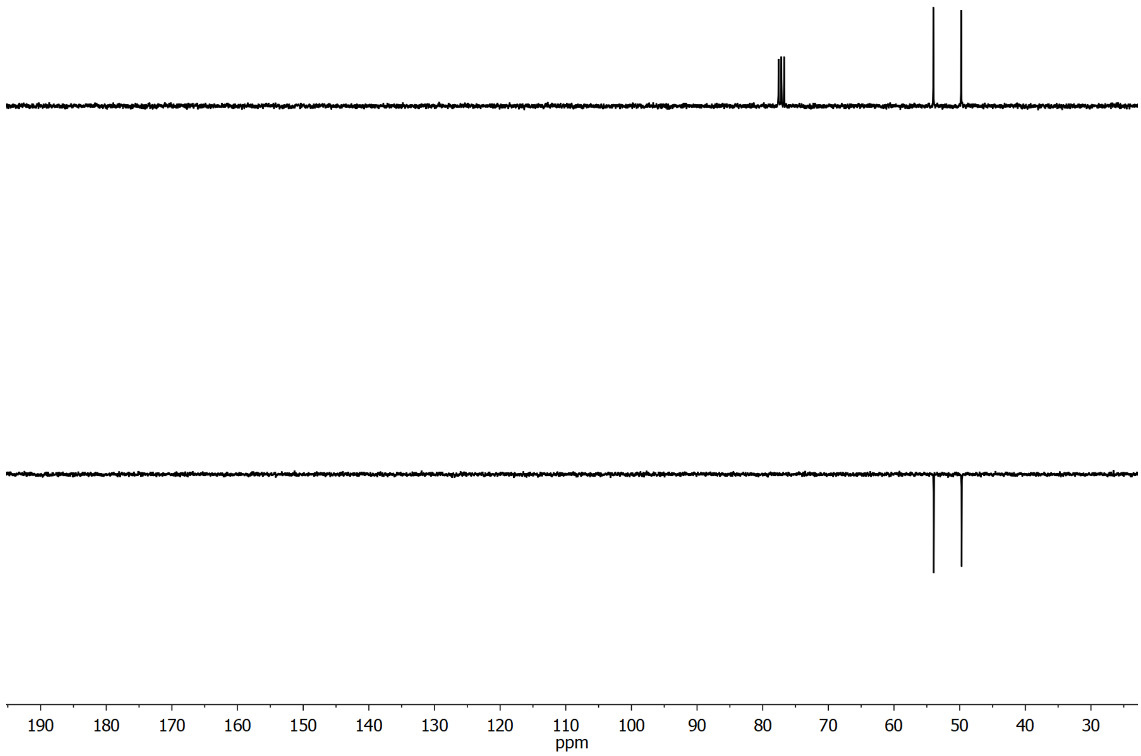

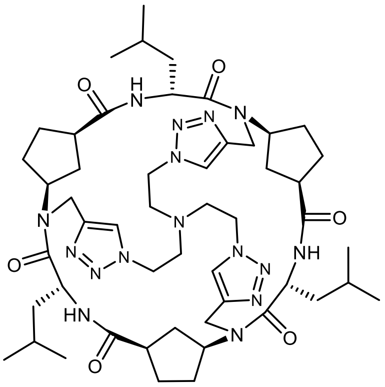

D2

HPLC-CROMATOGRAM

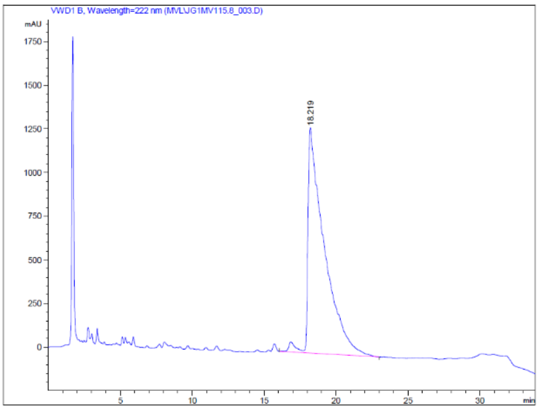

IR

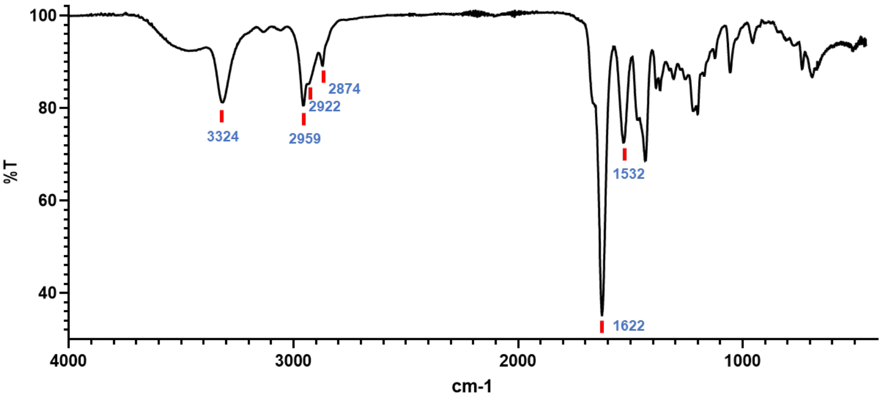

^{1}H-NMR (CD_2Cl_2, 298 K, 500 MHz)

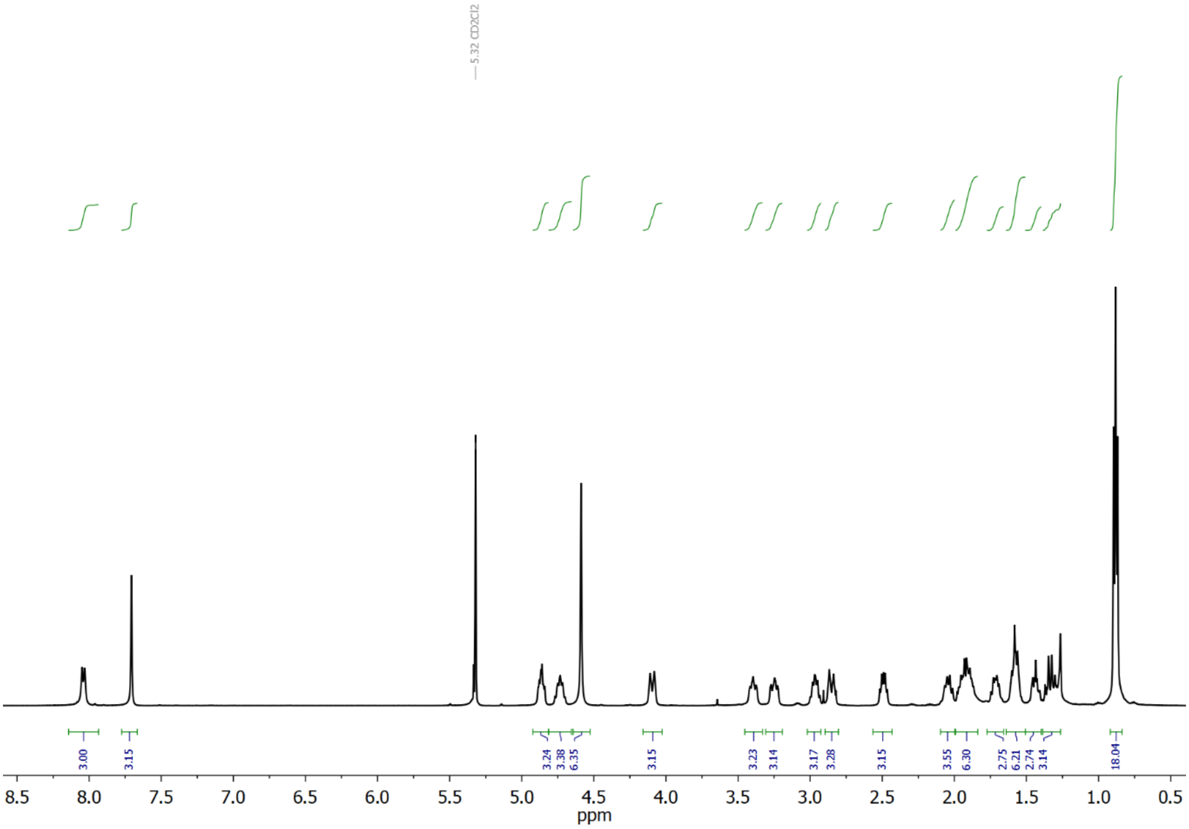

^{13}C and DEPT NMR (CD_2Cl_2, 298 K, 126 MHz)

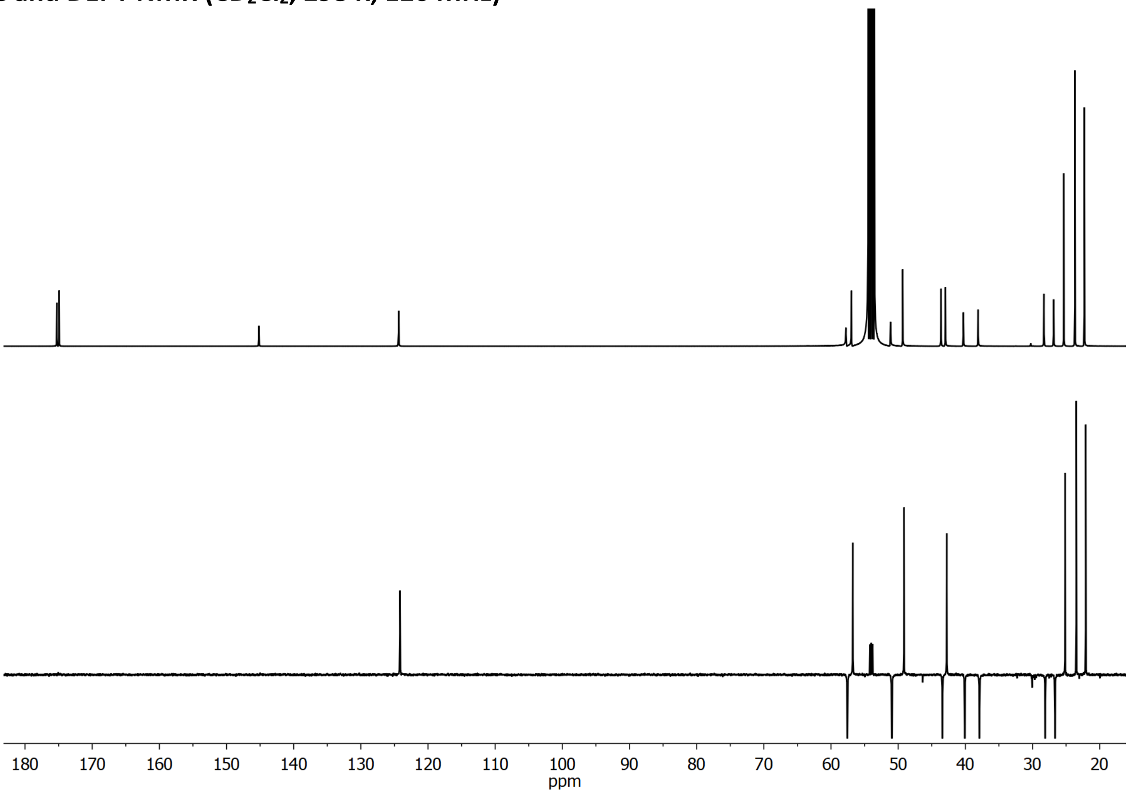

COSY (CD_2Cl_2, 298 K, 500 MHz)

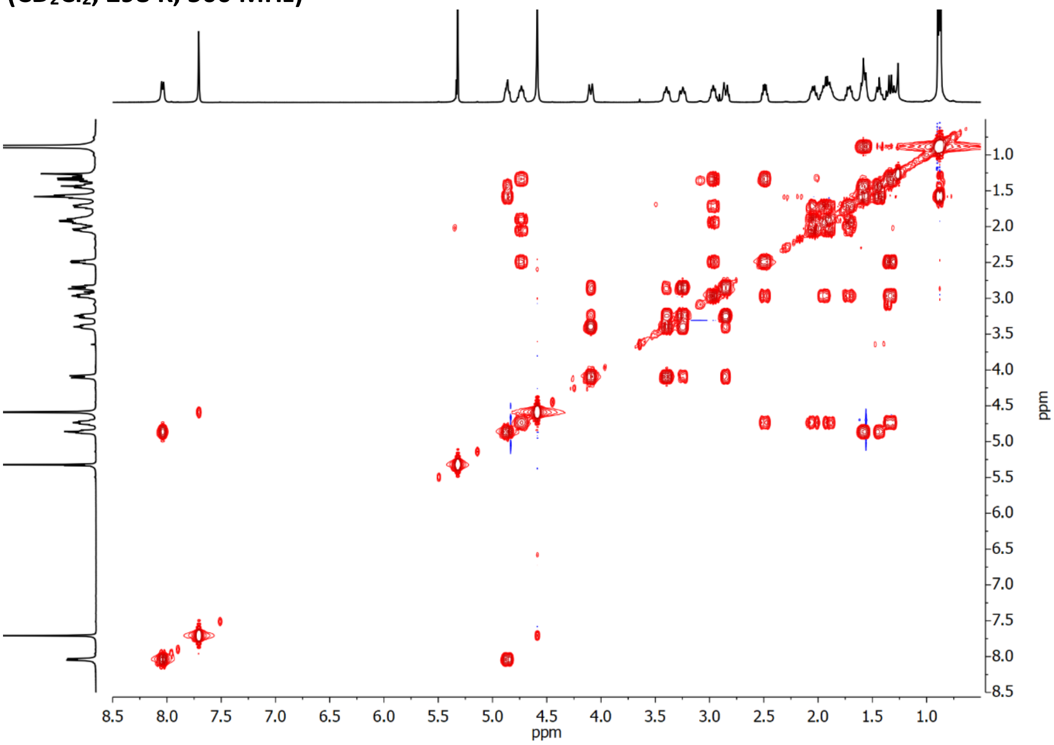

TOCSY (CD_2Cl_2, 298 K, 500 MHz)

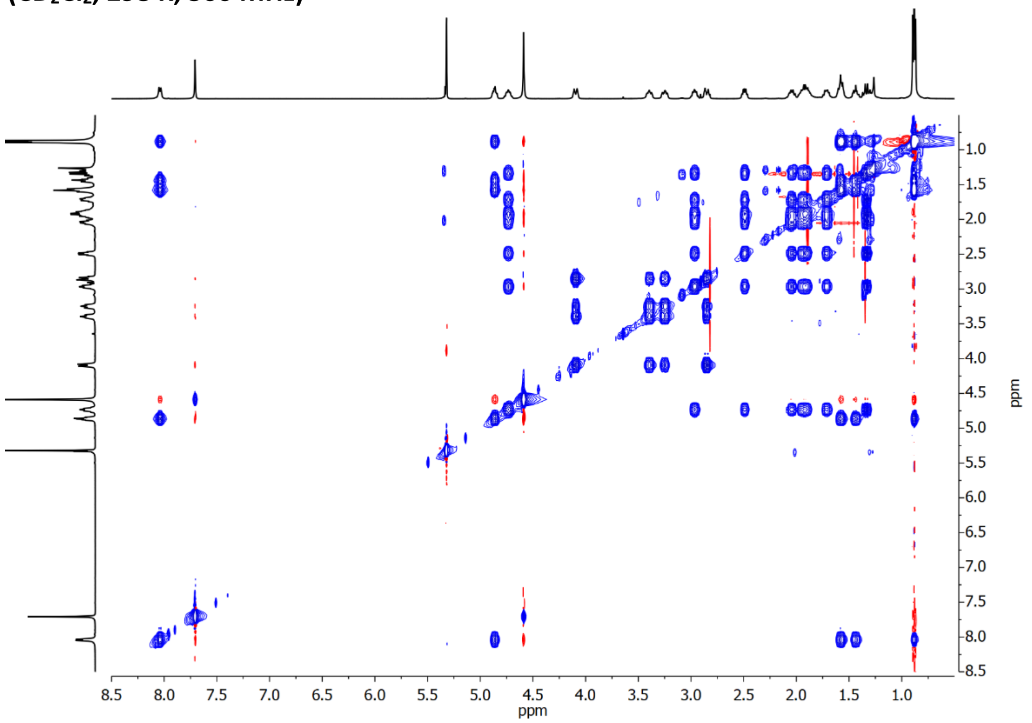

ROESY (CD_2Cl_2, 298 K, 500 MHz)

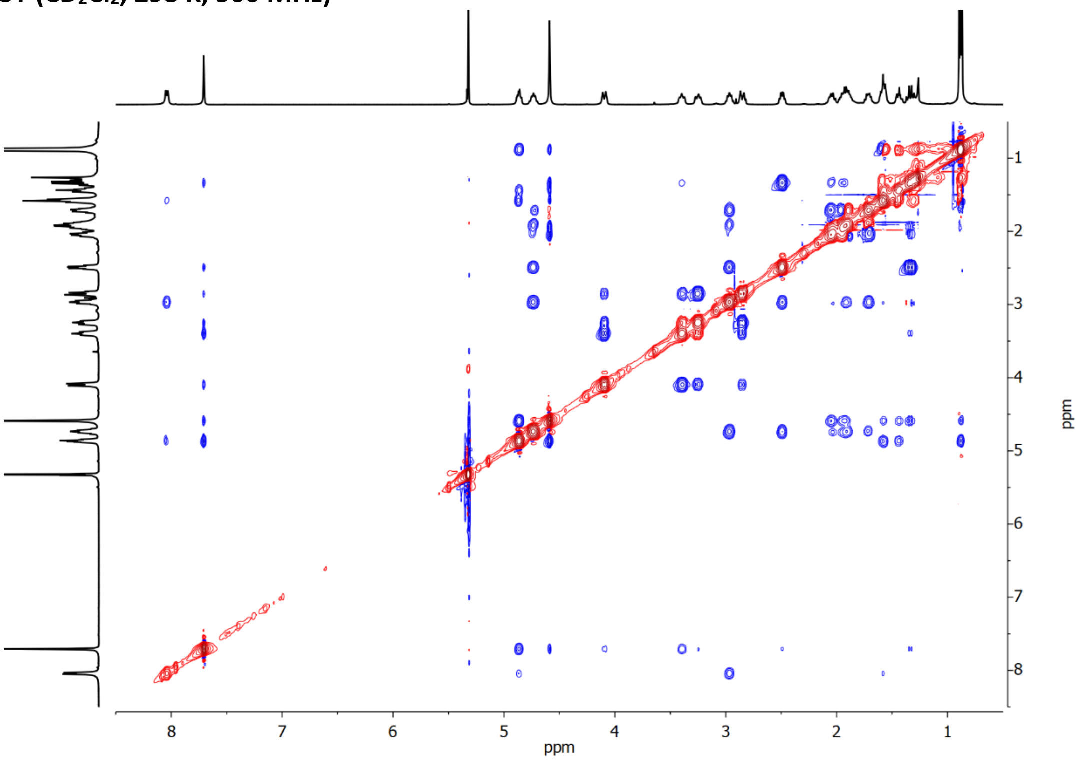

HSQC (CD_2Cl_2, 298 K, 500 MHz)

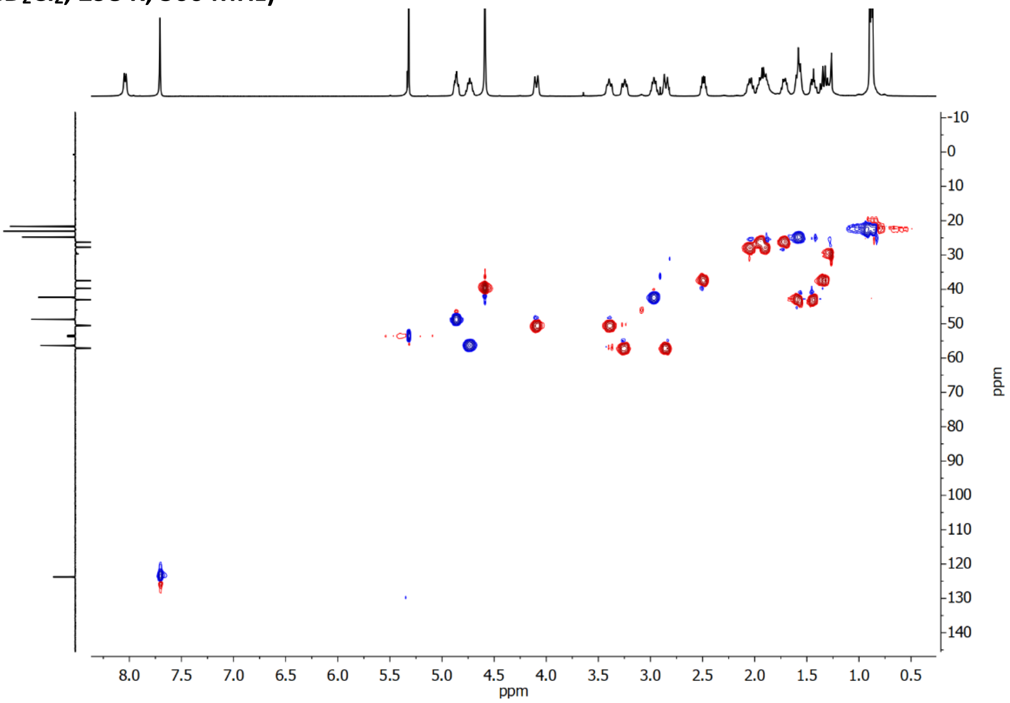

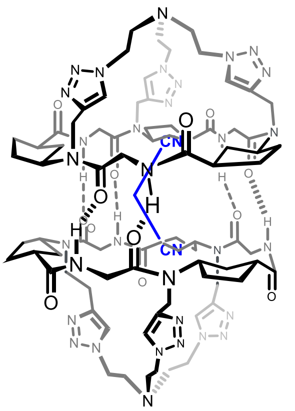

D2-manolonitrile

^{1}H-NMR (CD_2Cl_2/CD_3CN, 298 K, 500 MHz)

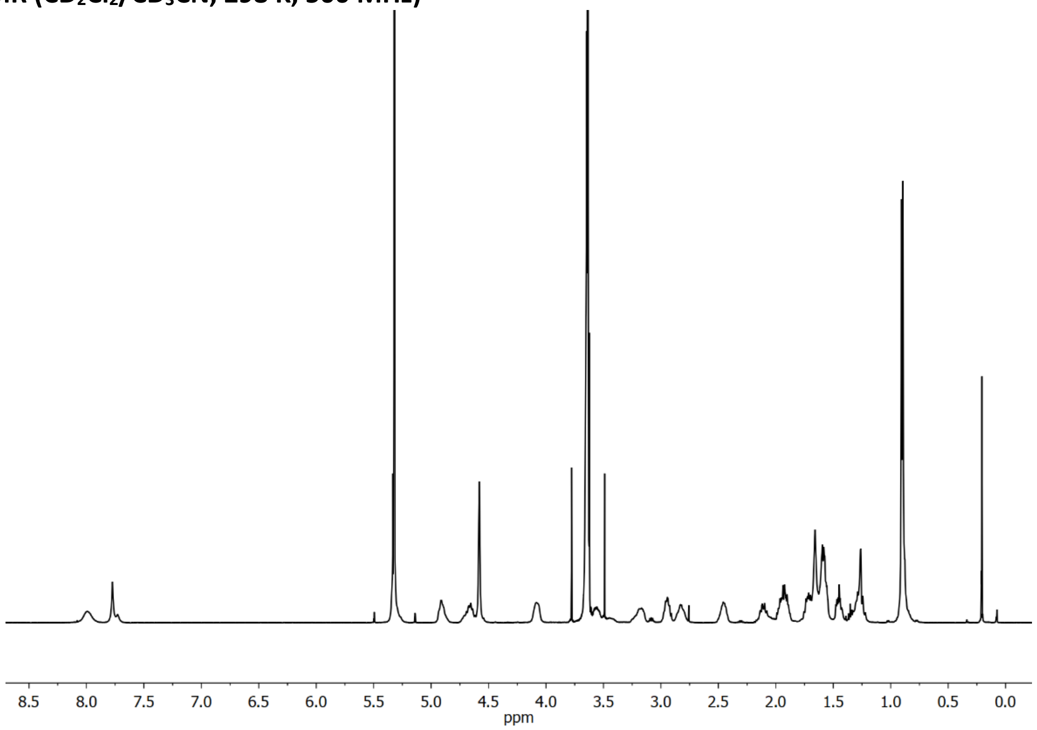

COSY (CD_2Cl_2/CD_3CN, 298 K, 500 MHz)

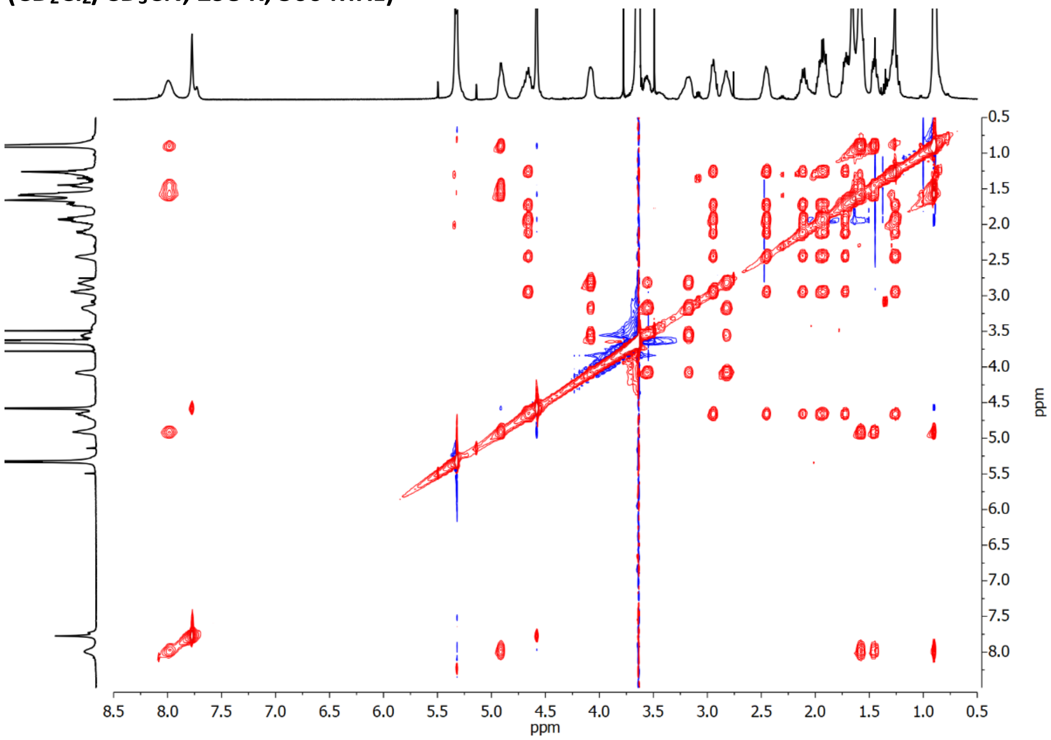

TOCSY (CD_2Cl_2/CD_3CN, 298 K, 500 MHz)

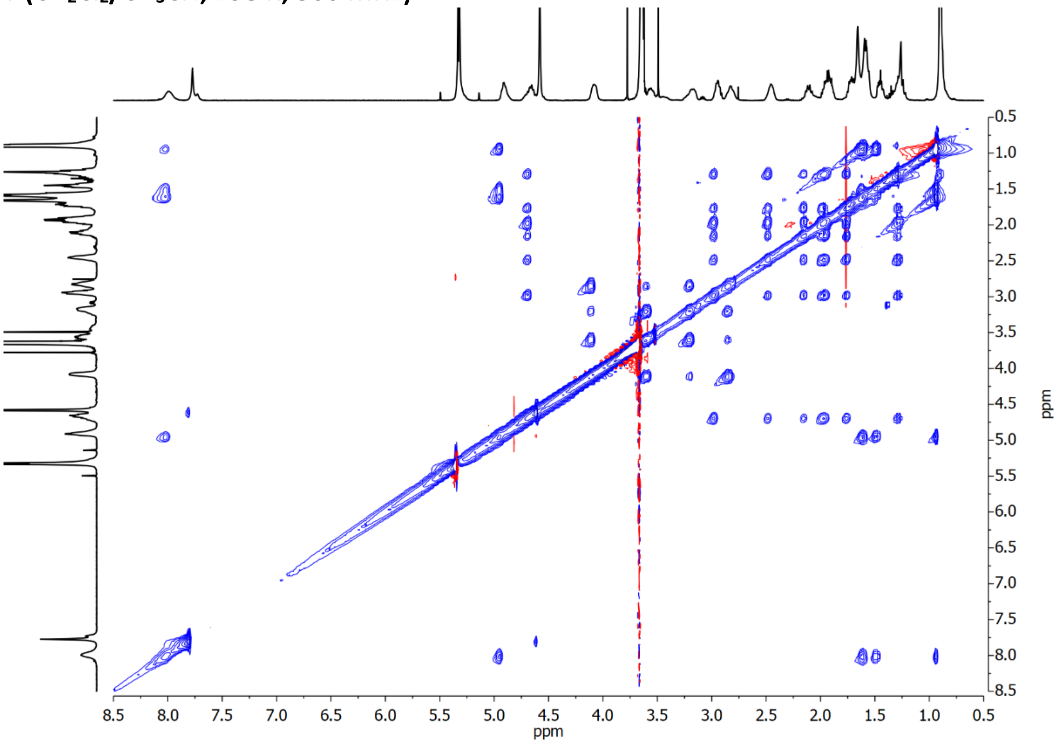

ROESY (CD_2Cl_2/CD_3CN, 298 K, 500 MHz)

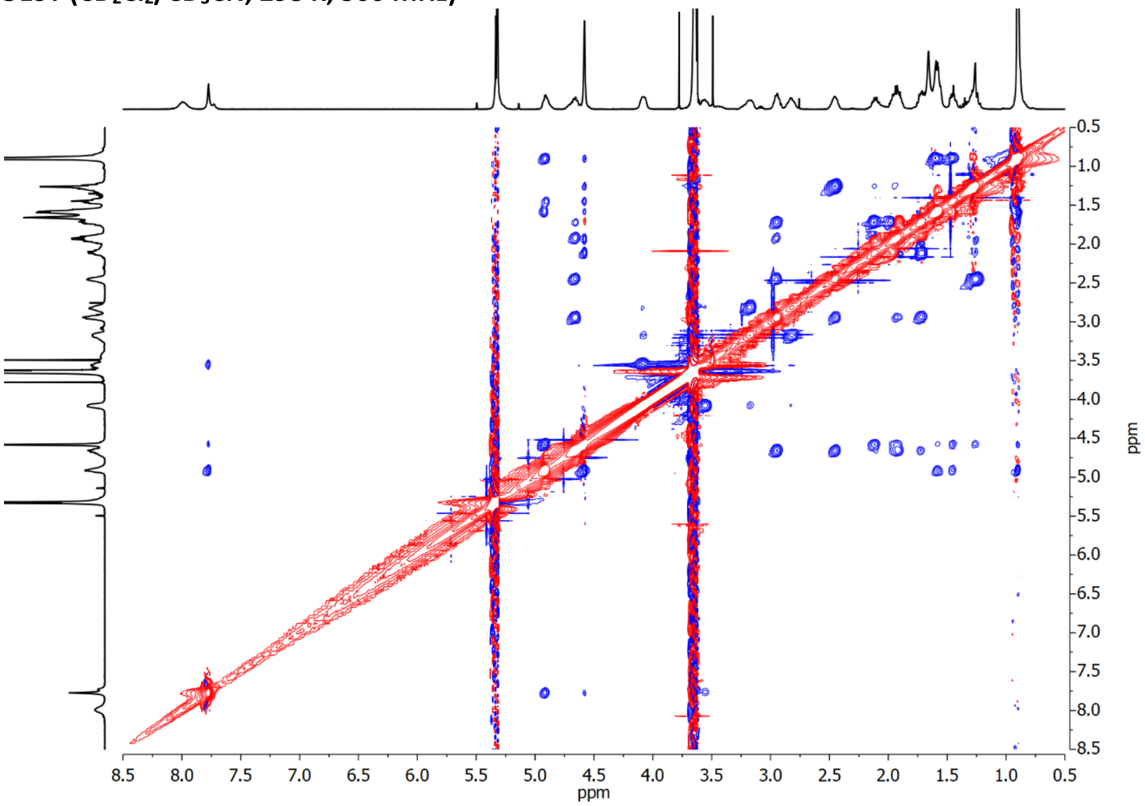

HSQC (CD_2Cl_2/CD_3CN, 298 K, 500 MHz)

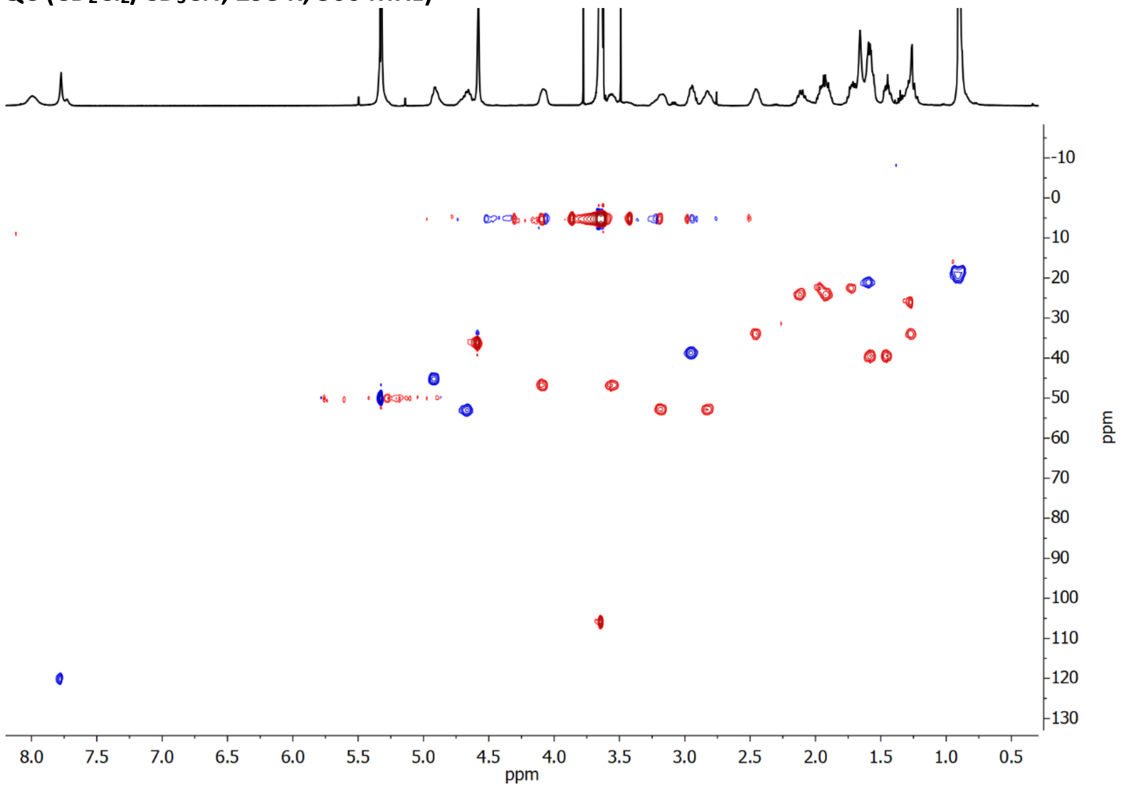

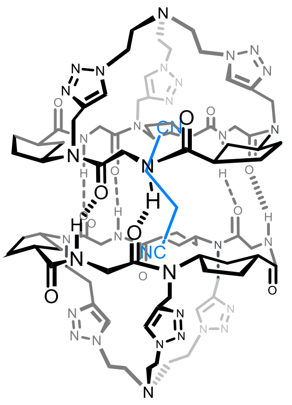

D2-succinonitrile

^{1}H-NMR (CD_2Cl_2/CD_3CN, 298 K, 500 MHz)

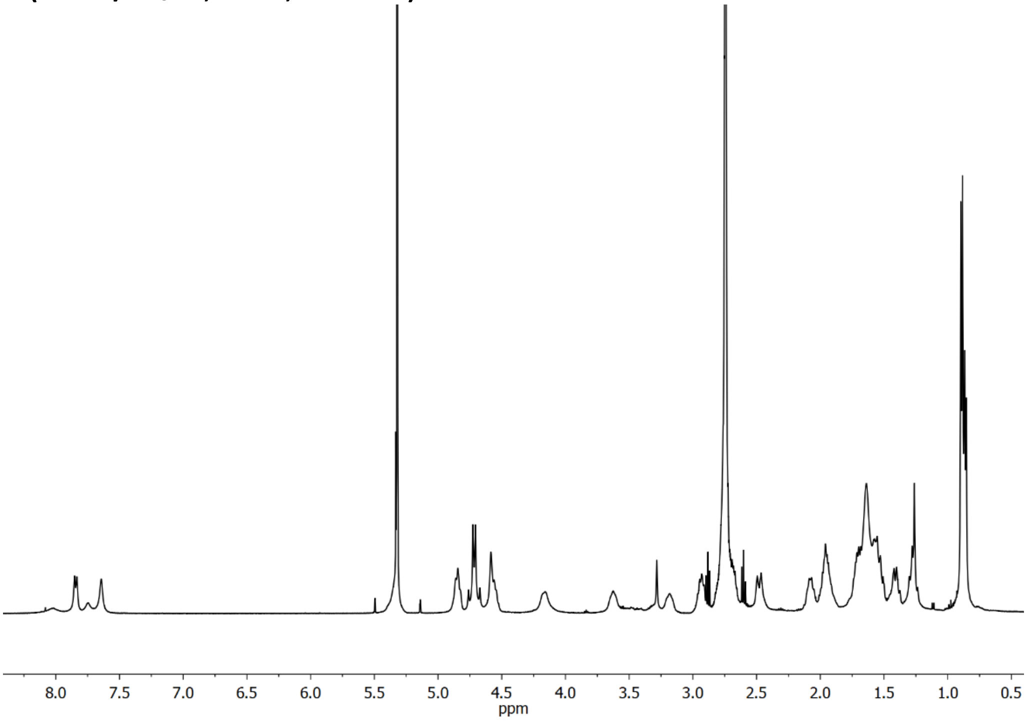

COSY (CD_2Cl_2/CD_3CN, 298 K, 500 MHz)

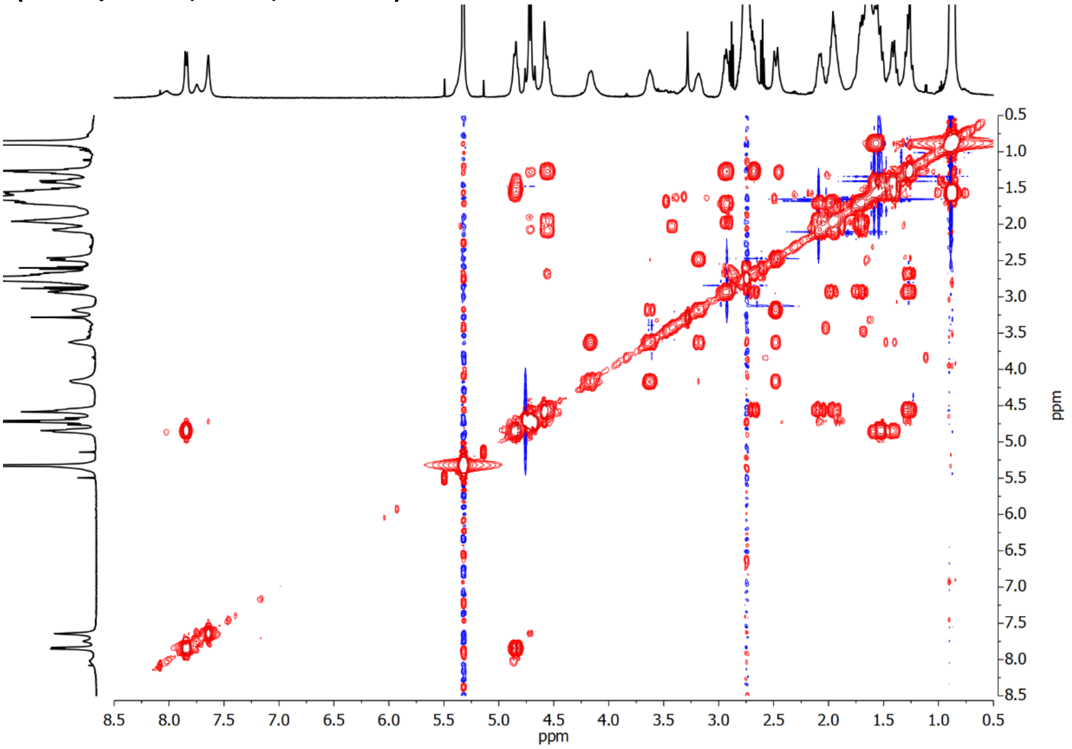

TOCSY (CD_2Cl_2/CD_3CN, 298 K, 500 MHz)

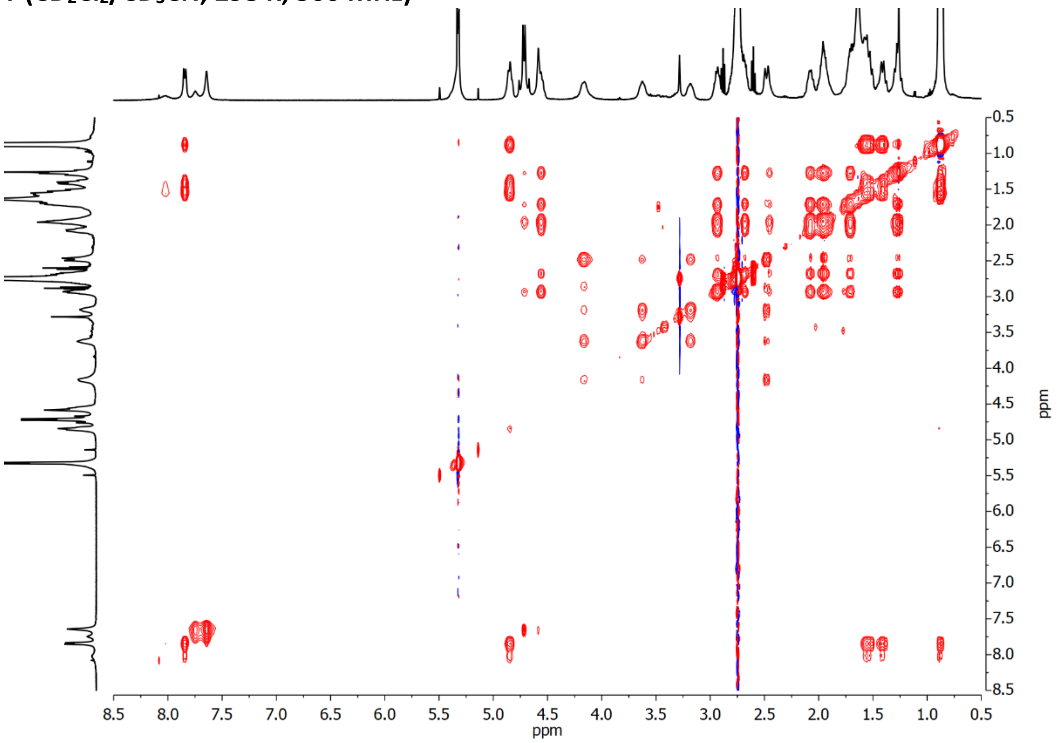

HSQC (CD_2Cl_2/CD_3CN, 298 K, 500 MHz)

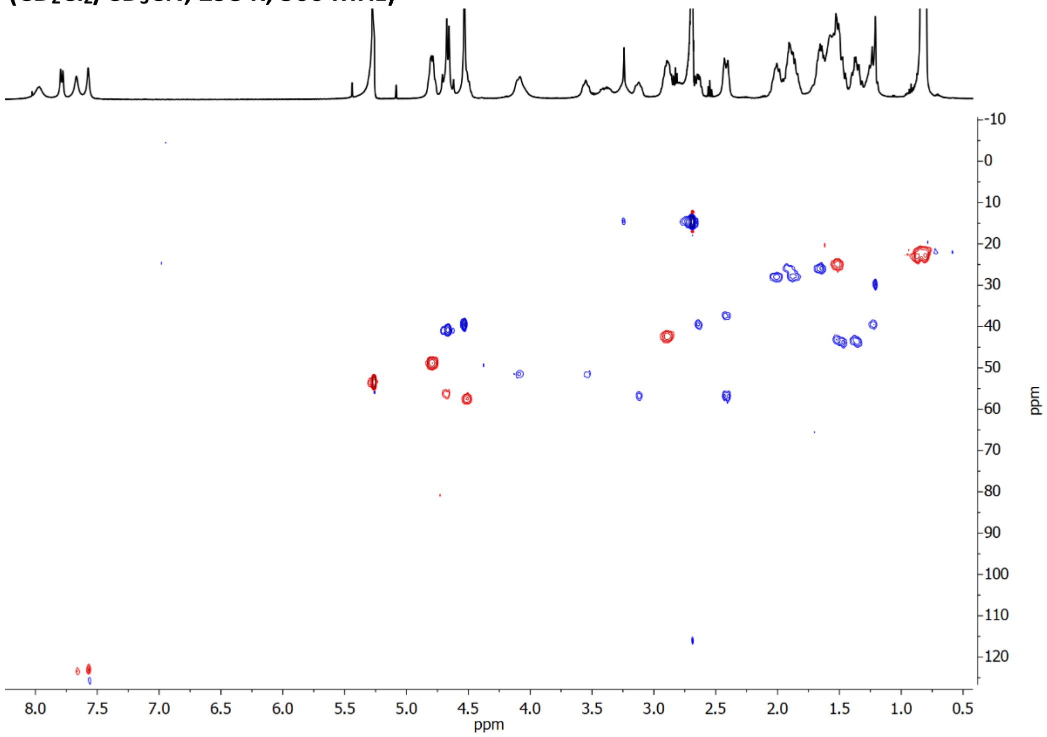

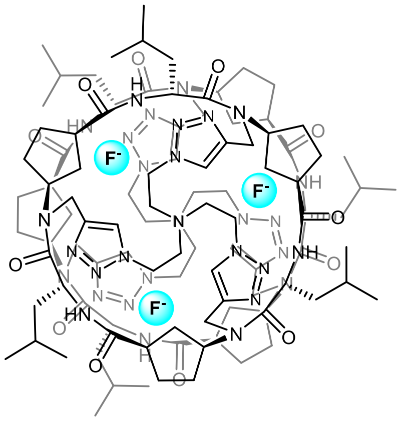

(mF⁻·nH₂O)⊂2CP2

IR

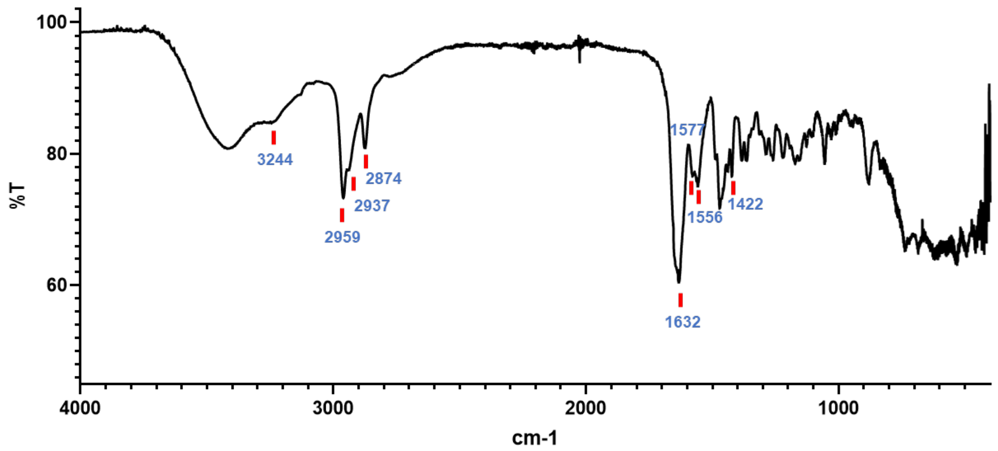

^{1}H-NMR (CD_2Cl_2/CD_3CN, 298 K, 500 MHz)

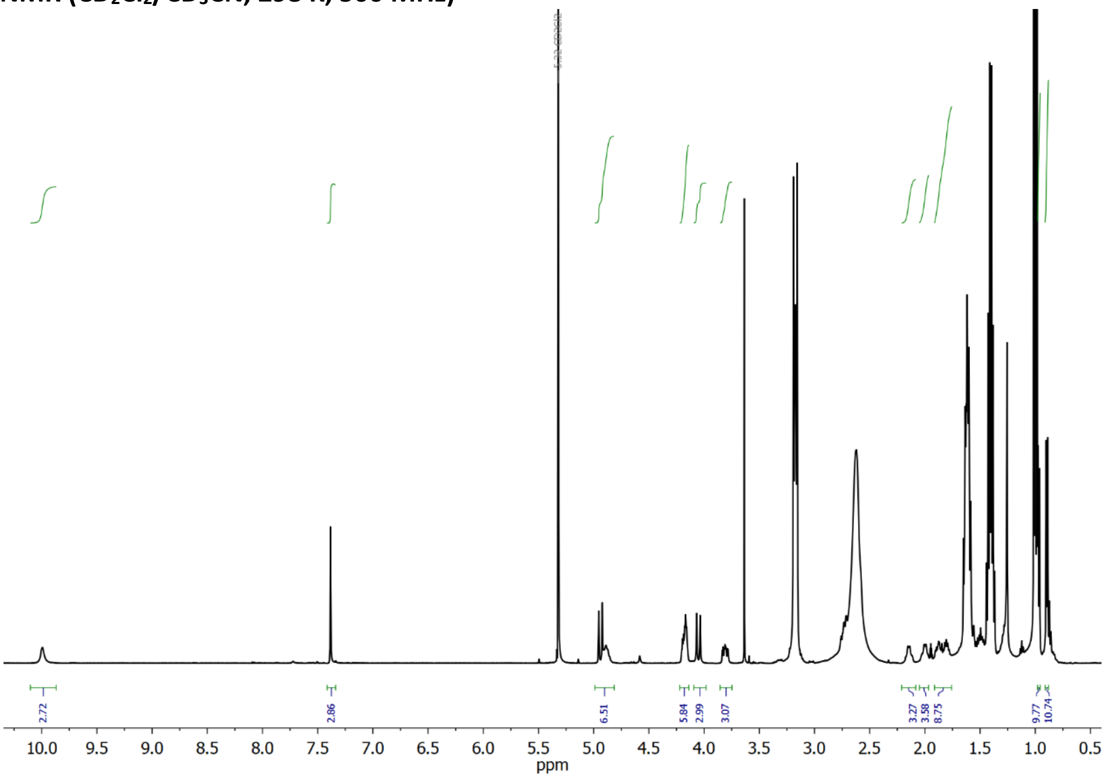

^{13}C-NMR (CD_2Cl_2 /CD_3CN, 298 K, 126 MHz)

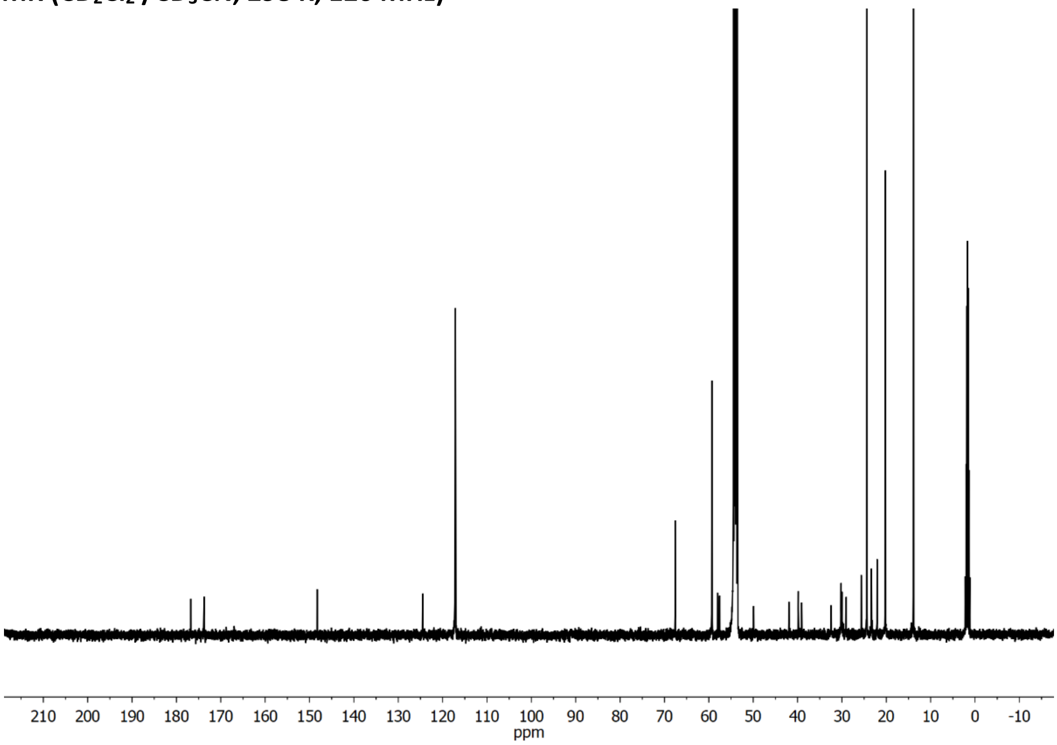

COSY (CD_2Cl_2/CD_3CN, 298 K, 500 MHz)

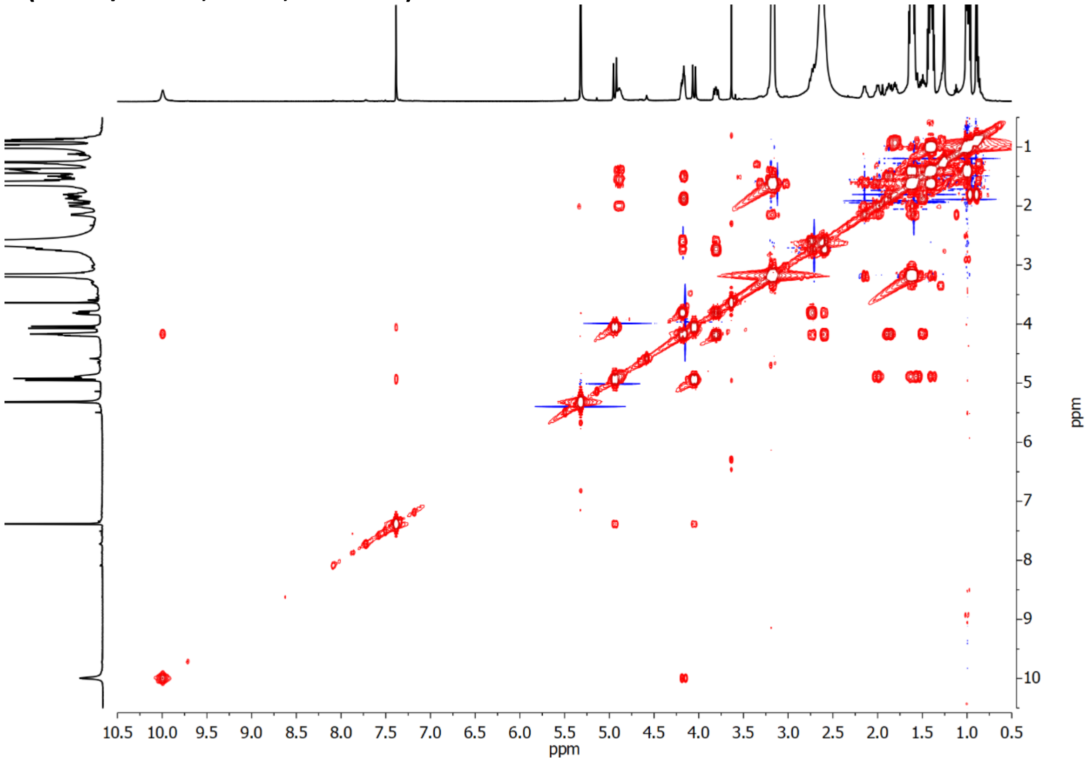

TOCSY (CD_2Cl_2/CD_3CN, 298 K, 500 MHz)

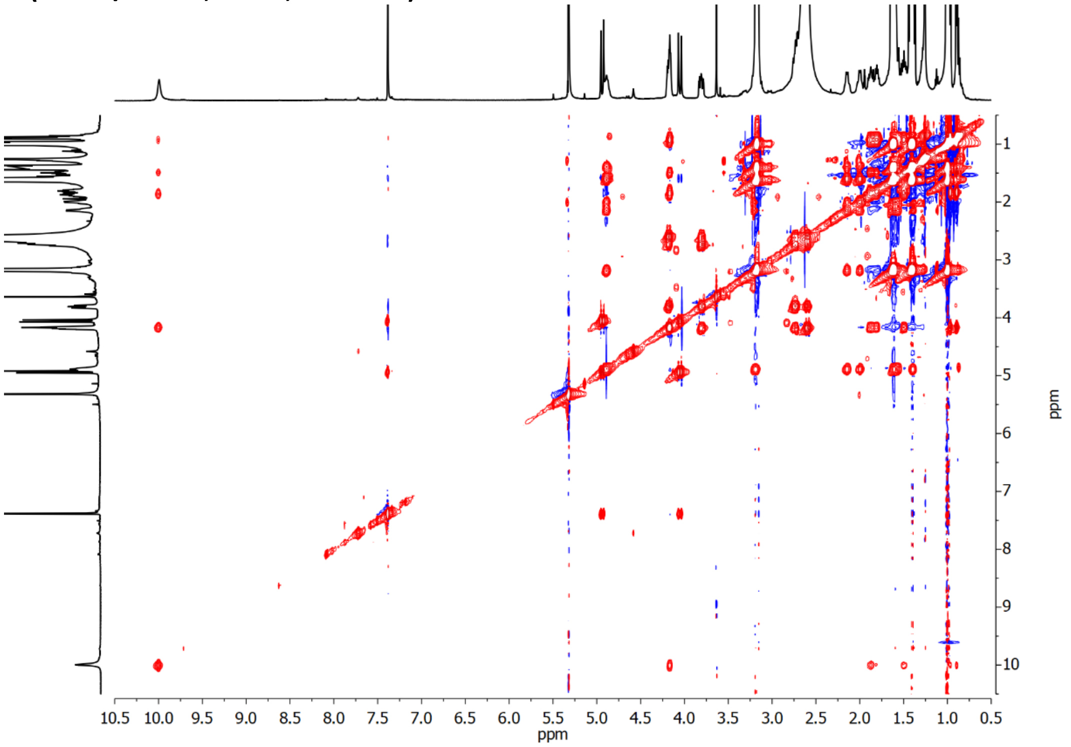

ROESY (CD_2Cl_2/CD_3CN, 298 K, 500 MHz)

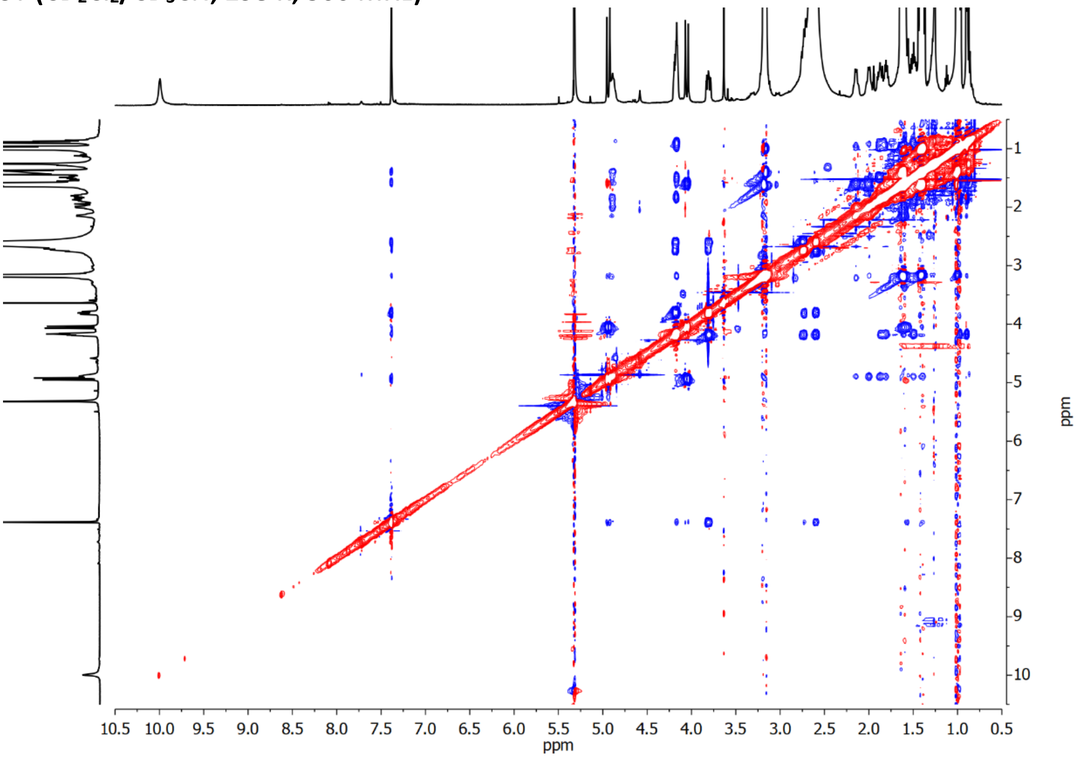

HSQC (CD_2Cl_2/CD_3CN, 298 K, 500 MHz)

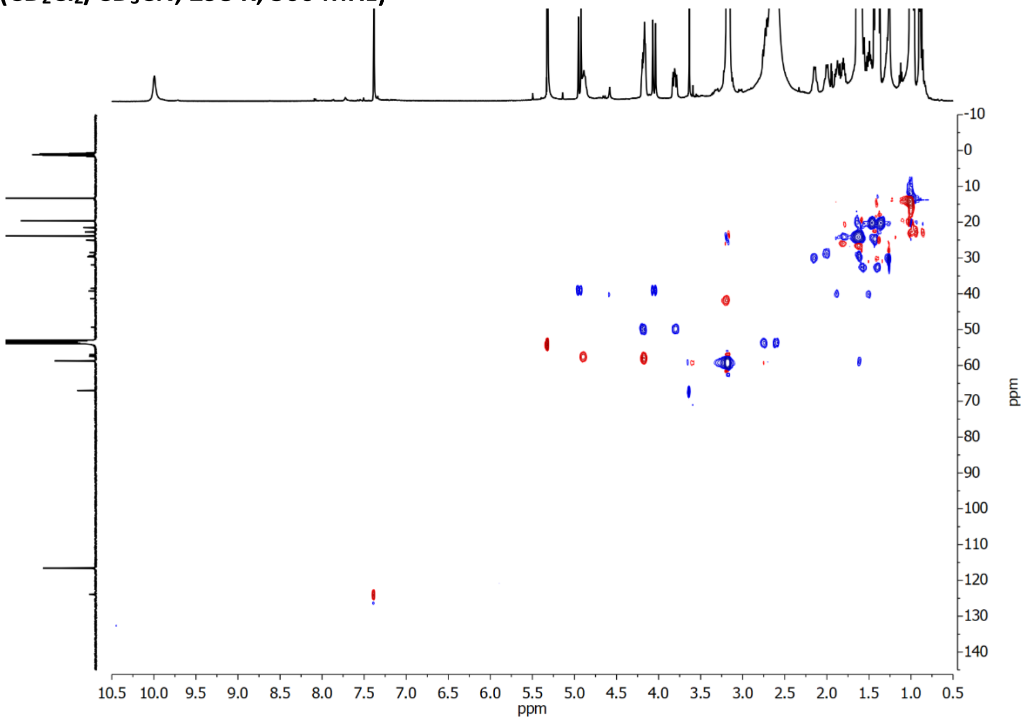

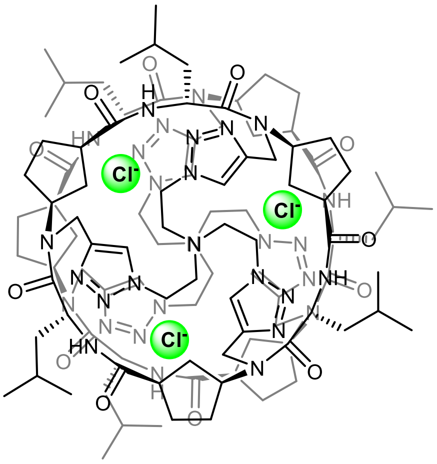

($mCl^{-}\cdot nH_2O$)⊂2CP2

^{1}H-NMR (CD_2Cl_2, 298 K, 500 MHz) of D2 + 24.7 equiv. TBACl

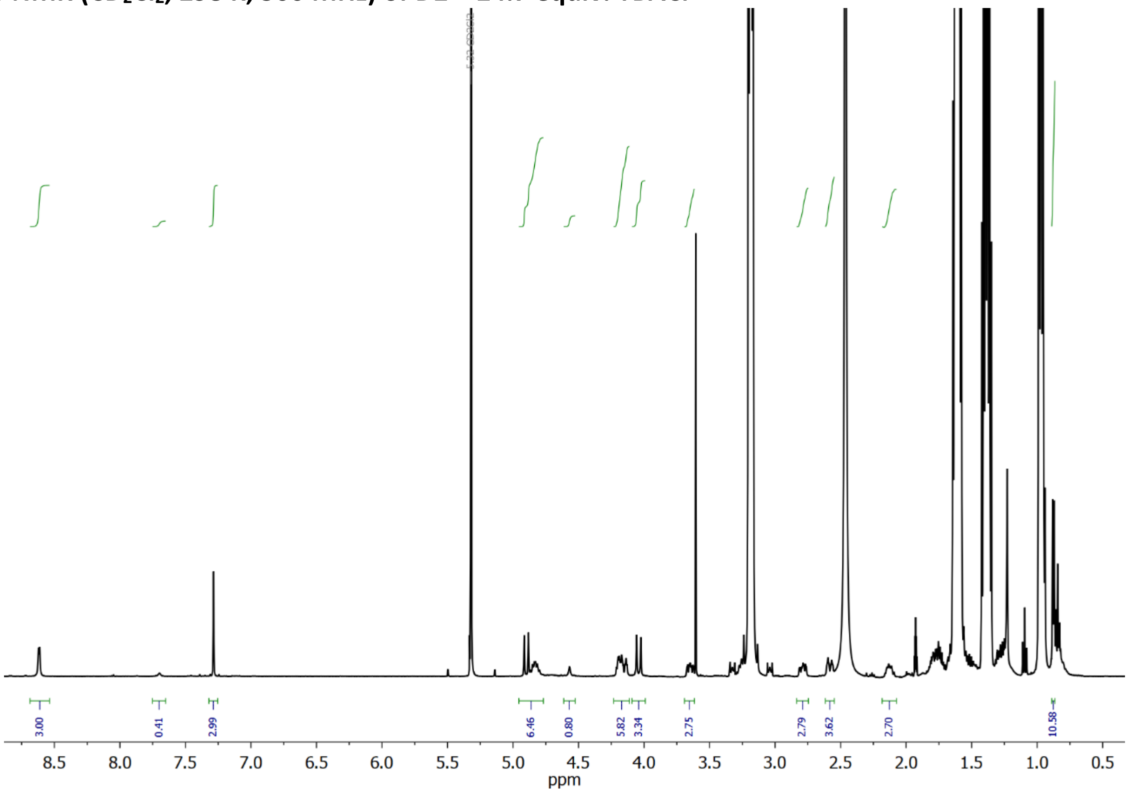

(mBr⁻·nH$_2$O)⊂2CP2

^{1}H-NMR (CD$_2$Cl$_2$, 298 K, 500 MHz) of D2 + 28.1 equiv. TBAB

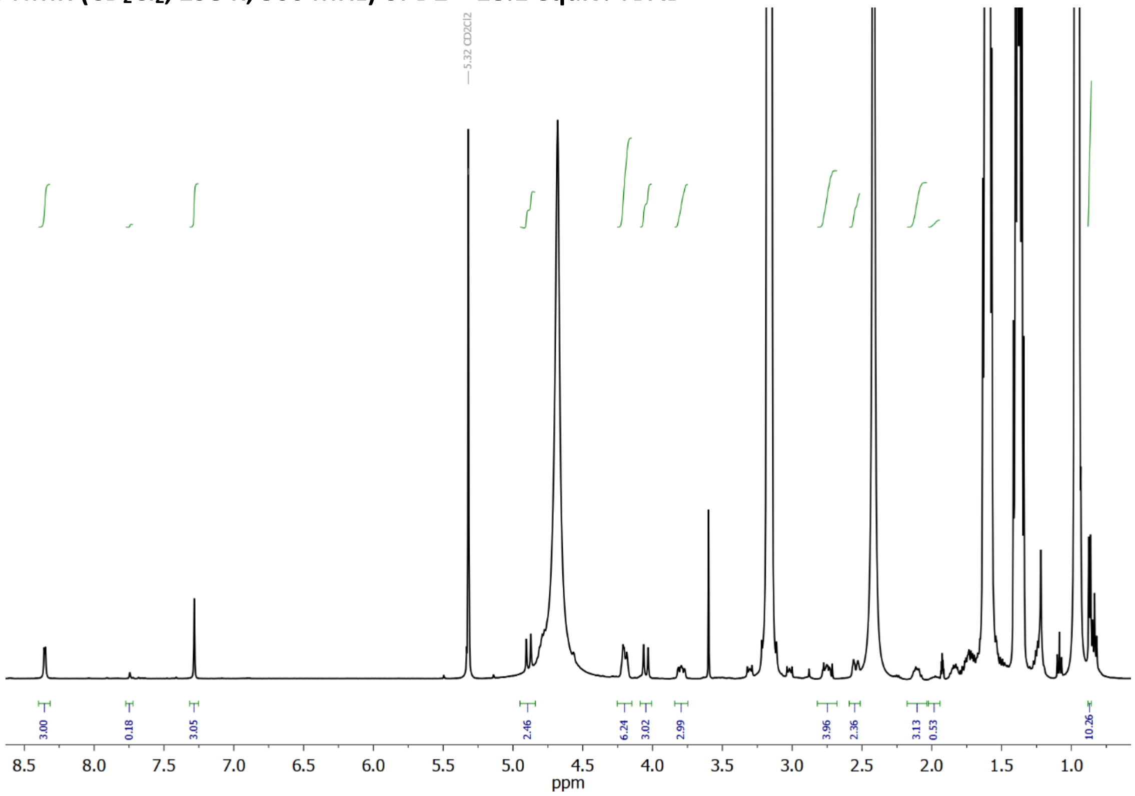

(mI⁻·nH$_2$O)⊂2CP2

^{1}H-NMR (CD$_2$Cl$_2$, 298 K, 500 MHz) of D2 + 86 equiv. TBAI

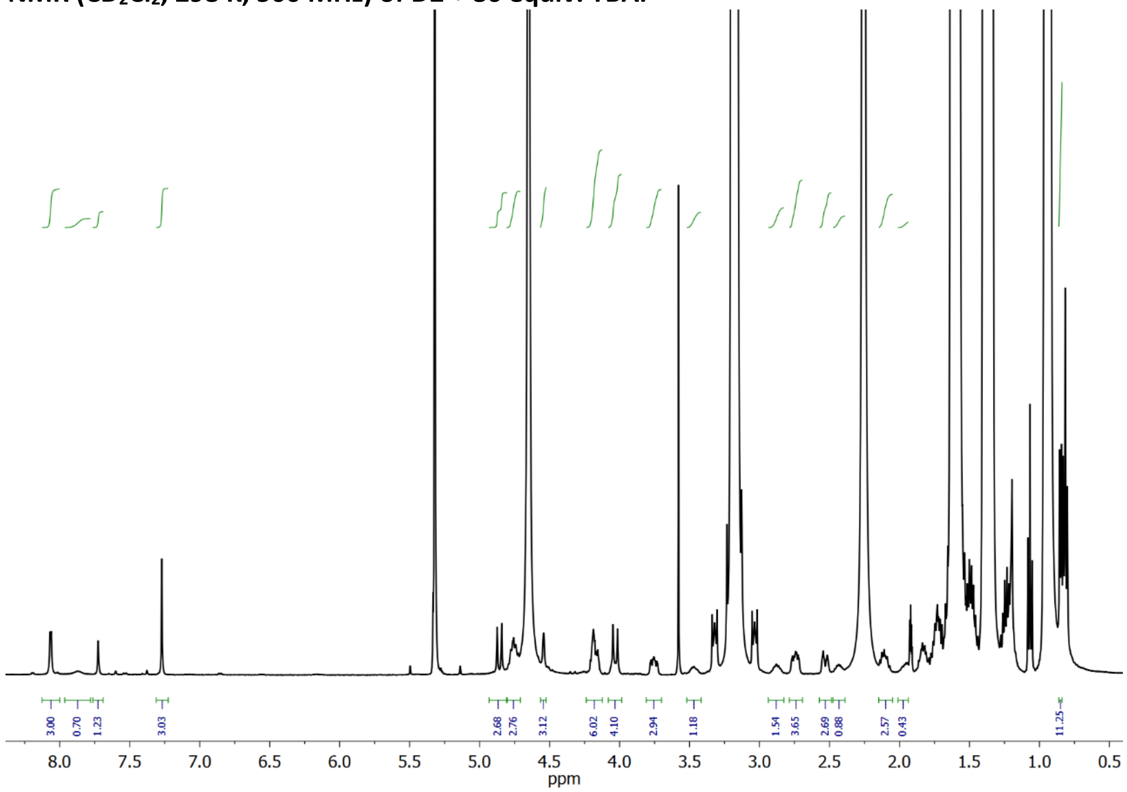

(mNO$_3^-$·nH$_2$O)⊂2CP2

^{1}H-NMR (CD_2Cl_2, 298 K, 500 MHz) of D2 + 60 equiv. TBAN

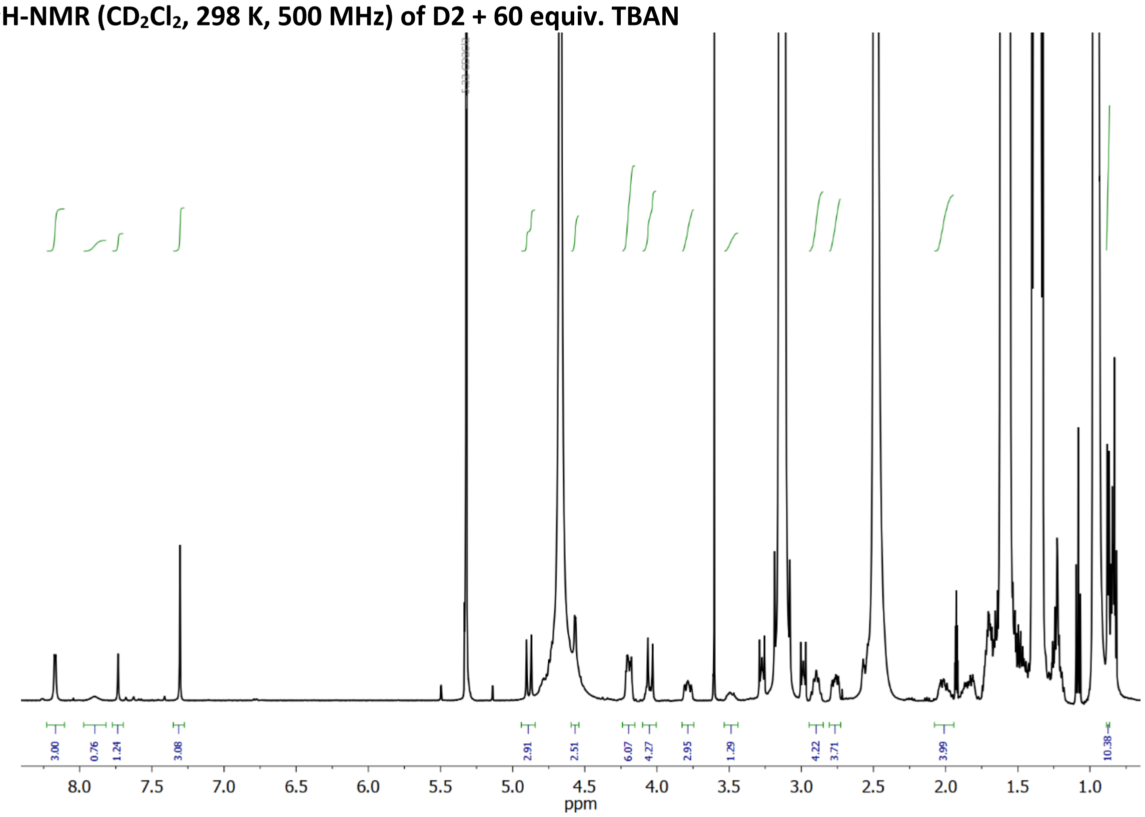

(mAcO$^-$·nH$_2$O)⊂2CP2

^{1}H-NMR (CD_2Cl_2, 298 K, 500 MHz) of D2 + 66 equiv. TBAAc

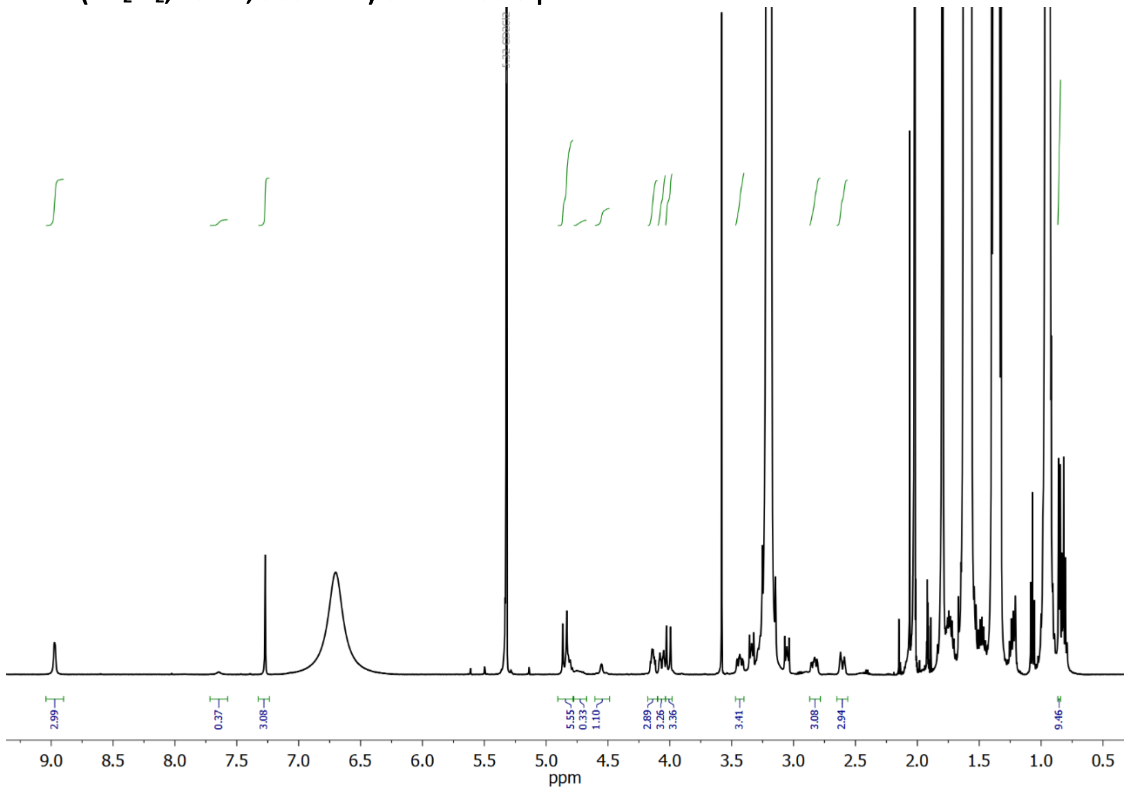

($mN_3^{-}\cdot nH_2O$)⊂2CP2

^{1}H-NMR (CD_2Cl_2, 298 K, 500 MHz) of D2 + 35 equiv. $TBAN_3$

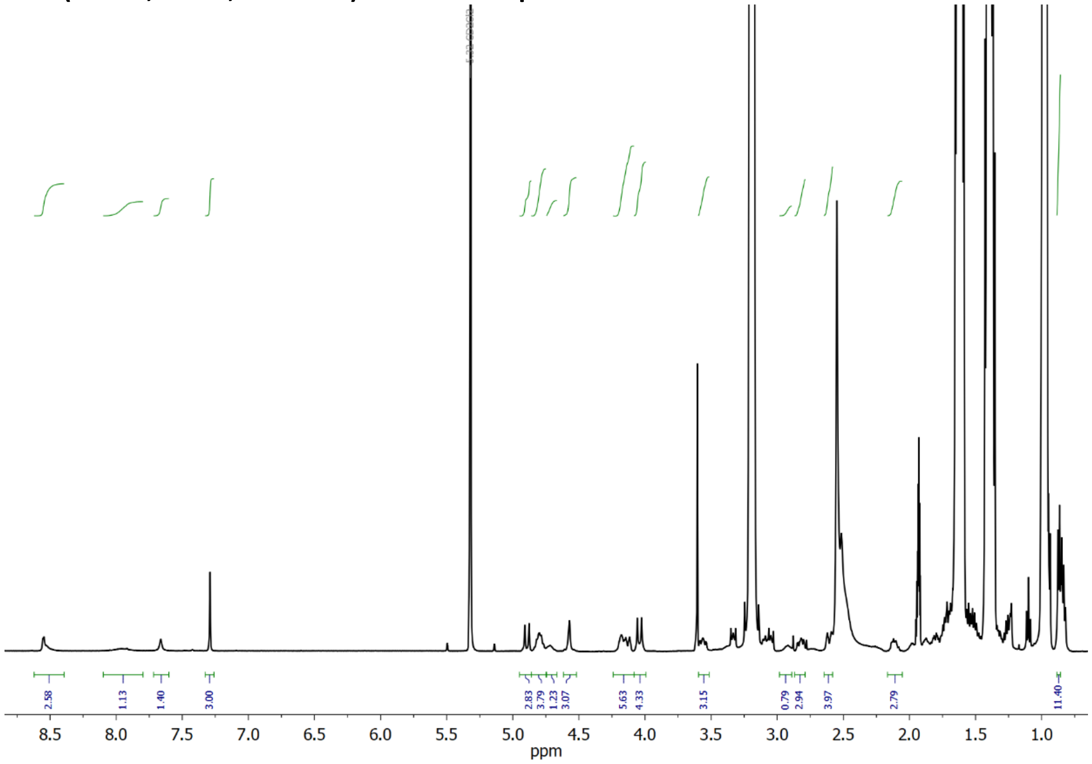

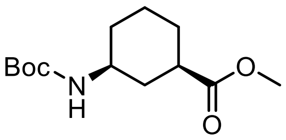

2b

^{1}H-NMR (CDCl$_3$, 298 K, 500 MHz)

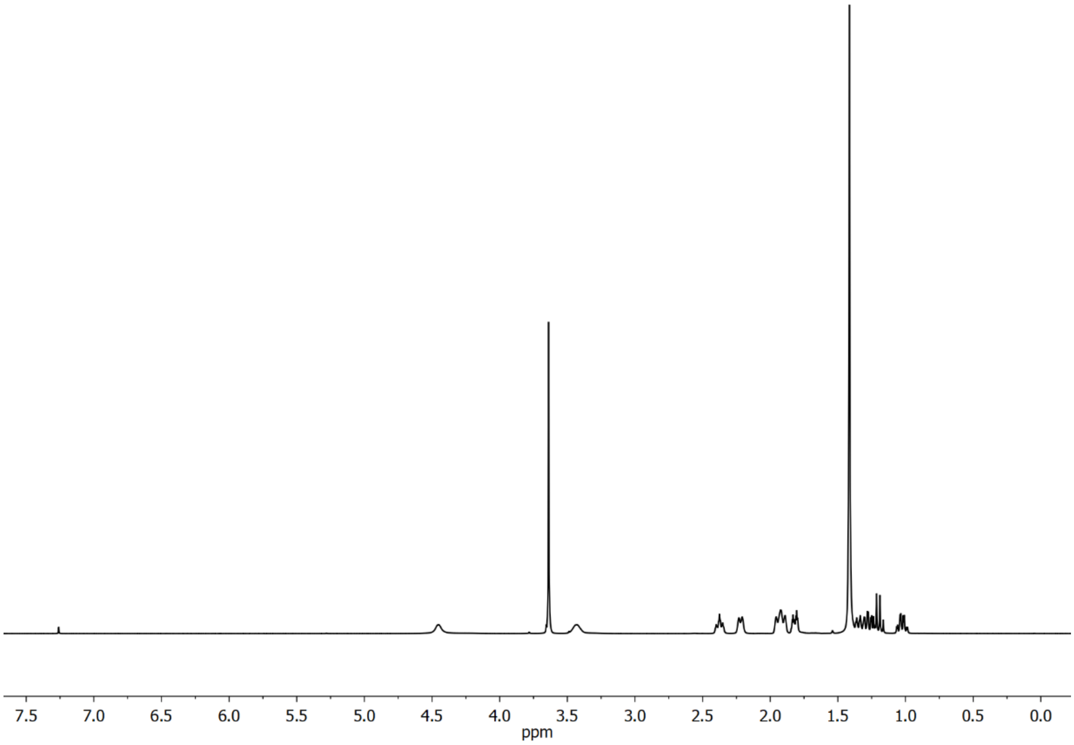

^{13}C and DEPT NMR (CDCl$_3$, 298 K, 126 MHz)

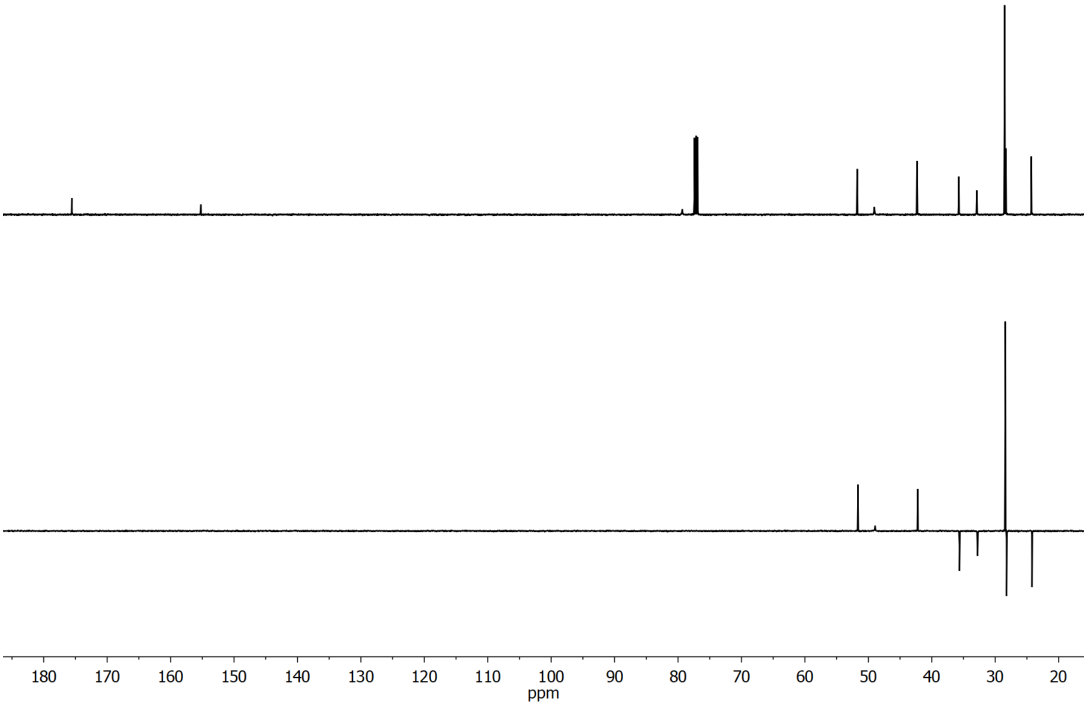

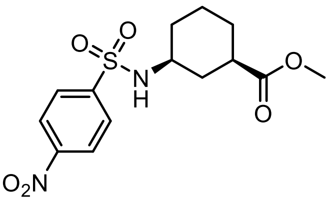

2c

^{1}H-NMR (CDCl$_3$, 298 K, 500 MHz)

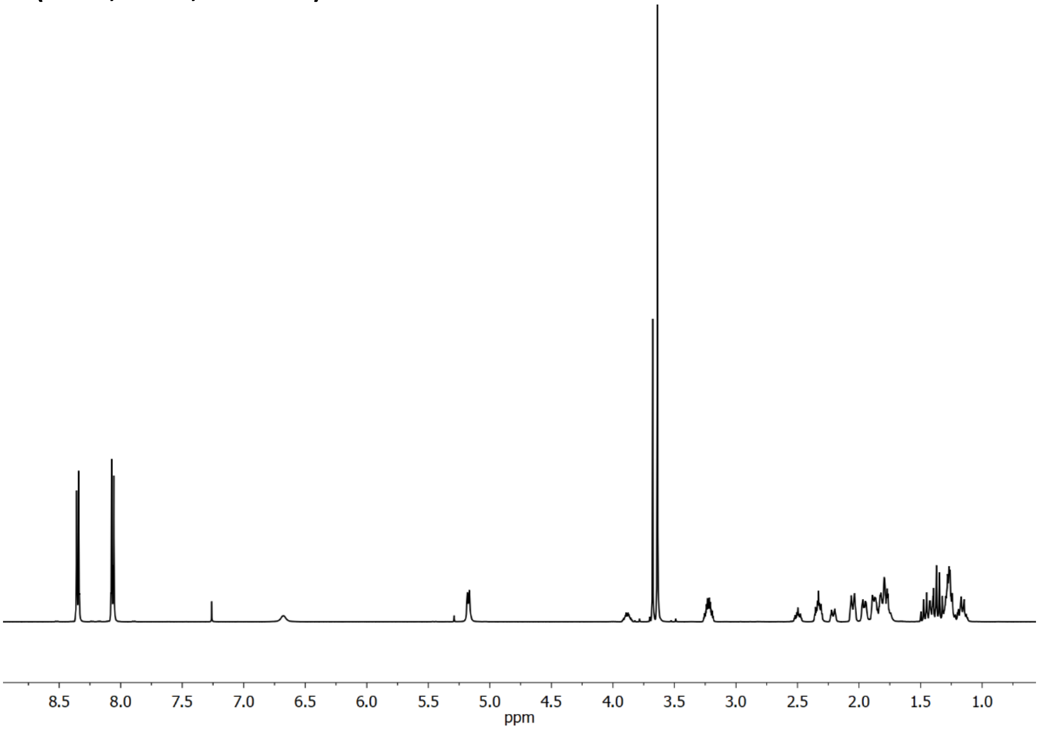

^{13}C and DEPT NMR (CDCl$_3$, 298 K, 126 MHz)

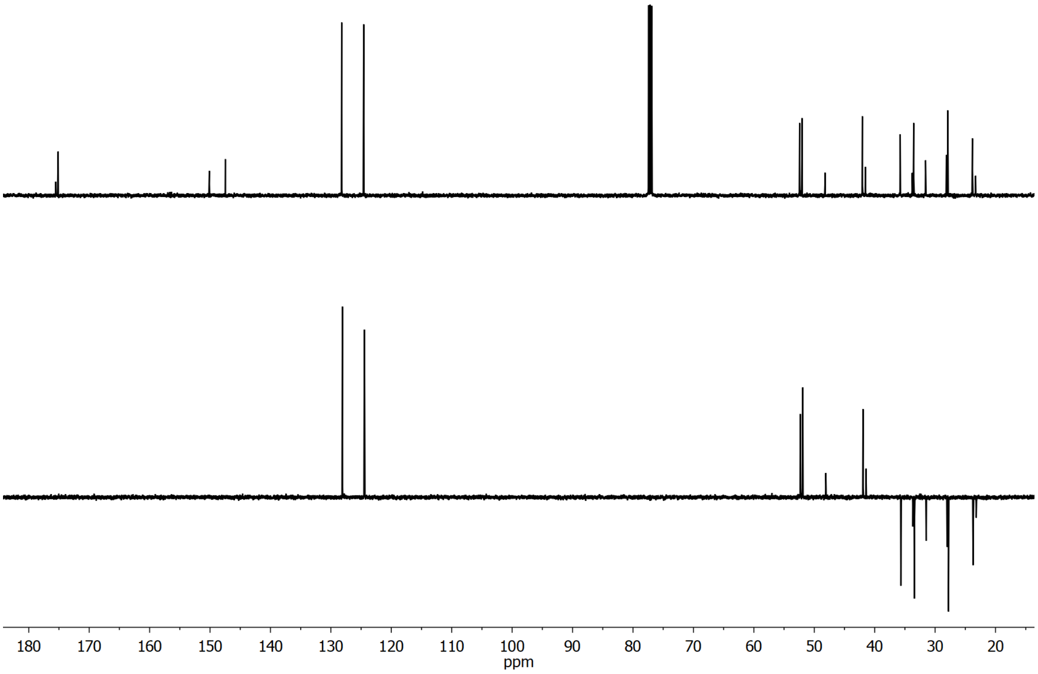

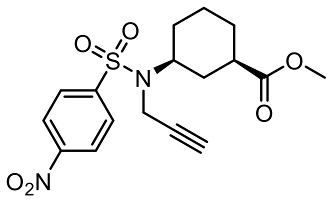

2d

^{1}H-NMR ($CDCl_3$, 298 K, 500 MHz)

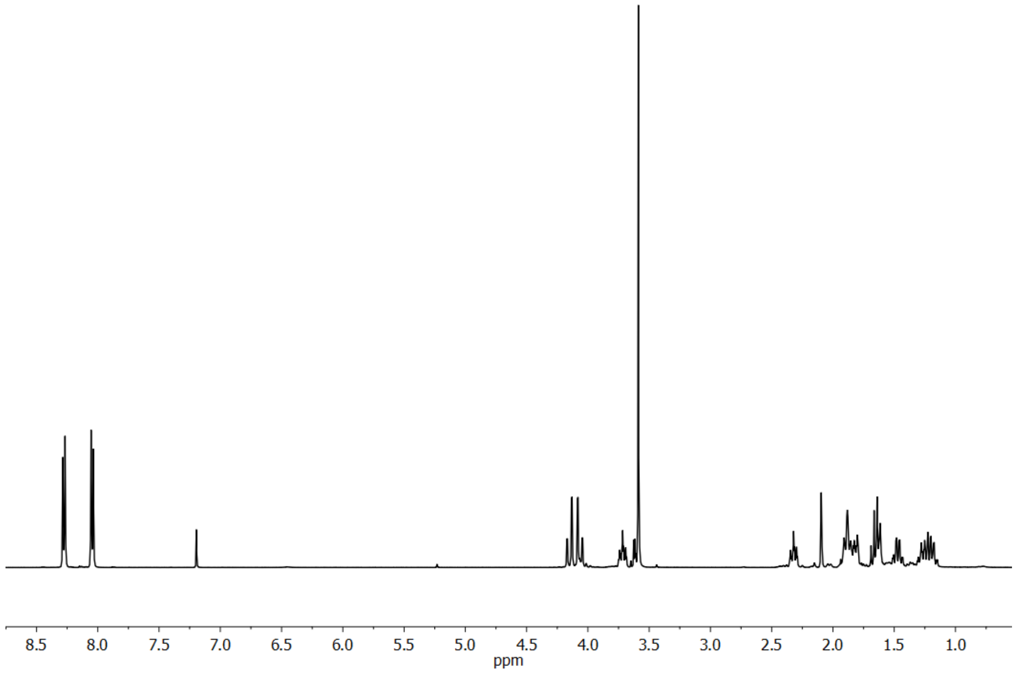

^{13}C and DEPT NMR ($CDCl_3$, 298 K, 126 MHz)

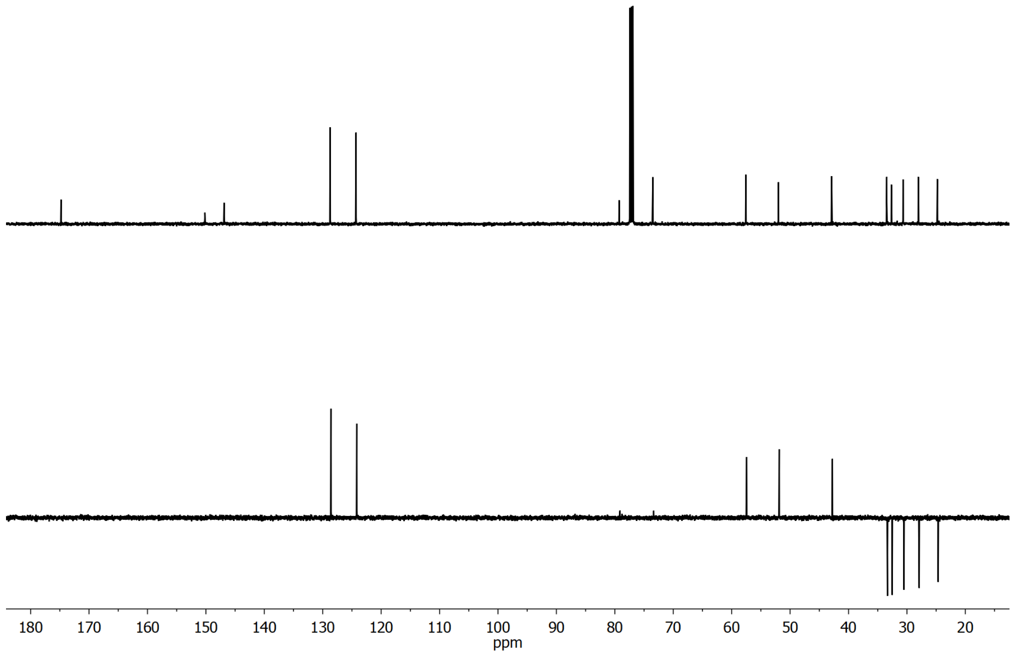

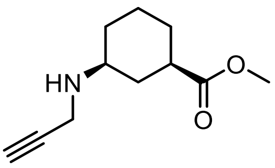

2e

^{1}H-NMR ($CDCl_3$, 298 K, 500 MHz)

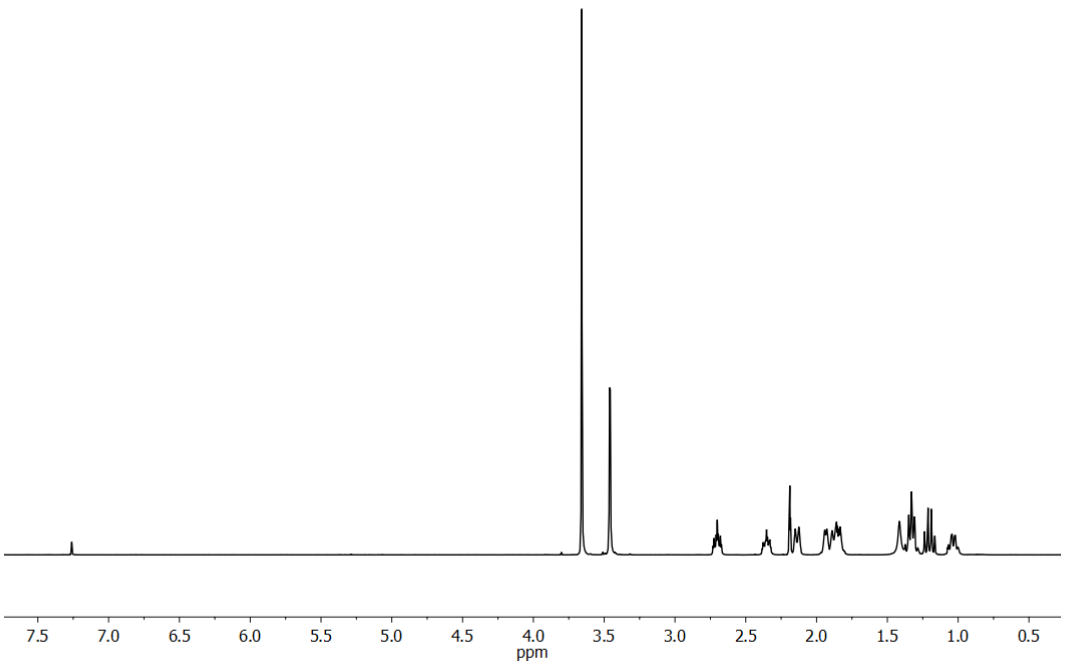

^{13}C and DEPT NMR ($CDCl_3$, 298 K, 126 MHz)

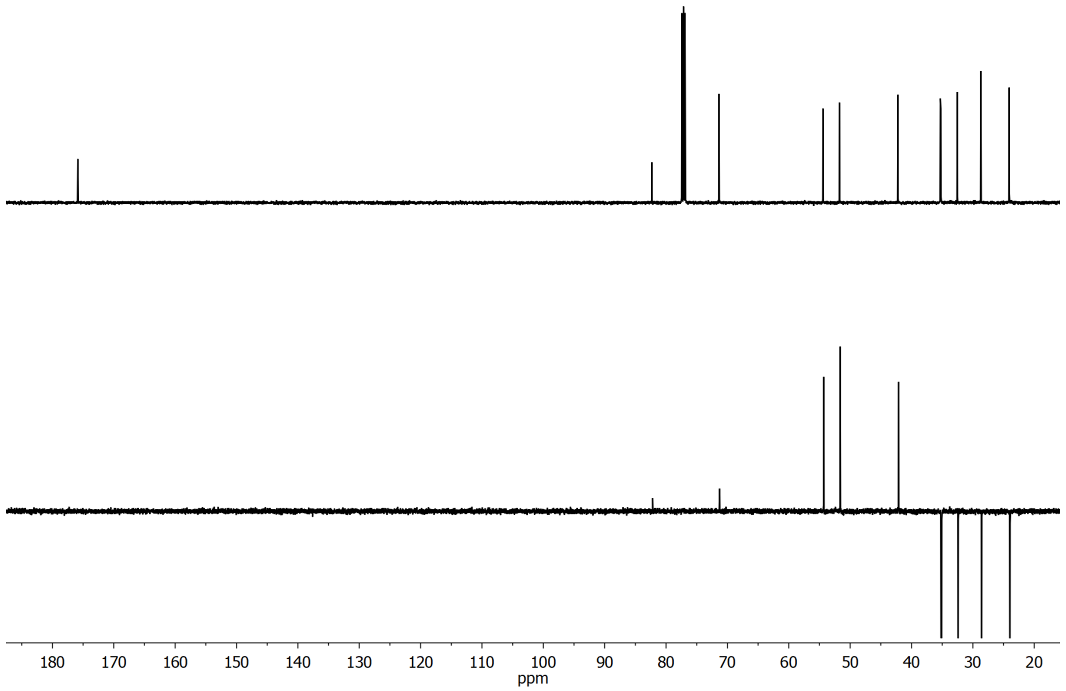

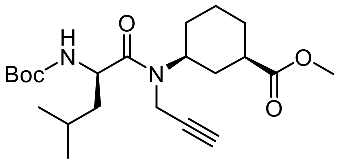

2f

^{1}H-NMR ($CDCl_3$, 298 K, 500 MHz)

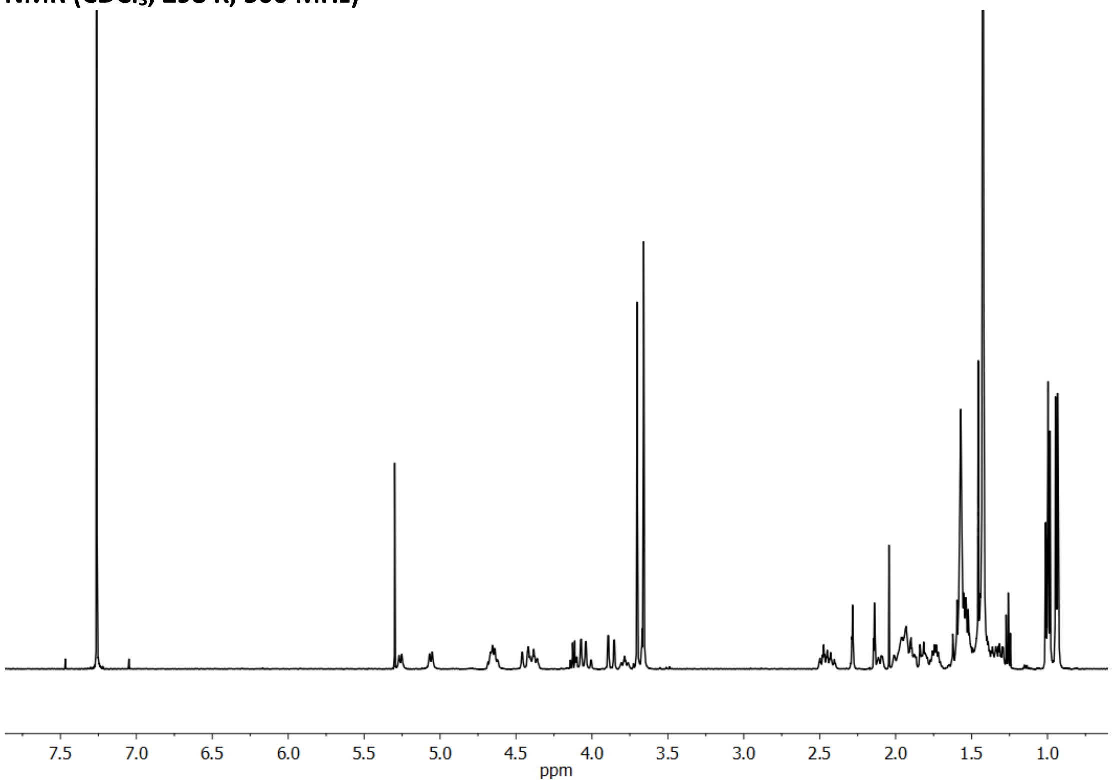

^{13}C and DEPT NMR ($CDCl_3$, 298 K, 126 MHz)

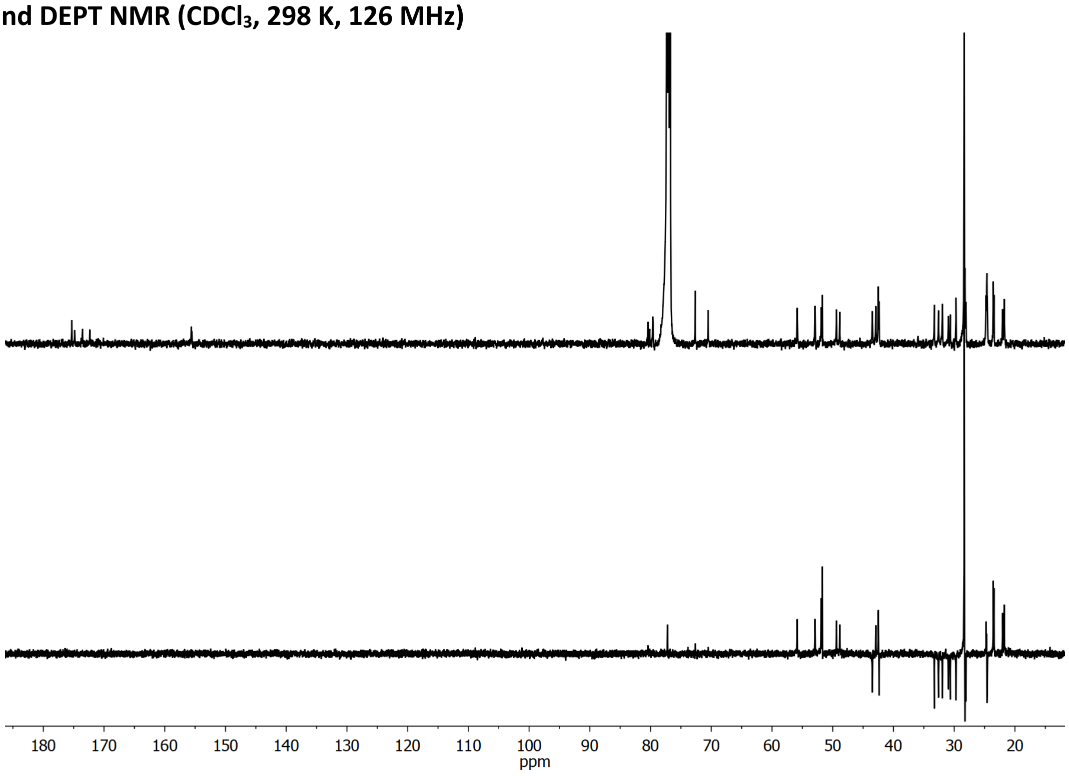

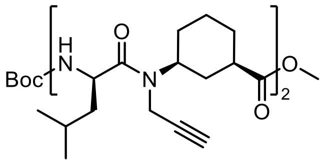

2g

^{1}H-NMR ($CDCl_3$, 298 K, 500 MHz)

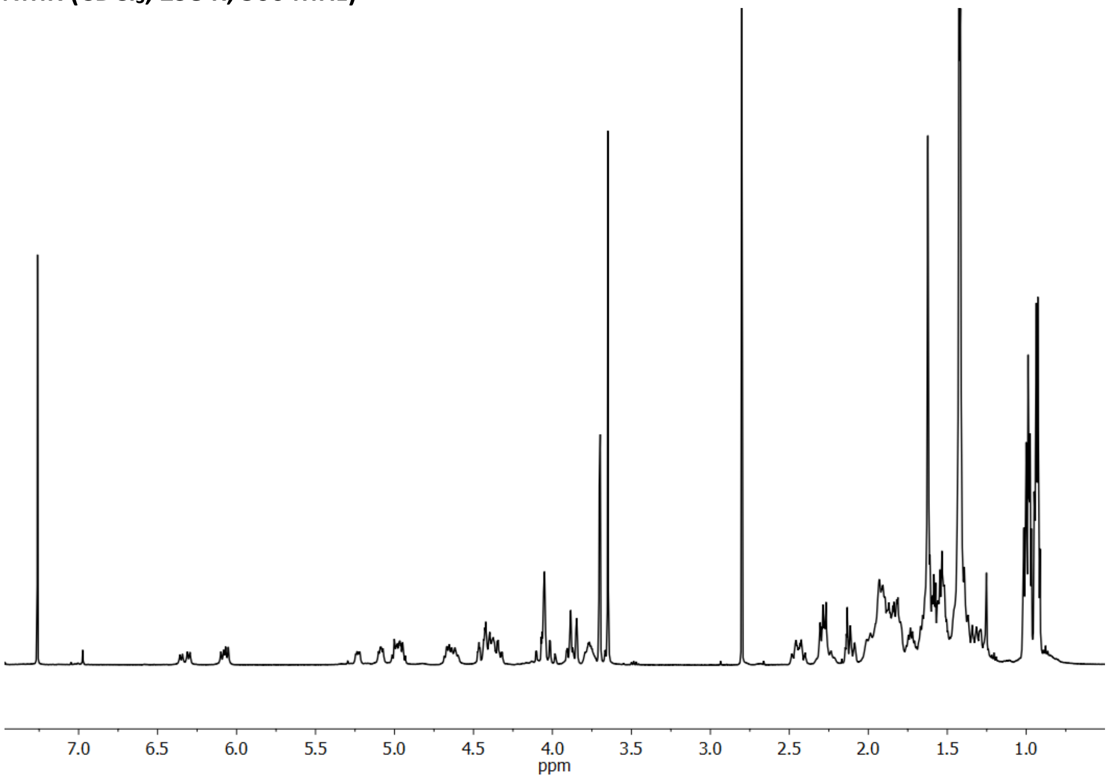

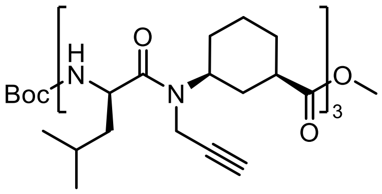

2h

^{1}H-NMR (CDCl$_3$, 298 K, 500 MHz)

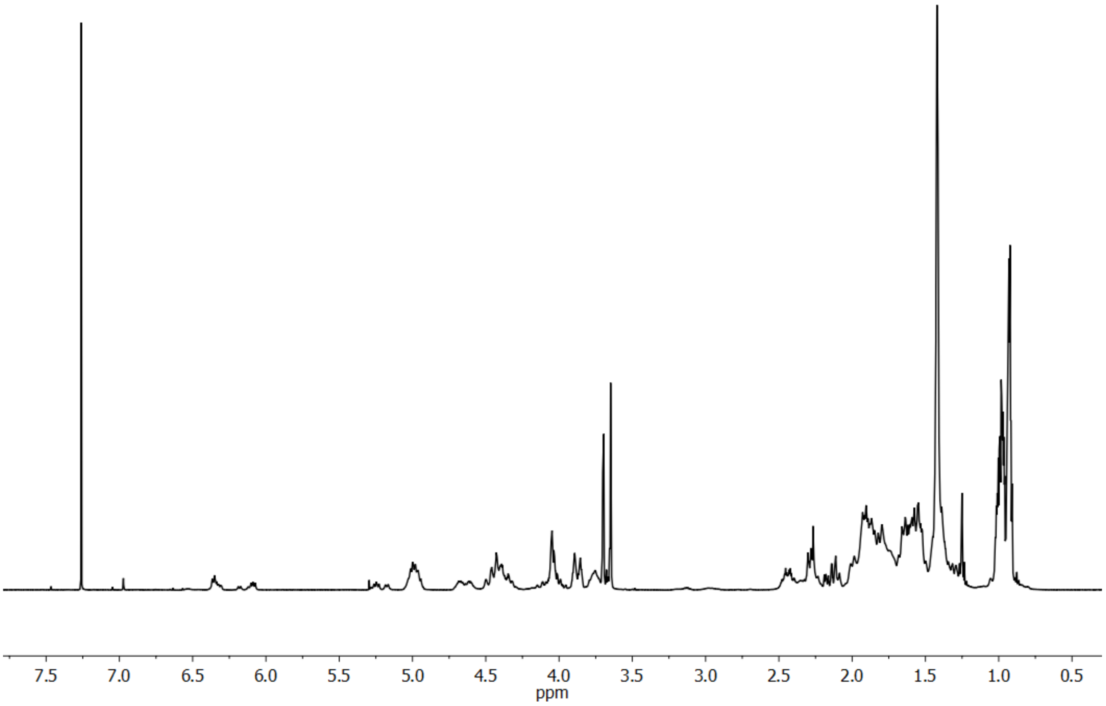

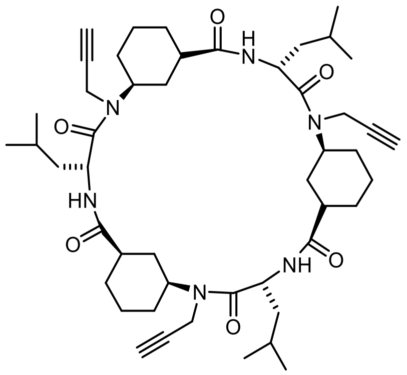

CP3

IR

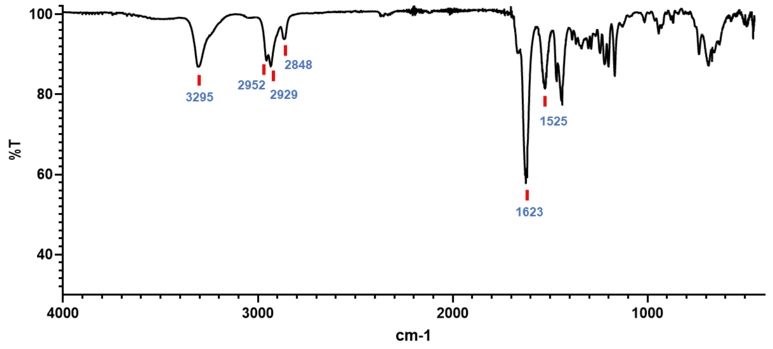

^{1}H-NMR ($CDCl_3$, 298 K, 500 MHz)

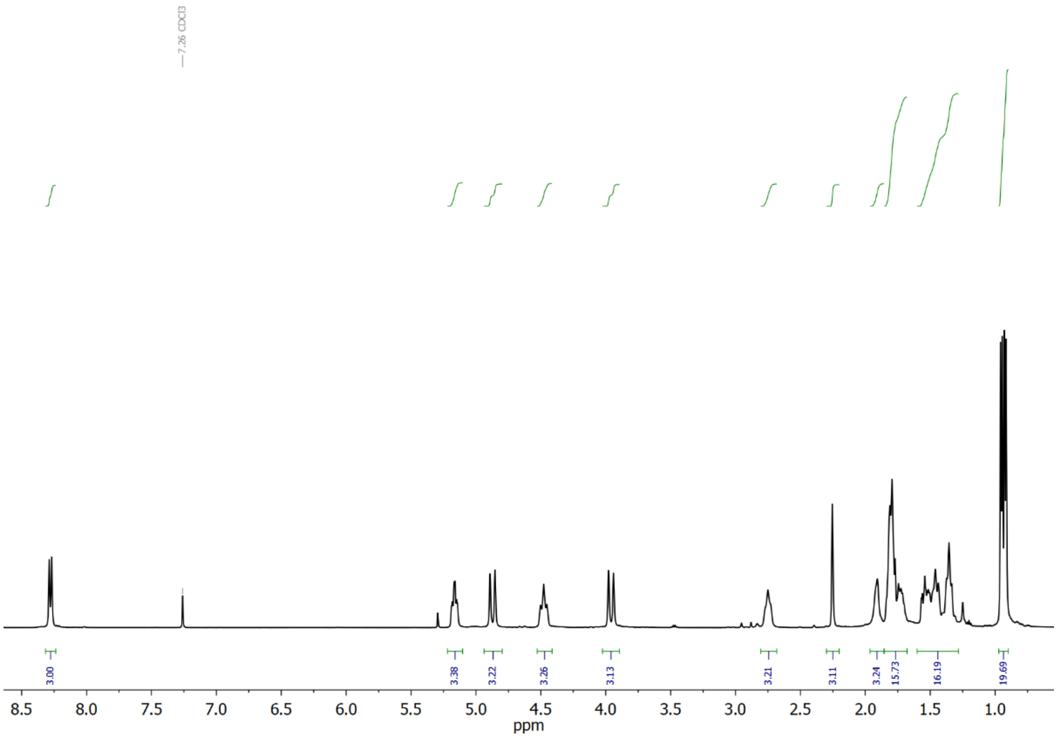

^{13}C and DEPT NMR (CD_2Cl_2, 298 K, 126 MHz)

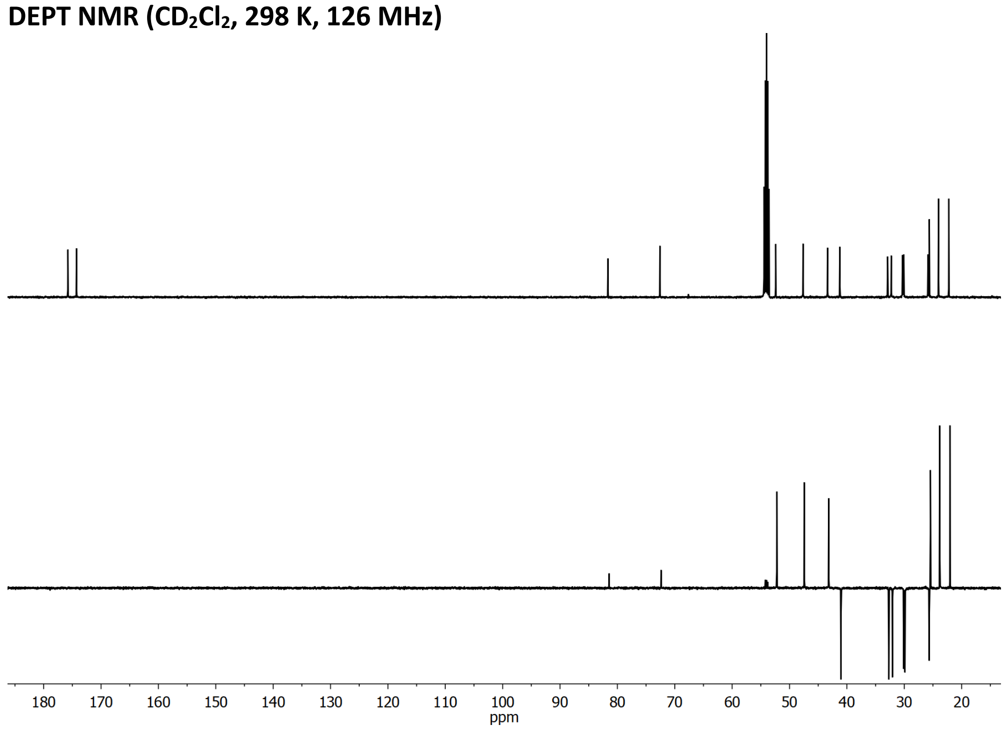

COSY ($CDCl_3$, 298 K, 500 MHz)

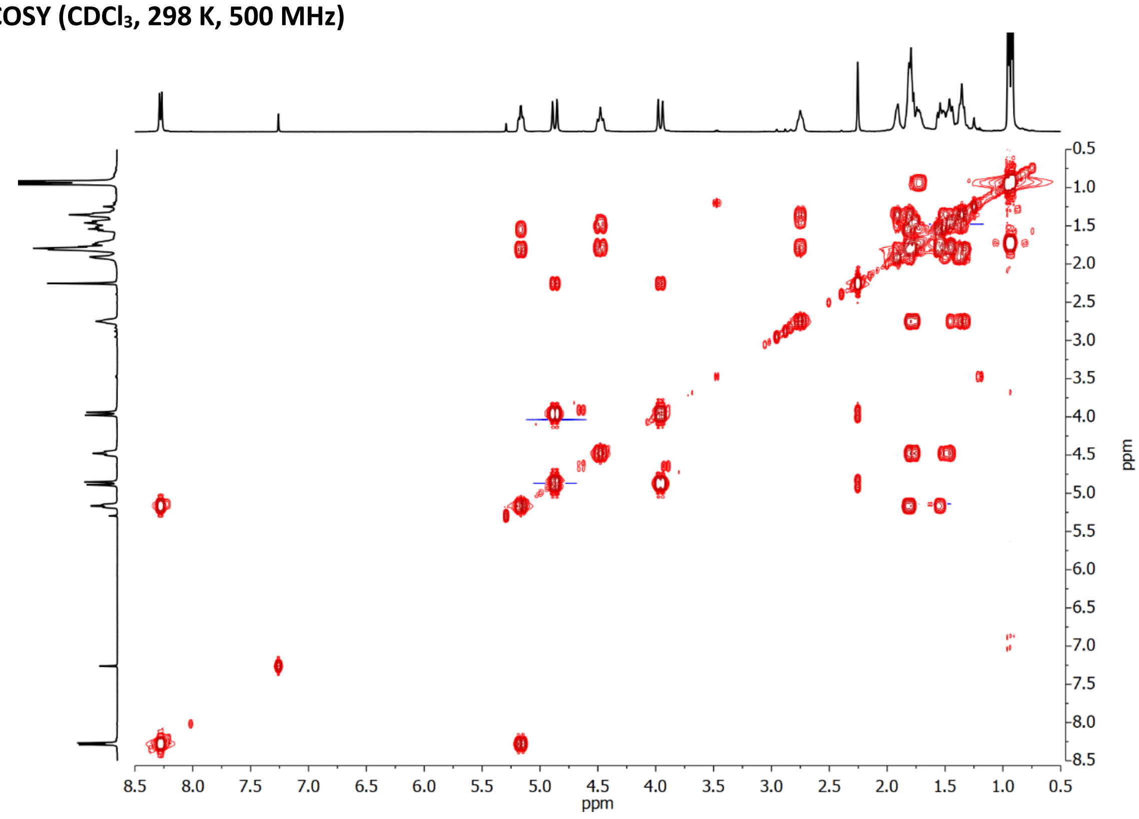

TOCSY ($CDCl_3$, 298 K, 500 MHz)

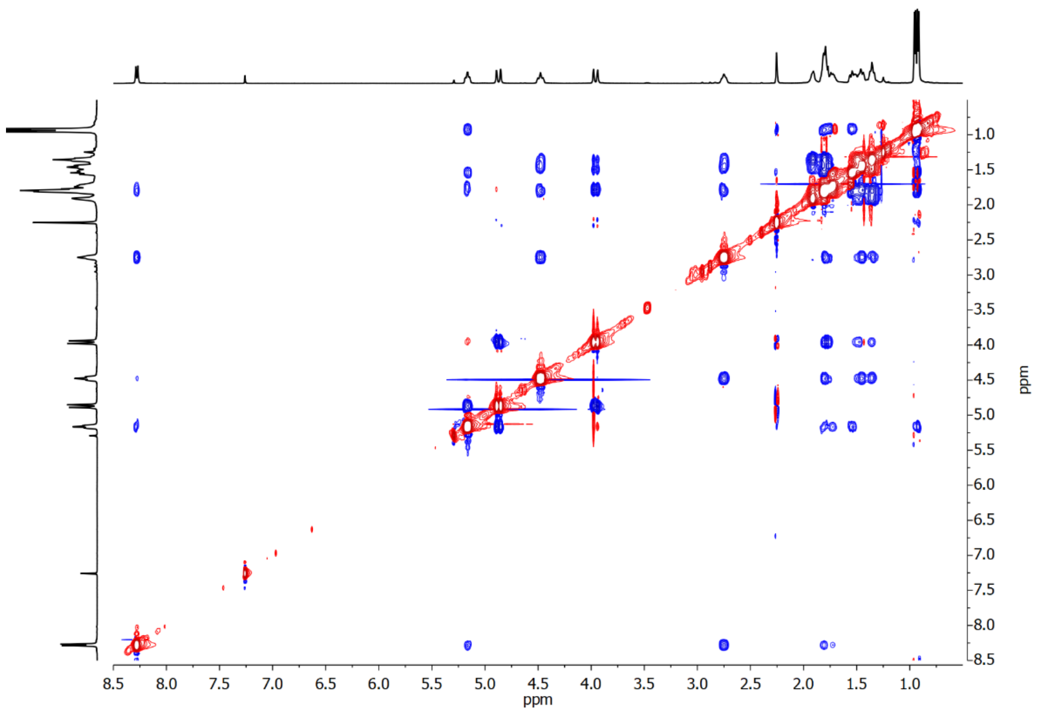

ROESY ($CDCl_3$, 298 K, 500 MHz)

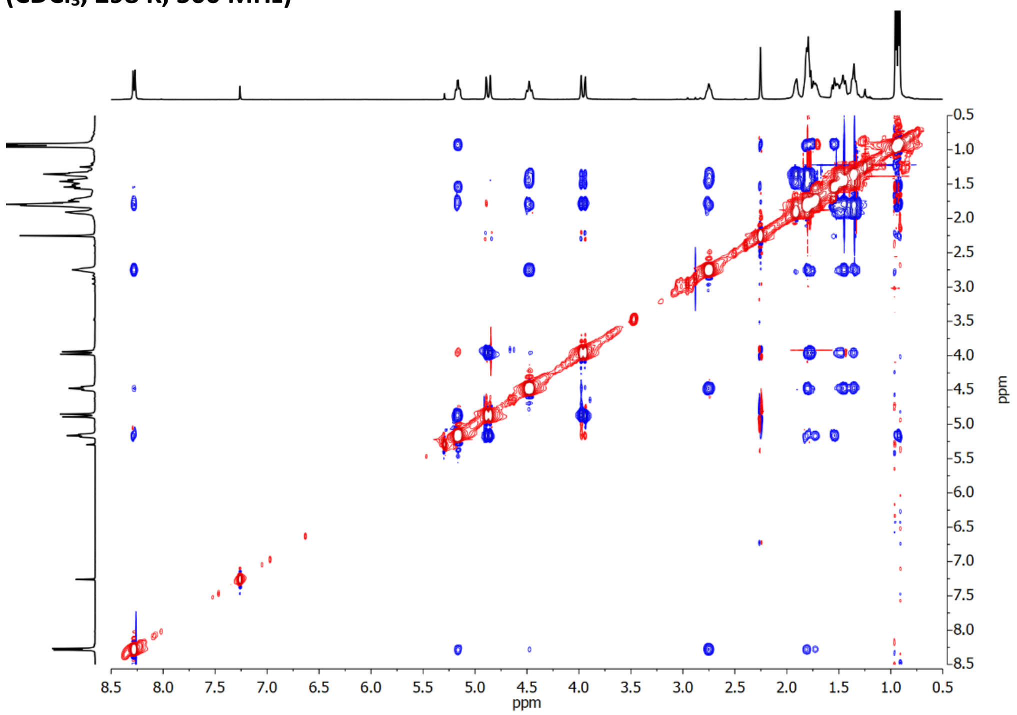

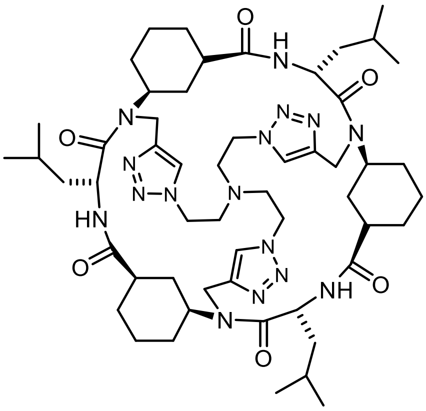

D4

HPLC-CROMATOGRAM

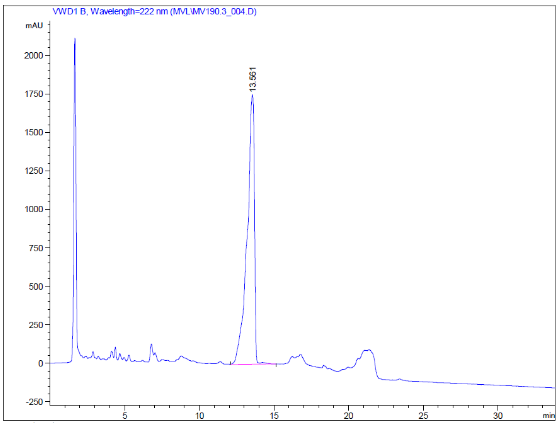

IR

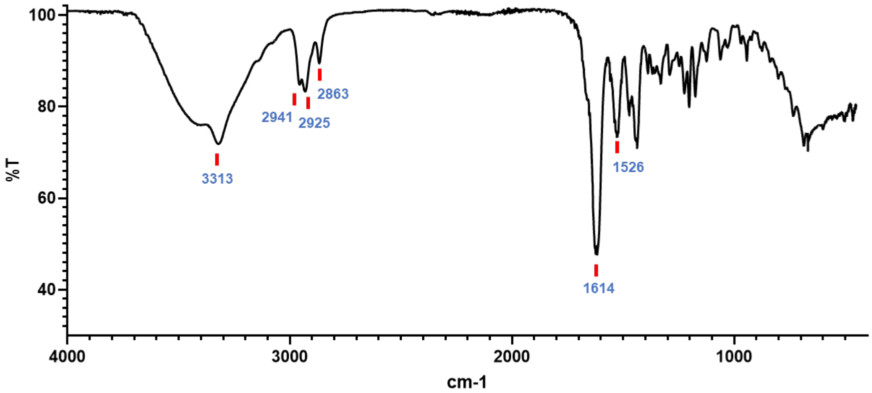

^{1}H-NMR (CD_2Cl_2, 298 K, 500 MHz)

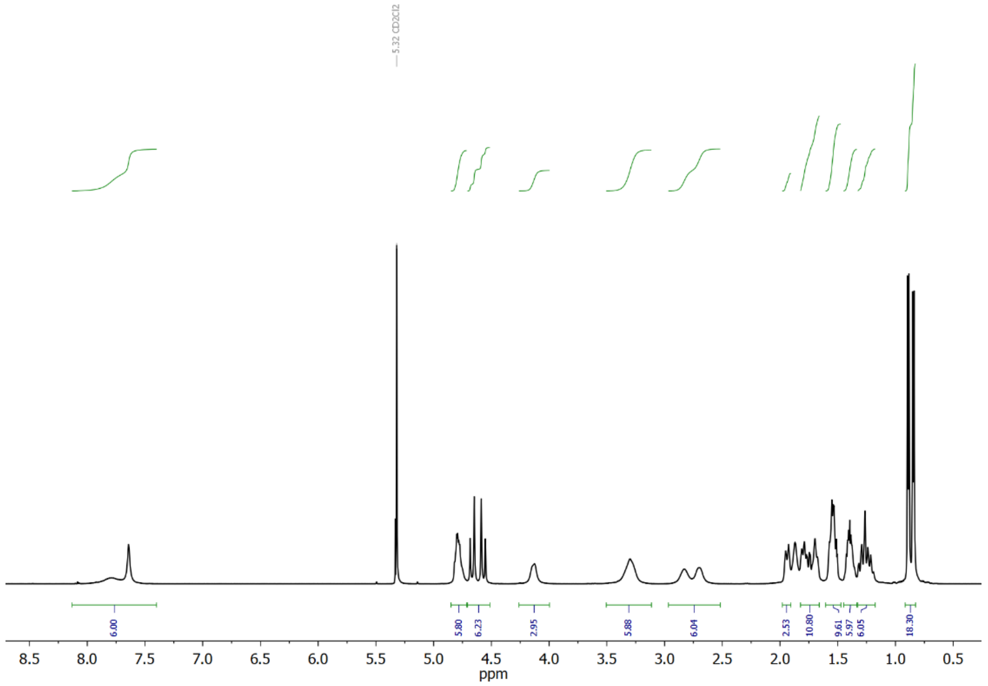

^{13}C-NMR (CD_2Cl_2, 298 K, 126 MHz)

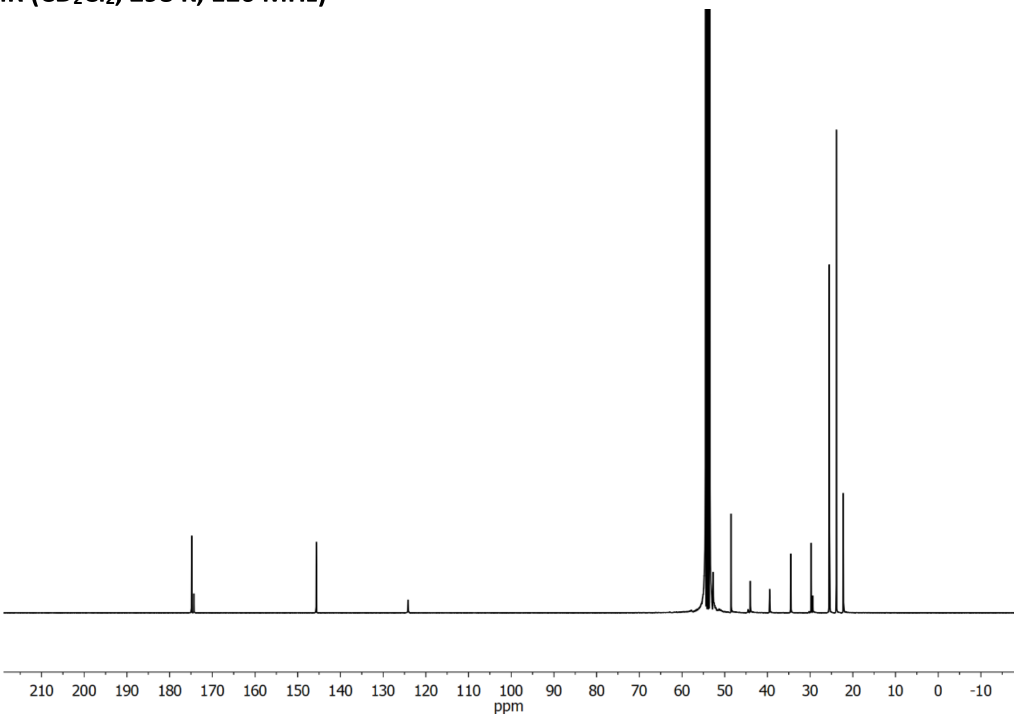

COSY (CD_2Cl_2, 298 K, 500 MHz)

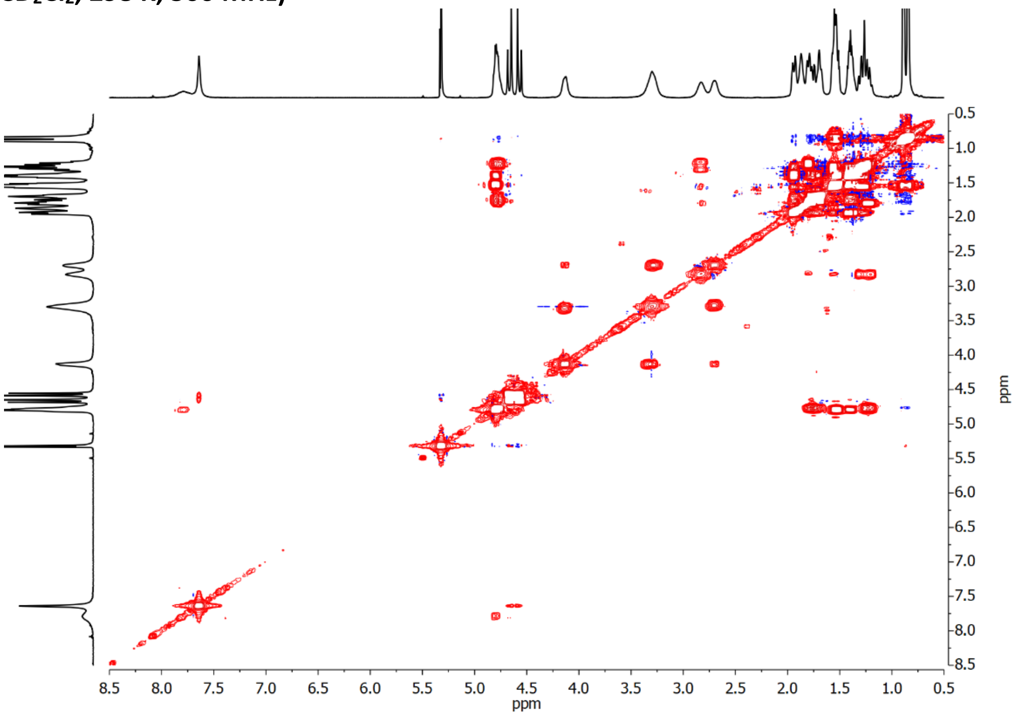

TOCSY (CD_2Cl_2, 298 K, 500 MHz)

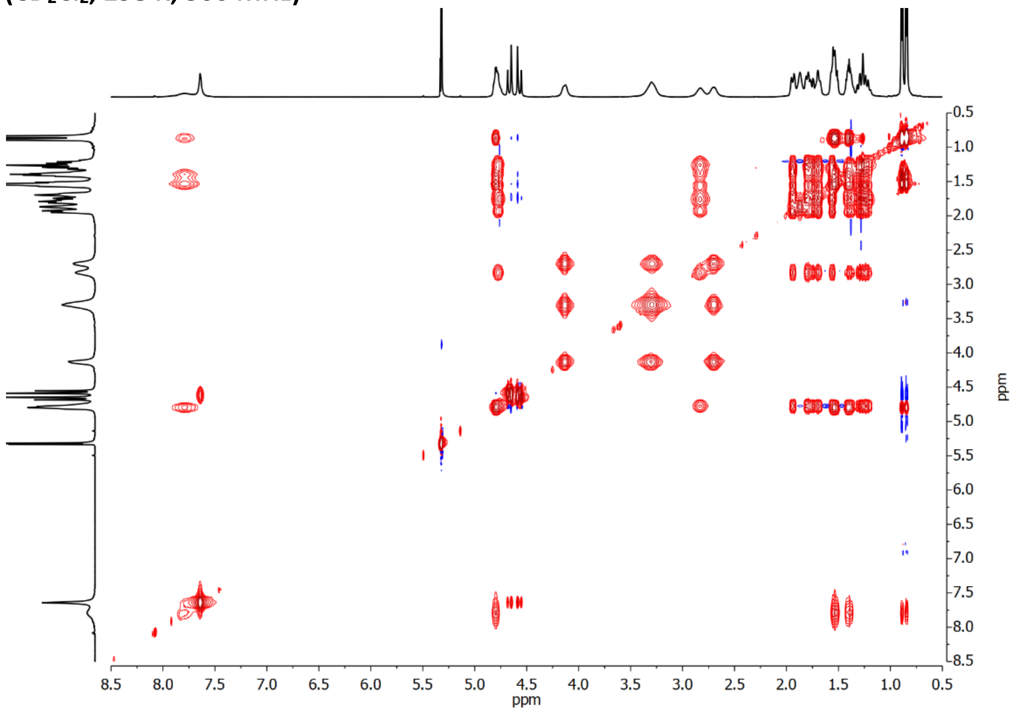

ROESY (CD_2Cl_2, 298 K, 500 MHz)

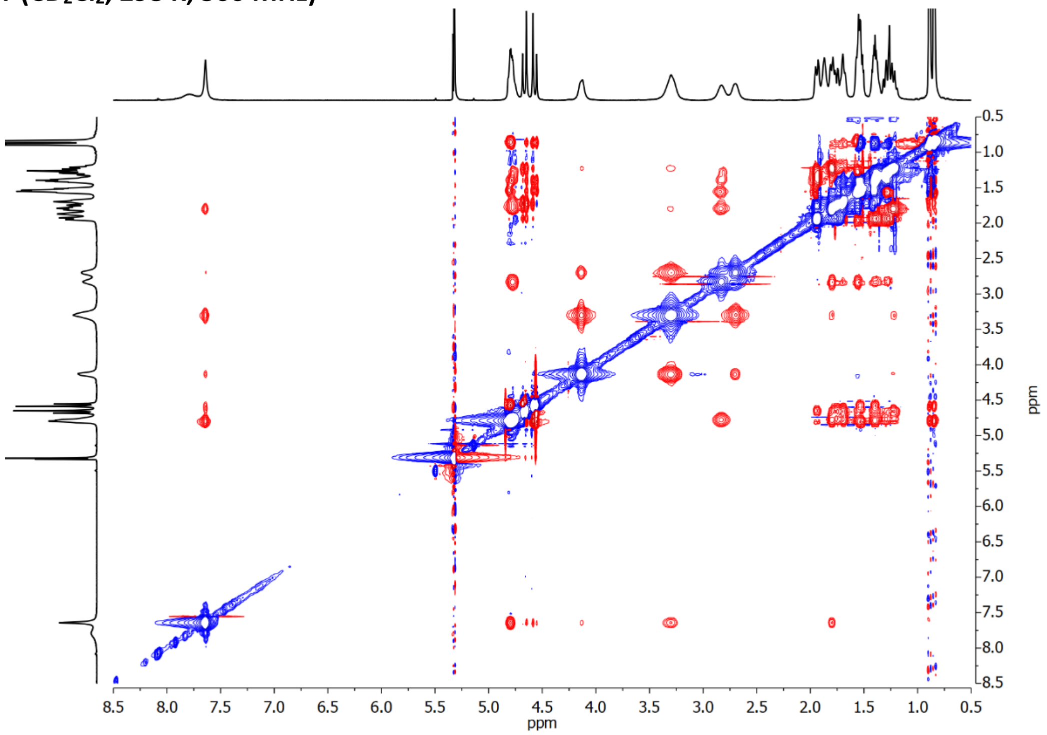

HSQC (CD_2Cl_2, 298 K, 500 MHz)

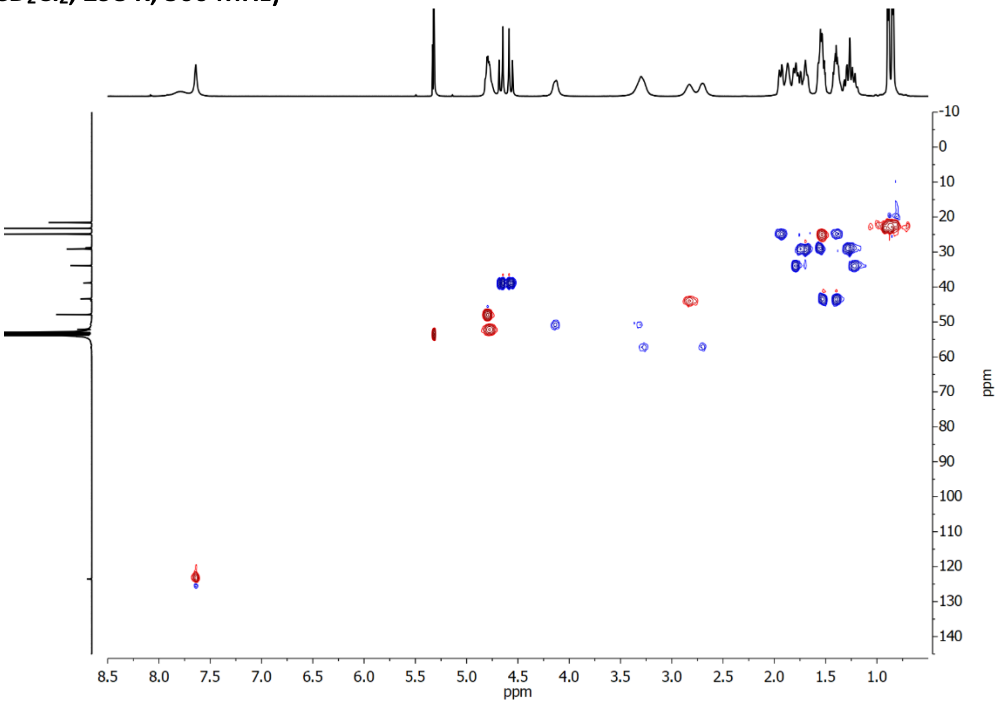

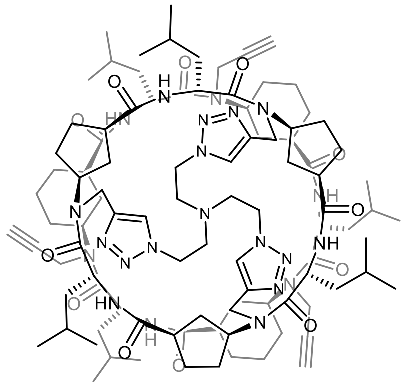

D2-3

^{1}H-NMR (CD_2Cl_2, 298 K, 500 MHz)

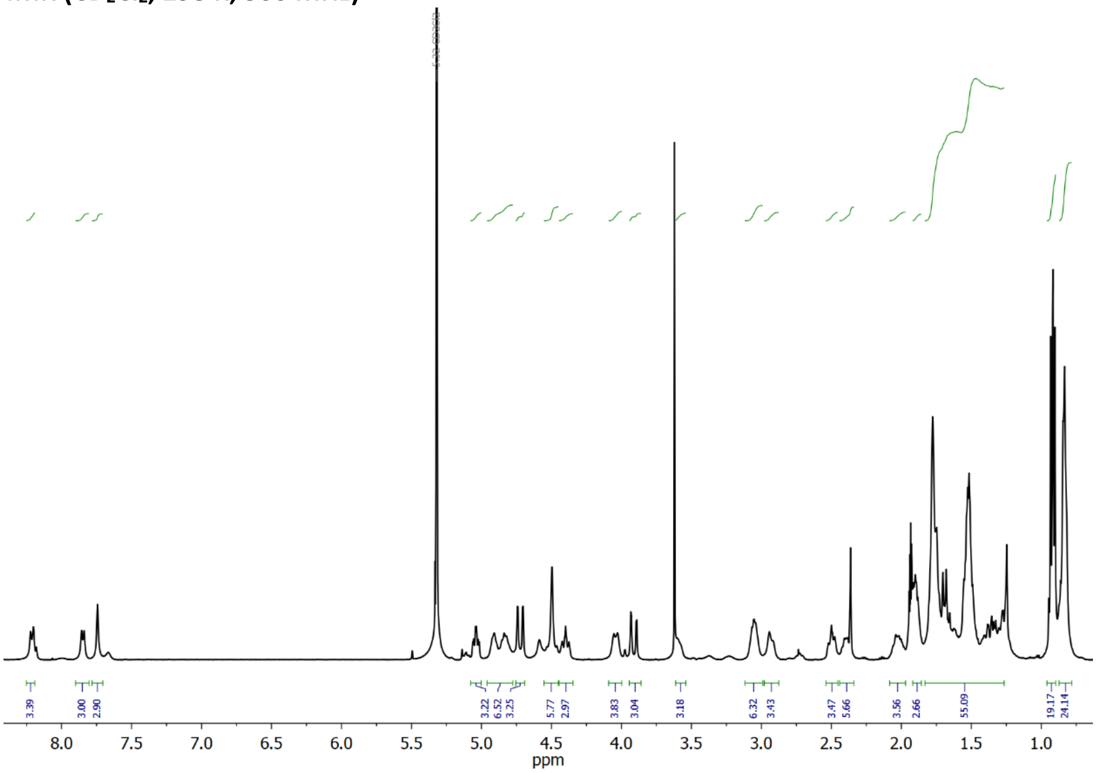

^{13}C and DEPT NMR (CD_2Cl_2, 298 K, 126 MHz)

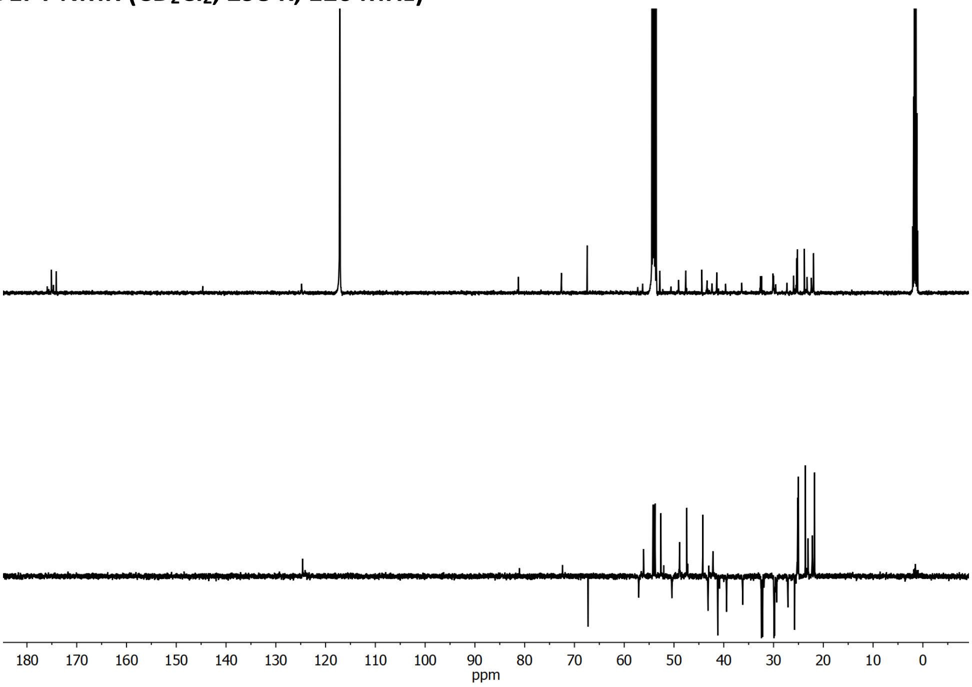

COSY (CD_2Cl_2, 298 K, 500 MHz)

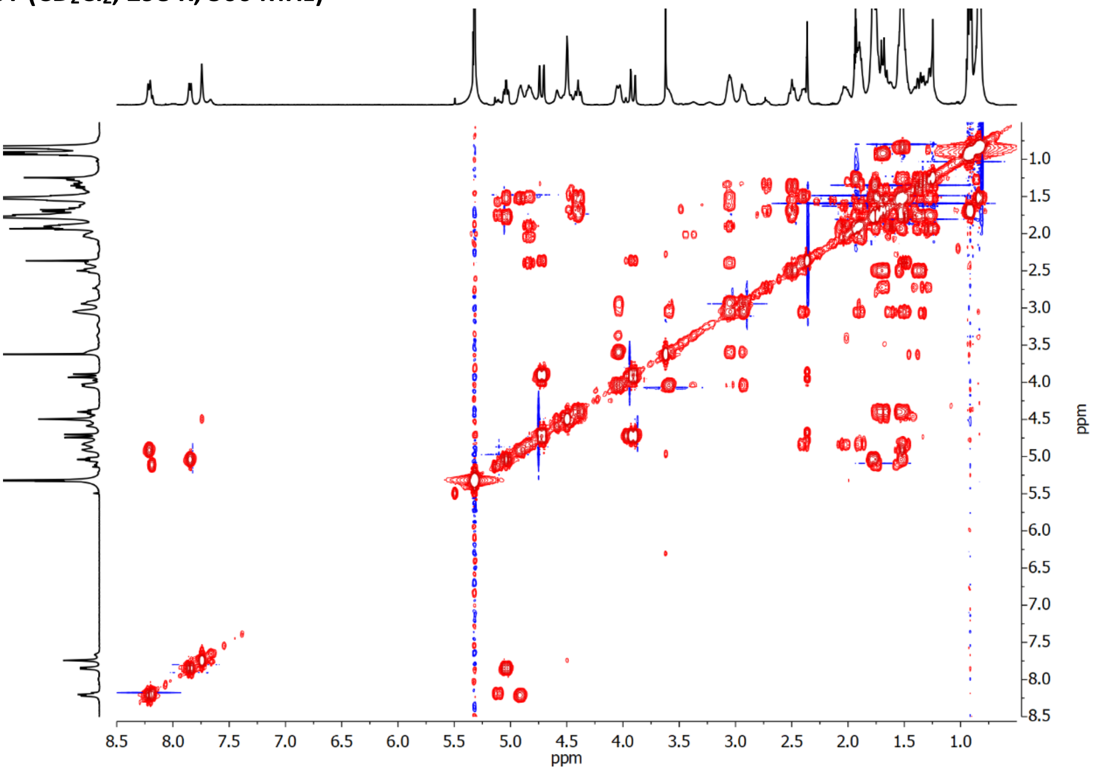

TOCSY (CD_2Cl_2, 298 K, 500 MHz)

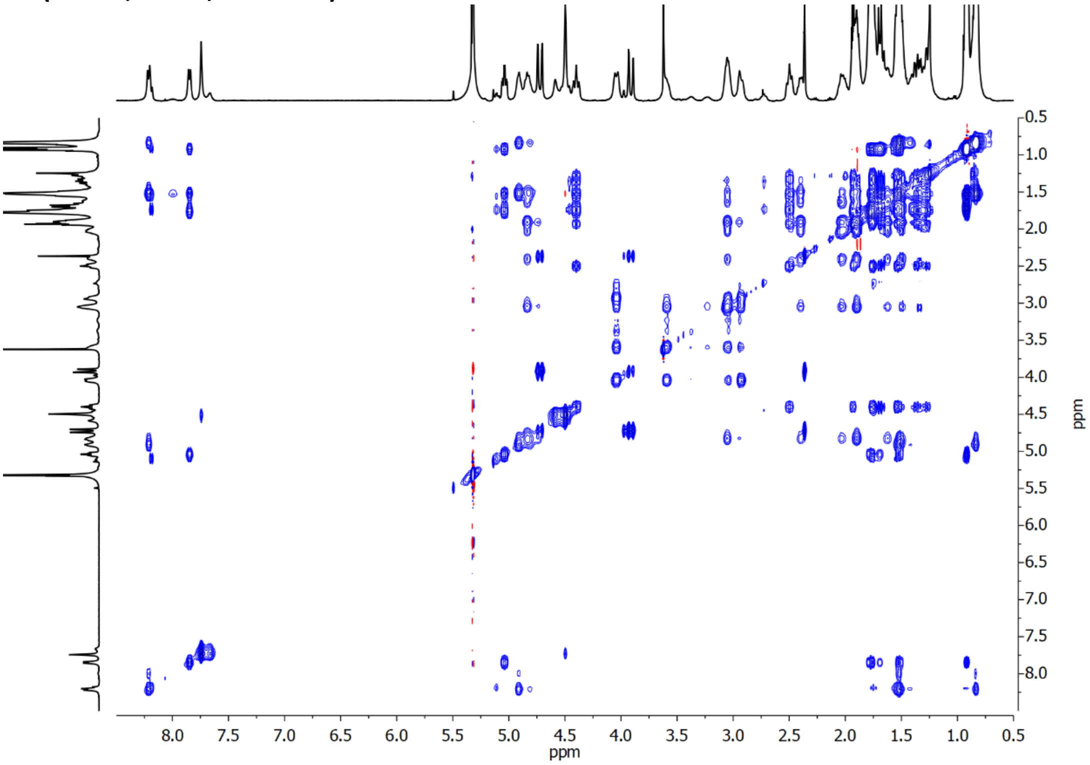

ROESY (CD_2Cl_2, 298 K, 500 MHz)

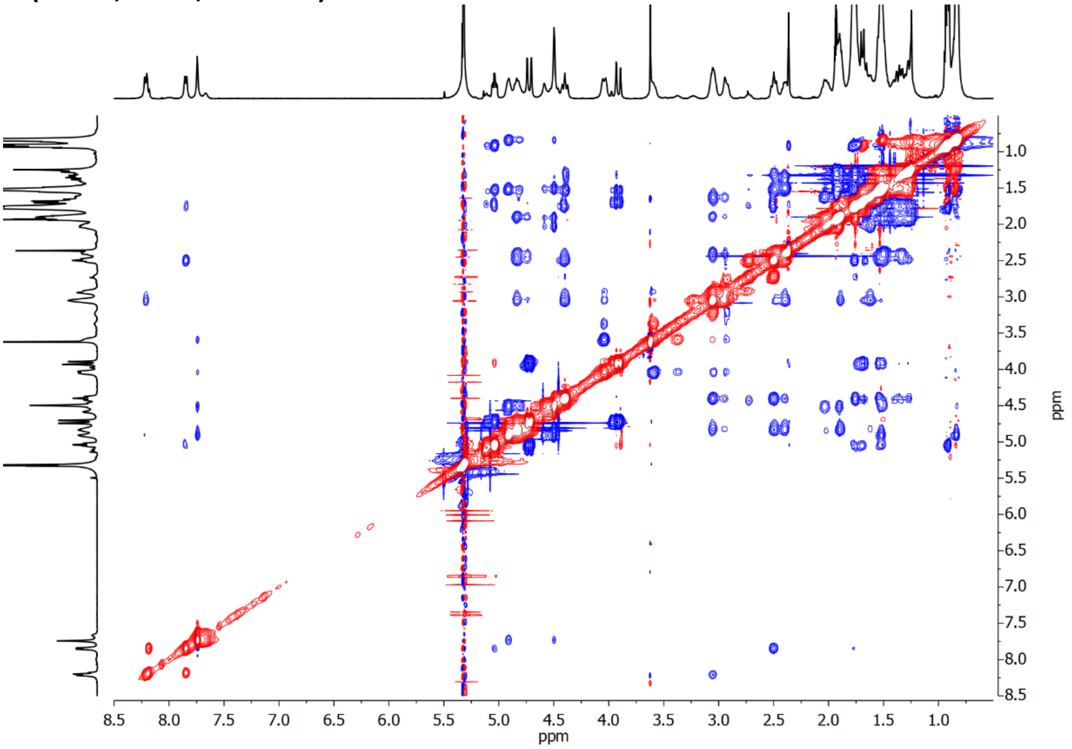

HSQC (CD_2Cl_2, 298 K, 500 MHz)

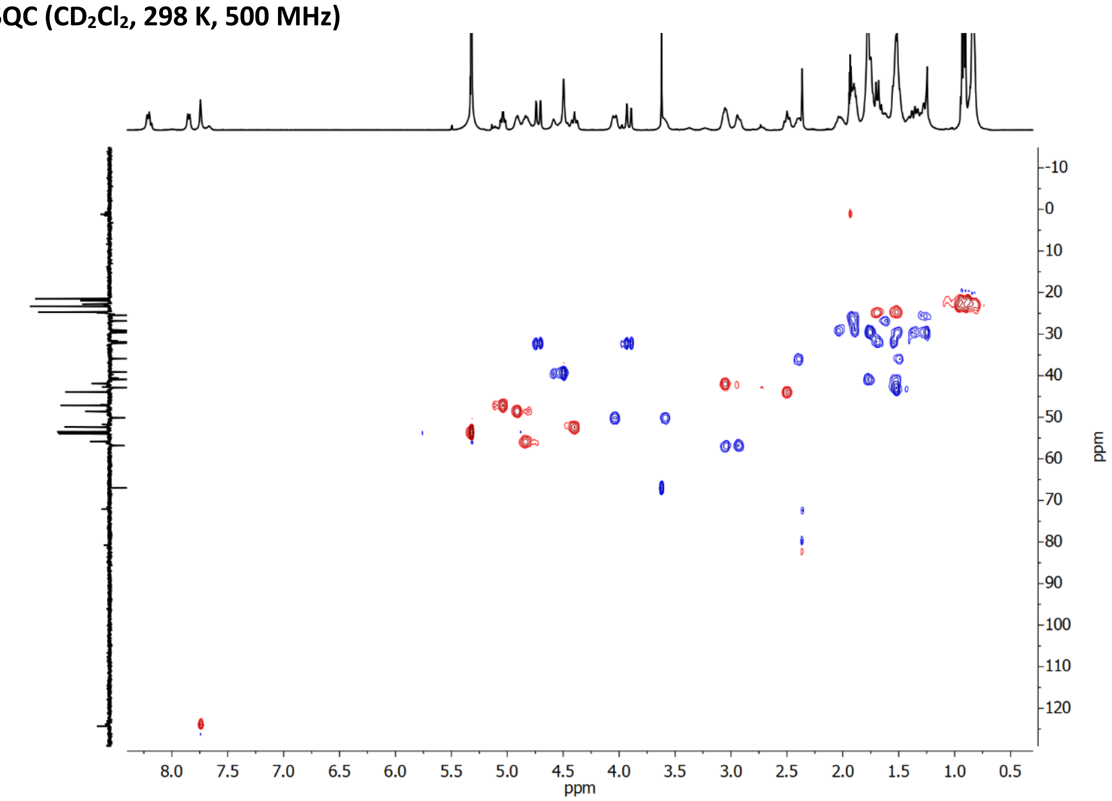

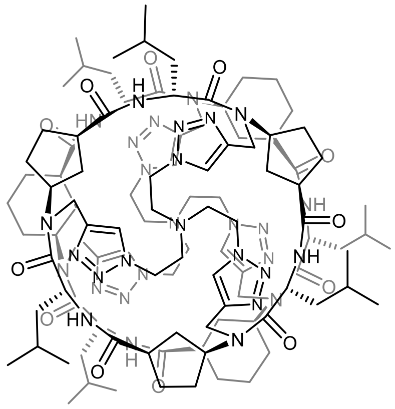

D2-4

^{1}H-NMR (CD_2Cl_2, 298 K, 500 MHz)

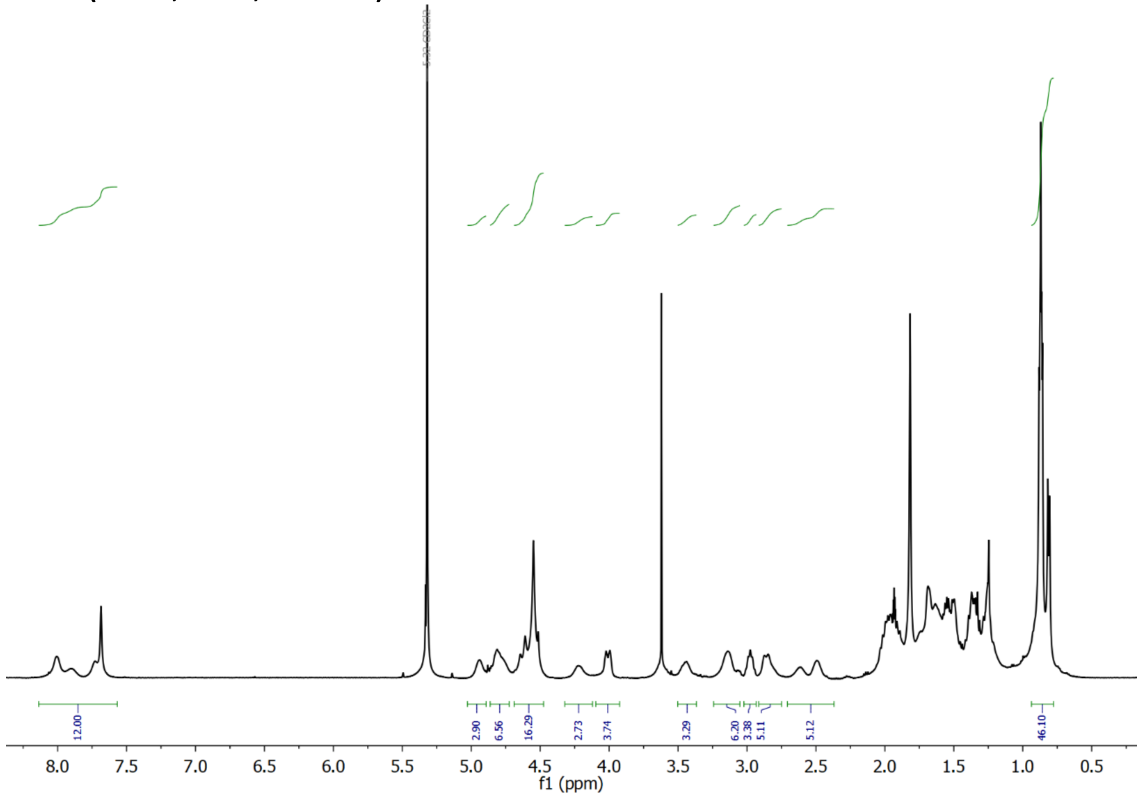

^{13}C and DEPT NMR (CD_2Cl_2, 298 K, 126 MHz)

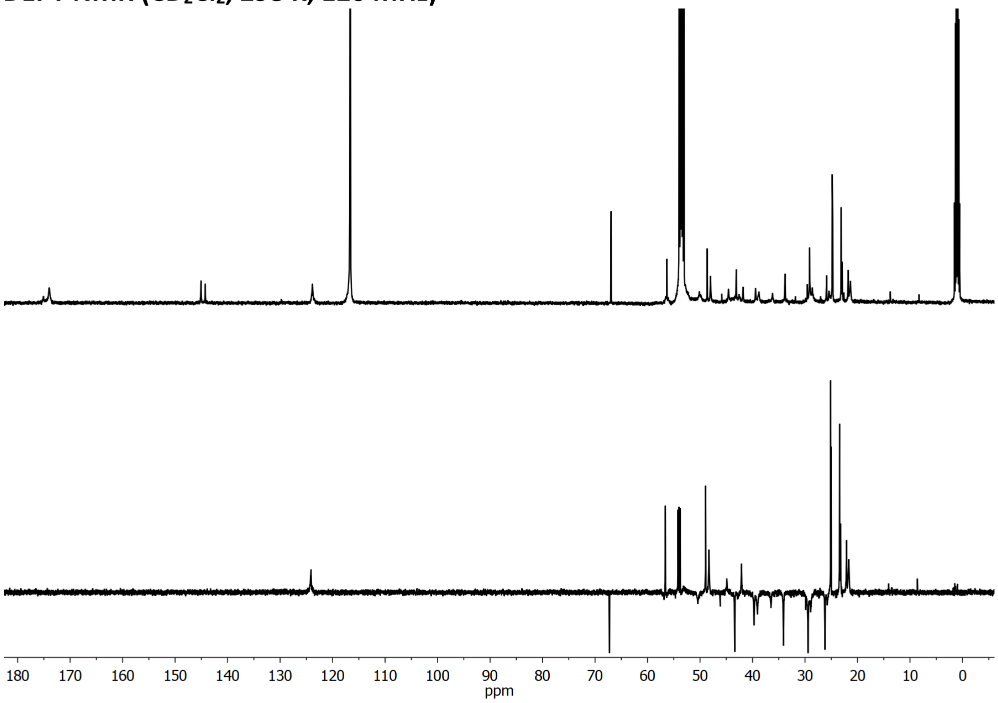

COSY (CD_2Cl_2, 298 K, 500 MHz)

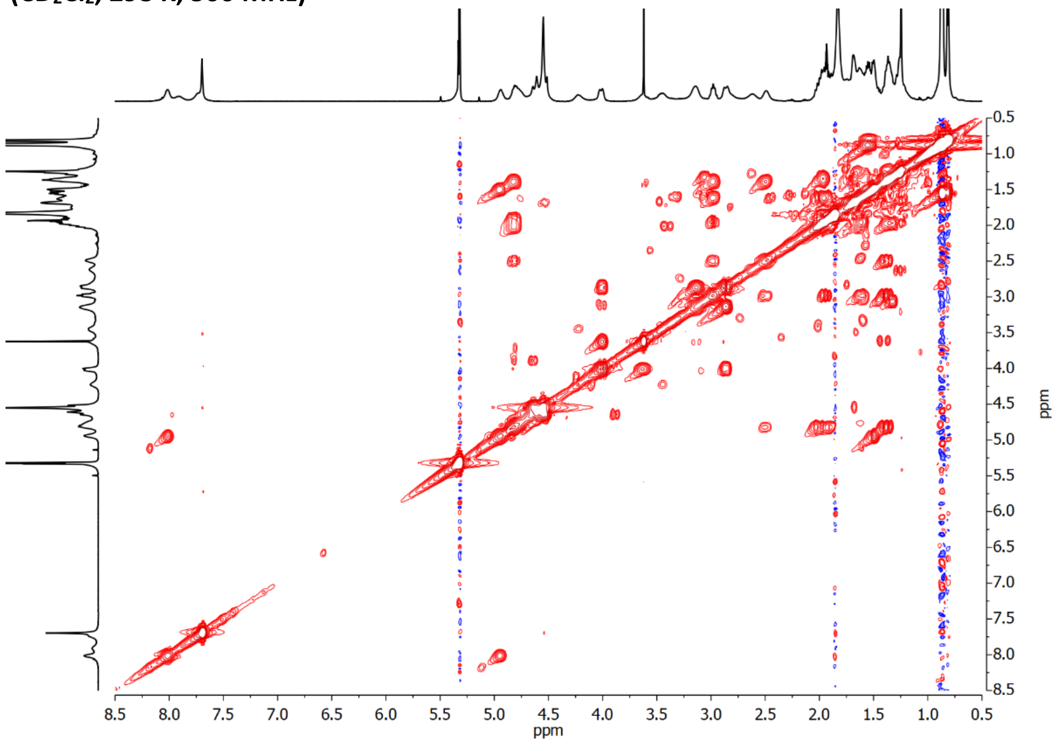

TOCSY (CD_2Cl_2, 298 K, 500 MHz)

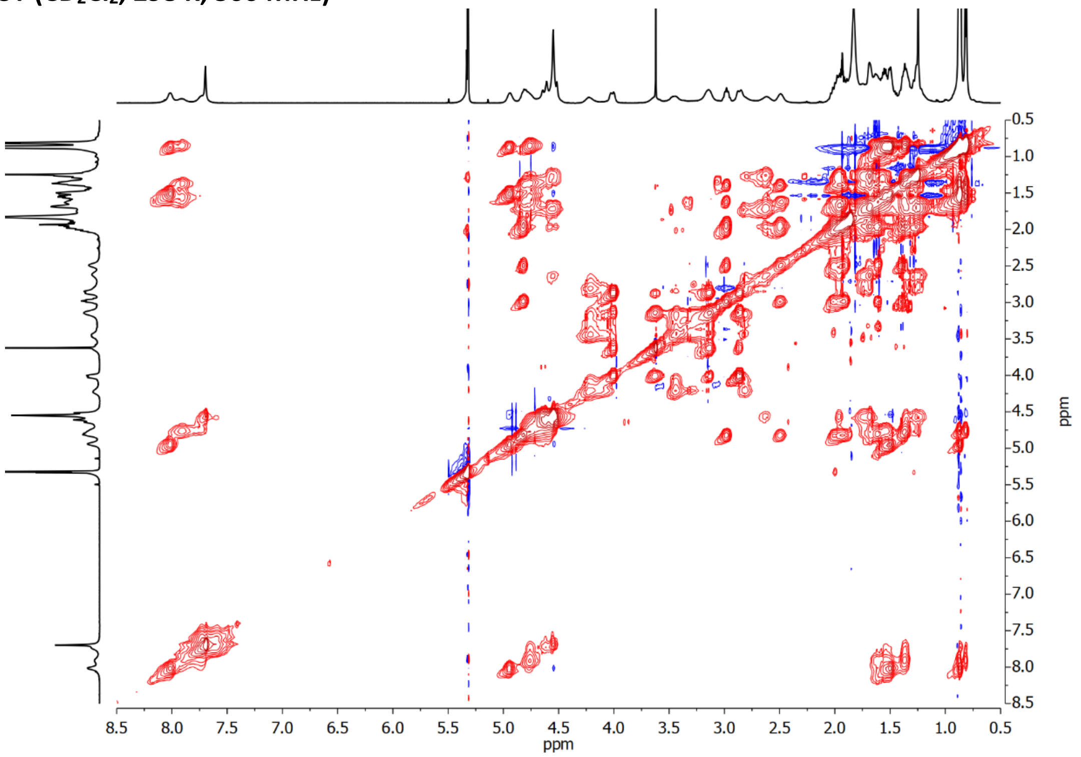

ROESY (CD_2Cl_2, 298 K, 500 MHz)

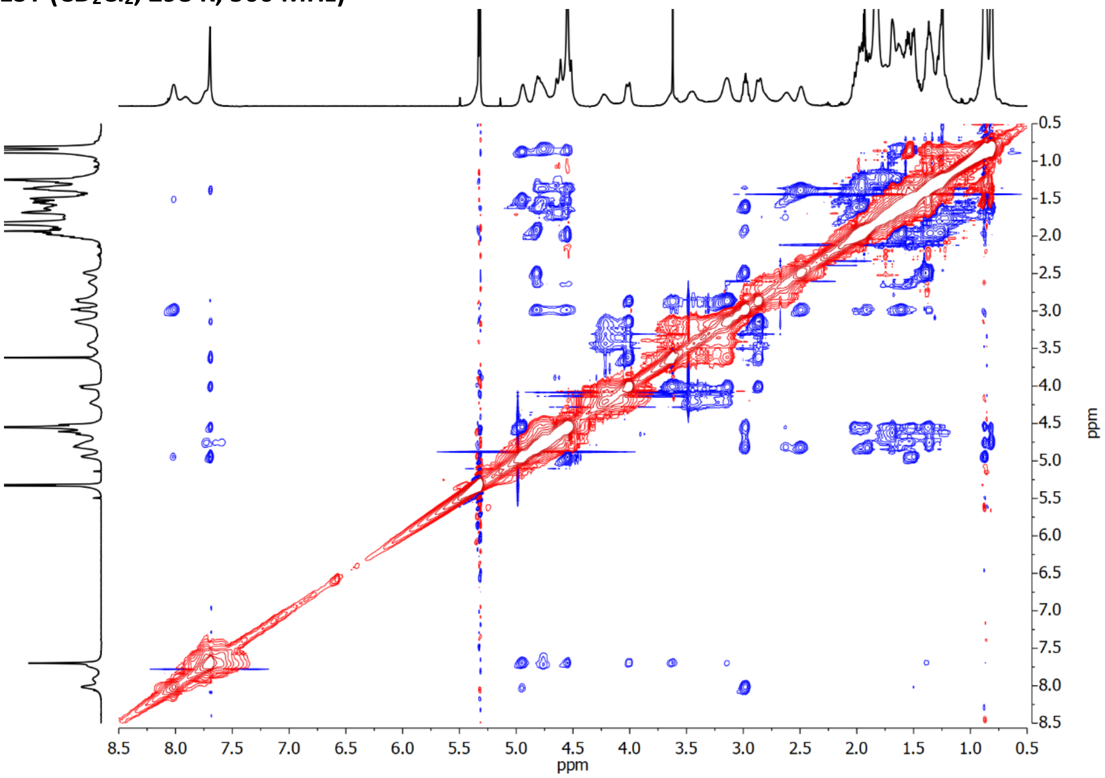

HSQC (CD_2Cl_2, 298 K, 500 MHz)

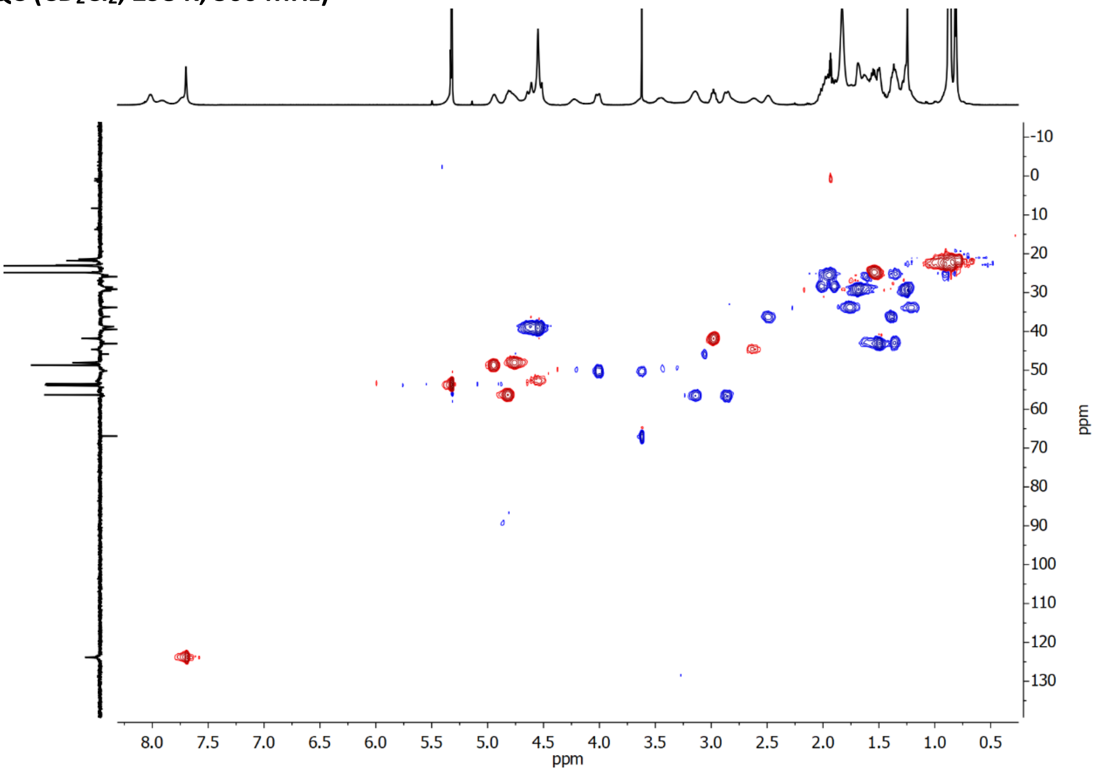

9. EXPERIMENTAL CRYSTAL STRUCTURE DETERMINATION

Supplementary Table 3. **Summary of the experimental crystal structure determination for ACN⊂D2 (CCDC-2311118).**

Crystal data

$2(C_{51}H_{78}N_{16}O_6)\cdot 2(C_2H_3N)\cdot 3(H_2O)$ [+solvent]	$F(000) = 4648$
$M_r = 2158.73$	$D_x = 1.05$ Mg m^{-3}
Orthorhombic, $P2_12_12_1$	Cu $K\alpha$ radiation, $\lambda = 1.54178$ Å
Hall symbol: P 2ac 2ab	Cell parameters from 9897 reflections
$a = 19.0634$ (10) Å	$\theta = 2.7$–$49.9°$
$b = 23.1346$ (11) Å	$\mu = 0.59$ mm^{-1}
$c = 30.9593$ (15) Å	$T = 100$ K
$V = 13653.8$ (12) Å^3	Plate, colourless
$Z = 4$	0.20 × 0.15 × 0.07 mm

Data collection

Bruker D8 VENTURE PHOTON III-14 diffractometer	16658 independent reflections
Radiation source: INCOATEC microfocus sealed tube, Incoatec IµS 3.0	12631 reflections with $I > 2\sigma(I)$
Incoatec multilayer mirror monochromator	$R_{int} = 0.157$
Detector resolution: 7.3910 pixels mm^{-1}	$\theta_{max} = 54.2°$, $\theta_{min} = 2.4°$
ω and phi scans	$h = -20\rightarrow 20$
Absorption correction: multi-scan BRUKER *SADABS2016*/2	$k = -24\rightarrow 24$
$T_{min} = 0.798$, $T_{max} = 0.973$	$l = -32\rightarrow 32$
168870 measured reflections	

Refinement

Refinement on F^2	Secondary atom site location: dual
Least-squares matrix: full	Hydrogen site location: mixed
$R[F^2 > 2\sigma(F^2)] = 0.113$	H atoms treated by a mixture of independent and constrained refinement
$wR(F^2) = 0.329$	$w = 1/[\sigma^2(F_o^2) + (0.2P)^2]$ where $P = (F_o^2 + 2F_c^2)/3$
$S = 1.28$	$(\Delta/\sigma)_{max} = 0.002$
16658 reflections	$\Delta\rangle_{max} = 0.45$ e Å^{-3}
1432 parameters	$\Delta\rangle_{min} = -0.26$ e Å^{-3}
3125 restraints	Absolute structure: Flack x determined using 4395 quotients [(I+)-(I-)]/[(I+)+(I-)] (Parsons, Flack and Wagner, Acta Cryst. B69 (2013) 249-259).
0 constraints	Absolute structure parameter: 0.27 (12)
Primary atom site location: dual	

Supplementary Table 4. Summary of the experimental crystal structure determination for ACN⊂D4 (CCDC-2311116).

Crystal data

$2(C_{54}H_{84}N_{16}O_6)\cdot C_2H_3N\cdot 3(H_2O)$ [+solvent]	$D_x = 1.037$ Mg m^{-3}
$M_r = 2201.84$	Cu $K\alpha$ radiation, $\lambda = 1.54178$ Å
Hexagonal, $P6_322$	Cell parameters from 9898 reflections
$a = 18.6252$ (7) Å	$\theta = 3.3–61.1°$
$c = 23.4693$ (13) Å	$\mu = 0.57$ mm^{-1}
$V = 7050.7$ (7) Å^3	$T = 100$ K
$Z = 2$	Block, clear colourless
$F(000) = 2376$	0.30 × 0.21 × 0.19 mm

Data collection

Bruker D8 VENTURE PHOTON-III C14 diffractometer	4242 independent reflections
Radiation source: microfocus sealed tube, Incoatec IμS 3.0	3392 reflections with $I > 2\sigma(I)$
Multilayer mirror monochromator	$R_{int} = 0.045$
Detector resolution: 7.3910 pixels mm^{-1}	$\theta_{max} = 67.4°$, $\theta_{min} = 5.1°$
φ or ω oscillation scans	$h = -20 \rightarrow 22$
Absorption correction: multi-scan Krause, L., Herbst-Irmer, R., Sheldrick, G. M., Stalke, D. (2015). "Comparison of silver and molybdenum microfocus X-ray sources for single-crystal structure determination" J. Appl. Cryst. 48, 3-10. doi:10.1107/S1600576714022985	$k = -22 \rightarrow 22$
$T_{min} = 0.69$, $T_{max} = 0.90$	$l = -28 \rightarrow 28$
103061 measured reflections	

Refinement

Refinement on F^2	Secondary atom site location: difference Fourier map
Least-squares matrix: full	Hydrogen site location: mixed
$R[F^2 > 2\sigma(F^2)] = 0.094$	H atoms treated by a mixture of independent and constrained refinement
$wR(F^2) = 0.331$	$w = 1/[\sigma^2(F_o^2) + (0.2P)^2]$ where $P = (F_o^2 + 2F_c^2)/3$
$S = 1.47$	$(\Delta/\sigma)_{max} < 0.001$
4242 reflections	$\Delta\rangle_{max} = 0.64$ e Å^{-3}
280 parameters	$\Delta\rangle_{min} = -0.51$ e Å^{-3}
429 restraints	Absolute structure: Flack x determined using 1209 quotients [(I+)-(I-)]/[(I+)+(I-)] (Parsons, Flack and Wagner, Acta Cryst. B69 (2013) 249-259).
Primary atom site location: dual	Absolute structure parameter: 0.69 (11)

Supplementary Table 5. Summary of the experimental crystal structure determination for ($F\cdot nH_2O$)⊂D2). (CCDC-2311117).

Crystal data

$4(C_{51}H_{78}N_{16}O_6)\cdot6(C_{16}H_{36}N)\cdot6F\cdot41.36O$	$F(000) = 6790$
$M_r = 6275.64$	$D_x = 1.144$ Mg m^{-3}
Monoclinic, $P2_1$	Cu $K\alpha$ radiation, $\lambda = 1.54178$ Å
$a = 17.9270$ (4) Å	Cell parameters from 9788 reflections
$b = 44.9593$ (11) Å	$\theta = 2.2$–$78.2°$
$c = 23.2733$ (6) Å	$\mu = 0.68$ mm^{-1}
$\beta = 103.8350$ (12)°	$T = 100$ K
$V = 18213.7$ (8) Å^3	Block, clear colourless
$Z = 2$	0.29 × 0.27 × 0.12 mm

Data collection

Bruker D8 VENTURE PHOTON-III C14 diffractometer	79341 independent reflections
Radiation source: microfocus sealed tube, Incoatec IμS 3.0	67636 reflections with $I > 2\sigma(I)$
Multilayer mirror monochromator	$R_{int} = 0.054$
Detector resolution: 7.3910 pixels mm^{-1}	$\theta_{max} = 79.2°$, $\theta_{min} = 2.0°$
ϕ or ω oscillation scans	$h = -22\rightarrow22$
Absorption correction: multi-scan Krause, L., Herbst-Irmer, R., Sheldrick, G. M., Stalke, D. (2015). "Comparison of silver and molybdenum microfocus X-ray sources for single-crystal structure determination" J. Appl. Cryst. 48, 3-10. doi:10.1107/S1600576714022985	$k = -55\rightarrow57$
$T_{min} = 0.83$, $T_{max} = 0.93$	$l = -29\rightarrow29$
1856239 measured reflections	

Refinement

Refinement on F^2	Secondary atom site location: difference Fourier map
Least-squares matrix: full	Hydrogen site location: mixed
$R[F^2 > 2\sigma(F^2)] = 0.082$	H atoms treated by a mixture of independent and constrained refinement
$wR(F^2) = 0.244$	$w = 1/[\sigma^2(F_o^2) + (0.161P)^2 + 7.7973P]$ where $P = (F_o^2 + 2F_c^2)/3$
$S = 1.02$	$(\Delta/\sigma)_{max} = 0.001$
79341 reflections	$\Delta\rangle_{max} = 0.79$ e Å^{-3}
4769 parameters	$\Delta\rangle_{min} = -0.50$ e Å^{-3}
7514 restraints	Absolute structure: Flack x determined using 26999 quotients [(I+)-(I-)]/[(I+)+(I-)] (Parsons, Flack and Wagner, Acta Cryst. B69 (2013) 249-259).
Primary atom site location: dual	Absolute structure parameter: 0.07 (4)

Supplementary Table 6. Summary of the experimental crystal structure determination for (Cl·nH_2O)⊂D2 (CCDC-2311119).

Crystal data

$2(C_{51}H_{78}N_{16}O_6)\cdot4(C_{48}H_{72}N_6O_6)\cdot3(C_{16}H_{36}N)\cdot2(C_4H_8O_2)\cdot3(Cl)\cdot3.828(H2\ O)$ [+solvent]	$D_x = 1.094$ Mg m^{-3}
$M_r = 6417.90$	Cu $K\alpha$ radiation, $\lambda = 1.54178$ Å
Trigonal, $R32$	Cell parameters from 9273 reflections
$a = 27.4008$ (5) Å	$\theta = 2.7–78.3°$
$c = 44.9581$ (10) Å	$\mu = 0.76$ mm^{-1}
$V = 29232.4$ (12) Å^3	$T = 100$ K
$Z = 3$	Block, clear colourless
$F(000) = 10471$	0.20 × 0.18 × 0.10 mm

Data collection

Bruker D8 VENTURE PHOTON-III C14 diffractometer	14102 independent reflections
Radiation source: microfocus sealed tube, Incoatec IμS 3.0	13202 reflections with $I > 2\sigma(I)$
Multilayer mirror monochromator	$R_{int} = 0.069$
Detector resolution: 7.3910 pixels mm^{-1}	$\theta_{max} = 79.4°$, $\theta_{min} = 3.2°$
ϕ or ω oscillation scans	$h = -34 \rightarrow 34$
Absorption correction: multi-scan Krause, L., Herbst-Irmer, R., Sheldrick, G. M., Stalke, D. (2015). "Comparison of silver and molybdenum microfocus X-ray sources for single-crystal structure determination" J. Appl. Cryst. 48, 3-10. doi:10.1107/S1600576714022985	$k = -34 \rightarrow 34$
$T_{min} = 0.86$, $T_{max} = 0.93$	$l = -54 \rightarrow 57$
449147 measured reflections	

Refinement

Refinement on F^2	Secondary atom site location: difference Fourier map
Least-squares matrix: full	Hydrogen site location: mixed
$R[F^2 > 2\sigma(F^2)] = 0.052$	H atoms treated by a mixture of independent and constrained refinement
$wR(F^2) = 0.151$	$w = 1/[\sigma^2(F_o^2) + (0.094P)^2 + 28.8774P]$ where $P = (F_o^2 + 2F_c^2)/3$
$S = 1.03$	$(\Delta/\sigma)_{max} = 0.001$
14102 reflections	$\Delta\rangle_{max} = 0.63$ e Å^{-3}
743 parameters	$\Delta\rangle_{min} = -0.36$ e Å^{-3}
674 restraints	Absolute structure: Flack x determined using 5854 quotients [(I+)-(I-)]/[(I+)+(I-)] (Parsons, Flack and Wagner, Acta Cryst. B69 (2013) 249-259).
Primary atom site location: dual	Absolute structure parameter: -0.029 (14)

Computing details

Data collection: Bruker *APEX4* software[15]

Cell refinement: *SAINT* V8.40B (Bruker AXS LLC, 2019)[16]

Data reduction: *SAINT* V8.40B (Bruker AXS LLC, 2019)[16]

Absorption correction and scaling: SADABS[17] or TWINABS[17]

Program(s) used to solve structure: SHELXT 2018/2[18] or SHELXD[19]

Program(s) used to refine structure: *SHELXL2019*/2[20]

Absolute structure[21]

CCDC-2311116 to CCDC-2311119 contains the supplementary crystallographic data for all the single crystals studied in this paper. The data can be obtained free of charge from The Cambridge Crystallographic Data Centre via *https://www.ccdc.cam.ac.uk/structures*.

ACN-D2 (CCDC: 2311118)

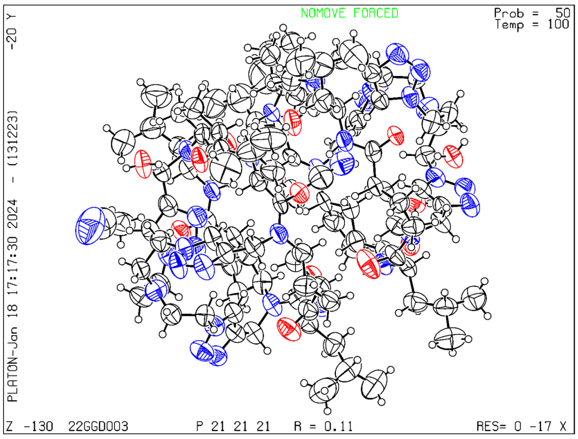

ACN-D4 (CCDC: 2311116)

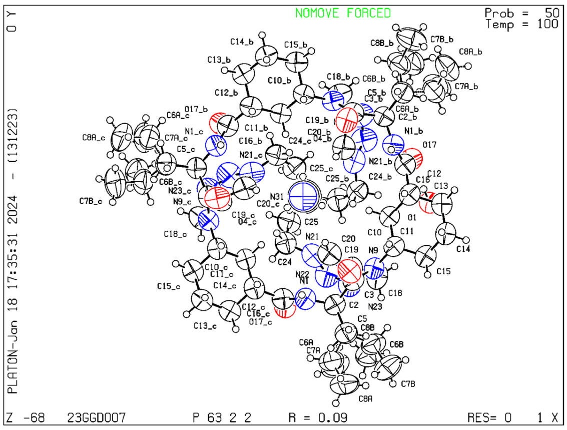

Cl-D2 (CCDC: 2311119)

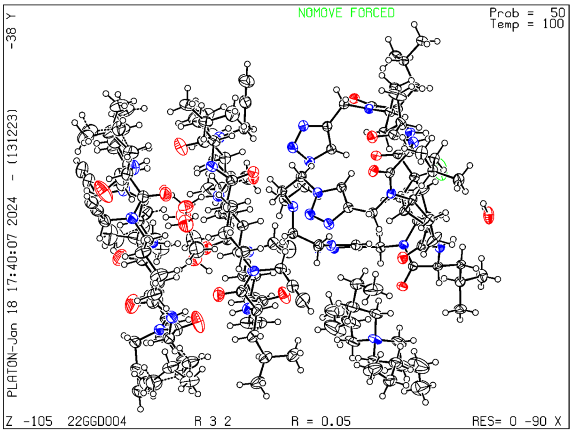

F-D2 (CCDC: 2311117)

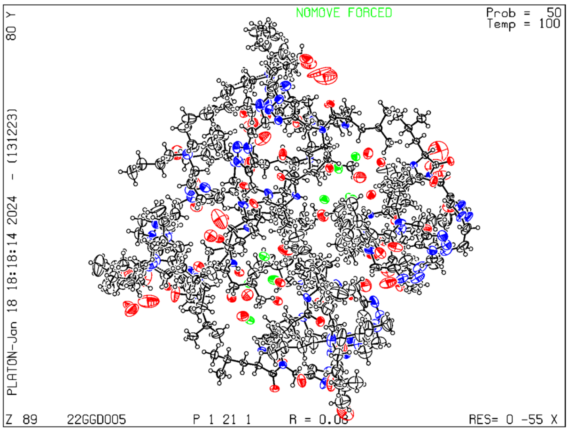

Supplementary Fig. 50. ORTEP diagrams of the four crystal structures presented in this work. The CCDC code is also indicated. Notice that **Cl-D2** correspond to **3Cl$^-$·4H$_2$O⊂2CP2**, **F-D2** correspond to **3F$^-$·8H$_2$O⊂2CP2**, **ACN-D2** correspond to **ACN⊂D2** and **ACN-D4** correspond to **ACN⊂D4**.

10. SUPPLEMENTARY REFERENCES

1. Jiménez, M. A., Nieto, J. L., Herranz, J., Rico, M. & Santoro, J. 1H NMR and CD evidence of the folding of the isolated ribonuclease 50-61 fragment. *FEBS Lett.* **221**, 320-324 (1987).
2. Sharman, G. J., Griffiths-Jones, S. R., Jourdan, M. & Searle, M. S. Effects of amino acid ϕ, ψ propensities and secondary structure interactions in modulating Hα chemical shifts in peptide and protein β-sheet. *J. Am. Chem. Soc.* **123**, 12318-12324 (2001).
3. Avbelj, F., Kocjan, D. & Baldwin, R. L. Protein chemical shifts arising from α-helices and β-sheets depend on solvent exposure. *Proc. Natl. Acad. Sci. U S A* **101**, 17394-17397 (2004).
4. Qi, R., Luo, Y., Ma, B., Nussinov, R. & Wei, G. Conformational distribution and α-helix to β-sheet transition of human amylin fragment dimer. *Biomacromolecules* **15**, 122-131 (2014).
5. a) Becke, A. D. Density-functional thermochemistry. III. The role of exact exchange. *J. Chem. Phys.* **98**, 5648-5652 (1993); b) Lee, C., Yang, W. & Parr, R. G. Development of the Colle-Salvetti correlation-energy formula into a functional of the electron density. *Phys. Rev. B* **37**, 785-789 (1988); c) Grimme, S., Antony, J., Schwabe, T. & Mück-Lichtenfeld, C. Density functional theory with dispersion corrections for supramolecular structures, aggregates, and complexes of (bio)organic molecules. *Org. Biomol. Chem.* **5**, 741-758 (2007); d) Grimme, S., Antony, J., Ehrlich, S. & Krieg, H. A consistent and accurate ab initio parametrization of density functional dispersion correction (DFT-D) for the 94 elements H-Pu. *J. Chem. Phys.* **132**, 154104-154119 (2010); e) Grimme, S., Ehrlich, S. & Goerigk, L. Effect of the damping function in dispersion corrected density functional theory. *J. Comput. Chem.* **32**, 1456-1465 (2011).
6. a) Rahman, F. *et al.* Binding and Assembly of a Benzotriazole Cavitand in Water. *Angew. Chem. Int. Ed.* **61**, e2022055 (2022); Guan, H. W. *et al.* Recognition of hydrophilic molecules in deep cavitand hosts with water-mediated hydrogen bonds. *Chem. Commun.* **57**, 8147-8150 (2021); c) Daver, H., Rebek, J. & Himo, F. Modeling the Reaction of Carboxylic Acids and Isonitriles in a Self-Assembled Capsule. *Chem. Eur. J.* **26**, 10861-10870 (2020).
7. McNally, B. A., Koulov, A. V., Smith, B. D., Joos, J. B. & Davis, A. P. A fluorescent assay for chloride transport; identification of a synthetic anionophore with improved activity. *Chem. Commun.* 1087-1089 (2005).
8. Lisbjerg, M. *et al.* Biotin[6]uril esters: Chloride-selective transmembrane anion carriers employing C-H···anion interactions. *J. Am. Chem. Soc.* **137**, 4948-4951 (2015).
9. Gilchrist, A. M. *et al.* Supramolecular methods: the 8-hydroxypyrene-1,3,6-trisulfonic acid (HPTS) transport assay. *Supramol. Chem.* **33**, 325-344 (2021).
10. Amorín, M., Castedo, L. & Granja, J. R. New cyclic peptide assemblies with hydrophobic cavities: The structural and thermodynamic basis of a new class of peptide nanotubes. *J. Am. Chem. Soc.* **125**, 2844-2845 (2003).
11. Amorín, M., Brea, R. J., Castedo, L. & Granja, J. R. The Smallest α,γ-Peptide Nanotubulet Segments: Cyclic α,γ-Tetrapeptide Dimers. *Org. Lett.* **7**, 4681–4684 (2005).
12. Fuertes, A., Ozores, H. L., Amorín, M. & Granja, J. R. Self-assembling Venturi-like peptide nanotubes. *Nanoscale* **9**, 748–753 (2017).
13. Pizzi, A. *et al.* Tight Xenon Confinement in a Crystalline Sandwich-like Hydrogen-Bonded Dimeric Capsule of a Cyclic Peptide. *Angew. Chem., Int. Ed.* **58**, 14472-4476 (2019).
14. Goddard-Borger, E. D. & Stick, R. V. An efficient, inexpensive, and shelf-stable diazotransfer reagent: Imidazole-1-sulfonyl azide hydrochloride. *Org. Lett.* **9**, 3797-3800 (2007).
15. Bruker. *APEX4*. Bruker AXS LLC, Madison, WI, USA. (2022).
16. Bruker. *SAINT*. Bruker AXS LLC, Madison, WI, USA. (2022).
17. Krause, L., Herbst-Irmer, R., Sheldrick, G. M., Stalke, D. Comparison of silver and molybdenum microfocus X-ray sources for single-crystal structure determination. J. *Appl. Cryst*. **48**, 3-10. (2015).

18. Sheldrick, G. M. SHELXT--Integrated space-group and crystal-structure determination. *Acta Cryst*. A71, 3-8 (2015).
19. Sheldrick, G. M. A short history of SHELX. *Acta Cryst*. Section A. **64**, 112-122 (2008).
20. Sheldrick, G. M. Crystal structure refinement with SHELXL. *Acta Cryst*. C71, 3-8 (2015).
21. Parsons, Flack and Wagner. Use of intensity quotients and differences in absolute structure refinement. *Acta Cryst*. B69, 249-259 (2013).
